# Synthesis and Biological Application of Isosteviol-Based 1,3-Aminoalcohols

**DOI:** 10.3390/ijms222011232

**Published:** 2021-10-18

**Authors:** Dániel Ozsvár, Viktória Nagy, István Zupkó, Zsolt Szakonyi

**Affiliations:** 1 Interdisciplinary Excellence Center, Institute of Pharmaceutical Chemistry, University of Szeged, H-6720 Szeged, Hungary; ozsmozs88@gmail.com; 2 Department of Pharmacodynamics and Biopharmacy, University of Szeged, H-6720 Szeged, Hungary; nagy.viktoria07@gmail.com (V.N.); zupko.istvan@szte.hu (I.Z.); 3 Interdisciplinary Centre of Natural Products, University of Szeged, H-6720 Szeged, Hungary

**Keywords:** 1,3-aminoalcohol, isosteviol, antiproliferative activity, chiral pool, diterpene, Mannich

## Abstract

Starting from isosteviol, a series of diterpenoid 1,3-aminoalcohol derivatives were stereoselectively synthesised. The acid-catalysed hydrolysis and rearrangement of natural stevioside gave isosteviol, which was transformed to the key intermediate methyl ester. In the next step, Mannich condensation of diterpenoid ketone, paraformaldehyde, and secondary amines resulted in the formation of 1,3-aminoketones with different stereoselectivities. During the Mannich condensation with dibenzylamine, an interesting *N*-benzyl → *N*-methyl substituent exchange was observed. Reduction of 1,3-aminoketones produced diastereoisomeric 1,3-aminoalcohols. Alternatively, aminoalcohols were obtained via stereoselective hydroxy-formylation, followed by oxime preparation, reduction, and finally, reductive alkylation of the obtained primary aminoalcohols. An alternative 1,3-aminoalcohol library was prepared by reductive amination of the intermediate 3-hydroxyaldehyde obtained from isosteviol in two-step synthesis. Cytotoxic activity of compounds against human tumour cell lines (A2780, SiHa, HeLa, MCF-7 and MDA-MB-231) was investigated. In our preliminary study, the 1,3-aminoalcohol function and *N*-benzyl substitution seemed to be essential for the reliable antiproliferative activity. To extend their application, a diterpenoid condensed with 2-phenylimino-1,3-thiazine and -1,3-oxazine was also attempted to prepare, but only formation of thioether intermediate was observed.

## 1. Introduction

Terpenoids, also known as isoprenoids, are the most numerous and structurally diverse group of natural products present in most plants [1]. Several studies have confirmed that this class of compounds displays a wide array of very important pharmacological properties [2]. Between terpenoids, the diterpenoid stevioside with a complex *ent*-kaurane skeleton and three glucose moieties has been the focus of attention in recent decades [3,4]. Stevioside is extracted from the plant *Stevia rebaudiana*, which is a perennial herbal shrub of the *Asteraceae* family that originated from Brazil and Paraguay in South America, while cultivated for its sweet leaves [5]. It is applied in food chemistry as a commercial sweetener considered to be a non-caloric sugar substitute. In recent years, stevioside and steviol, its aglycon, have attracted scientific attention because of their broad spectrum of biological activities, including antihyperglycemic, [6] antihypertensive, [7,8] antitumour, [9,10] and immunomodulatory actions [11] beside several other biological activities [12,13].

Isosteviol, a structural isomer of steviol, is a tetracyclic diterpenoid with an *ent*-beyerane skeleton obtained by acidic hydrolysis of stevioside [14]. In recent years, isosteviol derivatives have drawn high interest because of their biological activities, including anti-inflammatory, [15] glucocorticoid agonist, [16] antibacterial [17], anticancer [18,19], or even cardioprotective properties [20].

Cytotoxic activities of isosteviol derivatives, obtained by microbial and chemical transformations, have also induced much attention in recent years [21,22]. Several of the novel isosteviol derivatives have been successfully synthesised by chemical modification of isosteviol, and some of these derivatives exhibited good cytotoxic activity as potential drug molecules. Li and co-workers reported compounds with an α-methylenecyclopentanone moiety in the D-ring of isosteviol displaying remarkable anticancer activity against MDA-MB-231 cell line with an IC_50_ value of 1.58 μM [23]. Jayachandra and co-workers synthesised isosteviol analogues showing a potential protective effect against DOX-induced cardiotoxicity in zebrafish embryos in vivo [24]. Tao and co-workers reported that *Esophageal* carcinoma cells were more sensitive to 1,3-aminoalcohols, exhibiting anticancer activities superior to Cisplatin with an IC_50_ value 4.01 μM [25].

1,3-Aminoalcohols may also serve as a building blocks of many natural and synthetic products, and they exhibit wide-ranging biological and catalytic activities [26,27,28]. In a previous work, Tao and co-workers prepared a series of compounds by modifying a crucial aminoalcohol fragment of the D-ring of isosteviol affording significantly improved anticancer activities [25]. In a similar study, a hydroxythiourea derivative has been described as a useful candidate for the treatment of tumours on different cell lines [29].

In the present contribution, we report the preparation of a new library of isosteviol-based chiral bifunctional synthons, such as β-aminoketones, 1,3-aminoalcohols, and 1,3-heterocycles fused with *ent*-beyerane, starting from commercially available natural stevioside. We also planned to investigate the preliminary study of the effect of keto-amine and 1,3-aminoalcohol functions and the stereochemistry and substitution level of amine function on antiproliferative activity on multiple human cancer cell lines.

## 2. Results and Discussion

### 2.1. Synthesis of Isosteviol-Based 1,3-Aminoalcohols

**Scheme 1:**
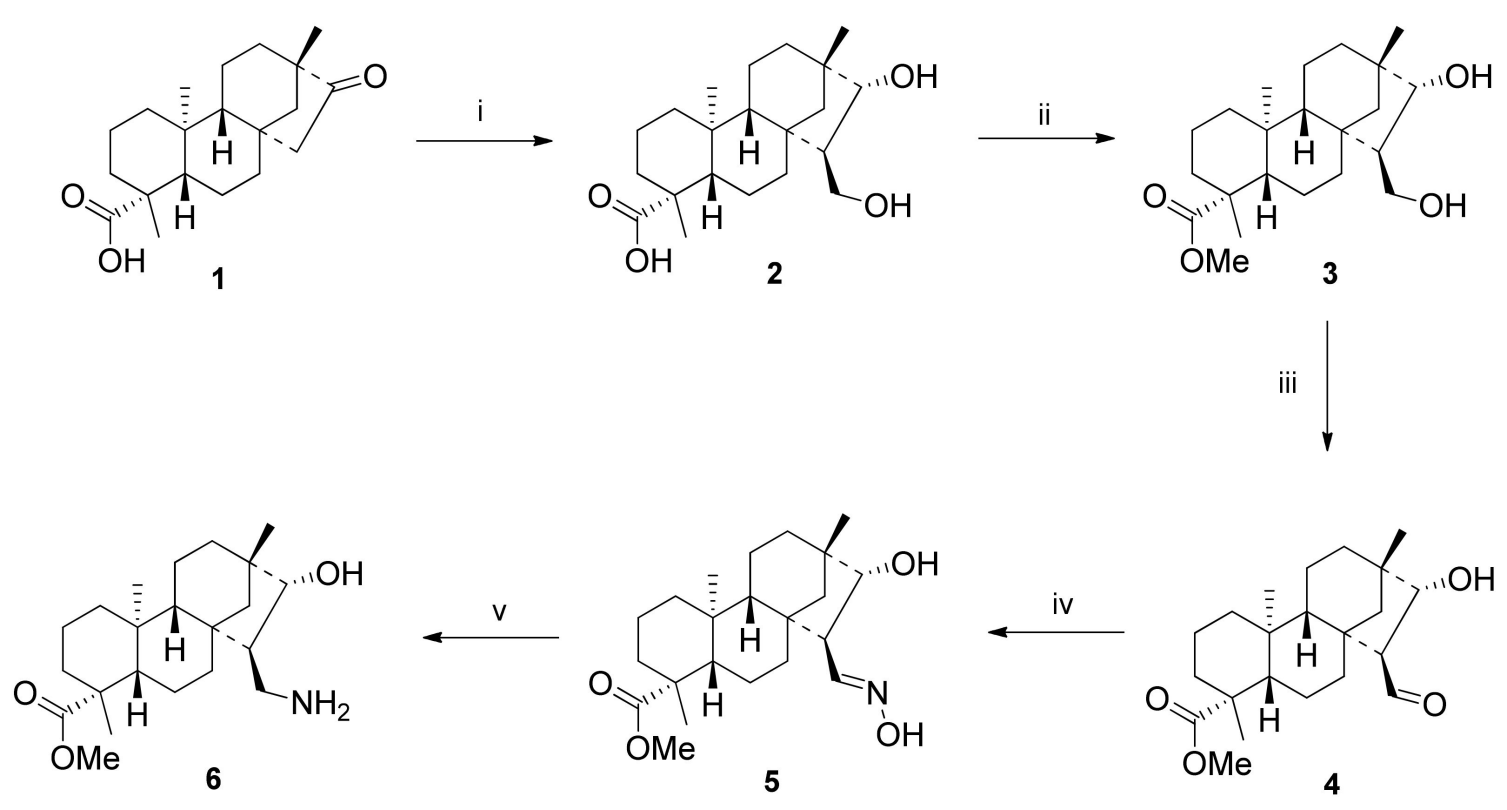
Stereoselective synthesis of isosteviol-based 1,3-aminoalcohol **6**. (i) HCHO, NaOH (2 eq.), EtOH,1 h, 60 °C, 70%; (ii) CH_2_N_2_, Et_2_O, 5 min, 25 °C, 72%; (iii) 10 mol% TEMPO, NCS (2 eq.), TBAB (1 eq.), DCM/H_2_O, 12 h, reflux, 90%; (iv) H_2_NOH-HCl (2 eq.), EtOH, 12 h, reflux, 76%; (v) Raney-Ni, H_2_ (10 atm), THF, 12 h, 25 °C, 83%.

Starting from commercially available stevioside or mixtures of steviol glycosides, key intermediates 3-hydroxyaldehyde **4** and primary 1,3-aminoalcohol **6** were prepared in a four- and six-step synthesis (Scheme 1). Isosteviol **1** was obtained from commercially available natural stevioside by acid-catalysed hydrolysis and rearrangement [30].

Diol **2** was synthesised in a stereoselective manner with good yield in a one-pot *Aldol-Cannizzaro* process in two steps according to literature methods [31,32]. Esterification of **2** was carried out with diazomethane in Et_2_O resulting in methyl ester **3** [31]. The TBAB-catalysed oxidisation of **3** with TEMPO and NCS gave regioselectively **4** in excellent yield. In the next step, compound **5** was obtained via oximation of **4** with hydroxylamine hydrochloride in the presence of NaHCO_3_ in ethanol and then it was converted to 1,3-aminoalcohol **6** with hydrogenation catalysed by Raney-Ni in THF in good yield (Scheme 1).

### 2.2. Synthesis of Isosteviol-Based 1,3-Aminoalcohols via Schiff Bases

**Scheme 2:**
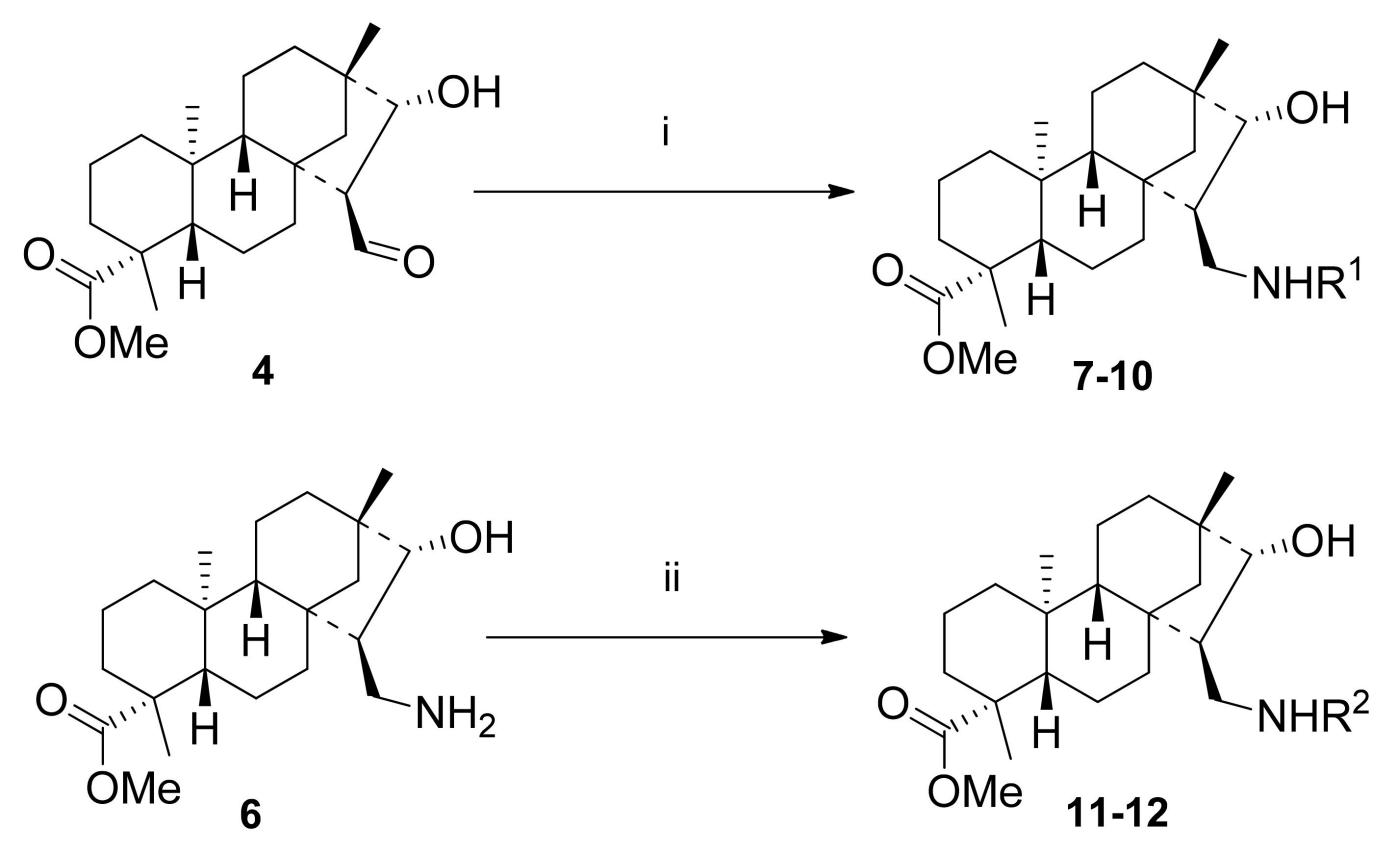
Synthesis of isosteviol-based 1,3-aminoalcohols. (i) 1) R^1^NH_2_ (1 eq.), dry EtOH, 3 h, 25 °C; 2) dry MeOH, NaBH_4_ (2 eq.), 3–4 h, 25 °C, 64–83%; (ii) 1) aldehydes (1 eq.), dry EtOH, 3 h, 25 °C; 2) dry MeOH, NaBH_4_ (2 eq.), 3–4 h, 25 °C, 64–65%.

The results of our previous study on *N*-substituted steviol-based aminodiols and related literature data on the antiproliferative activity of primary 1,3-aminoalcohols based on isosteviol predicted the interest of *N*-substituted 1,3-aminoalcohols. Therefore, a small library of 1,3-aminoalcohols was prepared to study structure–bioactivity relationship of *N*-substitution and antiproliferative activity [25,33]. Syntheses were accomplished via two pathways: reductive amination of hydroxyaldehyde **4** with primary amines or reductive alkylation of primary 1,3-aminoalcohol **6** with different aldehydes via formation of Schiff bases, followed by reduction with NaBH_4_ under mild conditions. The desired *N*-substituted 1,3-aminoalcohols (**7**–**12**) were isolated in acceptable yields. Reaction conditions and yields are presented in Scheme 2 and Table 1.

### 2.3. Syntheses and Reduction of Isosteviol-Based 1,3-Aminoketones Obtained via Mannich Condensation

**Scheme 3:**
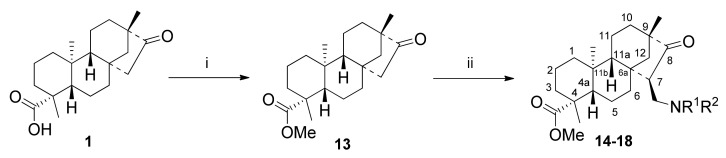
Synthesis of aminoketones **14**–**18** via Mannich condensation. (i) CH_2_N_2_, Et_2_O, 5 min, 25 °C, 79%; (ii) NHR^1^R^2^_*_HCl (1 eq.), (CH_2_O)_n_ (2 eq.), AcOH, 24 h, reflux, 13–68%.

Isosteviol methyl ester **13** was prepared from **1** with diazomethane in excellent yield [34]. The Mannich condensation of **13** was accomplished with paraformaldehyde and different secondary amine HCl salts in glacial acetic acid, resulting in a library of aminoketones with good to moderate yields (Scheme 3, Table 2) [28]. The condensation reaction took place in an exclusive stereoselective manner, forming a single diastereoisomer with (7*R*) configuration of the new stereocenter at C15. Results are collected in Table 2.

**Scheme 4:**
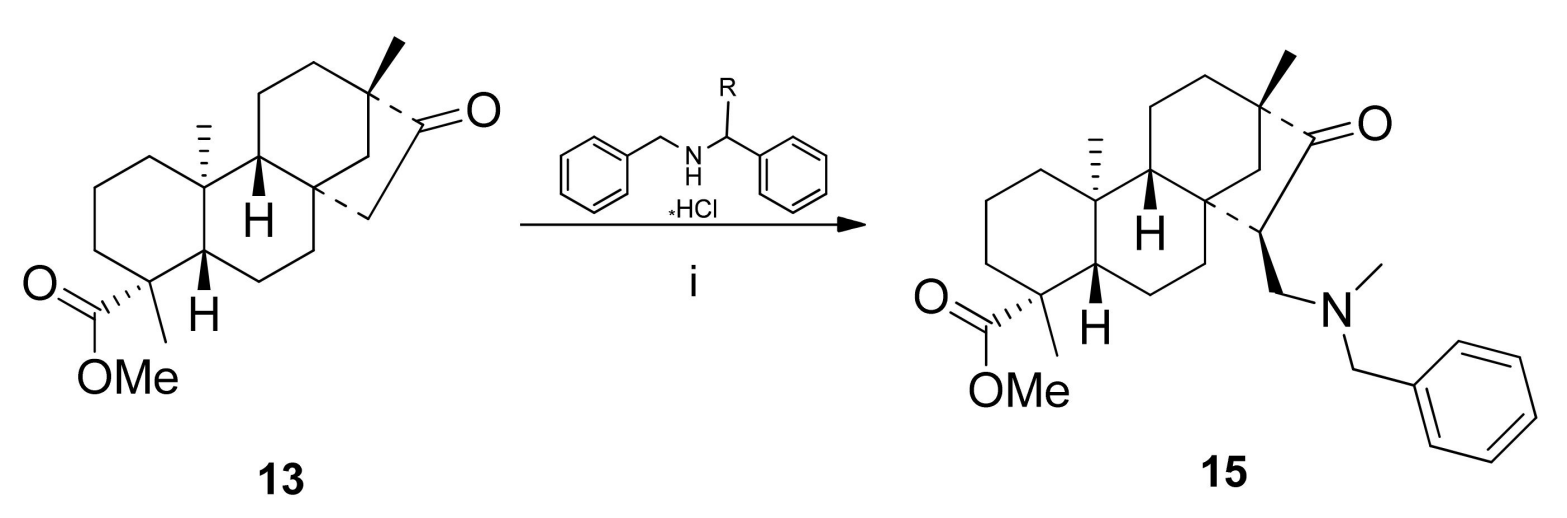
Substituent exchange under Mannich condensation. R = H, Me(*S*), Me(*R*). (i) (CH_2_O)_n_ (2 eq.), AcOH, 24 h, reflux, 13%.

As entry 6 of Table 2 shows, the condensation of **13** with dibenzylamine hydrochloride surprisingly led to *N*-methyl-*N*-benzyl derivative **15** instead of the expected *N*,*N*-dibenzyl-substituted product, although with a low yield (13%). When the reaction was repeated with both *N*-benzyl-*N*-(*S*)-α-methylbenzylamine and the corresponding (7*R)* enantiomer, **15** as a single product could be isolated again (Scheme 4).

**Scheme 5:**
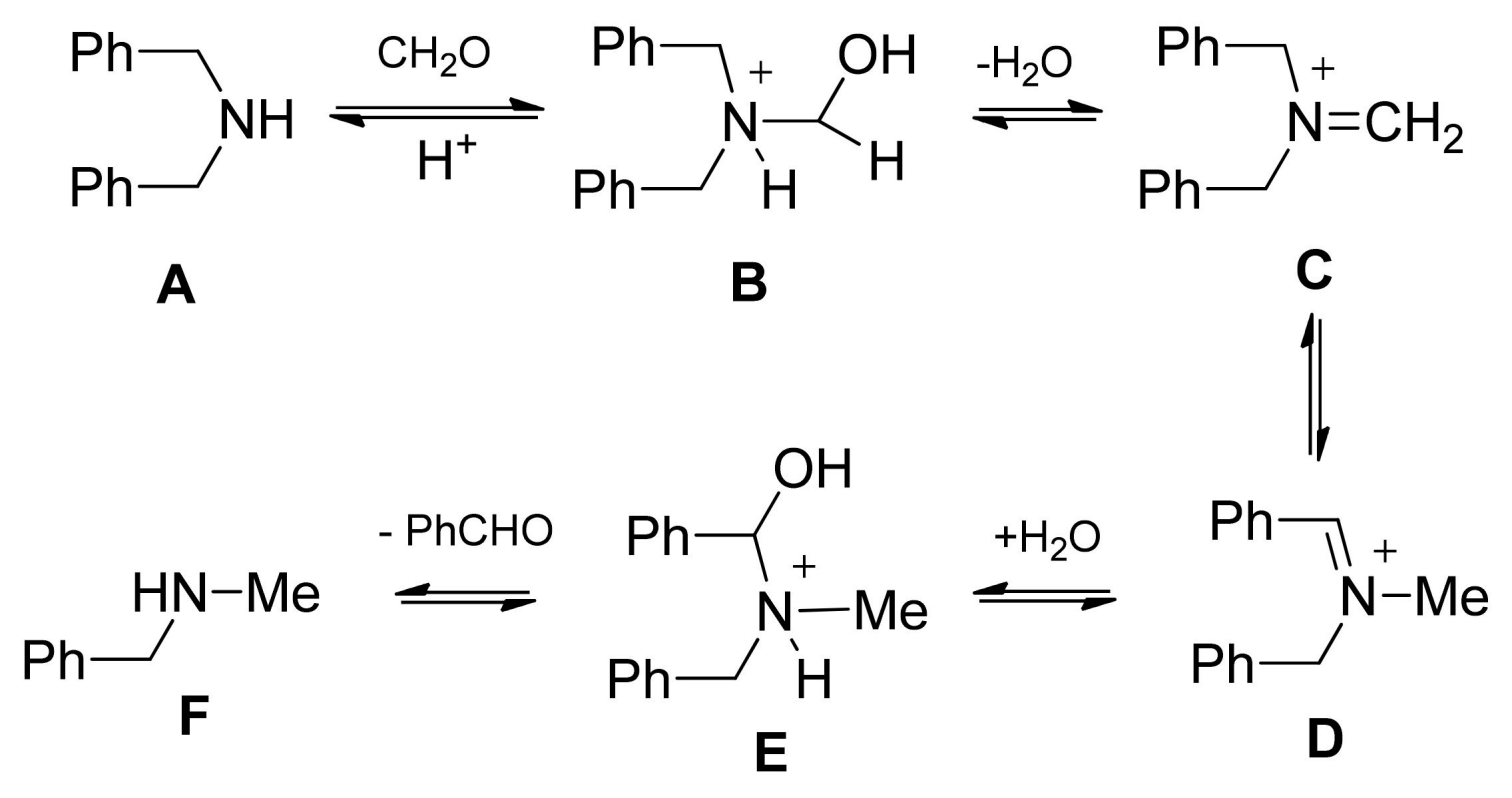
Proposed substituent exchange under Mannich condensation of dibenzylamine.

This interesting *N*-benzyl → *N*-methyl substituent exchange can be explained with the special steric hindrance of the diterpenoid skeleton with *N*-methyl-*N*-benzylamine representing the limit of the Mannich condensation in the case of this special ring system (Scheme 5). According to the classical mechanism of Mannich condensation, the first step is the formation of an iminium ion in the reaction of dibenzylamine and formaldehyde (Scheme 5). Because of steric hindrance, iminium species **C** cannot react with the enolate of the ketone. Rather, under the applied conditions, isomeric iminium salt **D** is formed. This step is followed by water addition and benzaldehyde elimination, resulting in *N*-methyl-*N*-benzylamine (**F**), ready for the condensation to give **15**.

**Scheme 6:**
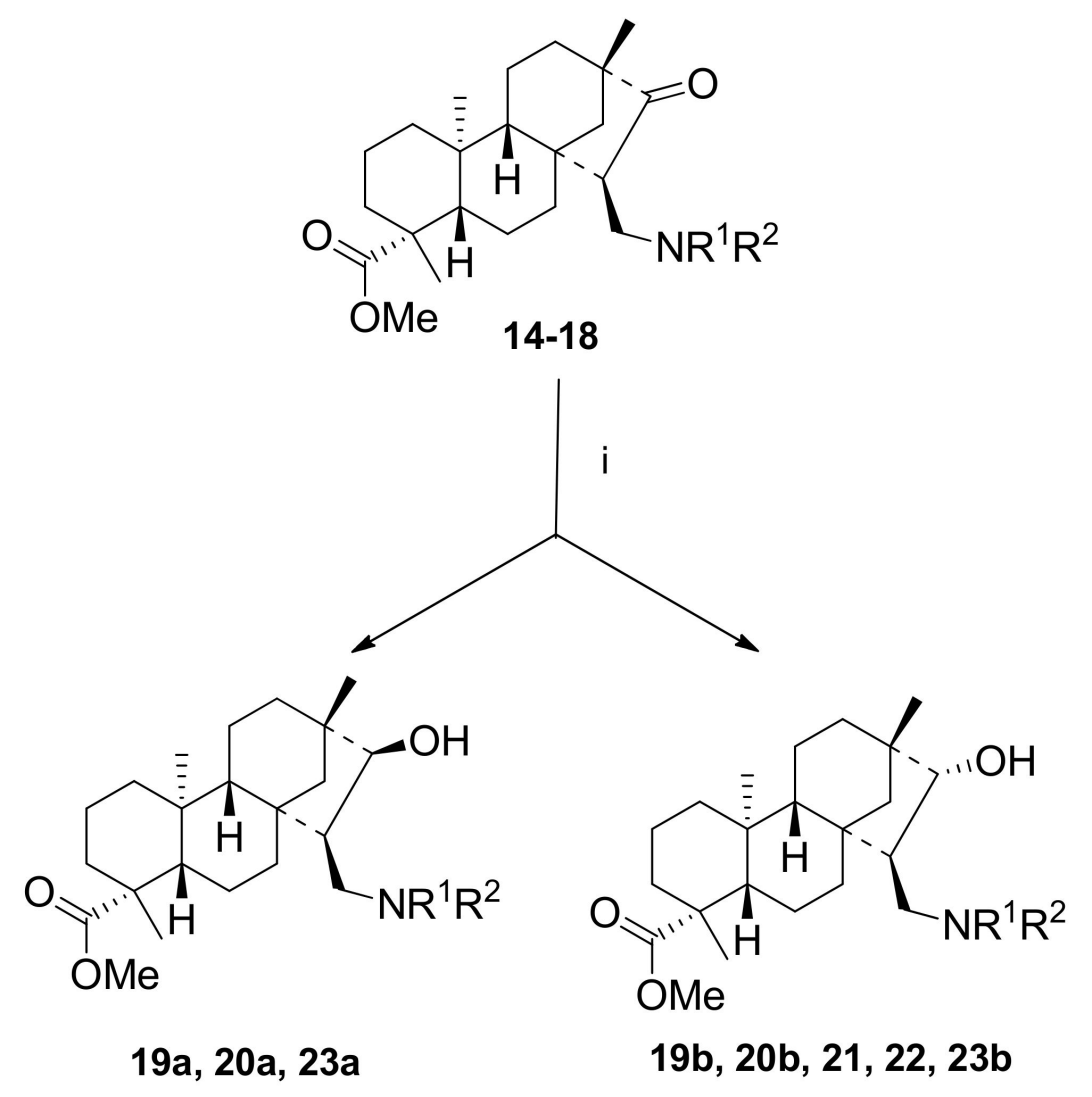
Synthesis of aminoalcohols **19a**–**23b** via reduction of **14**–**18** with NaBH_4_. (i) NaBH_4_ (2 eq.), dry MeOH, 2–3 h, 25 °C, 10–75%.

The reduction of aminoketones **14**–**18** with NaBH_4_ under mild conditions provided diastereomeric mixtures of 1,3-aminoalcohols. Reaction routes are outlined in Scheme 6. When pyrrolidinoaminoketone (**16**) or dimethylaminoketone (**17**) derivatives were applied, the reaction proceeded in a highly stereoselective way, resulting in the formation of **21** and **22** as single diastereoisomers. In other cases, diastereomeric mixtures were formed. Data are presented in Table 3.

The different steric hindrances of *N*-substituents can explain the different stereoselectivity of reduction of amino ketones. Probably in the case of less hindrance aminomethyl substituents (**15**-**17**), a cyclic complex can be formed with the protic solvent, and the hydride can attack from only the less sterically hindrance side, while this complex cannot be formed in the case of bulky *N*-substitution (**14** and **18**) and therefore the attack of the hydride can take place both side of the carbonyl function, resulting in a mixture of diastereoisomers [35].

**Figure 1:**
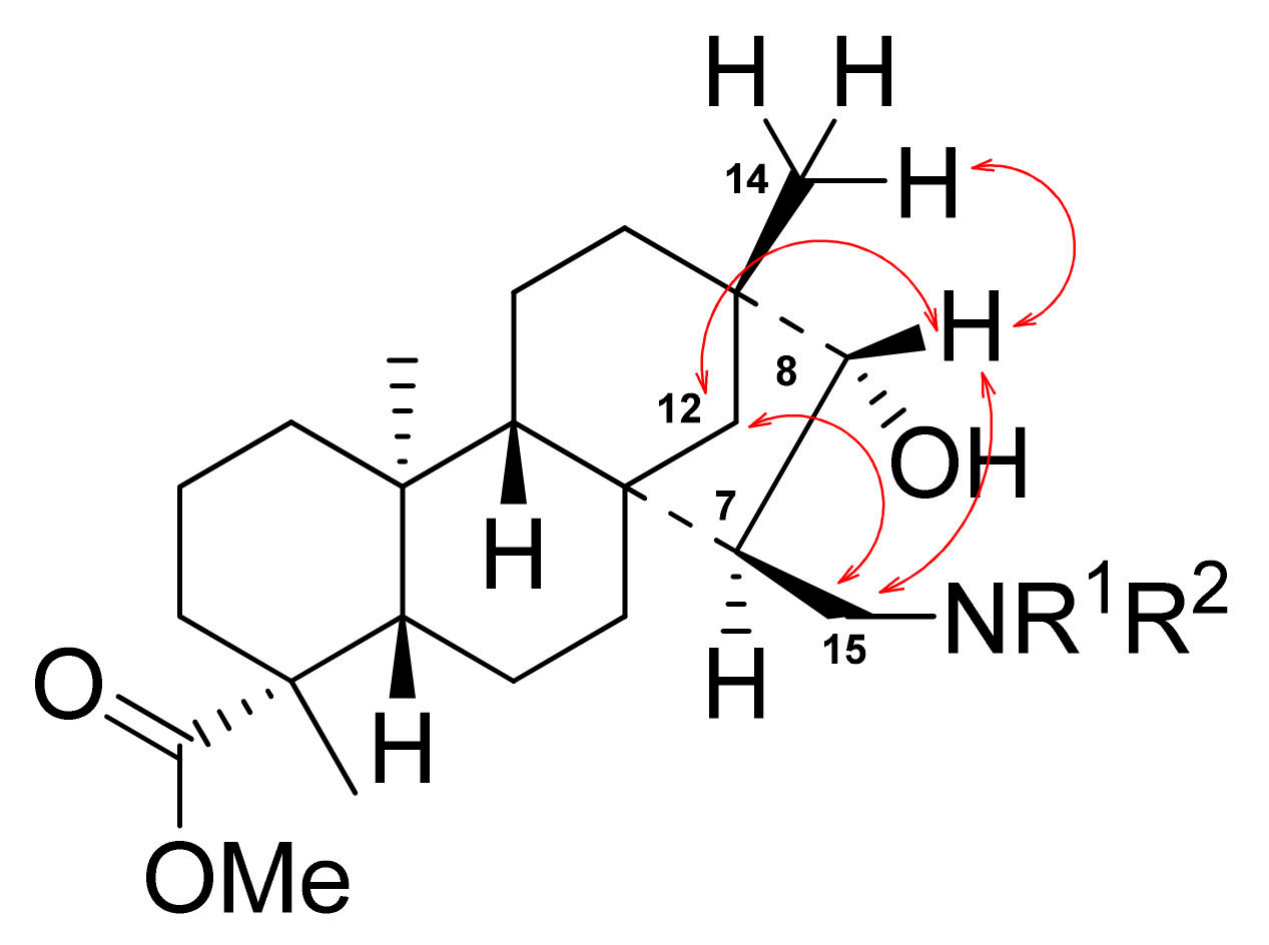
Determination of the structure of 1,3-aminoalcohols by NOESY.

The relative, therefore absolute configurations of the new stereocenters of aminoalcohols **19**-**23** at position 7 and 8 were determined by NMR with NOESY spectral analysis, based on the observation of NOE effects between H-C(12) and H-C(8), H-C(8) and H-C(15), H-C(12) and Me-C(17), as well as between H-C(12) and H-C(15). Thus, the structure of **19b**, **20b**, **21**, **22**, and **23b** was determined as outlined in Figure 1. Similarly, NOE effects were observed in the case of **19a**, **20a**, and **23a**.

**Scheme 7:**
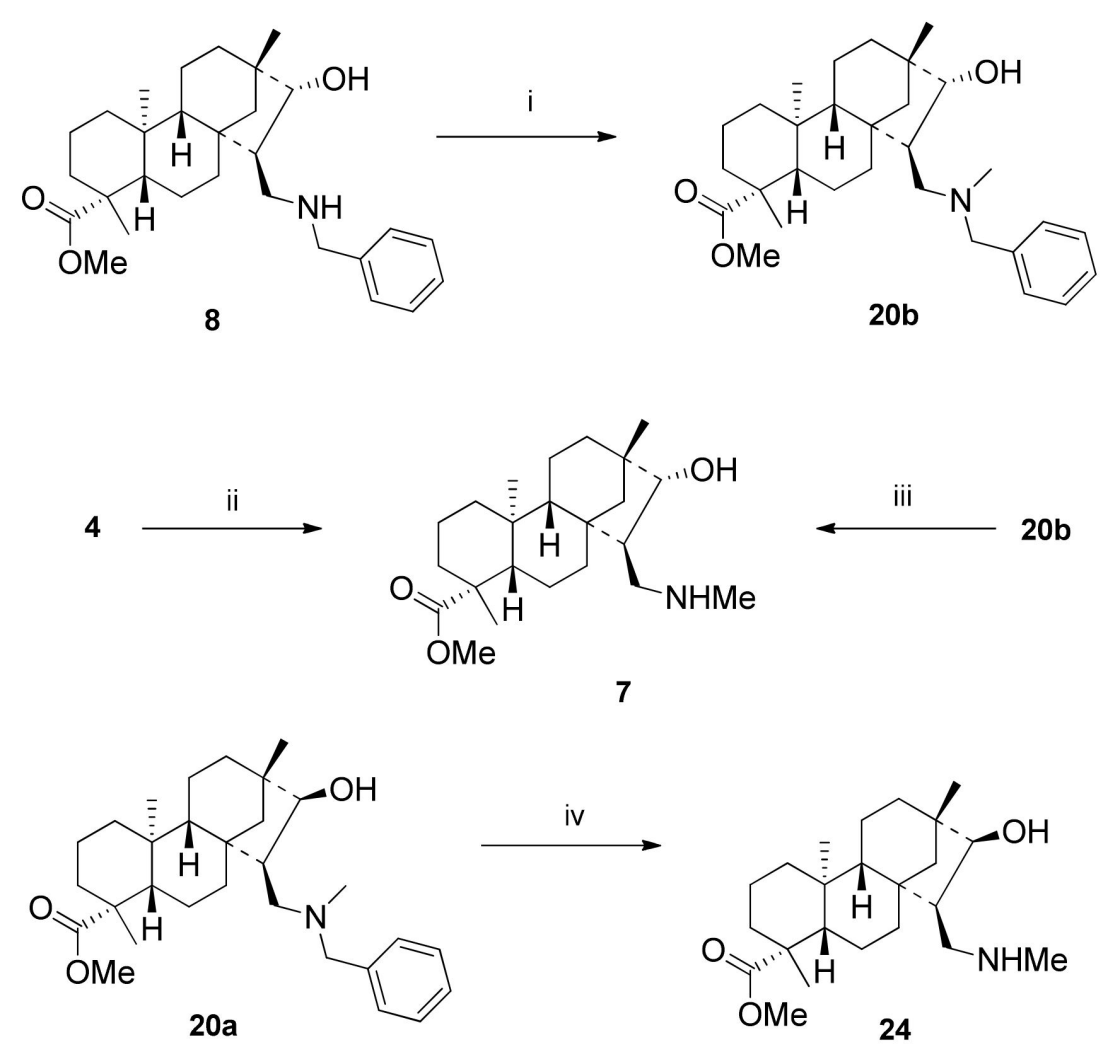
Alternative determination of the structure of 1,3-aminoalcohols via synthesis. (i) DCM, Et_3_N (1 eq.), MeI (1 eq.), 4 h, 25 °C, 77%; (ii) 1) MeNH_2_ (1 eq.), EtOH, 3 h, 25 °C; 2) MeOH, NaBH_4_ (2 eq.), 3–4 h, 25 °C, 77%; (iii) MeOH, 5% Pd/C, H_2_ (1 atm), 12 h, 25 °C, 24%; (iv) MeOH, 5% Pd/C, H_2_ (1 atm), 12 h, 25 °C, 28%.

Beside the NOESY experiments, the configurations of the newly formed stereocenters of 1,3-aminoalcohols were determined via two alternative synthetic pathways (Scheme 7). Reductive amination of **4** (obtained from **3** with known stereochemistry) with benzylamine followed by methylation of **8** with iodomethane yielded a product that was identical with **20b** obtained as a major product of the reduction of aminoketone **15**. Alternatively, debenzylation of **20b** over 5% Pd/C catalyst in methanol resulted in *N*-methyl aminoalcohol identical with **7** obtained by reductive amination of **4** with methylamine. Diastereoisomer **24** was also prepared by debenzylation of **20a** over 5% Pd/C catalyst (Scheme 7).

### 2.4. Attempted Ring Closure of Isosteviol-Based Thiourea Derivatives

**Scheme 8:**
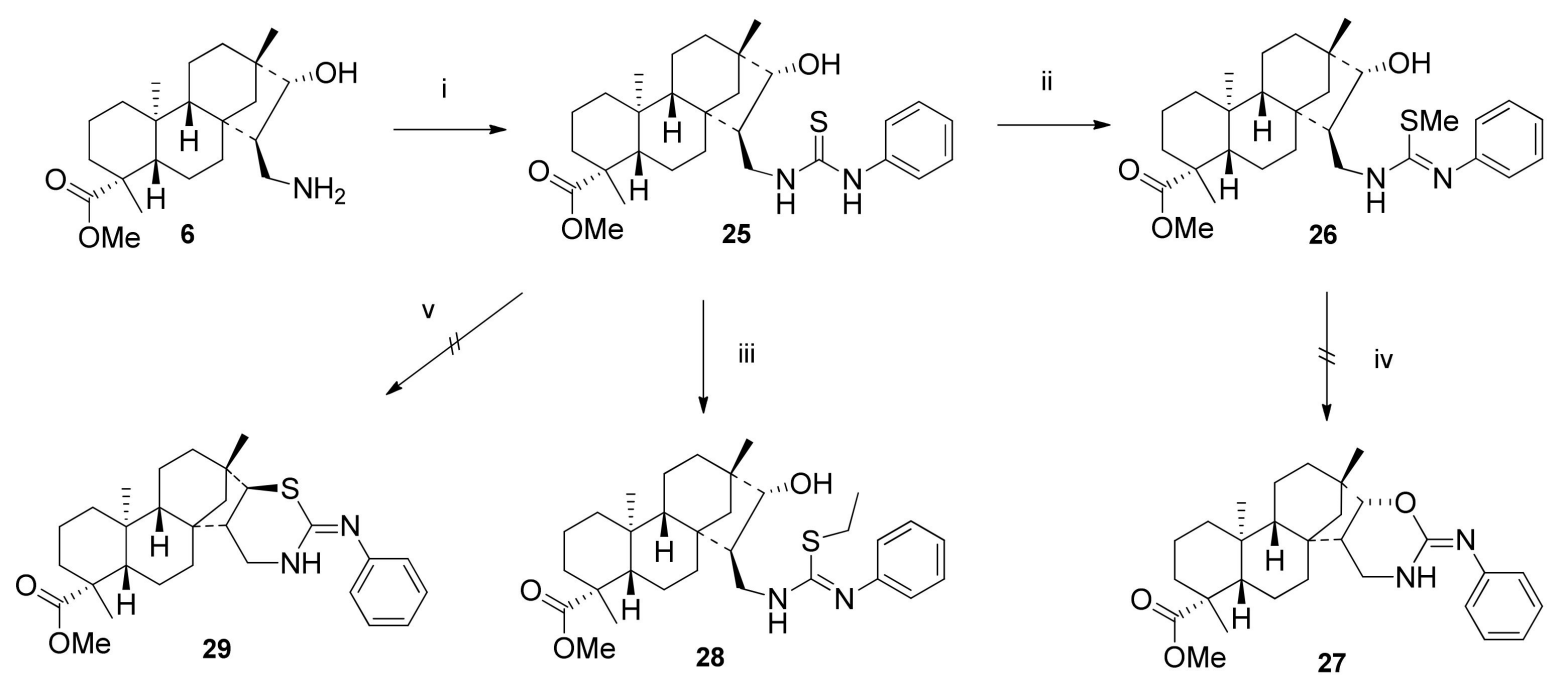
Attempted synthesis of 2-phenylimino-1,3-oxazine and -1,3-thiazine. (i) PhNCS (1 eq.), DCM, 2 h, 25 °C, 91%; (ii) MeI (5 eq.), EtOH, 2 h, 25 °C, 69%; (iii) 18% HCl, dry EtOH, 12 h, reflux, 46%; (iv) 10% KOH, EtOH, 4 h, reflux; (v) 18% HCl, EtOH, 12 h, reflux.

In our earlier studies we observed expressed cytotoxic activity of monoterpene-fused 2-phenylimino-1,3-oxazines and -1,3-thiazines on human cancer cell lines [36,37]. Consequently, we decided to convert primary 1,3-aminoalcohol **6** into its 1,3-oxazine and 1,3-thiazine derivatives. Reaction of **6** with phenyl isothiocyanate in DCM at room temperature provided the corresponding thiourea **25** with excellent yield (Scheme 8) [37]. The ring closure of thiourea **25** was attempted in a two-step procedure, which involved the treatment of **25** with methyl iodide, followed by alkaline-induced elimination of methyl mercaptol. Unfortunately, only the formation of thiomethyl ether intermediate **26** was observed. Alternatively, acid-promoted dehydrative cyclisation of **25** by treatment with EtOH containing 18% hydrochloric acid gave surprisingly only thioethyl ether **28** instead of the expected 2-phenylimino-1,3-thiazine **29** in moderate yield [38]. This reaction can be explained by the steric hindrance of the diterpene skeleton which inhibits the attack of sulphur on H-C(16) carbon, meanwhile the reaction of HCl with EtOH under the applied conditions can generate EtCl in situ, which reacts with thiourea **25** similarly to MeI, resulting in thioether **28** (Scheme 8).

### 2.5. Antiproliferative Properties of the Prepared Diterpenes

The antiproliferative activities of the prepared diterpene analogues were determined by means of MTT assay on a panel of human adherent cancer lines, including cells from cervical (HeLa, SiHa), breast (MDA-MB-231, MCF-7), and ovary cancers (A2780) as given in Table 4. Based on the obtained activities, some conclusions could be arrived at with respect to structure–activity relationships. Since the original diol (**3**), its aldehyde analogue (**4**), and the corresponding oxime (**5**) elicited no relevant effect on the growth of cancer cells, an amino function seems to be essential for antiproliferative activity (Table 4). Primary amine **6** as well as secondary amine derivatives **7**–**12** exerted similarly pronounced antiproliferative action, and the calculated IC_50_ values of these compounds are comparable to or lower than those of reference agent cisplatin. The cell line-independent IC_50_ values may be interpreted as a marker of general cytotoxic property of molecules 6–12. From the results presented in Table 4, it seems to be clear that both the aminoalcohol function and the *N*-benzyl substitution (**8–12**), but not the aliphatic substitution (**7** and **24**), are essential for the remarkable antiproliferative activity. Cervical cell lines are especially sensitive to these agents. Aminoketones **14**–**18** are much less effective and most of them exert only negligible activity. Reduced analogues (**19**–**23**/**24**), bearing tertiary amino function, proved to elicit more pronounced action compared only with aminoketones, and the orientation of the newly formed alcohol function has no substantial impact on the efficacy of the product. Phenylthioureido analogue **25** exerted some modest activities with IC_50_ values between 10 and 23 μM, while the thioether type compounds **26** and **28** do not favour the antiproliferative action of the diterpene skeleton.

## 3. Conclusions

In summary, a series of novel isosteviol derivatives containing 1,3-aminoalcohol and thiourea moieties have been synthesised with moderated to good yields, and their cytotoxic activities against five human cancer cell lines (HeLa, Siha, MCF7, MDA-MB-231, A2780) have been investigated. Starting from commercially available stevioside, a new family of isosteviol-based chiral 1,3-aminoalcohols and thioureid derivatives were prepared through hydroxyaldehyde and isosteviol methyl ester as key intermediates via stereoselective transformations. The resulting 1,3-aminoalcohols exert remarkable antiproliferative action of human cancer cell lines. The in vitro pharmacological studies have clearly shown that the *N*-benzyl substituent at the amino function is essential and some of the prepared molecules proved to be more potent than anticancer agent cisplatin used clinically.

## 4. Materials and Methods

**General methods**: Commercially available reagents were used as obtained from suppliers (Molar Chemicals Ltd., Halásztelek, Hungary; Merck Ltd., Budapest, Hungary and VWR International Ltd., Debrecen, Hungary), while solvents were dried according to standard procedures. Optical rotations were measured in MeOH at 20 °C with a Perkin-Elmer 341 polarimeter (PerkinElmer Inc., Shelton, CT, USA). Chromatographic separations and monitoring of reactions were carried out on Merck Kieselgel 60 (Merck Ltd., Budapest, Hungary). Melting points were determined on a Kofler apparatus (Nagema, Dresden, Germany). ^1^H- and ^13^C-NMR spectra were recorded on Brucker Avance DRX 500 spectrometer (Bruker Biospin, Karlsruhe, Baden Württemberg, Germany) [500 MHz (^1^H) and 125 MHz (^13^C), δ = 0 (TMS)]. Chemical shifts are expressed in ppm (δ) relative to TMS as internal reference. *J* values are given by Hz. All ^1^H/^13^C NMR, NOESY, 2D-HMBC, and 2D-HMQC spectra are available in Supporting Information file. HRMS flow injection analysis was performed with a Thermo Scientific Q Exactive Plus hybrid quadrupole-Orbitrap (Thermo Fisher Scientific, Waltham, MA, USA) mass spectrometer coupled to a Waters Acquity I-Class UPLC™ (Waters, Manchester, UK).

**Starting materials**: Stevioside was obtained from Molar Chemicals Ltd., Halásztelek, Hungary. Isosteviol **1** was prepared from commercially available stevioside or a mixture of steviol glycosides in a one-step synthesis according to the literature method, and all its spectroscopic data were the same as described in the literature [30].

Compounds **2**, **3**, and **13** were prepared by literature methods. Their spectroscopic data and physical and chemical properties were similar to those reported therein [31,34]. ^1^H, ^13^C, COSY, HSQC, HMBC, and NOESY NMR spectra of new compounds are available in Supplementary Materials.

**(4*R*,6a*S*,7*R*,8*R*,9*S*,11b*S*)-Methyl 7-formyl-8-hydroxy-4,9,11b-trimethyltetradecahydro-6a,9-methanocyclohepta[a]naphthalene-4-carboxylate (4):** To a solution of 3 (4.70 mmol, 1.73 g) in DCM/H_2_O (50 / 50 mL), TEMPO (10 mol%, 73 mg), NCS (9.40 mmol, 1.26 g), and TBAB (4.70 mmol, 1.52 g) was added. After 12 h reflux the reaction was found to be completed (indicated by TLC), and the mixture was extracted with DCM (3 × 50 mL). The combined organic phase was extracted with water (1 × 50 mL), dried (Na_2_SO_4_), filtered, and concentrated. The purification of the crude product was accomplished by column chromatography on silica gel with an appropriate solvent mixture (*n*-hexane/EtOAc = 4:1). Yield: 1.53 g (90%); white crystals; m.p. 151–152 °C; ${\left[\alpha \right]}_{D}^{20}$ = –117 (*c* 0.24 MeOH); ^1^H-NMR (500 MHz, CDCl_3_) δ (ppm): 0.86 (s, 3H), 0.89–0.93 (m, 1H), 0.97 (s, 3H), 1.01–1.06 (m, 3H), 1.12–1.14 (m, 1H), 1.17 (s, 3H), 1.20–1.27 (m, 2H), 1.37–1.40 (m, 1H), 1.42–1.45 (m, 1H), 1.53–1.85 (m, 8H), 2.18 (d, 2H, *J* = 13.3 Hz), 2.92 (s, 1H), 3.64 (s, 3H), 4.26 (d, 1H, *J* = 4.9 Hz), 9.93 (d, 1H, *J* = 2.5 Hz); ^13^C-NMR (125 MHz, CDCl_3_) δ (ppm) 13.0 (CH_3_), 18.8 (CH_2_), 19.7 (CH_2_), 21.6 (CH_2_), 24.5 (CH_3_), 28.8 (CH_3_), 33.0 (CH_2_), 35.9 (CH_2_), 37.9 (C_q_), 38.2 (C_q_), 39.6 (CH_2_), 41.2 (C_q_), 43.7 (C_q_), 46.5 (C_q_), 51.2 (CH_3_), 53.9 (CH_2_), 56.8 (CH), 57.4 (CH), 61.7 (CH), 78.3 (CH), 177.8 (C=O), 204.4 (CH). C_22_H_34_O_4_ (362.50): 363.26. HRMS (ESI+): *m*/*z* calcd. for C_22_H_35_O_4_ [M + H]^+^ 363.2535; found 363.25230.

**(4*R*,6a*S*,7*R*,8*R*,9*S*,11b*S*)-Methyl 8-hydroxy-7-((hydroxyimino)methyl)-4,9,11b-trimethyltetradecahydro-6a,9-methanocyclohepta[a]naphthalene-4-carboxylate (5)**: A mixture of compound 4 (4.20 mmol, 1.53 g) and hydroxylamine hydrochloride (8.40 mmol, 0.58 g) in 50 mL EtOH was stirred in presence of NaHCO_3_ (4.20 mmol, 0.35 g) at 60 °C for 2 h, then the reaction mixture was concentrated under vacuum and extracted with 50 mL DCM and 50 mL water. The water phase was extracted further with DCM (3 × 50 mL) and the combined organic phase was washed with saturated NaCl aqueous solution (1 × 50 mL), dried (Na_2_SO_4_), and concentrated under vacuum. The product obtained was purified by column chromatography (*n*-hexane/EtOAc = 2:1). Yield: 1.21 g (76%); white crystals; m.p. 113–114 °C; ${\left[\alpha \right]}_{D}^{20}$ = –90 (*c* 0.40 MeOH); ^1^H-NMR (500 MHz, CDCl_3_) δ (ppm): 0.75 (s, 3H), 0.84–0.90 (m, 1H), 0.94 (s, 3H), 0.97–1.06 (m, 4H), 1.11–1.17 (m, 4H), 1.19–1.26 (m, 1H), 1.40 (dd, 2H, *J* = 2.6 Hz, 12.0 Hz), 1.56–1.72 (m, 6H), 1.76–1.85 (m, 2H), 2.16 (d, 1H, *J* = 13.3 Hz), 2.71–2.73 (m, 1H), 3.17 (s, 1H), 3.63 (s, 3H), 3.76 (d, 1H, *J* = 4.8 Hz), 7.47 (d, 1H, *J* = 8.5 Hz), 8.90 (s, 1H,); ^13^C-NMR (125 MHz, CDCl_3_) δ (ppm): 13.1 (CH_3_), 18.8 (CH_2_), 19.5 (CH_2_), 20.8 (CH_2_), 24.9 (CH_3_), 28.8 (CH_3_), 33.0 (CH_2_), 35.8 (CH_2_), 38.0 (CH_2_), 38.2 (C_q_), 39.6 (CH_2_), 41.4 (C_q_), 43.7 (C_q_), 44.8 (C_q_), 49.9 (CH), 51.3 (CH_3_), 53.9 (CH_2_), 56.9 (CH), 57.3 (CH), 83.7 (CH), 153.9 (CH), 178.1 (C=O). HRMS (ESI+): *m*/*z* calcd. for C_22_H_36_NO_4_ [M + H]^+^ 378.2644; found 378.2639.

**(4*R*,6a*S*,7*R*,8*R*,9*S*,11b*S*)-Methyl 7-aminomethyl-8-hydroxy-4,9,11b-trimethyltetradecahydro-6a,9-methanocyclohepta[a]naphthalene-4-carboxylate (6):** To a suspension of Raney nickel (0.20 g) in THF (20 mL) solution of oxime 5 (1.53 g, 4.10 mmol) in THF (30 mL) was added and the mixture was stirred under H_2_ atmosphere (10 atm) at room temperature for 12 h. The mixture was then filtered and evaporated, and the crude product was purified by crystallisation (*n*-hexane/DCM). Compound 6: 1.24 g (83%); white crystals; m.p. 165–166 °C; ${\left[\alpha \right]}_{D}^{20}$ = −42 (*c* 0.33 MeOH); ^1^H-NMR (500 MHz, CDCl_3_) δ (ppm): 0.73 (s, 3H), 0.84–0.90 (m, 1H), 0.92 (s, 3H), 0.94–1.07 (m, 5H), 1.16–1.20 (m, 4H), 1.34–1.43 (m, 2H), 1.60–1.85 (m, 9H), 2.15–2.17 (m, 3H), 2.44 (t, 1H, *J* = 11.8 Hz), 3.13 (dd, 1H, *J* = 3.4 Hz, 11.3 Hz), 3.50 (d, 1H, *J* = 4.4 Hz), 3.63 (s, 3H); ^13^C-NMR (125 MHz, CDCl_3_) δ (ppm): 13.1 (CH_3_), 19.0 (CH_2_), 19.5 (CH_2_), 22.1 (CH_2_), 25.1 (CH_3_), 28.9 (CH_3_), 33.2 (CH_2_), 35.0 (CH_2_), 38.0 (CH_2_), 38.2 (C_q_), 39.7 (CH_2_), 40.9 (C_q_), 42.6 (C_q_), 43.8 (C_q_), 44.1 (CH_2_), 50.7 (CH), 51.2 (CH_3_), 54.2 (CH_2_), 57.2 (CH), 57.8 (CH), 87.7 (CH), 177.9 (C=O). HRMS (ESI+): *m*/*z* calcd. for C_22_H_38_NO_3_ [M + H]^+^ 364.2852; found 364.2846.

**General procedure for preparation of aminoalcohol with primary amines and aldehydes:** Method A: To a solution of **4** (0.10 g, 0.28 mmol) in dry EtOH (10 mL), primary amines (0.28 mmol) were added in one portion and the solution was stirred at room temperature for 3 h and then evaporated to dryness. The residue was dissolved in dry EtOH (10 mL), stirred for a further 1 h, and evaporated to dryness again. The product was dissolved in dry MeOH (10 mL) and NaBH_4_ (0.56 mmol, 0.02 g) was added in small portions to the mixture under ice cooling. After stirring for 4 h at room temperature, the mixture was evaporated to dryness, and the residue was dissolved in H_2_O (20 mL) and extracted with DCM (3 × 20 mL). The combined organic layer was dried (Na_2_SO_4_), filtered and evaporated to dryness. The crude product obtained was purified by column chromatography on silica gel (CHCl_3_/MeOH = 19:1).

Method B: To a solution of **6** (0.10 g, 0.28 mmol) in dry EtOH (10 mL), aldehydes (0.28 mmol) were added in one portion, and the solution was stirred at room temperature for 3 h and then evaporated to dryness. The product was dissolved in dry EtOH (10 mL) and stirred for a further 1 h and evaporated to dryness again. The crude product was dissolved in dry MeOH (10 mL) and NaBH_4_ (0.56 mmol, 0.02 g) was added in small portions to the mixture under ice cooling. After stirring for 4 h at room temperature, the mixture was evaporated to dryness, and the residue was dissolved in H_2_O (20 mL) and extracted with DCM (3 × 20 mL). The combined organic layer was dried (Na_2_SO_4_), filtered and evaporated to dryness. The crude product obtained was purified by column chromatography on silica gel (CHCl_3_/MeOH = 19:1).

**(4*R*,6a*S*,7*R*,8*R*,9*S*,11b*S*)-Methyl 8-hydroxy-4,9,11b-trimethyl-7-((methylamino)methyl)tetradecahydro-6a,9-methanocyclohepta[a]naphthalene-4-carboxylate (7)**: The reaction was accomplished starting from compound 4 with 33 wt% methylamine (0.28 mmol, 0.02 mL) according to the general procedure Method A. Yield: 0.09 g (80%). An alternative synthesis of 7 was accomplished from 26 with a yield of 0.03 g (24%). The product (0.15 g, 0.33 mmol) in MeOH (25 mL) was added to a suspension of palladium-on-carbon (5% Pd/C, 0.10 g), and the mixture was stirred under a H_2_ atmosphere (1 atm) at room temperature. After completion of the reaction (monitored by TLC, 24 h), the mixture was filtered through a Celite pad, and the solution was evaporated to dryness. The crude product was purified by column chromatography on silica gel (CHCl_3_/MeOH = 9:1). Compound 7: white crystals; m.p. 135–136 °C; ${\left[\alpha \right]}_{D}^{20}$ = –66 (*c* 0.37 MeOH); ^1^H-NMR (500 MHz, CDCl_3_) δ (ppm): 0.73 (s, 3H), 0.85–0.88 (m, 1H), 0.92 (s, 3H), 0.94–1.07 (m, 5H), 1.16–1.21 (m, 4H), 1.36–1.43 (m, 2H), 1.59–1.82 (m, 8H), 1.87–1.89 (m, 1H), 2.01 (s, 2H), 2.16 (d, 1H, *J* = 13.3 Hz), 2.33 (t, 1H, *J* = 11.3 Hz), 2.48 (s, 3H), 2.92 (d, 1H, *J* = 11.0 Hz), 3.46–3.47 (m, 1H), 3.63 (s, 3H); ^13^C-NMR (125 MHz, CDCl_3_) δ (ppm): 13.1 (CH_3_), 19.0 (CH_2_), 19.6 (CH_2_), 22.1 (CH_2_), 25.0 (CH_3_), 28.9 (CH_3_), 33.1 (CH_2_), 35.1 (CH_2_), 36.8 (CH_3_), 38.0 (CH_2_), 38.1 (C_q_), 39.6 (CH_2_), 40.8 (C_q_), 42.4 (C_q_), 43.8 (C_q_), 47.9 (CH), 51.1 (CH_3_), 54.3 (CH_2_), 54.7 (CH_2_), 57.2 (CH), 57.9 (CH), 88.3 (CH), 177.8 (C=O). HRMS (ESI+): *m*/*z* calcd. for C_23_H_40_NO_3_ [M + H]^+^ 378.3008; found 378.3003.

**(4*R*,6a*S*,7*R*,8*R*,9*S*,11b*S*)-Methyl 7-((benzylamino)methyl)-8-hydroxy-4,9,11b-trimethyltetradecahydro-6a,9-methanocyclohepta[a]naphthalene-4-carboxylate (8)**: The reaction was accomplished starting from compound 4 with benzylamine (0.28 mmol, 0.02 mL) according to the general procedure Method A. Yield: 0.11 g (83%); white crystals; m.p. 114–115 °C; ${\left[\alpha \right]}_{D}^{20}$ = –75 (*c* 0.33 MeOH); ^1^H-NMR (500 MHz, CDCl_3_) δ (ppm): 0.70 (s, 3H), 0.83–0.90 (m, 2H), 0.92 (s, 3H), 0.94–1.06 (m, 4H), 1.15 (s, 3H), 1.17–1.21 (m, 1H), 1.34 (dd, 1H, *J* = 2.5 Hz, 11.3 Hz), 1.39–1.42 (m, 1H), 1.58–1.62 (m, 4H), 1.70–1.81 (m, 4H), 1.84–1.88 (m, 1H), 2.15 (d, 1H, *J* = 13.3 Hz), 2.35 (t, 1H, *J* = 11.9 Hz), 3.02 (dd, 1H, *J* = 4.0 Hz, 11.0 Hz), 3.47 (d, 1H, *J* = 4.9 Hz), 3.60 (s, 3H), 3.76 (d, 1H, *J* = 13.4 Hz), 3.88 (d, 1H, *J* = 13.4 Hz), 7.22–7.26 (m, 1H), 7.30–7.34 (m, 4H); ^13^C-NMR (125 MHz, CDCl_3_) δ (ppm): 13.1 (CH_3_), 19.0 (CH_2_), 19.6 (CH_2_), 22.1 (CH_2_), 25.1 (CH_3_), 29.0 (CH_3_), 33.0 (CH_2_), 35.0 (CH_2_), 38.0 (CH_2_), 38.1 (C_q_), 39.6 (CH_2_), 40.6 (C_q_), 42.3 (C_q_), 43.7 (C_q_), 48.5 (CH), 51.2 (CH_3_), 51.8 (CH_2_), 54.2 (CH_2_), 54.3 (CH_2_), 57.1 (CH), 57.8 (CH), 88.7 (CH), 126.9 (CH), 128.0 (2xCH), 128.4 (2xCH), 140.6 (C_q_), 177.9 (C=O). HRMS (ESI+): *m*/*z* calcd. for C_29_H_44_NO_3_ [M + H]^+^ 454.3321; found 454.3223.

**(4*R*,6a*S*,7*R*,8*R*,9*S*,11b*S*)-Methyl 8-hydroxy-4,9,11b-trimethyl-7-((((*S*)-1-phenylethyl)amino)methyl)tetradecahydro-6a,9-methanocyclohepta[a]naphthalene-4-carboxylate (9)**: The reaction was accomplished starting from compound 4 with (*S*)-(–)-α-methylbenzylamine (0.28 mmol, 0.04 mL) according to the general procedure Method A. Yield: 0.09 g (70%); white crystals; m.p. 108–110 °C; ${\left[\alpha \right]}_{D}^{20}$ = +63 (*c* 0.29 MeOH); ^1^H-NMR (500 MHz, CDCl_3_) δ (ppm): 0.59 (s, 3H), 0.78–0.84 (m, 1H), 0.89 (s, 3H), 0.92–1.03 (m, 5H), 1.09 (s, 3H), 1.11–1.23 (m, 2H), 1.36–1.41 (m, 2H), 1.56–1.69 (m, 4H), 1.75–1.83 (m, 2H), 1.94 (d, 3H, *J* = 6.7 Hz), 2.12 (d, 1H, *J* = 13.1 Hz), 2.34 (d, 1H, *J* = 13.0 Hz), 2.81 (t, 1H, *J* = 13.0 Hz), 3.00 (dd, 1H, *J* = 3.6 Hz, 12.5 Hz), 3.59 (s, 3H), 3.75 (d, 1H, *J* = 4.6 Hz), 4.54–4.58 (m, 1H), 7.36–7.39 (m, 1H), 7.44 (t, 2H, *J* = 7.3 Hz), 7.66 (d, 2H, *J* = 7.4 Hz); ^13^C-NMR (125 MHz, CDCl_3_) δ (ppm): 13.4 (CH_3_), 18.8 (CH_2_), 19.4 (CH_2_), 21.8 (CH_2_), 21.9 (CH_3_), 24.6 (CH_3_), 28.8 (CH_3_), 33.1 (CH_2_), 34.7 (CH_2_), 37.9 (CH_2_), 38.0 (C_q_), 39.6 (CH_2_), 41.4 (C_q_), 43.0 (CH), 43.0 (C_q_), 43.5 (C_q_), 48.5 (CH_2_), 51.4 (CH_3_), 53.5 (CH_2_), 57.1 (CH), 58.1 (CH), 58.5 (CH), 84.3 (CH), 127.7 (2xCH), 129.2 (CH), 129.5 (2xCH), 136.4 (C_q_), 177.5 (C=O). HRMS (ESI+): *m*/*z* calcd. for C_30_H_46_NO_3_ [M + H]^+^ 468.3478; found 468.3472.

**(4*R*,6a*S*,7*R*,8*R*,9*S*,11b*S*)-Methyl 8-hydroxy-4,9,11b-trimethyl-7-((((*R*)-1-phenylethyl)amino)methyl)tetradecahydro-6a,9-methanocyclohepta[a]naphthalene-4-carboxylate (10)**: The reaction was accomplished starting from compound 4 with (*R*)-(–)-α-methylbenzylamine (0.28 mmol, 0.04 mL) according to the general procedure Method A. Yield: 0.08 g (64%); white crystals; m.p. 104–105 °C; ${\left[\alpha \right]}_{D}^{20}$ = –18 (*c* 1.01 MeOH); ^1^H-NMR (500 MHz, CDCl_3_) δ (ppm): 0.61 (s, 3H), 0.81–0.91 (m, 3H), 0.93 (s, 3H), 0.96–1.02 (m, 3H), 1.11 (s, 3H), 1.17–1.21 (m, 1H), 1.36 (d, 3H, *J* = 6.6 Hz), 1.38–1.42 (m, 1H), 1.44–1.47 (m, 1H), 1.55–1.60 (m, 3H), 1.64–1.70 (m, 3H), 1.76–1.80 (m, 3H), 2.13 (d, 1H, *J* = 13.3 Hz), 2.34 (t, 1H, *J* = 11.2 Hz), 2.84 (dd, 1H, *J* = 4.2 Hz, 11.1 Hz), 3.48 (d, 1H, *J* = 5.0 Hz), 3.55 (s, 3H), 3.77 (dd, 1H, *J* = 6.6 Hz, 6.6 Hz), 7.20–7.24 (m, 1H), 7.29–7.32 (m, 4H); ^13^C-NMR (125 MHz, CDCl_3_) δ (ppm): 12.8 (CH_3_), 18.9 (CH_2_), 19.5 (CH_2_), 22.1 (CH_2_), 24.1 (CH_3_), 25.1 (CH_3_), 28.8 (CH_3_), 33.0 (CH_2_), 35.0 (CH_2_), 38.1 (CH_2_) 38.1 (C_q_), 39.6 (CH_2_), 40.6 (C_q_), 42.3 (C_q_), 43.7 (C_q_), 48.9 (CH), 50.2 (CH_2_), 51.0 (CH), 54.4 (CH_2_), 57.1 (CH), 57.9 (CH), 58.8 (CH), 88.7 (CH), 126.4 (2xCH), 126.9 (CH), 128.4 (2xCH), 146.2 (C_q_), 177.9 (C=O). HRMS (ESI+): *m*/*z* calcd. for C_30_H_46_NO_3_ [M + H]^+^ 468.3478; found 468.3472.

**(4*R*,6a*S*,7*R*,8*R*,9*S*,11b*S*)-Methyl 8-hydroxy-7-(((4-methoxybenzyl)amino)methyl)-4,9,11b-trimethyltetradecahydro-6a,9-methanocyclohepta[a]naphthalene-4-carboxylate (11)**: The reaction was accomplished starting from compound 6 with 4-anisaldehyde (0.28 mmol, 0.03 mL) according to the general procedure Method B. Yield: 0.09 g (64%); white crystals; m.p. 123–125 °C; ${\left[\alpha \right]}_{D}^{20}$ = –34 (*c* 0.17 MeOH); ^1^H-NMR (500 MHz, CDCl_3_) δ (ppm): 0.65 (s, 3H), 0.78–0.84 (m, 1H), 0.89 (s, 3H), 0.91–1.06 (m, 5H), 1.10–1.16 (m, 4H), 1.26–1.44 (m, 5H), 1.58–1.63 (m, 2H), 1.68–1.69 (m, 2H), 1.77–1.82 (m, 2H), 2.13 (d, 1H, *J* = 13.7 Hz), 2.31 (d, 1H, *J* = 12.6 Hz), 2.79 (t, 1H, *J* = 12.9 Hz), 3.03 (dd, 1H, *J* = 3.5 Hz, 12.1 Hz), 3.59 (s, 3H), 3.66 (d, 1H, *J* = 4.8 Hz), 3.75 (s, 3H), 4.00 (d, 1H, *J* = 13.1 Hz), 4.20 (d, 1H, *J* = 13.1 Hz), 6.91 (d, 2H, *J* = 8.5 Hz), 7.54 (d, 2H, *J* = 8.5 Hz); ^13^C-NMR (125 MHz, CDCl_3_) δ (ppm): 13.2 (CH_3_), 18.8 (CH_2_), 19.4 (CH_2_), 21.8 (CH_2_), 24.7 (CH_3_), 28.9 (CH_3_), 33.0 (CH_2_), 34.8 (CH_2_), 37.9 (CH_2_), 38.0 (C_q_), 39.5 (CH_2_), 41.4 (C_q_), 42.9 (C_q_), 43.6 (C_q_), 43.7 (CH), 48.7 (CH_2_), 49.9 (CH_2_), 51.3 (CH_3_), 53.4 (CH_2_), 55.2 (CH_3_), 56.9 (CH), 57.8 (CH), 84.9 (CH), 114.4 (2xCH), 122.3 (C_q_), 131.8 (2xCH), 160.2 (C_q_), 177.6 (C=O). HRMS (ESI+): *m*/*z* calcd. for C_30_H_46_NO_4_ [M + H]^+^ 484.3427; found 484.3421.

**(4*R*,6a*S*,7*R*,8*R*,9*S*,11b*S*)-Methyl 7-(((4-fluorobenzyl)amino)methyl)-8-hydroxy-4,9,11b-trimethyltetradecahydro-6a,9-methanocyclohepta[a]naphthalene-4-carboxylate (12)**: The reaction was accomplished starting from compound 6 with 4-fluorobenzaldehyde (0.28 mmol, 0.03 mL), according to the general procedure Method B. Yield: 0.09 g (65%); white crystals; m.p. 118–119 °C; ${\left[\alpha \right]}_{D}^{20}$ = –27 (*c* 0.15 MeOH); ^1^H-NMR (500 MHz, CDCl_3_) δ (ppm): 0.70 (s, 3H), 0.82–0.87 (m, 1H), 0.91–0.92 (m, 4H), 0.96–1.06 (m, 4H), 1.15–1.21 (m, 4H), 1.34 (dd, 1H, *J* = 2.2 Hz, 11.8 Hz), 1.40 (d, 1H, *J* = 14.1 Hz), 1.58–1.88 (m, 10H), 2.16 (d, 1H, *J* = 13.1 Hz), 2.34 (d, 1H, *J* = 11.8 Hz), 3.00 (dd, 1H, *J* = 3.9 Hz, 10.7 Hz), 3.45 (d, 1H, *J* = 4.7 Hz), 3.60 (s, 3H), 3.73 (d, 1H, *J* = 13.0 Hz), 3.84 (d, 1H, *J* = 13.0 Hz), 7.00 (t, 2H, *J* = 8.5 Hz), 7.26–7.31 (m, 2H); ^13^C-NMR (125 MHz, CDCl_3_) δ (ppm): 13.1 (CH_3_), 19.0 (CH_2_), 19.6 (CH_2_), 22.1 (CH_2_), 25.0 (CH_3_), 28.9 (CH_3_), 33.0 (CH_2_), 35.0 (CH_2_), 38.0 (CH_2_), 38.1 (C_q_), 39.6 (CH_2_), 40.7 (C_q_), 42.3 (C_q_), 43.8 (C_q_), 48.4 (CH), 51.1 (CH_3_), 51.7 (CH_2_), 53.4 (CH_2_), 54.3 (CH_2_), 57.2 (CH), 57.8 (CH), 88.6 (CH), 115.0 (CH), 115.2 (CH), 129.5 (CH), 129.6 (CH), 136.3 (C_q-F_), 136.4 (C_q-F_) 161.0 (C_q-F_), 162.9 (C_q-F_), 177.8 (C=O); ^19^F-NMR (470 MHz, CDCl_3_) δ (ppm): -116.2 (C_q-F_). C_29_H_42_FNO_3_ (471.65): 472.32. HRMS (ESI+): *m*/*z* calcd. for C_29_H_43F_NO_3_ [M + H]^+^ 472.3227; found 472.3222.

**General procedure for the preparation ofamino ketones:** To a solution of isosteviol methyl ester **13** (1.80 mmol, 0.60 g) in glacial acetic acid (4 mL), paraformaldehyde (3.60 mmol, 0.10 g) and then secondary amine hydrochlorides (1.80 mmol) was added, and the mixture was treated under reflux conditions for 1.5 h. The solvent was evaporated, and the residue was dissolved in DCM (100 mL). The solution was washed with 5% aqueous KOH (100 mL) and the aqueous phase was extracted with DCM (2 × 100 mL). The combined organic layer was dried with Na_2_SO_4_, filtered, and evaporated. The crude product was purified by column chromatography on silica gel (CHCl_3_/MeOH = 19:1).

**(4*R*,6a*S*,7*R*,9*S*,11b*S*)-Methyl 4,9,11b-trimethyl-7-(morpholinomethyl)-8-oxotetradecahydro-6a,9-methanocyclohepta[a]naphthalene-4-carboxylate (14)**: The reaction was accomplished starting from compound 13 with morpholine hydrochloride (1.80 mmol, 0.22 g) according to the general procedure. Yield: 0.53 g (68%); white crystals; m.p. 148–149 °C; ${\left[\alpha \right]}_{D}^{20}$ = –33 (*c* 0.56 MeOH); ^1^H-NMR (500 MHz, CDCl_3_) δ (ppm): 0.69 (s, 3H), 0.87–0.90 (m, 1H), 0.93 (s, 3H), 1.17–1.24 (m, 7H), 1.26–1.27 (m, 1H), 1.32–1.38 (m, 1H), 1.57–1.60 (m, 1H), 1.68–1.72 (m, 3H), 1.78–1.88 (m, 2H), 1.97–2.02 (m, 1H), 2.10–2.14 (m, 1H), 2.18 (d, 1H, *J* = 13.3 Hz), 2.31 (d, 1H, *J* = 10.1 Hz), 2.39–2.41 (m, 2H), 2.48–2.55 (m, 4H), 3.63 (s, 3H), 3.68–3.75 (m, 4H); ^13^C-NMR (125 MHz, CDCl_3_) δ (ppm): 13.0 (CH_3_), 19.0 (CH_2_), 19.5 (CH_2_), 20.2 (CH_3_), 22.1 (CH_2_), 28.9 (CH_3_), 36.2 (CH_2_), 37.3 (CH_2_), 38.2 (CH_2_), 38.2 (C_q_), 39.6 (CH_2_), 41.6 (C_q_), 43.8 (C_q_), 48.0 (C_q_), 50.9 (CH), 51.4 (CH_3_), 53.0 (CH_2_), 54.1 (2xCH_2_), 57.1 (CH), 57.3 (CH), 57.7 (CH_2_), 67.1 (2xCH_2_), 177.8 (C=O), 224.1 (C=O). HRMS (ESI+): *m*/*z* calcd. for C_26_H_44_NO_4_ [M + H]^+^ 432.3114; found 432.3108.

**(4*R*,6a*S*,7*R*,9*S*,11b*S*)-Methyl 7-((benzyl(methyl)amino)methyl)-4,9,11b-trimethyl-8-oxotetradecahydro-6a,9-methanocyclohepta[a]naphthalene-4-carboxylate (15)**: The reaction was accomplished starting from compound 13 with *N*-methyl-N-benzylamine hydrochloride (1.80 mmol, 0.28 g) according to the general procedure. Yield: 0.40 g (47%); white crystals; m.p. 154–155 °C; ${\left[\alpha \right]}_{D}^{20}$ = –40 (*c* 0.73 MeOH); ^1^H-NMR (500 MHz, CDCl_3_) δ (ppm): 0.71 (s, 3H), 0.87–0.90 (m, 1H), 0.93 (s, 3H), 0.99–1.05 (m, 1H), 1.16–1.17 (m, 1H), 1.21 (m, 4H), 1.23–1.29 (m, 2H), 1.32–1.38 (m, 1H), 1.43 (d, 1H, *J* = 14.1 Hz), 1.60 (d, 1H, J = 12.3 Hz), 1.67–1.70 (m, 3H), 1.79–1.86 (m, 2H), 2.02–2.10 (m, 2H), 2.15–2.19 (m, 4H), 2.35 (d, 1H, *J* = 4.0 Hz, 12.6 Hz), 2.26–2.60 (m, 1H), 2.66–2.70 (m, 1H), 3.43 (d, 1H, *J* = 13.1 Hz), 3.51 (s, 3H), 3.59 (d, 1H, *J* = 13.1 Hz), 7.22–7.25 (m, 1H), 7.30–7.36 (m, 4H); ^13^C-NMR (125 MHz, CDCl_3_) δ (ppm): 13.1 (CH_3_), 18.9 (CH_2_), 19.6 (CH_2_), 20.2 (CH_3_), 22.5 (CH_2_), 28.8 (CH_3_), 35.9 (CH_2_), 37.5 (CH_2_), 38.0 (CH_2_), 38.2 (C_q_), 39.7 (CH_2_), 41.8 (CH_3_), 41.8 (C_q_), 43.8 (C_q_), 47.9 (C_q_), 50.9 (CH_3_), 51.3 (CH), 52.8 (CH_2_), 57.1 (CH), 57.3 (C_q_), 57.3 (CH), 63.5 (CH_2_), 126.9 (CH), 128.1 (2xCH), 129.1 (2xCH), 139.1 (C_q_), 177.9 (C=O), 224.2 (C=O). HRMS (ESI+): *m*/*z* calcd. for C_30_H_44_NO_3_ [M + H]^+^ 466.3321; found 466.3316.

**(4*R*,6a*S*,7*R*,9*S*,11b*S*)-Methyl 4,9,11b-trimethyl-8-oxo-7-(pyrrolidin-1-ylmethyl)tetradecahydro-6a,9-methanocyclohepta[a]naphthalene-4-carboxylate (16)**: The reaction was accomplished starting from compound 13 with pyrrolidine hydrochloride (1.80 mmol, 0.19 g) according to the general procedure. Yield: 0.44 g (59%); white crystals; m.p. 138–139 °C; ${\left[\alpha \right]}_{D}^{20}$ = –61 (*c* 0.77 MeOH); ^1^H-NMR (500 MHz, CDCl_3_) δ (ppm): 0.69 (s, 3H), 0.86–0.92 (m, 1H), 0.95 (s, 3H), 0.99–1.05 (m, 1H), 1.12–1.24 (m, 7H), 1.24–1.26 (m, 1H), 1.31–1.37 (m, 1H), 1.40–1.44 (m, 1H), 1.57–1.60 (m, 1H), 1.66–1.70 (m, 2H), 1.74–1.85 (m, 7H), 1.87–1.93 (m, 1H), 2.10–2.18 (m, 2H), 2.45–2.52 (m, 6H), 2.72–2.76 (m, 1H), 3.62 (s, 3H); ^13^C-NMR (125 MHz, CDCl_3_) δ (ppm): 13.0 (CH_3_), 18.9 (CH_2_), 19.6 (CH_2_), 20.3 (CH_3_), 21.9 (CH_2_), 23.9 (2xCH_2_), 28.7 (CH_3_), 35.8 (CH_2_), 37.6 (CH_2_), 38.1 (CH_2_), 38.2 (C_q_), 39.7 (CH_2_), 41.7 (C_q_), 43.8 (C_q_), 47.9 (C_q_), 51.0 (CH_3_), 52.3 (CH), 52.8 (CH_2_), 54.5 (2xCH_2_), 54.7 (CH_2_), 57.2 (CH), 57.3 (CH), 180.0 (C=O), 224.3 (C=O). HRMS (ESI+): *m*/*z* calcd. for C_26_H_42_NO_3_ [M + H]^+^ 416.3165; found 416.3159.

**(4*R*,6a*S*,7*R*,9*S*,11b*S*)-Methyl 7-((dimethylamino)methyl)-4,9,11b-trimethyl-8-oxotetradecahydro-6a,9-methanocyclohepta[a]naphthalene-4-carboxylate (17)**: The reaction was accomplished starting from compound 13 with dimethylamine hydrochloride (1.80 mmol, 0.15 g) according to the general procedure. Yield: 0.44 g (62%); white crystals; m.p. 129–130 °C; ${\left[\alpha \right]}_{D}^{20}$ = –66 (*c* 0.16 MeOH); ^1^H-NMR (500 MHz, CDCl_3_) δ (ppm): 0.71 (s, 3H), 0.86–0.92 (m, 1H), 0.95 (s, 3H), 0.98–1.05 (m, 1H), 1.13–1.16 (m, 2H), 1.18 (m, 4H), 1.22–1.28 (m, 2H), 1.31–1.37 (m, 1H), 1.41–1.45 (m, 1H), 1.58–1.62 (m, 1H), 1.65–1.70 (m, 2H), 1.73 (d, 1H, *J* = 2.7 Hz, 11.9 Hz),1.77–1.86 (m, 2H), 1.88–1.97 (m, 1H), 2.00–2.04 (m, 1H), 2.17 (d, 1H, *J* = 13.3 Hz), 2.23 (s, 6H), 2.24–2.28 (m, 1H), 2.44–2.50 (m, 2H), 3.62 (s, 3H); ^13^C-NMR (125 MHz, CDCl_3_) δ (ppm): 13.1 (CH_3_), 18.9 (CH_2_), 19.5 (CH_2_), 20.3 (CH_3_), 21.9 (CH_2_), 28.7 (CH_3_), 35.9 (CH_2_), 37.6 (CH_2_), 38.1 (CH_2_), 38.2 (C_q_), 39.7 (CH_2_), 41.7 (C_q_), 43.8 (C_q_), 45.9 (2xCH_3_), 48.0 (C_q_), 50.9 (CH_3_), 51.0 (CH), 52.7 (CH_2_), 57.1 (CH), 57.3 (CH), 58.4(CH_2_), 177.9 (C=O), 224.0 (C=O). HRMS (ESI+): *m*/*z* calcd. for C_24_H_40_NO_3_ [M + H]^+^ 390.3008; found 390.3004.

**(4*R*,6a*S*,7*R*,9*S*,11b*S*)-Methyl 7-((diethylamino)methyl)-4,9,11b-trimethyl-8-oxotetradecahydro-6a,9-methanocyclohepta[a]naphthalene-4-carboxylate (18)**: The reaction was accomplished starting from compound 13 with diethylamine hydrochloride (1.80 mmol, 0.20 g) according to the general procedure. Yield: 0.42 g (56%); white crystals; m.p. 110–111 °C; ${\left[\alpha \right]}_{D}^{20}$ = –18 (*c* 0.41 MeOH); ^1^H-NMR (500 MHz, CDCl_3_) δ (ppm): 0.71 (s, 3H), 0.86–0.91 (m, 1H), 0.93 (s, 3H), 0.98–1.06 (m, 7H), 1.13–1.17 (m, 2H), 1.18 (m, 4H), 1.22–1.27 (m, 2H), 1.30–1.36 (m, 1H), 1.57–1.59 (m, 1H), 1.65–1.70 (m, 3H), 1.74–1.89 (m, 2H), 1.97–2.03 (m, 1H), 2.06–2.10 (m, 1H), 2.17 (d, 1H, *J* = 13.3 Hz), 2.33 (dd, 1H, *J* = 3.7 Hz, 12.9 Hz), 2.42–2.52 (m, 3H), 2.59–2.68 (m, 3H), 3.61 (s, 3H); ^13^C-NMR (125 MHz, CDCl_3_) δ (ppm): 11.1 (2xCH_3_), 13.0 (CH_3_), 18.9 (CH_2_), 19.5 (CH_2_), 20.2 (CH_3_), 22.1 (CH_2_), 28.8 (CH_3_), 36.1 (CH_2_), 37.4 (CH_2_), 38.2 (CH_2_), 38.3 (C_q_), 39.7 (CH_2_), 41.7 (C_q_), 43.8 (C_q_), 46.3 (2xCH_2_), 47.9 (C_q_), 51.2 (CH_3_), 51.7 (CH), 53.0 (CH_2_), 53.1 (CH_2_), 57.3 (CH), 57.5 (CH), 178.0 (C=O), 224.6 (C=O). HRMS (ESI+): *m*/*z* calcd. for C_26_H_44_NO_3_ [M + H]^+^ 418.3297; found 418.3302.

**General procedure for preparation of aminoalcohols with Sodium borohydride:** To a solution of amino ketones **14**–**18** (0.92 mmol) in dry MeOH (10 mL) NaBH_4_ (1.84 mmol, 0.07 g) was added in small portions under ice cooling. After stirring for 2–3 h, the mixture was evaporated to dryness, and the residue was dissolved in H_2_O (20 mL) and extracted with DCM (3 × 20 mL). The combined organic layer was dried (Na_2_SO_4_), filtered and evaporated to dryness. The crude product obtained was purified by column chromatography on silica gel (CHCl_3_/MeOH = 19:1).

**(4*R*,6a*S*,7*R*,8*S*,9*S*,11b*S*)-Methyl 8-hydroxy-4,9,11b-trimethyl-7-(morpholinomethyl)tetradecahydro-6a,9 methanocyclohepta[a]naphthalene-4-carboxylate (19a)**: The reaction was accomplished starting from compound 14 according to the general procedure. Yield: 0.12 g (28%); white crystals; m.p. 123–124 °C; ${\left[\alpha \right]}_{D}^{20}$ = –46 (*c* 0.38 MeOH); ^1^H-NMR (500 MHz, CDCl_3_) δ (ppm): 0.69 (s, 3H), 0.75 (d, 1H, *J* = 11.3 Hz), 1.87–0.92 (m, 2H), 0.95 (s, 3H), 1.00–1.03 (m, 1H), 1.06–1.09 (m, 1H), 1.14–1.18 (m, 4H), 1.19–1.24 (m, 2H), 1.40–1.47 (m, 2H), 1.58–1.60 (m, 2H), 1.64–1.71 (m, 2H), 1.75–1.82 (m, 3H), 2.17 (d, 1H, *J* = 13.5 Hz), 2.31–2.46 (m, 4H), 2.67–2.75 (m, 3H), 3.64 (s, 3H), 3.68–3.70 (m, 4H), 3.79 (d, 1H, *J* = 6.2 Hz); ^13^C-NMR (125 MHz, CDCl_3_) δ (ppm): 13.1 (CH_3_), 19.0 (CH_2_), 19.4 (CH_2_), 21.1 (CH_3_), 22.0 (CH_2_), 28.8 (CH_3_), 34.0 (CH_2_), 38.0 (CH_2_), 38.1 (C_q_), 38.2 (CH_2_), 39.2 (CH), 39.7 (CH_2_), 42.1 (C_q_), 43.8 (C_q_), 44.8 (C_q_), 51.0 (CH_3_), 52.7 (CH_2_), 53.0 (2xCH_2_), 56.7 (CH), 57.2 (2xCH), 66.9 (2xCH_2_), 88.1 (CH), 177.8 (C=O). HRMS (ESI+): *m*/*z* calcd. for C_26_H_44_NO_4_ [M + H]^+^ 434.3270; found 434.3265.

**(4*R*,6a*S*,7*R*,8*R*,9*S*,11b*S*)-Methyl 8-hydroxy-4,9,11b-trimethyl-7-(morpholinomethyl)tetradecahydro-6a,9-methanocyclohepta[a]naphthalene-4-carboxylate (19b)**: The reaction was accomplished starting from compound 14 according to the general procedure. Yield: 0.10 g (24%); white crystals; m.p. 147–148 °C; ${\left[\alpha \right]}_{D}^{20}$ = –58 (*c* 0.41 MeOH); ^1^H-NMR (500 MHz, CDCl_3_) δ (ppm): 0.70 (s, 3H), 0.85–0.91 (m, 2H), 0.93 (s, 3H), 0.96–1.08 (m, 4H), 1.16–1.22 (m, 4H), 1.35 (dd, 1H, *J* = 2.5 Hz, 11.5 Hz), 1.40–1.43 (m, 1H), 1.58–1.65 (m, 4H), 1.68–1.72 (m, 2H), 1.76–1.82 (m, 3H), 2.00–2.02 (m, 1H), 2.15–2.23 (m, 2H), 2.34 (s, 1H), 2.53 (dd, 1H, *J* = 3.9 Hz, 11.7 Hz), 2.68 (s, 2H), 3.44 (d, 1H, *J* = 5.1 Hz), 3.63 (s, 3H), 3.66–3.68 (m, 2H), 3.72–3.76 (m, 2H); ^13^C-NMR (125 MHz, CDCl_3_) δ (ppm): 12.9 (CH_3_), 18.9 (CH_2_), 19.5 (CH_2_), 22.2 (CH_2_), 25.1 (CH_3_), 28.9 (CH_3_), 32.9 (CH_2_), 34.9 (CH_2_), 38.0 (CH_2_), 38.0 (C_q_), 39.5 (CH_2_), 40.5 (C_q_), 41.8 (C_q_), 43.7 (C_q_), 44.1 (CH), 51.1 (CH_3_), 54.2 (3xCH_2_), 57.0 (CH), 57.8 (CH), 61.9 (CH_2_), 67.3 (2xCH_2_), 88.7 (CH), 177.9 (C=O). HRMS (ESI+): *m*/*z* calcd. for C_26_H_44_NO_4_ [M + H]^+^ 434.3270; found 434.3265.

**(4*R*,6a*S*,7*R*,8*S*,9*S*,11b*S*)-Methyl 7-((benzyl(methyl)amino)methyl)-8-hydroxy-4,9,11b-trimethyltetradecahydro-6a,9-methanocyclohepta[a]naphthalene-4-carboxylate (20a)**: The reaction was accomplished starting from compound 15 according to the general procedure. Yield: 0.02 g (10%); white crystals; m.p. 125–127 °C; ${\left[\alpha \right]}_{D}^{20}$ = –37 (*c* 0.38 MeOH); ^1^H-NMR (500 MHz, CDCl_3_) δ (ppm): 0.70 (s, 3H), 0.76 (d, 1H, *J* = 11.5 Hz), 0.87–0.93 (m, 2H), 0.98 (s, 3H), 1.01–1.04 (m, 1H), 1.08 (d, 1H, *J* = 12.1 Hz), 1.16–1.25 (m, 6H), 1.40–1.47 (m, 2H), 1.57–1.62 (m, 1H), 1.65–1.71 (m, 3H), 1.78–1.82 (m, 3H), 2.15–2.21 (m, 4H), 2.41–2.50 (m, 2H), 2.90 (t, 1H, *J* = 11.8 Hz), 3.45–3.51 (m, 1H), 3.63–3.67 (m, 4H), 3.85 (d, 1H, *J* = 3.1 Hz), 7.24–7.33 (m, 5H); ^13^C-NMR (125 MHz, CDCl_3_) δ (ppm): 13.2 (CH_3_), 19.0 (CH_2_), 19.4 (CH_2_), 21.2 (CH_3_), 22.1 (CH_2_), 29.0 (CH_3_), 34.0 (CH_2_), 37.9 (CH_2_), 38.1 (CH_2_), 38.2 (CH_2_), 39.6 (CH_2_), 40.5 (CH), 40.8 (CH_3_), 42.1 (C_q_), 43.8 (C_q_), 44.9 (C_q_), 51.2 (CH_3_), 52.8 (CH_2_), 56.8 (CH_2_), 57.2 (CH), 57.2 (CH), 61.8 (CH_2_), 80.4 (CH), 127.3 (CH), 128.4 (2xCH), 128.9 (2xCH), 138.1 (C_q_), 177.9 (C=O). HRMS (ESI+): *m*/*z* calcd. for C_30_H_46_NO_3_ [M + H]^+^ 468.3478; found 468.3472.

**(4*R*,6a*S*,7*R*,8*R*,9*S*,11b*S*)-Methyl 7-((benzyl(methyl)amino)methyl)-8-hydroxy-4,9,11b-trimethyltetradecahydro-6a,9-methanocyclohepta[a]naphthalene-4-carboxylate (20b)**: The reaction was accomplished starting from compound 15 according to the general procedure. Yield: 0.18 g (42%). Alternative procedure for the synthesis of 24b: To a solution of 8 (0.12 mmol, 0.05 g) in DCM (5 mL), Et_3_N (0.12 mmol, 15 µL) and iodomethane (0.12 mmol, 7 µL) were added. The solution was stirred for 4 h at room temperature. Then water (20 mL) was added, and the mixture was extracted with DCM (3 × 15 mL). The organic phase was dried (Na_2_SO_4_) and evaporated to dryness. The crude product was purified by column chromatography on silica gel (CHCl_3_/MeOH 9:1). Yield: 0.04 g (77%); white crystals; m.p. 113–114 °C; ${\left[\alpha \right]}_{D}^{20}$ = –21 (*c* 0.24 MeOH); ^1^H-NMR (500 MHz, CDCl_3_) δ (ppm): 0.73 (s, 3H), 0.84–0.90 (m, 5H), 0.94–1.07 (m, 4H), 1.13–1.20 (m, 4H), 1.26–1.28 (m, 1H), 1.38–1.42 (m, 1H), 1.55–1.60 (m, 2H), 1.62–1.73 (m, 3H), 1.75–1.83 (m, 3H), 2.03–2.07 (m, 1H), 2.16 (d, 1H, *J* = 12.6 Hz), 2.27 (s, 3H), 2.36 (t, 1H, *J* = 11.9 Hz), 2.49–2.52 (m, 1H), 3.37–3.42 (m, 2H), 3.75 (d, 1H, *J* = 13.3 Hz), 7.23–7.28 (m, 1H), 7.30–7.32 (m, 4H); ^13^C-NMR (125 MHz, CDCl_3_) δ (ppm): 13.0 (CH_3_), 19.0 (CH_2_), 19.6 (CH_2_), 22.2 (CH_2_), 25.1 (CH_3_), 28.9 (CH_3_), 33.1 (CH_2_), 34.9 (CH_2_), 38.0 (CH_2_), 38.1 (CH_q_), 39.6 (CH_2_), 40.5 (C_q_), 42.2 (C_q_), 42.6 (CH_3_), 43.8 (C_q_), 45.2 (CH), 51.0 (CH_3_), 54.3 (CH_2_), 57.1 (CH), 58.0 (CH), 60.5 (CH_2_), 63.2 (CH_2_), 88.4 (CH), 127.2 (CH), 128.4 (2xCH), 129.2 (2xCH), 139.2 (C_q_), 177.8 (C=O). HRMS (ESI+): *m*/*z* calcd. for C_30_H_46_NO_3_ [M + H]^+^ 468.3478; found 468.3472.

**(4*R*,6a*S*,7*R*,8*R*,9*S*,11b*S*)-Methyl 8-hydroxy-4,9,11b-trimethyl-7-(pyrrolidin-1-ylmethyl)tetradecahydro-6a,9-methanocyclohepta[a]naphthalene-4-carboxylate (21)**: The reaction was accomplished starting from compound 16 according to the general procedure. Yield: 0.30 g (70%); white crystals; m.p. 143–144 °C; ${\left[\alpha \right]}_{D}^{20}$ = –20 (*c* 0.35 MeOH); ^1^H-NMR (500 MHz, CDCl_3_) δ (ppm): 0.68 (s, 3H), 0.81 (d, 1H, *J* = 11.7 Hz), 0.85–0.92 (m, 2H), 0.94 (s, 3H), 0.99–1.10 (m, 1H), 1.09 (dd, 1H, *J* = 2.1 Hz, 12.1 Hz), 1.13–1.21 (m, 4H), 1.22–1.26 (m, 1H), 1.27–1.32 (m, 1H), 1.42–1.54 (m, 3H), 1.59–1.72 (m, 5H), 1.82–1.86 (m, 1H), 2.08 (s, 2H), 2.17–2.19 (m, 1H), 2.35–2.39 (m, 3H), 2.71–2.73 (m, 1H), 2.88 (s, 1H), 3.58–3.63 (m, 1H), 3.64 (s, 3H), 3.86 (s, 1H), 3.96 (s, 1 H), 4.07 (d, 1H, *J* = 6.7 Hz), 4.76 (s, 1H); ^13^C-NMR (125 MHz, CDCl_3_) δ (ppm): 13.6 (CH_3_), 19.0 (CH_2_), 19.3 (CH_2_), 21.5 (CH_2_), 21.7 (CH_3_), 23.2 (CH_2_), 23.6 (CH_2_), 29.1 (CH_3_), 34.2 (CH_2_), 37.3 (CH_2_), 37.6 (CH_2_), 37.9 (C_q_), 39.4 (CH_2_), 41.4 (CH), 42.6 (C_q_), 43.8 (C_q_), 44.9 (C_q_), 51.4 (CH_3_), 53.0 (CH_2_), 53.3 (CH_2_), 55.3 (CH_2_), 56.4 (CH_2_), 56.8 (CH), 56.9 (CH), 76.4 (CH), 177.7 (C=O). HRMS (ESI+): *m*/*z* calcd. for C_26_H_44_NO_3_ [M + H]^+^ 418.3321; found 418.3316.

**(4*R*,6a*S*,7*R*,8*R*,9*S*,11b*S*)-Methyl 7-((dimethylamino)methyl)-8-hydroxy-4,9,11b-trimethyltetradecahydro-6a,9-methanocyclohepta[a]naphthalene-4-carboxylate (22)**: The reaction was accomplished starting from compound 17 according to the general procedure. Yield: 0.32 g (75%); white crystals; m.p. 149–150 °C; ${\left[\alpha \right]}_{D}^{20}$ = –70 (*c* 0.33 MeOH); ^1^H-NMR (500 MHz, CDCl_3_) δ (ppm): 0.70 (s, 3H), 0.82 (d, 1H, *J* = 11.7 Hz), 0.85–0.91 (m, 1H), 0.92–0.96 (m, 4H), 0.99–1.05 (m, 1H), 1.09 (dd, 1H, *J* = 2.1 Hz, 12.1 Hz), 1.17 (s, 3H), 1.19–1.32 (m, 3H), 1.42–1.53 (m, 3H), 1.60–1.64 (m, 3H), 1.70–1.77 (m, 2H), 1.83–1.86 (m, 1H), 2.18 (d, 1H, *J* = 13.4 Hz),1.42–1.47 (m, 2H), 2.73 (dd, 1H, *J* = 2.0 Hz, 11.6 Hz), 2.84 (s, 6H), 3.40 (t, 1H, *J* = 12.4 Hz), 3.63 (s, 3H), 4.07 (d, 1H, *J* = 6.8 Hz); ^13^C-NMR (125 MHz, CDCl_3_) δ (ppm): 13.6 (CH_3_), 18.9 (CH_2_), 19.3 (CH_2_), 21.5 (CH_3_), 21.5 (CH_2_), 29.0 (CH_3_), 34.2 (CH_2_), 37.3 (CH_2_), 37.6 (CH_2_), 38.0 (C_q_), 39.4 (CH_2_), 40.3 (CH), 41.7 (CH_3_), 42.6 (C_q_), 43.8 (C_q_), 44.9 (C_q_), 45.9 (CH_3_), 51.3 (CH_3_), 52.9 (CH_2_), 56.9 (CH), 57.0 (CH), 57.7 (CH_2_), 76.7 (CH), 177.6 (C=O), 224.0 (C=O). HRMS (ESI+): *m*/*z* calcd. for C_24_H_42_NO_3_ [M + H]^+^ 392.3165; found 392.3159.

**(4*R*,6a*S*,7*R*,8*S*,9*S*,11b*S*)-Methyl 7-((diethylamino)methyl)-8-hydroxy-4,9,11b-trimethyltetradecahydro-6a,9-methanocyclohepta[a]naphthalene-4-carboxylate (23a)**: The reaction was accomplished starting from compound 18 according to the general procedure. Yield: 0.04 g (10%); white crystals; m.p. 111–112 °C; ${\left[\alpha \right]}_{D}^{20}$ = –51 (*c* 0.24 MeOH); ^1^H-NMR (500 MHz, CDCl_3_) δ (ppm): 0.69 (s, 3H), 0.82 (d, 1H, *J* = 11.6 Hz), 0.85–0.88 (m, 1H), 0.92–0.95 (m, 4H), 1.00–1.05 (m, 1H), 1.10 (dd, 1H, *J* = 1.7 Hz, 11.9 Hz), 1.14–1.20 (m, 4H), 1.26–1.32 (m, 2H), 1.40–1.54 (m, 9H), 1.60–1.63 (m, 3H), 1.70–1.78 (m, 2H), 1.86 (m, 1 H, *J* = 13.9 Hz), 2.19 (d, 1H, *J* = 13.4 Hz), 2.42–2.45 (m, 1H), 2.75 (d, 1H, *J* = 10.5 Hz), 3.16 (m, 3H), 3.35 (t, 1H, *J* = 12.6 Hz), 3.61 (s, 3H), 4.07 (d, 1H, *J* = 6.1 Hz), 4.79 (s, 1H); ^13^C-NMR (125 MHz, CDCl_3_) δ (ppm): 7.4 (CH_3_), 9.0 (CH_3_), 13.5 (CH_3_), 18.9 (CH_2_), 19.3 (CH_2_), 21.6 (CH_3_), 21.7 (CH_2_), 28.9 (CH_3_), 34.3 (CH_2_), 37.3 (CH_2_), 37.7 (CH_2_), 38.0 (C_q_), 39.4 (CH_2_), 39.8 (CH), 42.6 (C_q_), 43.8 (C_q_), 44.7 (CH_2_), 45.1 (C_q_), 49.2 (CH_2_), 50.8 (CH_2_), 51.2 (CH_3_), 53.0 (CH_2_), 56.9 (CH), 57.0 (CH), 76.4 (CH), 177.6 (C=O). HRMS (ESI+): *m*/*z* calcd. for C_26_H_46_NO_3_ [M + H]^+^ 420.3478; found 420.3472.

**(4*R*,6a*S*,8*R*,9*S*,11b*S*)-Methyl 7-((diethylamino)methyl)-8-hydroxy-4,9,11b-trimethyltetradecahydro-6a,9-methanocyclohepta[a]naphthalene-4-carboxylate (23b)**: The reaction was accomplished starting from compound 18 according to the general procedure. Yield: 0.04 g (10%); white crystals; m.p. 120–121 °C; ${\left[\alpha \right]}_{D}^{20}$ = –41 (*c* 0.11 MeOH); ^1^H-NMR (500 MHz, CDCl_3_) δ (ppm): 0.72 (s, 3H), 0.87–0.90 (m, 1H), 0.94 (s, 3H), 0.99–1.05 (m, 3H), 1.08–1.12 (m, 1H), 1.15–1.22 (m, 5H), 1.33–1.36 (m, 4H), 1.43–1.46 (m, 1H), 1.51–1.54 (m, 5H), 1.61–1.64 (m, 2H), 1.73–1.80 (m, 2H), 1.84–1.90 (m, 2 H), 2.19 (d, 1H, *J* = 13.0 Hz), 2.26 (d, 1H, *J* = 13.0 Hz), 2.94 (t, 1H, *J* = 12.7 Hz), 3.01–3.03 (m, 1H), 3.17–3.27 (m, 3H), 3.46–3.48 (m, 1H), 3.61 (s, 3H), 3.87 (m, 1H), 4.95 (s, 1H); ^13^C-NMR (125 MHz, CDCl_3_) δ (ppm): 6.8 (CH_3_), 9.2 (CH_3_), 13.2 (CH_3_), 18.9 (CH_2_), 19.5 (CH_2_), 22.0 (CH_2_), 24.6 (CH_3_), 28.9 (CH_3_), 33.0 (CH_2_), 34.9 (CH_2_), 37.7 (CH_2_), 38.1 (C_q_), 39.4 (CH_2_), 41.6 (C_q_), 42.4 (CH), 43.2 (C_q_), 43.8 (C_q_), 44.3 (CH_2_), 49.5 (CH_2_), 51.2 (CH_3_), 53.6 (CH_2_), 55.7 (CH_2_), 56.9 (CH), 57.8 (CH), 84.3 (CH), 177.6 (C=O). HRMS (ESI+): *m*/*z* calcd. for C_26_H_46_NO_3_ [M + H]^+^ 420.3478; found 420.3472.

**(4*R*,6a*S*,7*R*,8*R*,9*S*,11b*S*)-Methyl 8-hydroxy-4,9,11b-trimethyl-7-((methylamino)methyl)tetradecahydro-6a,9-methanocyclohepta[a]naphthalene-4-carboxylate (24)**: To a suspension of palladium-on-carbon (5% Pd/C, 0.10 g) in MeOH (15 mL), 20a was added (0.24 mmol, 0.10 g) in MeOH (15 mL), and the mixture was stirred under a H_2_ atmosphere (1 atm) at room temperature. After completion of the reaction (as monitored by TLC, 24 h), the mixture was filtered through a Celite pad, and the solution was evaporated to dryness. The crude product was purified by column chromatography on silica gel (CHCl_3_/MeOH = 9:1). Yield: 0.02 g (28%); white crystals; m.p. 122–123 °C; ${\left[\alpha \right]}_{D}^{20}$ = –65 (*c* 0.19 MeOH); ^1^H-NMR (500 MHz, CDCl_3_) δ (ppm): 0.70 (s, 3H), 0.79 (d, 1H, *J* = 11.6 Hz), 0.85–0.92 (m, 2H), 0.94 (s, 3H), 0.98–1.04 (m, 1H), 1.08 (d, 1H, *J* = 11.8 Hz), 1.17 (m, 3H), 1.21–1.26 (s, 3H), 1.40–1.44 (m, 1H), 1.48–1.54 (m, 2H), 1.58–1.83 (m, 6H), 2.18 (d, 1H, *J* = 13.3 Hz), 2.41–2.44 (m, 1H), 2.55–2.57 (m, 1H), 2.68 (s, 3H), 3.20 (m, 1H), 3.63 (s, 3H), 3.97 (d, 1H, *J* = 6.6 Hz); ^13^C-NMR (125 MHz, CDCl_3_) δ (ppm): 13.4 (CH_3_), 18.9 (CH_2_), 19.3 (CH_2_), 21.3 (CH_3_), 21.7 (CH_2_), 29.0 (CH_3_), 34.1 (CH_2_), 37.5 (CH_2_), 37.7 (CH_2_), 38.0 (CH_2_), 39.5 (CH_2_), 40.3 (CH), 42.4 (C_q_), 43.8 (C_q_), 44.1 (CH_3_), 44.9 (C_q_), 51.2 (CH_3_), 52.8 (CH_2_), 57.0 (CH), 57.0 (CH), 57.8 (CH_2_), 77.8 (CH), 177.6 (C=O). HRMS (ESI+): *m*/*z* calcd. for C_23_H_40_NO_3_ [M + H]^+^ 378.3008; found 478.3003.

**(4*R*,6a*S*,7*R*,8*R*,9*S*,11b*S*)-Methyl 8-hydroxy-4,9,11b-trimethyl-7-((3-phenylthioureido)methyl)tetradecahydro-6a,9-methanocyclohepta[a]naphthalene-4-carboxylate (25)**: Compound 6 (0.20 g, 0.55 mmol) was dissolved in DCM (10 mL) and phenyl isothiocyanate (0.55 mmol, 0.06 mL) was added. The reaction mixture was stirred for 2 h at room temperature. After completion of the reaction (monitored by TLC), the mixture was extracted with water (3 × 10 mL). The water phase was extracted with DCM (3 × 10 mL) and the combined organic phase was dried with Na_2_SO_4_ and concentrated under vacuum. The crude product was purified by column chromatography on silica gel with CHCl_3_/MeOH 9:1. Yield: 0.25 g (91%); white crystals; m.p. 153–154 °C; ${\left[\alpha \right]}_{D}^{20}$ = +9 (*c* 0.48 MeOH); ^1^H-NMR (500 MHz, CDCl_3_) δ (ppm): 0.72 (s, 3H), 0.84–0.86 (m, 1H), 0.87 (s, 3H), 0.95–1.01 (m, 3H), 1.04–1.12 (m, 2H), 1.16 (s, 3H), 1.19–1.24 (m, 1H), 1.38–1.43 (m, 2H), 1.46–1.50 (m, 1H), 1.57–1.59 (m, 1H), 1.64–1.77 (m, 4H), 1.79–1.83 (m, 2H), 1.99–2.02 (m, 1H), 2.16 (d, 1H, *J* = 13.3 Hz), 3.40 (d, 1H, *J* = 4.9 Hz), 1.43–1.46 (m, 1H), 3.63 (s, 3H), 3.92–3.97 (m, 1H), 6.52 (s, 1H), 7.25–7.29 (m, 3H), 7.41 (t, 2H, *J* = 8.3 Hz); ^13^C-NMR (125 MHz, CDCl_3_) δ (ppm): 13.0 (CH_3_), 18.9 (CH_2_), 19.4 (CH_2_), 22.2 (CH_2_), 24.7 (CH_3_), 28.7 (CH_3_), 32.9 (CH_2_), 34.9 (CH_2_), 38.1 (CH_2_), 38.1 (C_q_), 39.7 (CH_2_), 41.1 (C_q_), 42.5 (C_q_), 43.7 (C_q_), 46.9 (CH), 48.8 (CH_2_), 51.3 (CH_3_), 53.8 (CH_2_), 57.1 (CH), 57.6 (CH), 87.6 (CH), 125.0 (2xCH), 127.0 (CH), 130.0 (2xCH), 136.3 (C_q_), 177.9 (C=O), 181.0 (C_q_). HRMS (ESI+): *m*/*z* calcd. for C_29_H_43_N_2_O_3_S [M + H]^+^ 498.2994; found 498.2989.

**(4*R*,6a*S*,7*R*,8*R*,9*S*,11b*S*)-Methyl 8-hydroxy-4,9,11b-trimethyl-7-((((methylthio)(phenylimino)methyl)amino)methyl)tetradecahydro-6a,9-methanocyclohepta[a]naphthalene-4-carboxylate (26)**: To a solution of 25 (0.30 mmol, 0.15 g), EtOH (10 mL) and iodomethane (1.50 mmol, 0.14 mL) were added. The solution was stirred for 2 h at room temperature then water (20 mL) was added, and the mixture was extracted with DCM (3 × 15 mL). The organic phase was dried with Na_2_SO_4_ and evaporated to dryness. The crude product was purified by column chromatography on silica gel (CHCl_3_/MeOH 9:1). Yield: 0.11 g (69%); white crystals; m.p. 164–165 °C; ${\left[\alpha \right]}_{D}^{20}$ = –92 (*c* 0.10 MeOH); ^1^H-NMR (500 MHz, CDCl_3_) δ (ppm): 0.71 (s, 3H), 0.82–0.88 (m, 4H), 0.92–1.07 (m, 5H), 1.12–1.17 (m, 4H), 1.29–1.32 (m, 2H), 1.38–1.43 (m, 1H), 1.51–1.70 (m, 4H), 1.67–1.70 (m, 2H), 1.76–1.82 (m, 2H), 1.87–1.89 (m, 1H), 2.16 (d, 1H, *J* = 13.3 Hz), 2.83 (t, 1H, *J* = 10.8 Hz), 3.30 (s, 1H), 3.47 (d, 1H, *J* = 12.0 Hz), 3.64 (s, 3H), 3.86 (s, 3H), 4.28 (s, 1H), 6.92 (d, 2H, *J* = 7.6 Hz), 6.98 (t, 1H, *J* = 7.4 Hz), 7.26–7.30 (m, 3H); ^13^C-NMR (125 MHz, CDCl_3_) δ (ppm): 12.8 (CH_3_), 18.9 (CH_2_), 19.4 (CH_2_), 22.1 (CH_2_), 24.9 (CH_3_), 28.9 (CH_3_), 33.0 (CH_2_), 34.9 (CH_2_), 38.0 (CH_2_), 38.1 (C_q_), 39.6 (CH_2_), 41.0 (C_q_), 42.4 (C_q_), 43.8 (C_q_), 44.4 (CH_2_), 47.5 (CH), 51.2 (CH_3_), 53.5 (CH_3_), 53.9 (CH_2_), 57.2 (CH), 57.6 (CH), 87.5 (CH), 122.4 (CH), 122.9 (2xCH), 129.4 (2xCH), 148.8 (C_q_), 154.0 (C_q_), 177.8 (C=O). HRMS (ESI+): *m*/*z* calcd. for C_30_H_45_N_2_O_3_S [M + H]^+^ 513.3151; found 513.3145.

**(4*R*,6a*S*,7*R*,8*R*,9*S*,11b*S*)-Methyl 8-hydroxy-4,9,11b-trimethyl-7-((((ethylthio)(phenylimino)methyl)amino)methyl)tetradecahydro-6a,9-methanocyclohepta[a]naphthalene-4-carboxylate (28)**: A solution of 25 (0.60 mmol, 0.30 g) in dry EtOH (10 mL) was added EtOH containing 18% hydrochloric acid (10 mL) and mixture was stirred at reflux temperature for 12 h. The reaction mixture was then concentrated under vacuum, dissolved in water (20 mL) and extracted with DCM (3 × 15 mL). The combined organic layer was washed with saturated NaCl aqueous solution (15 mL), dried (Na_2_SO_4_) and concentrated under vacuum. The crude product was purified by column chromatography on silica gel with CHCl_3_/MeOH 9:1.Yield: 0.15 g (46%); white crystals; m.p. 114–115 °C; ${\left[\alpha \right]}_{D}^{20}$ = –37 (*c* 0.28 MeOH); ^1^H-NMR (500 MHz, CDCl_3_) δ (ppm): 0.76 (s, 3H), 0.85–0.89 (m, 1H), 0.92 (s, 3H), 0.96–1.08 (m, 5H), 1.15 (s, 3H), 1.20–1.36 (m, 5H), 1.39–1.41 (m, 2H), 1.61–1.80 (m, 8H), 2.06–2.08 m, 1H), 2.16 (d, 1H, *J* = 13.3 Hz), 2.68–2.84 (m, 2H), 3.15 (t, 1H, *J* = 12.1 Hz), 3.49–3.50 (m, 1H), 3.56 (s, 3H), 3.68 (d, 1H, *J* = 9.1 Hz), 6.90 (d, 2H, *J* = 7.4 Hz), 6.99 (t, 1H, *J* = 7.4 Hz), 7.25–7.26 (m, 2H); ^13^C-NMR (125 MHz, CDCl_3_) δ (ppm): 13.0 (CH_3_), 14.6 (CH_3_), 18.9 (CH_2_), 19.5 (CH_2_), 22.2 (CH_2_), 24.8 (CH_3_), 25.3 (CH_2_), 28.8 (CH_3_), 33.0 (CH_2_), 34.9 (CH_2_), 38.1 (CH_2_), 38.2 (C_q_), 39.7 (CH_2_), 41.1 (C_q_), 42.5 (C_q_), 43.8 (C_q_), 46.1 (CH_2_), 47.3 (CH), 51.1 (CH_3_), 54.0 (CH_2_), 57.2 (CH), 57.7 (CH), 88.2 (CH), 122.3 (2xCH), 122.5 (CH), 128.7 (2xCH), 149.8 (C_q_), 177.9 (C=O). HRMS (ESI+): *m*/*z* calcd. for C_29_H_41_N_2_O_2_S [M + H]^+^ 527.3307; found 527.3301.

**Determination of antiproliferative effect:** The growth-inhibitory effects of the isosteviol-based 1,3-aminoalcohols were determined by a standard MTT (3-(4,5-dimethylthiazol-2-yl)-2,5-diphenyltetrazolium bromide) assay on a panel containing five cell lines, including Hela and Siha (cervical cancers), MDA-MB-231 and MCF-7 (breast cancers), and A2780 ovarian cancer cells [39]. All cell lines were purchased from European Collection of Cell Cultures (Salisbury, UK) except the SiHa, which was obtained from American Tissue Culture Collection (Manassas, VA, USA). The cells were maintained in minimal essential medium supplemented with 10% foetal bovine serum, 1% non-essential amino acids, and 1% penicillin-streptomycin at 37 °C in a humidified atmosphere containing 5% CO_2_. All media and supplements were obtained from Lonza Group Ltd., (Basel, Switzerland). Cancer cells were seeded into 96-well plates (5000 cells/well), after an overnight incubation the test compound was added in two different concentrations (10 µM and 30 µM) and incubated for other 72 h under cell-culturing condition. In the next step, 20 μL of 5 mg/mL MTT solution was added to each well and incubated for a further 4 h. The medium was removed, and the precipitated formazan crystals were dissolved in DMSO during 60 min of shaking at 37 °C. As the final step, the absorbance was measured at 545 nm by using a microplate reader. Untreated cells were included as controls. In the case of the most effective compounds (i.e., compounds eliciting higher than 50 or 85% at 10 or 30 μM, respectively), the assays were repeated with a set of dilutions (0.1–30 μM) in order to determine the IC_50_ values. Two independent experiments were performed with five wells for each condition. Cisplatin (Ebewe GmbH, Unterach, Austria), a clinically used anticancer agent, was used as a positive control. Calculations were performed by means of the GraphPad Prism 5.01 software (GraphPad Software Inc., San Diego, CA, USA).

## Figures and Tables

**Table 1:** Synthesis of aminoalcohols **7**–**12** via Schiff products.

Entry	Product	R^1^/R^2^	Yield [%]
1	**7**	Methyl	77
2	**8**	Benzyl	83
3	**9**	(*S*)-α-Methylbenzyl	70
4	**10**	(*R*)-α-Methylbenzyl	64
5	**11**	4-Methoxybenzyl	64
6	**12**	4-Fluorobenzyl	65

**Table 2:** Synthesis of aminoketones **14**–**18** via Mannich condensation.

Entry	Amine HCl	Product	R^1^	R^2^	Yield [%]
1	Morpholine	**14**	-CH_2_-CH_2_-O-CH_2_-CH_2_-		77
2	*N*-Methyl-*N*-benzylamine	**15**	Methyl	Benzyl	83
3	Pyrrolidine	**16**	-(CH_2_)_4_-		70
4	Dimethylamine	**17**	Methyl	Methyl	64
5	Diethylamine	**18**	Ethyl	Ethyl	64
6	Dibenzylamine	**15**	Methyl	Benzyl	65

**Table 3:** Synthesis of aminoketones **14**–**18** via Mannich condensation.

Entry	Aminoketones	Aminoalcohols	*dr* (a/b)	Yield (a/b) [%]
1	**14**	**19a, 19b**	1:1	28:24
2	**15**	**20a, 20b**	1:5	10:42
3	**16**	**21**	0:1	70
4	**17**	**22**	0:1	75
5	**18**	**23a, 23b**	1:1	10:10

**Table 4:** Antiproliferative properties of the tested diterpene analogues.

Compound	Conc. (μM)	Growth Inhibition (%) ± SEM ^[a]^ [Calculated IC_50_ (µM)]				
		HeLa	SiHa	MDA-MB-231	MCF-7	A2780
3	10 30	<20 <20	<20 <20	<20 <20	<20 <20	<20 33.22 ± 2.23
4	10 30	<20 21.47 ± 2.18	<20 26.54 ± 0.84	<20 <20	<20 <20	<20 36.90 ± 2.48
5	10 30	<20 <20	<20 <20	<20 <20	<20 <20	<20 <20
6	10 30 IC_50_	94.71 ± 0.13 95.06 ± 0.34 4.11	80.91 ± 0.70 91.52 ± 0.49 4.73	93.18 ± 0.84 97.15 ± 0.23 5.25	92.41 ± 1.34 91.89 ± 0.43 4.13	93.66 ± 0.39 93.77 ± 0.10 6.52
7	10 30 IC_50_	38.90 ± 2.34 100.18 ± 0.66 11.06	24.71 ± 2.36 100.08 ± 2.32 13.48	44.70 ± 1.61 96.15 ± 1.23 10.07	78.69 ± 0.56 100.87 ± 0.75 7.11	64.75 ± 3.04 101.13 ± 0.55 1.36
8	10 30 IC_50_	96.04 ± 0.17 96.21 ± 0.46 5.47	99.54 ± 0.50 100.06 ± 0.42 6.43	90.29 ± 0.55 96.20 ± 0.38 5.37	94.27 ± 0.39 95.32 ± 0.93 7.44	97.16 ± 0.38 97.38 ± 0.47 7.96
9	10 30 IC_50_	100.09 ± 0.29 100.24 ± 0.45 3.09	100.23 ± 0.85 100.23 ± 0.68 4.75	96.39 ± 0.67 99.76 ± 1.44 7.34	100.88 ± 0.42 101.25 ± 0.65 4.36	101.01 ± 0.30 101.07 ± 0.35 4.21
10	10 30 IC_50_	99.49 ± 0.45 99.69 ± 0.46 2.92	101.90 ± 0.44 102.41 ± 0.52 4.95	97.45 ± 0.72 96.13 ± 1.15 8.28	99.48 ± 0.77 100.41 ± 0.30 4.34	100.05 ± 0.75 101.23 ± 0.67 4.29
11	10 30 IC_50_	97.80 ± 1.27 98.16 ± 1.61 2.55	101.65 ± 0.66 102.49 ± 0.67 4.37	101.67 ± 0.90 99.45 ± 0.92 5.58	99.85 ± 0.53 100.44 ± 0.46 2.51	100.77 ± 0.22 100.78 ± 0.35 4.04
12	10 30 IC_50_	98.04 ± 1.42 99.81 ± 0.54 2.75	102.03 ± 0.47 102.91 ± 0.41 4.19	99.68 ± 0.67 99.15 ± 0.80 4.40	100.08 ± 0.21 100.12 ± 0.44 2.14	100.16 ± 0.61 100.55 ± 0.50 3.81
14	10 30 IC_50_	<20 <20	<20 40.01 ± 2.19	<20 <20	<20 <20	<20 86.09 ± 1.83 27.31
15	10 30	<20 34.61 ± 1.67	30.12 ± 2.84 53.90 ± 2.24	<20 <20	<20 <20	<20 <20
16	10 30	<20 <20	<20 <20	<20 <20	<20 <20	<20 <20
17	10 30	27.72 ± 0.98 35.73 ± 2.29	<20 20.30 ± 0.47	<20 <20	<20 <20	<20 34.07 ± 0.95
18	10 30	<20 26.53 ± 1.76	<20 <20	<20 <20	<20 <20	<20 <20
19a	10 30	49.69 ± 2.54 47.40 ± 2.02	33.90 ± 2.39 39.9 ± 2.05	<20 <20	<20 29.04 ± 2.24	49.13 ± 2.44 81.24 ± 0.62
19b	10 30	25.07 ± 2.30 43.36 ± 3.25	<20 76.41 ± 1.03	22.32 ± 1.15 27.31 ± 0.82	<20 64.26 ± 2.09	<20 68.03 ± 0.38
20a	10 30 IC_50_	50.54 ± 2.19 76.81 ± 1.25 10.17	44.71 ± 1.76 76.26 ± 0.47 12.20	52.87 ± 0.50 86.13 ± 0.57 9.20	37.27 ± 2.22 88.14 ± 2.32 17.29	36.64 ± 0.53 89.64 ± 0.82 16.08
20b	10 30 IC_50_	49.71 ± 1.38 82.06 ± 0.66 13.65	48.68 ± 2.18 99.45 ± 0.39 14.34	46.26 ± 1.63 89.73 ± 0.62 9.26	31.24 ± 1.57 89.44 ± 1.12 17.24	29.09 ± 1.16 96.18 ± 0.48 14.93
21	10 30	45.56 ± 0.46 67.21 ± 0.52	<20 25.66 ± 2.03	41.84 ± 0.52 51.39 ± 1.78	31.24 ± 0.92 84.31 ± 1.23	<20 79.72 ± 1.03
22	10 30	37.91 ± 0.44 57.71 ± 0.84	25.33 ± 2.07 75.04 ± 0.44	35.57 ± 0.40 40.20 ± 0.82	27.02 ± 1.04 74.21 ± 0.62	<20 68.28 ± 1.15
23	10 30	42.49 ± 0.68 65.17 ± 0.92	21.14 ± 1.35 79.86 ± 1.06	36.28 ± 0.59 48.41 ± 1.42	<20 72.25 ± 0.65	<20 68.28 ± 1.13
24	10 30	24.65 ± 1.66 49.66 ± 2.16	<20 45.36 ± 1.87	<20 21.00 ± 0.35	<20 75.64 ± 3.20	<20 74.59 ± 1.16
25	10 30 IC_50_	51.56 ± 1.59 98.88 ± 0.33 9.97	<20 92.46 ± 1.51 6.91	<20 95.47 ± 0.35 22.96	28.57 ± 2.62 94.16 ± 0.01 14.31	45.83 ± 1.85 95.59 ± 0.24 10.16
26	10 30 IC_50_	<20 92.08 ± 0.77 17.86	30.40 ± 3.38 90.27 ± 0.86 12.69	41.15 ± 2.07 95.37 ± 0.51 12.31	<20 44.31 ± 0.48	21.39 ± 3.18 94.66 ± 0.44 13.83
28	10 30	<20 62.35 ± 1.07	<20 51.53 ± 2.31	<20 52.63 ± 2.43	21.97 ± 2.57 81.02 ± 3.02	<20 68.62 ± 1.85
Cisplatin	10 30 IC_50_	42.61 ± 2.33 99.93 ± 0.26 12.43	60.98 ± 0.92 88.95 ± 0.53 4.29	67.51 ± 1.01 87.75 ± 1.10 3.74	53.03 ± 2.29 86.90 ± 1.24 5.78	83.57 ± 2.21 95.02 ± 0.28 1.30

^[a]^ Cancer cell growth inhibition values less than 20% were considered insignificant and are not given numerically.

## Supplementary Materials

The following are available online at https://www.mdpi.com/article/10.3390/ijms222011232/s1, ^1^H, ^13^C, COSY, HSQC, HMBC, and NOESY NMR spectra of new compounds are available.
