# Supplementary material for: Synthesis and Biological Application of Isosteviol-Based 1,3-Aminoalcohols
Source: Int J Mol Sci. 2021 Oct 18;22(20):11232. doi: 10.3390/ijms222011232 (PMC8538607; doi:10.3390/ijms222011232)

Supporting informations
for

Synthesis and biological application of isosteviol-based 1,3-aminoalcohols

Dániel Ozsvár [1], Viktória Nagy [2], István Zupkó [2,3] and Zsolt Szakonyi [1,3*]

[1] Institute of Pharmaceutical Chemistry, University of Szeged, Interdisciplinary Excellent Center, H-6720 Szeged, Eötvös utca 6, Hungary; ozsmozs88@gmail.com

[2] Department of Pharmacodynamics and Biopharmacy, University of Szeged, H-6720 Szeged, Eötvös utca 6, Hungary; zupko.istvan@szte.hu, nagy.viktoria07@gmail.com

[3] Interdisciplinary Centre of Natural Products, University of Szeged, H-6720 Szeged, Eötvös utca 6, Hungary

* Correspondence: szakonyi.zsolt@szte.hu; Tel.: +36-62-546809; Fax: +36-62-545705

Contents

^{1}H, ^{13}C NMR, COSY, NOESY, HSQC, HMBC spectra of new compounds 3 - 151

^{1}H-NMR of compound (4*R*,6a*S*,8*R*,9*S*,11b*S*)-Methyl 7-formyl-8-hydroxy-4,9,11b-trimethyltetradecahydro-6a,9-methanocyclohepta[a]naphthalene-4-carboxylate (**4**)

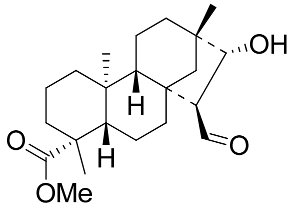

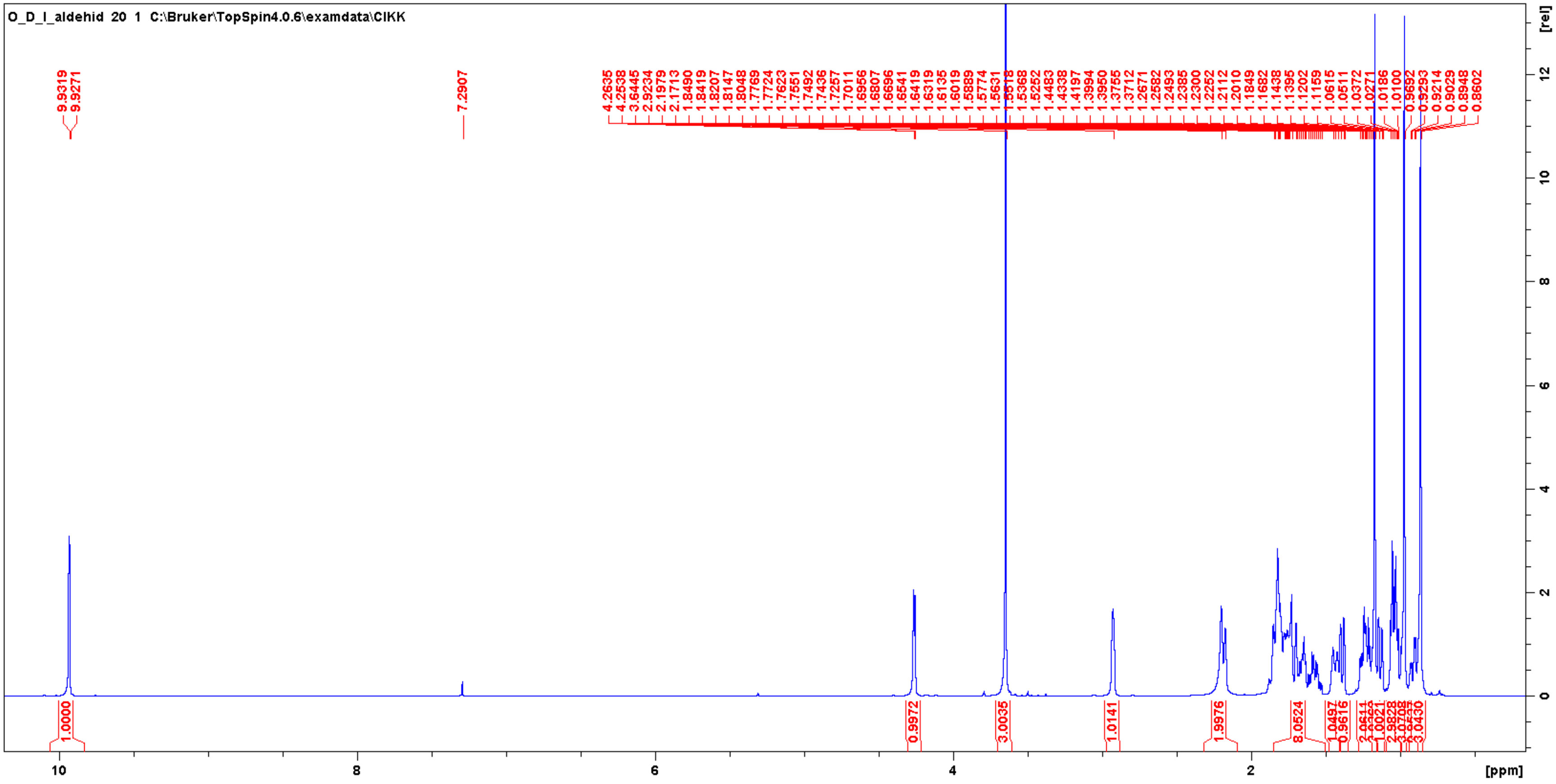

^{13}C-NMR of compound (4*R*,6a*S*,8*R*,9*S*,11b*S*)-Methyl 7-formyl-8-hydroxy-4,9,11b-trimethyltetradecahydro-6a,9-methanocyclohepta[a]naphthalene-4-carboxylate (**4**)

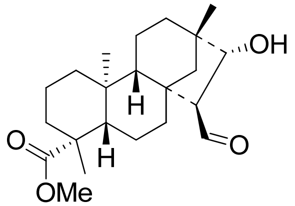

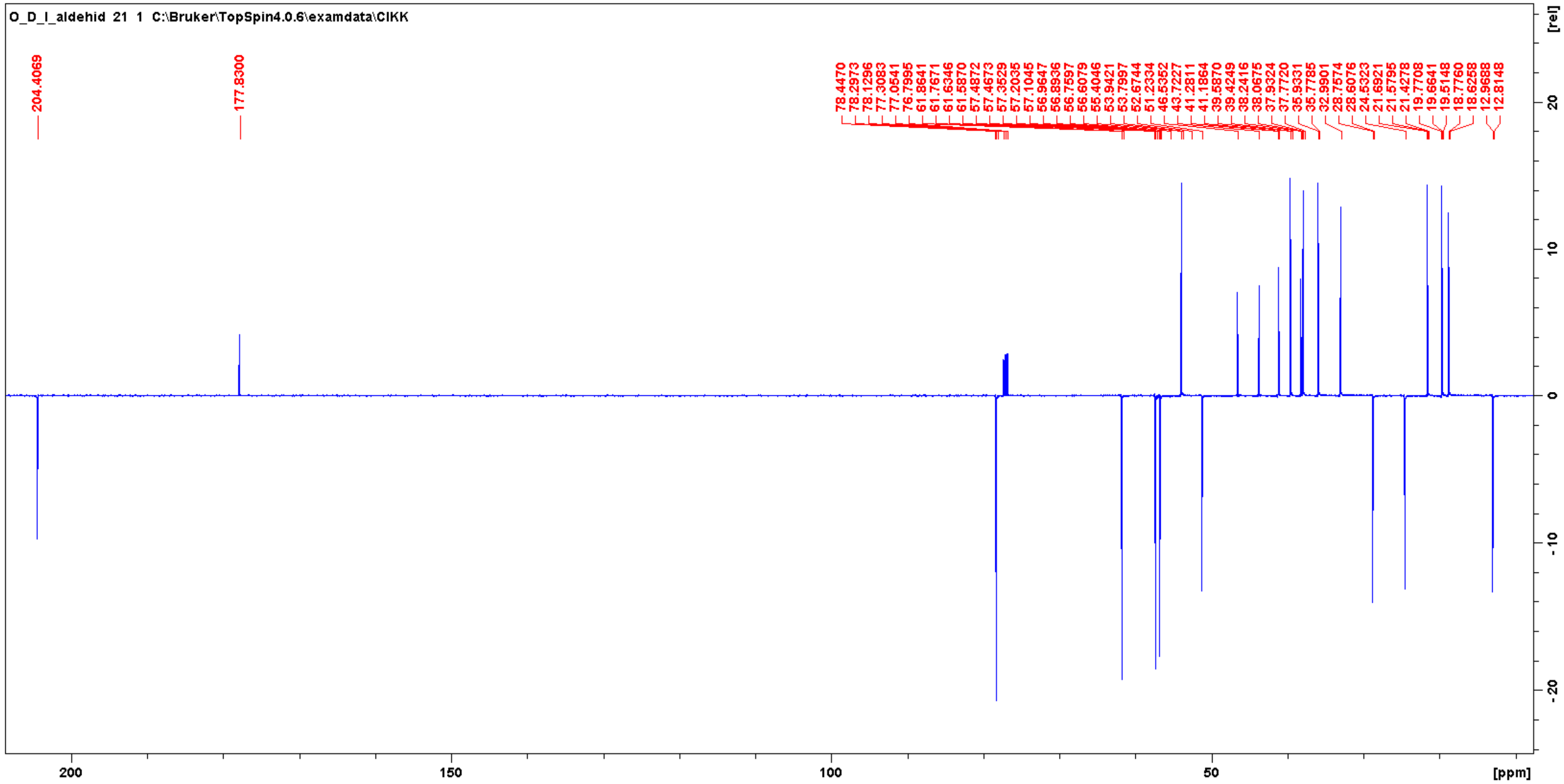

COSY of compound (4*R*,6a*S*,8*R*,9*S*,11b*S*)-Methyl 7-formyl-8-hydroxy-4,9,11b-trimethyltetradecahydro-6a,9-methanocyclohepta[a]naphthalene-4-carboxylate (**4**)

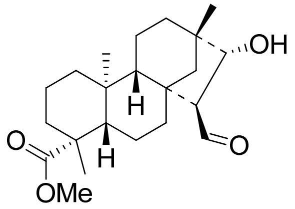

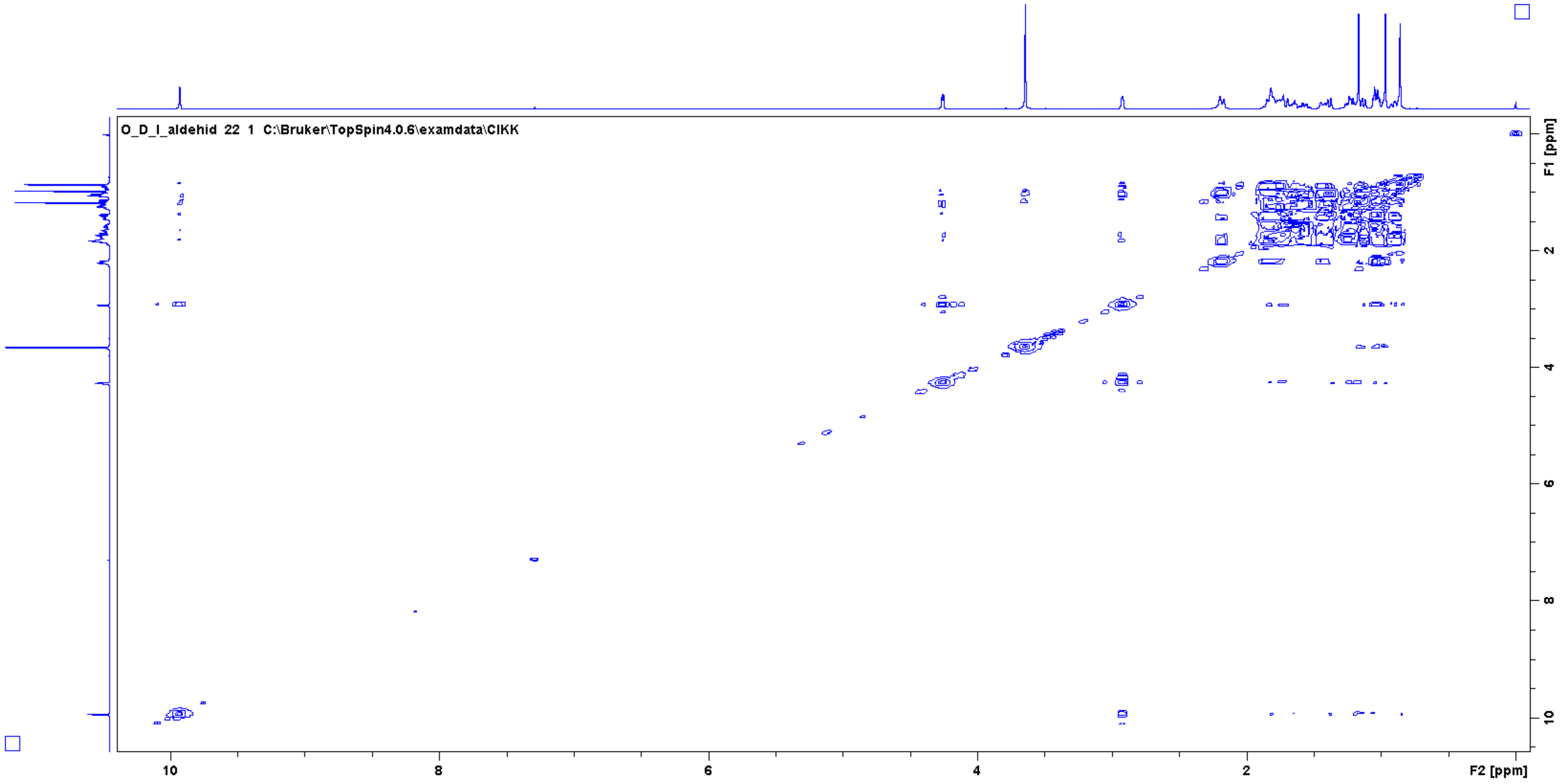

NOESY of compound (4*R*,6a*S*,8*R*,9*S*,11b*S*)-Methyl 7-formyl-8-hydroxy-4,9,11b-trimethyltetradecahydro-6a,9-methanocyclohepta[a]naphthalene-4-carboxylate (**4**)

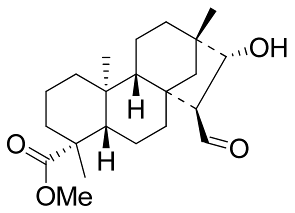

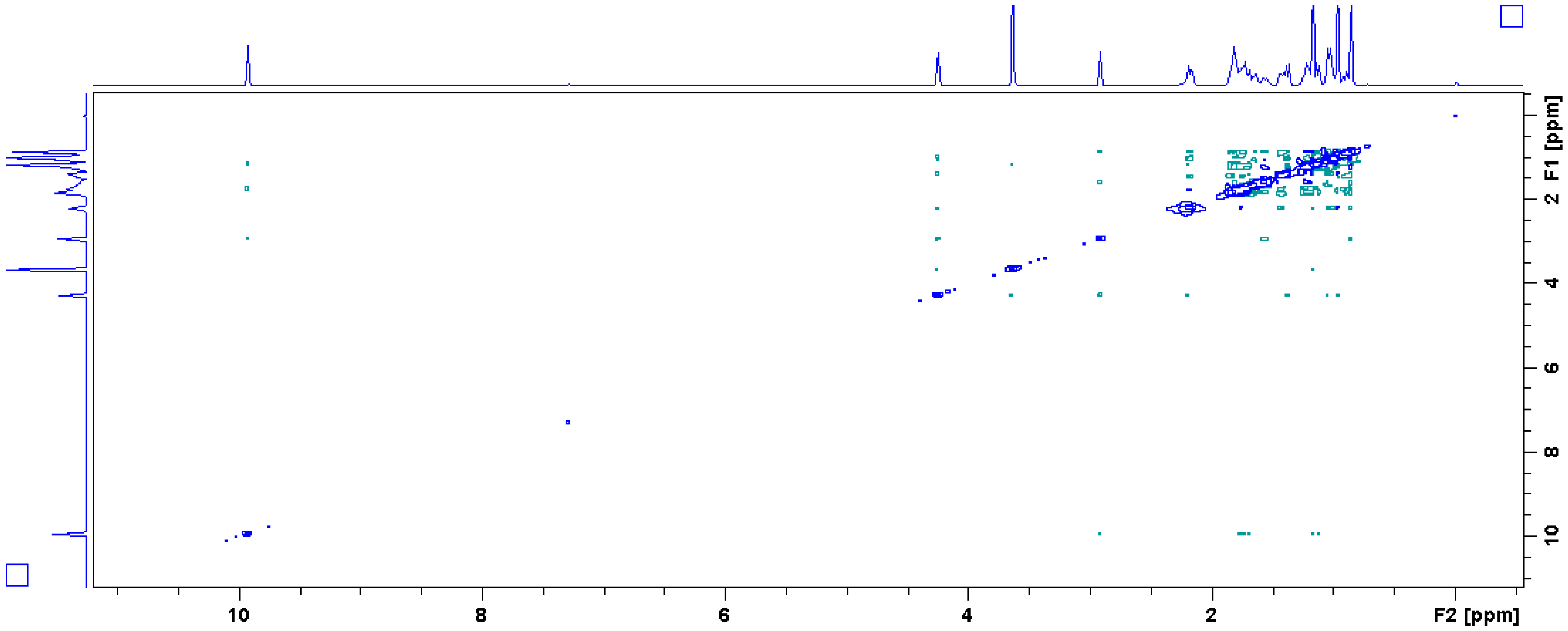

HSQC of compound (4*R*,6a*S*,8*R*,9*S*,11b*S*)-Methyl 7-formyl-8-hydroxy-4,9,11b-trimethyltetradecahydro-6a,9-methanocyclohepta[a]naphthalene-4-carboxylate (**4**)

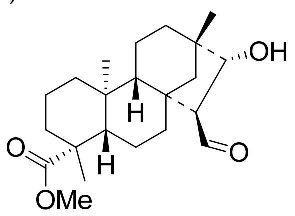

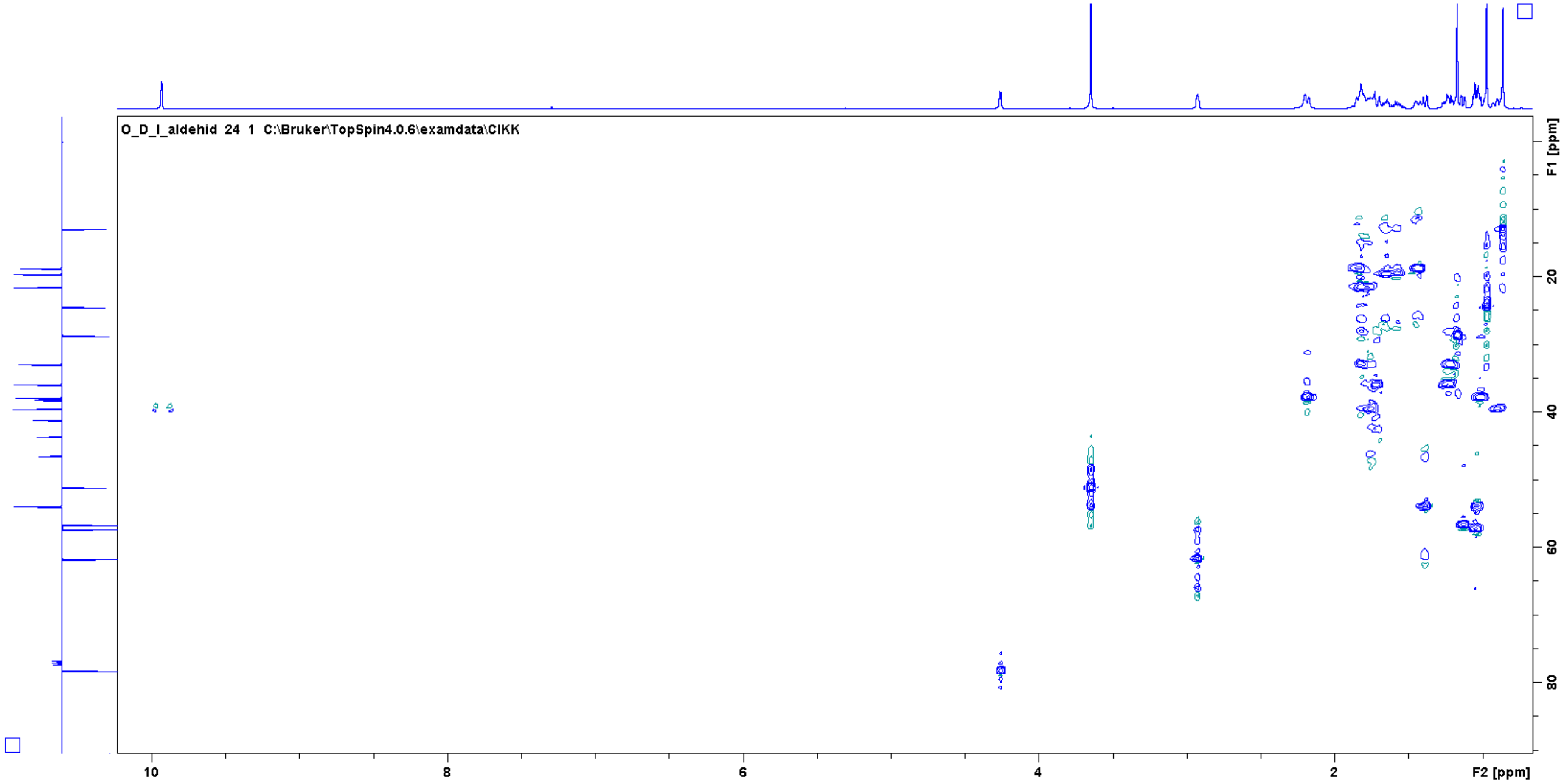

HMBC of compound (4*R*,6a*S*,8*R*,9*S*,11b*S*)-Methyl 7-formyl-8-hydroxy-4,9,11b-trimethyltetradecahydro-6a,9-methanocyclohepta[a]naphthalene-4-carboxylate (**4**)

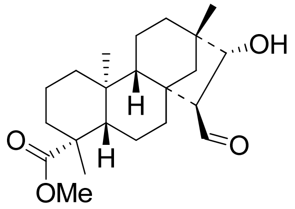

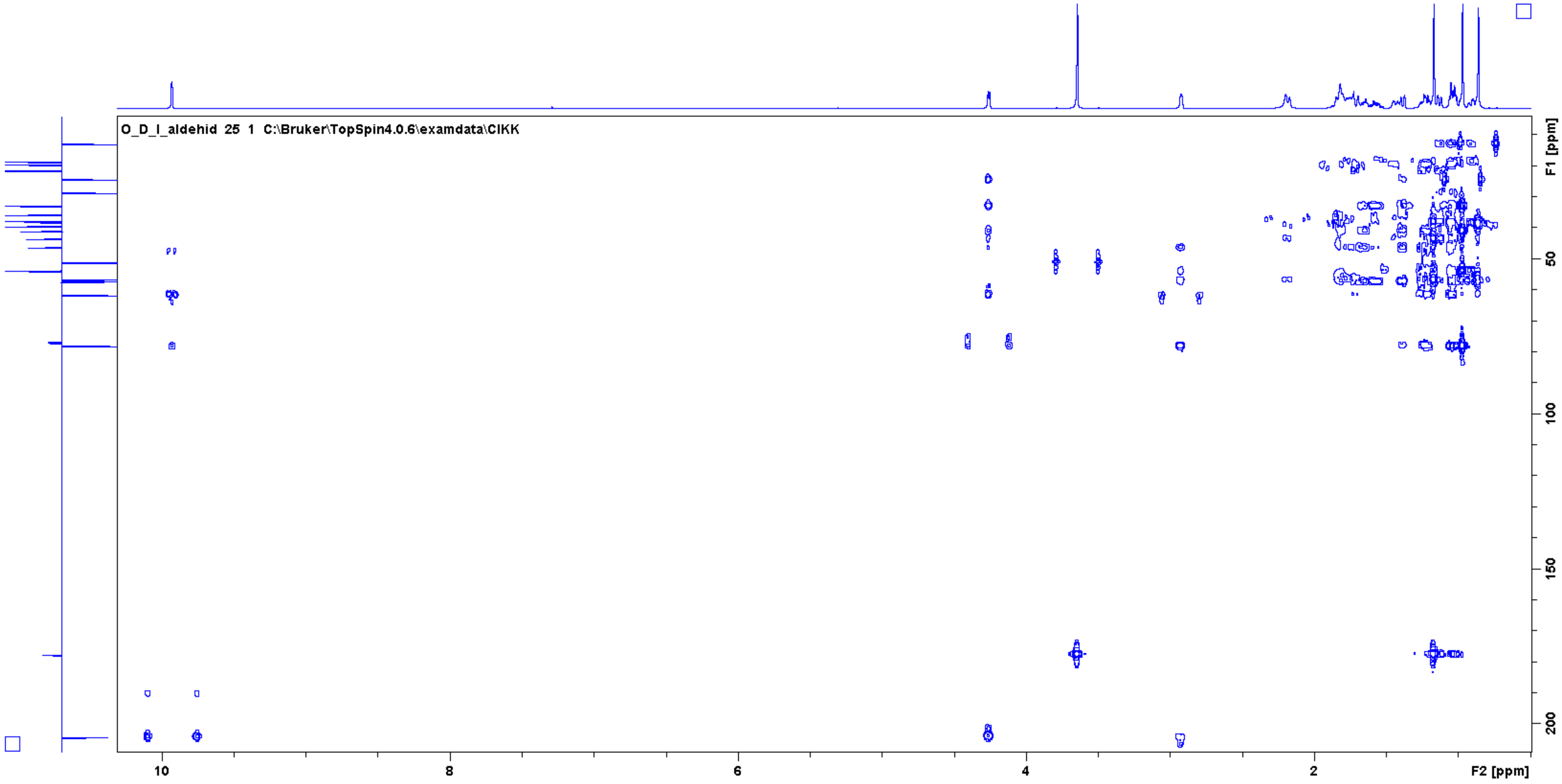

^{1}H-NMR of compound (4*R*,6a*S*,8*R*,9*S*,11b*S*)-Methyl 8-hydroxy-7-((hydroxyimino)methyl)-4,9,11b-trimethyltetradecahydro-6a,9-methanocyclohepta[a]naphthalene-4-carboxylate (**5**)

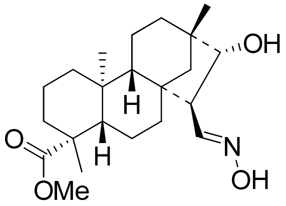

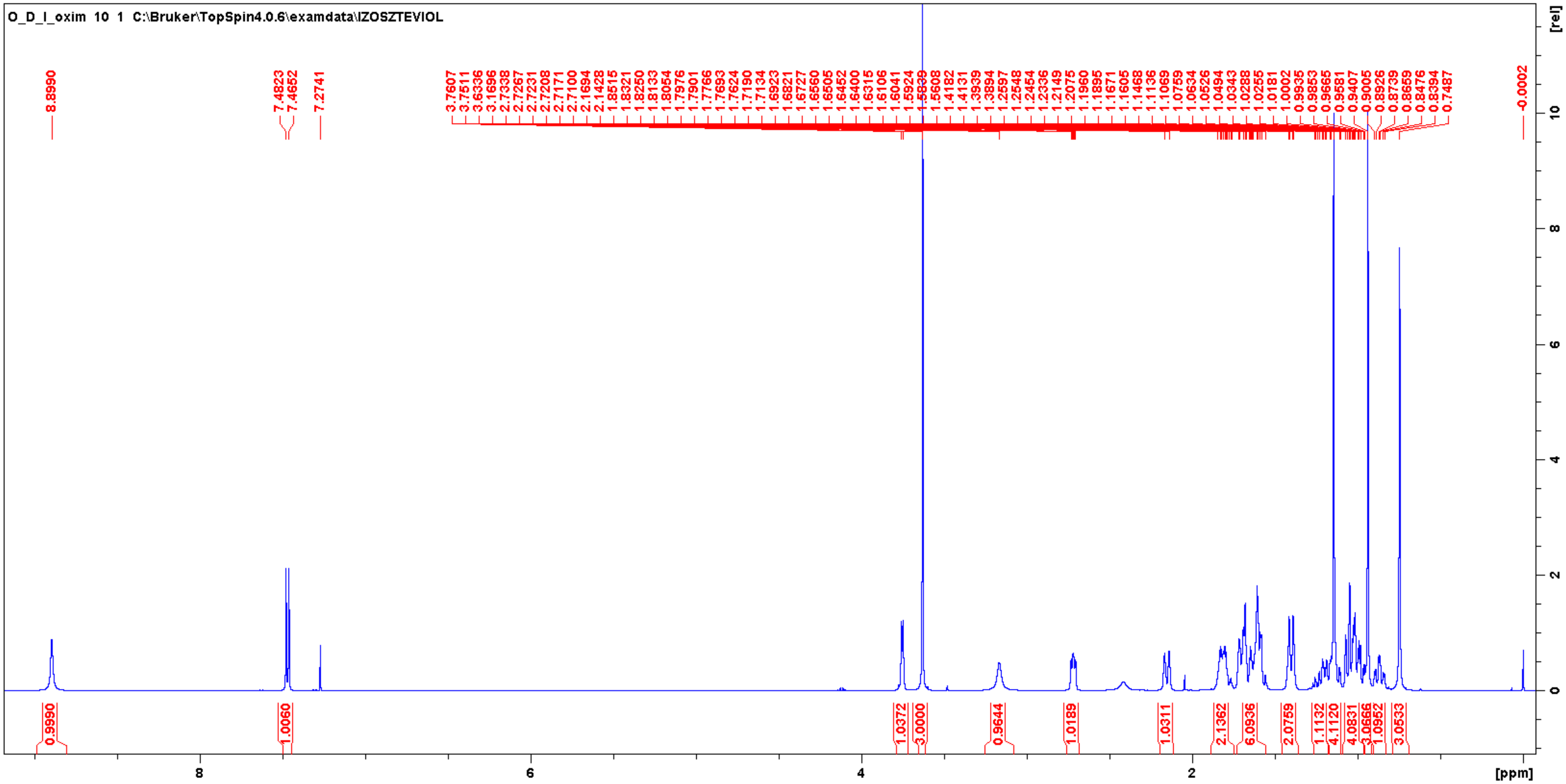

^{13}C-NMR of compound (4*R*,6a*S*,8*R*,9*S*,11b*S*)-Methyl 8-hydroxy-7-((hydroxyimino)methyl)-4,9,11b-trimethyltetradecahydro-6a,9-methanocyclohepta[a]naphthalene-4-carboxylate (**5**)

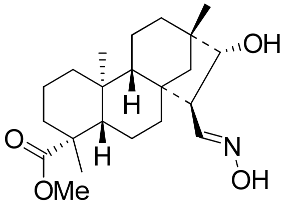

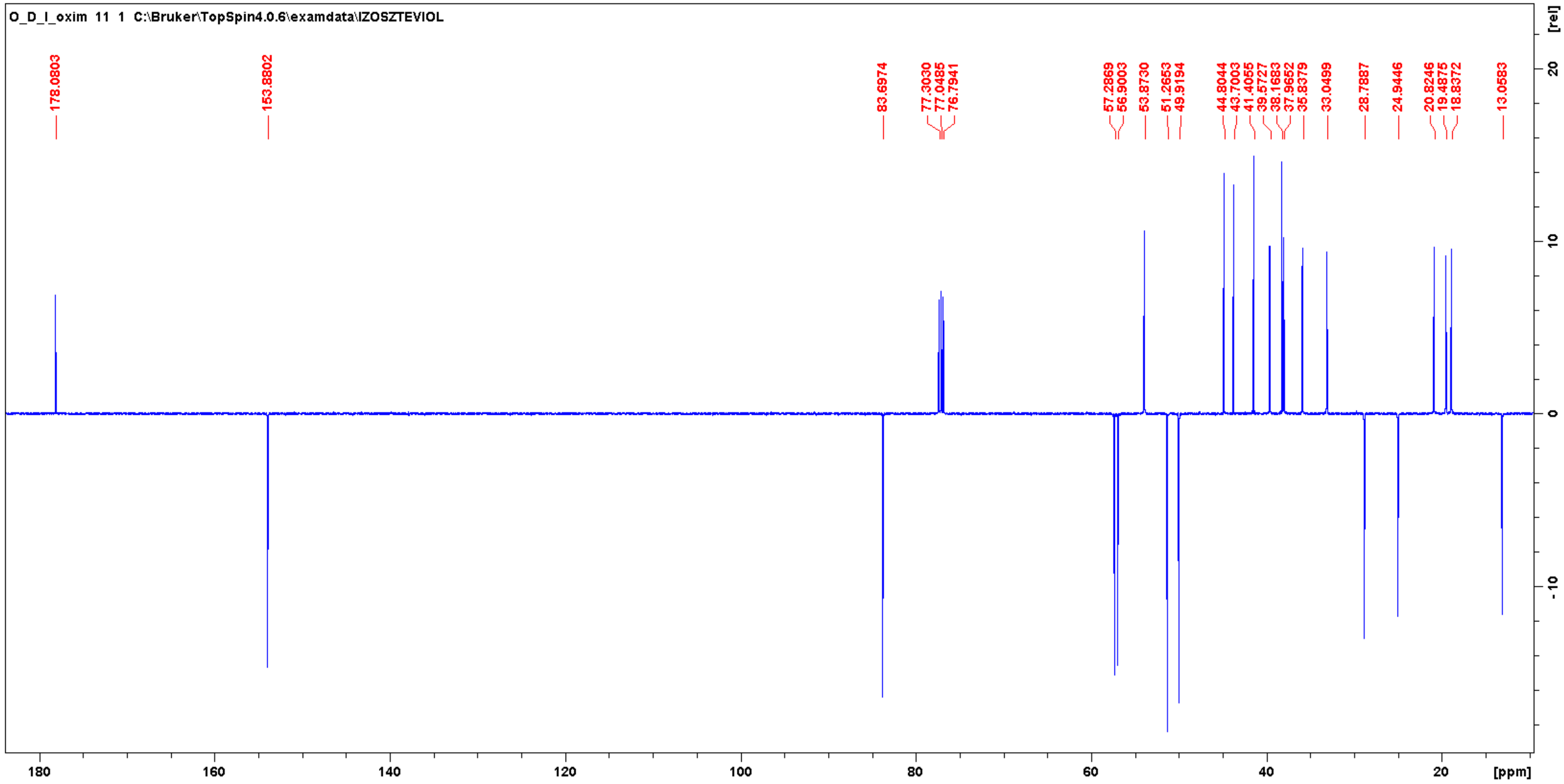

COSY of compound (4*R*,6a*S*,8*R*,9*S*,11b*S*)-Methyl 8-hydroxy-7-((hydroxyimino)methyl)-4,9,11b-trimethyltetradecahydro-6a,9-methanocyclohepta[a]naphthalene-4-carboxylate (**5**)

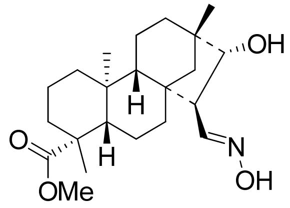

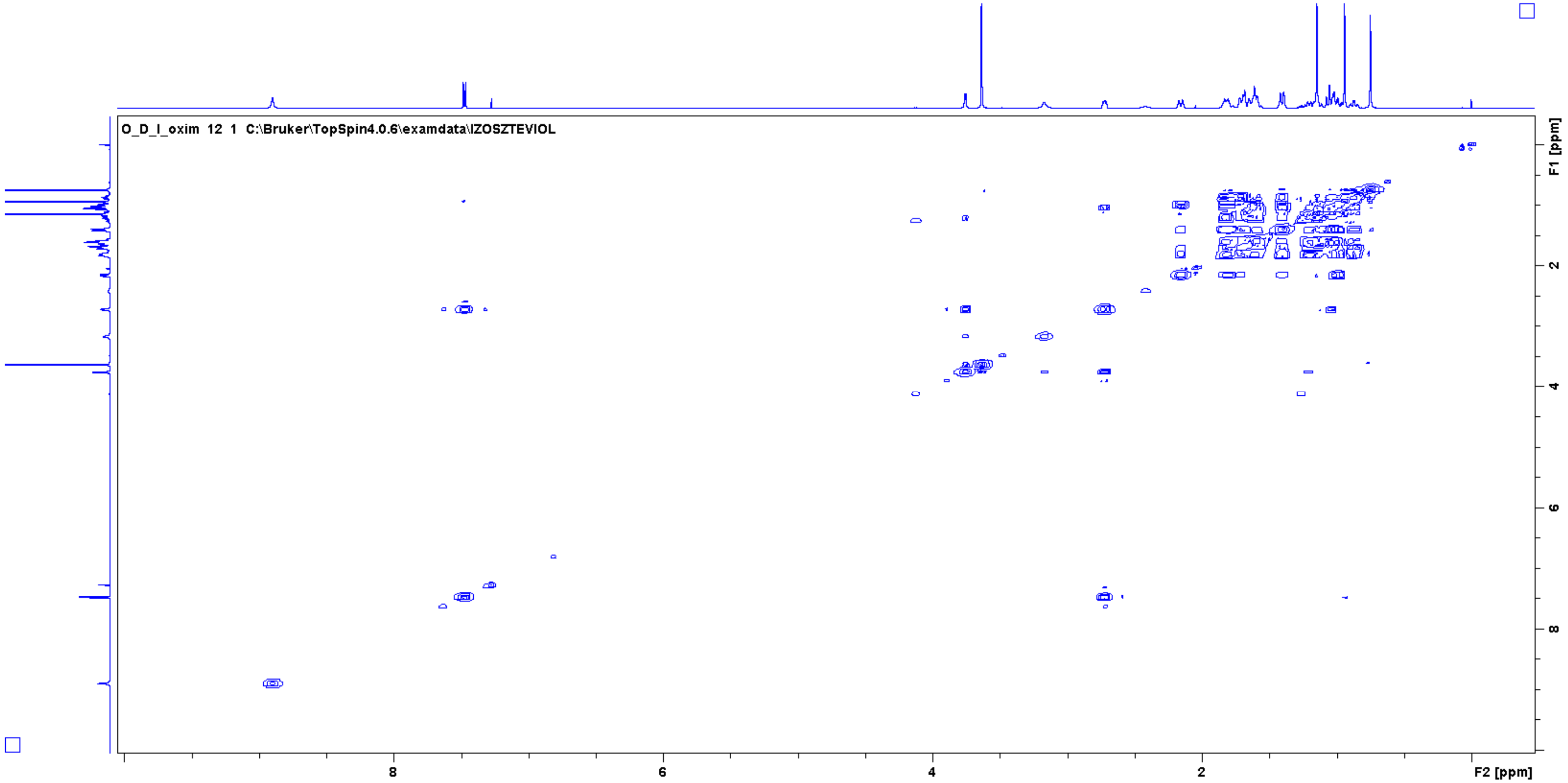

NOESY of compound (4*R*,6a*S*,8*R*,9*S*,11b*S*)-Methyl 8-hydroxy-7-((hydroxyimino)methyl)-4,9,11b-trimethyltetradecahydro-6a,9-methanocyclohepta[a]naphthalene-4-carboxylate (**5**)

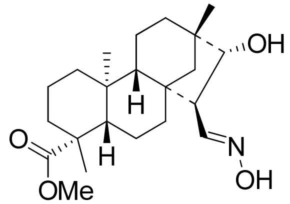

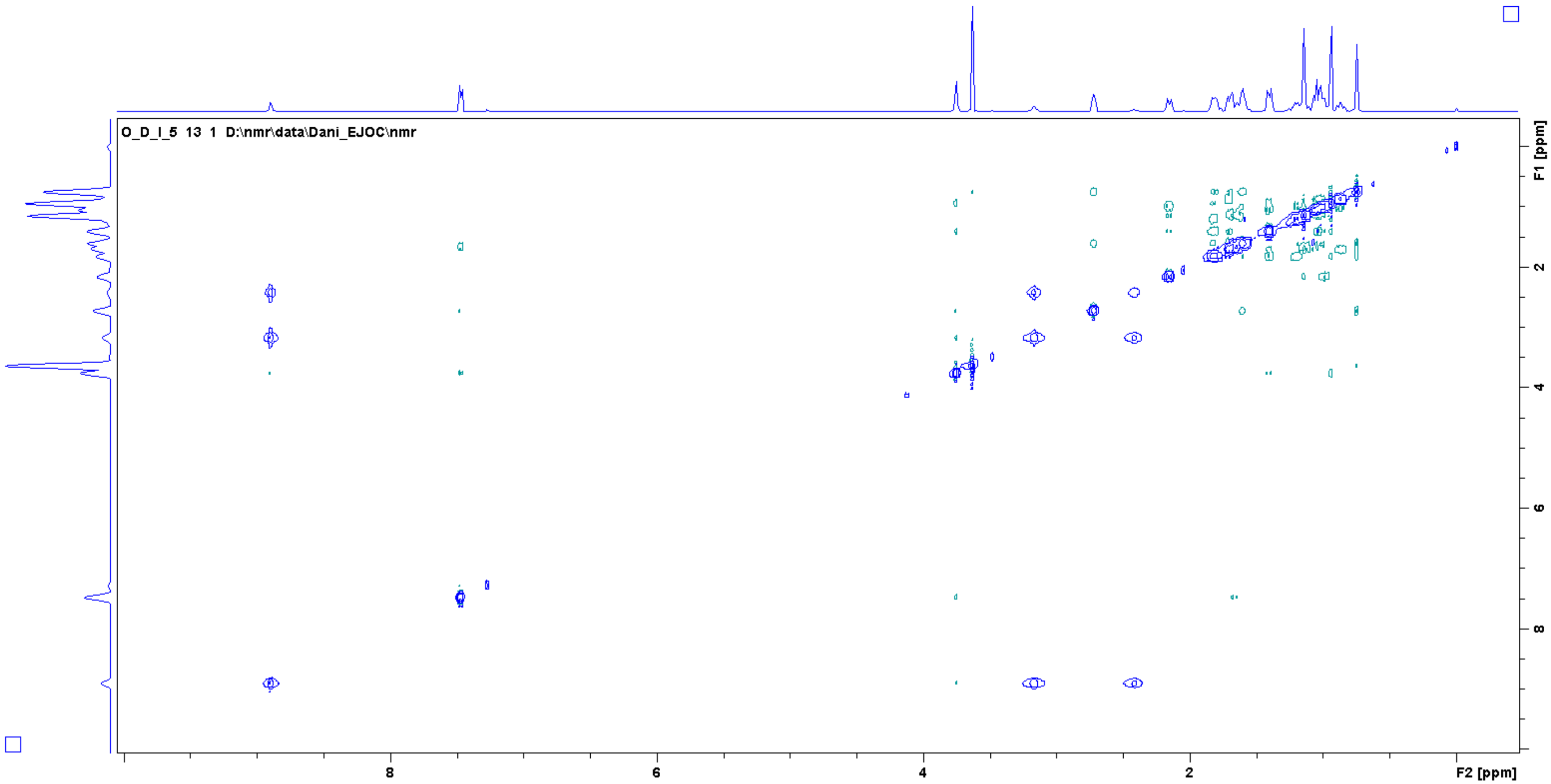

HSQC of compound (4*R*,6a*S*,8*R*,9*S*,11b*S*)-Methyl 8-hydroxy-7-((hydroxyimino)methyl)-4,9,11b-trimethyltetradecahydro-6a,9-methanocyclohepta[a]naphthalene-4-carboxylate (**5**)

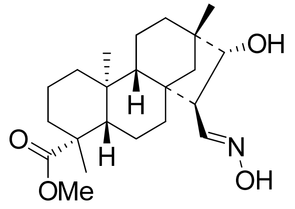

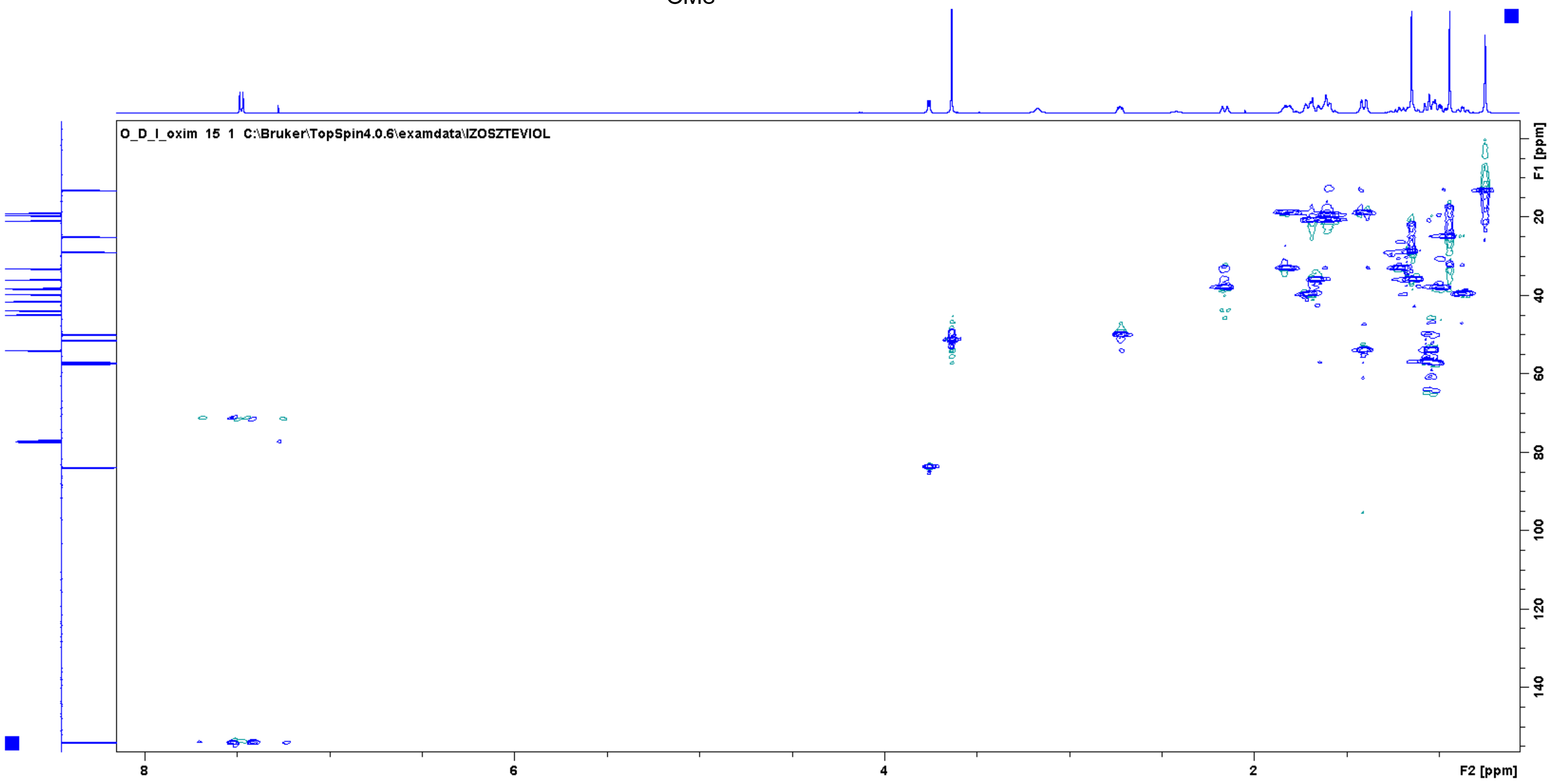

HMBC of compound (4*R*,6a*S*,8*R*,9*S*,11b*S*)-Methyl 8-hydroxy-7-((hydroxyimino)methyl)-4,9,11b-trimethyltetradecahydro-6a,9-methanocyclohepta[a]naphthalene-4-carboxylate (**5**)

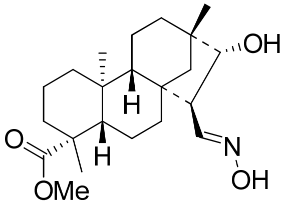

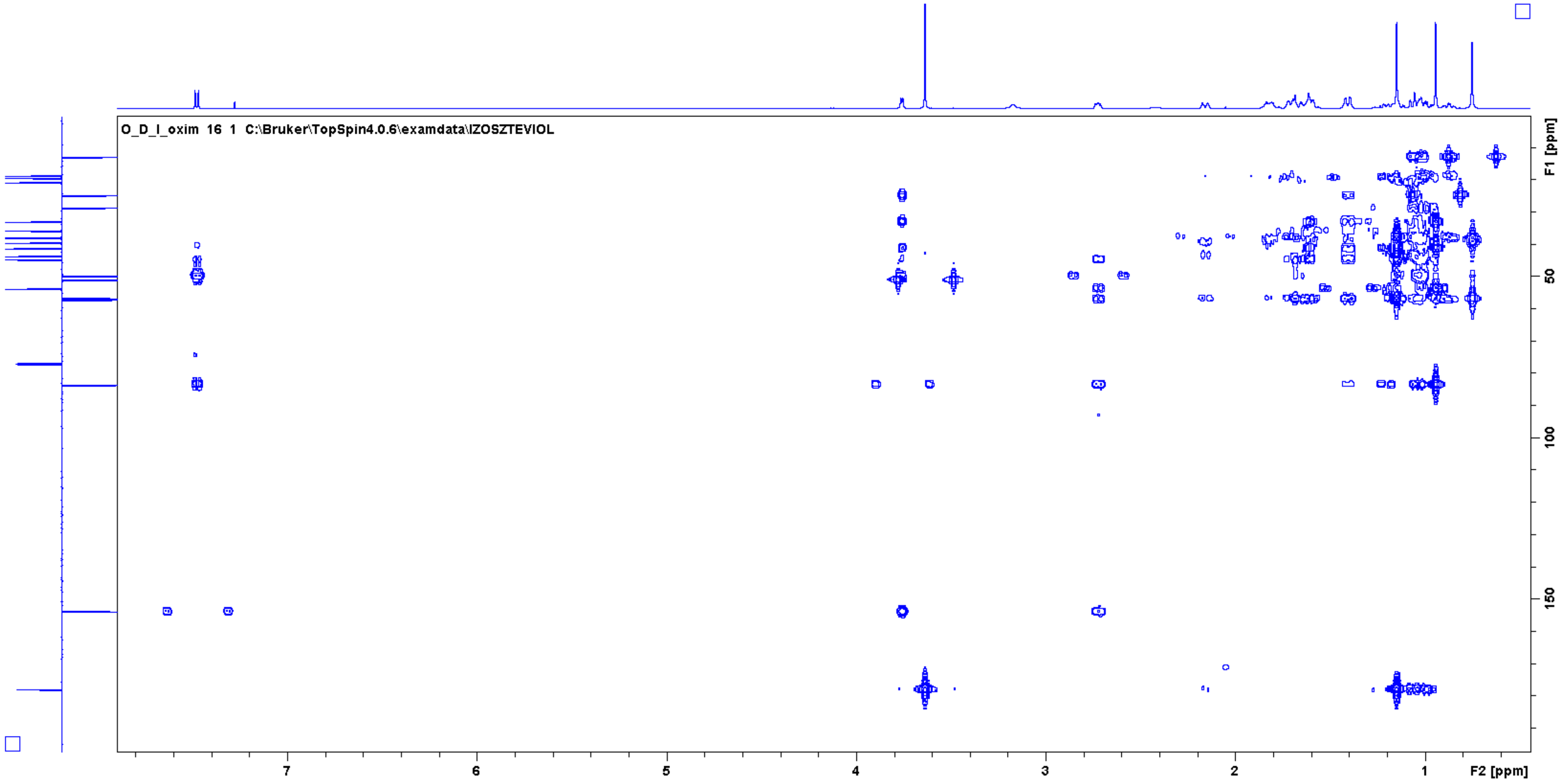

^{1}H-NMR of compound (4*R*,6a*S*,8*R*,9*S*,11b*S*)-Methyl 7-aminomethyl-8-hydroxy-4,9,11b-trimethyltetradecahydro-6a,9-methanocyclohepta[a]naphthalene-4-carboxylate (**6**)

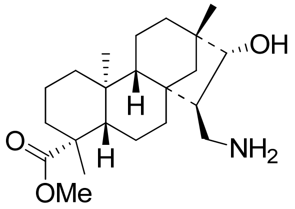

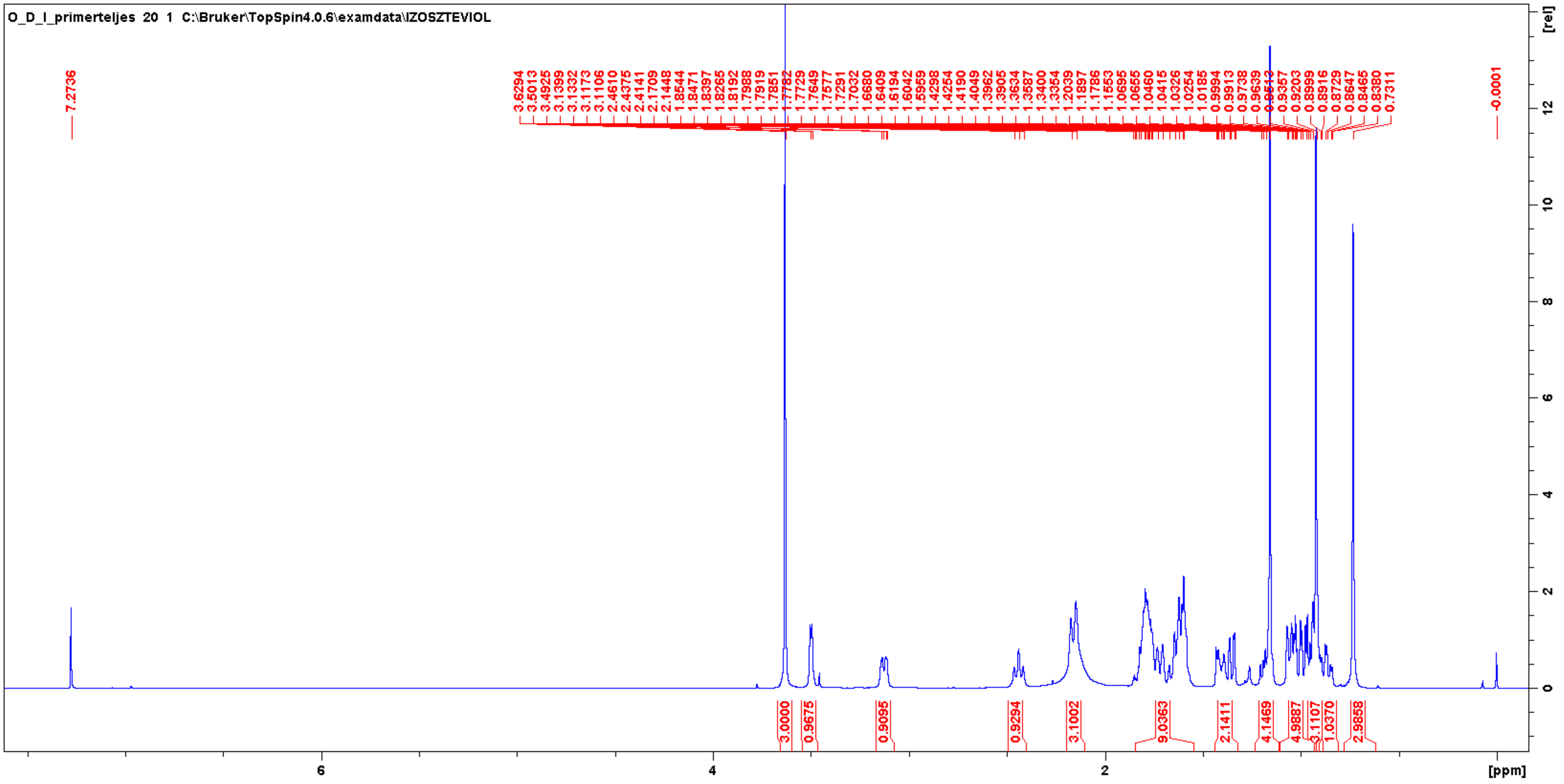

^{13}C-NMR of compound (4*R*,6a*S*,8*R*,9*S*,11b*S*)-Methyl 7-aminomethyl-8-hydroxy-4,9,11b-trimethyltetradecahydro-6a,9-methanocyclohepta[a]naphthalene-4-carboxylate (**6**)

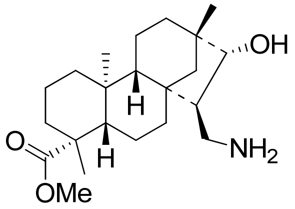

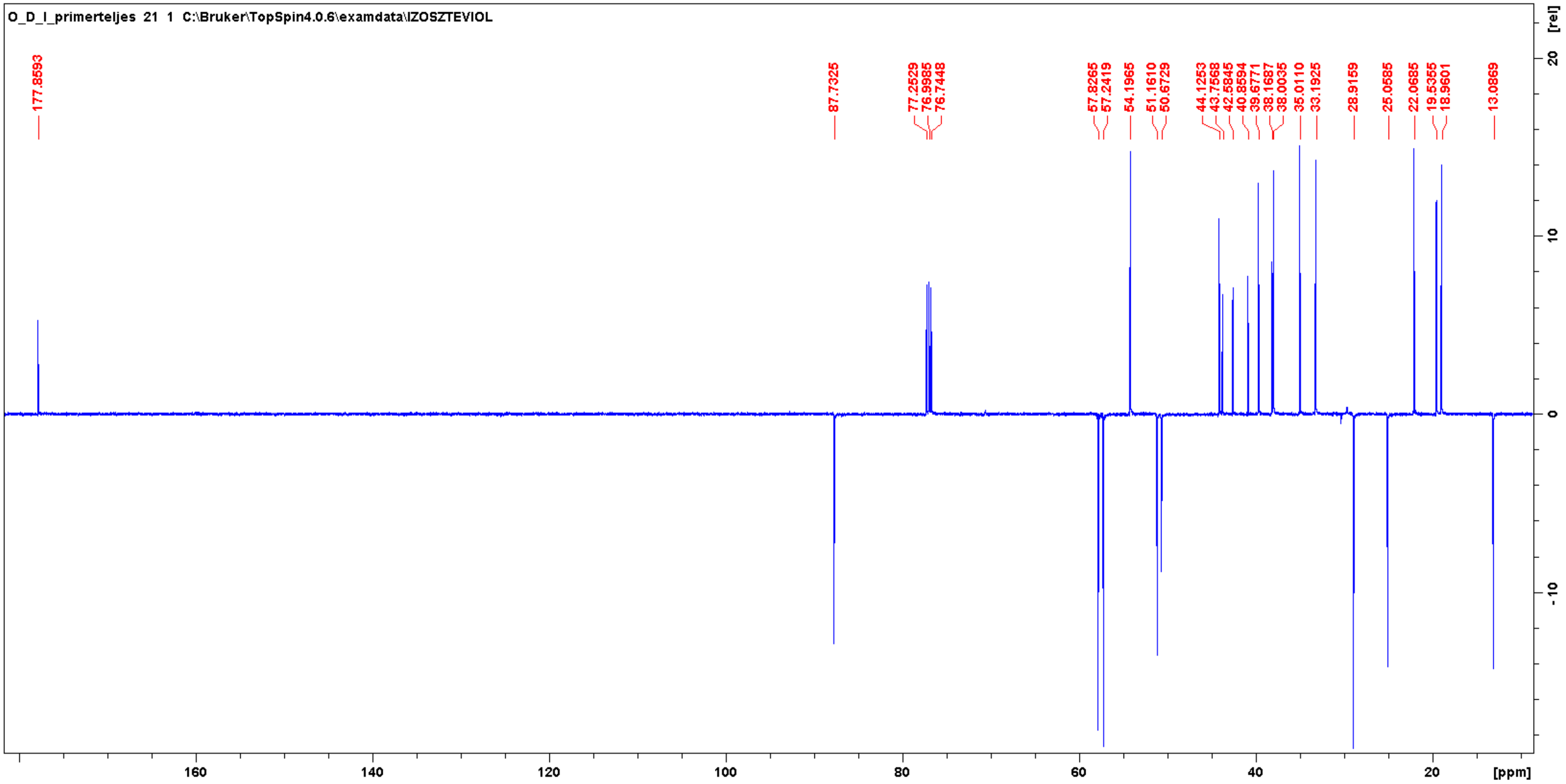

COSY of compound (4*R*,6a*S*,8*R*,9*S*,11b*S*)-Methyl 7-aminomethyl-8-hydroxy-4,9,11b-trimethyltetradecahydro-6a,9-methanocyclohepta[a]naphthalene-4-carboxylate (**6**)

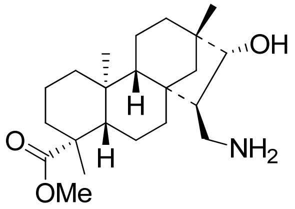

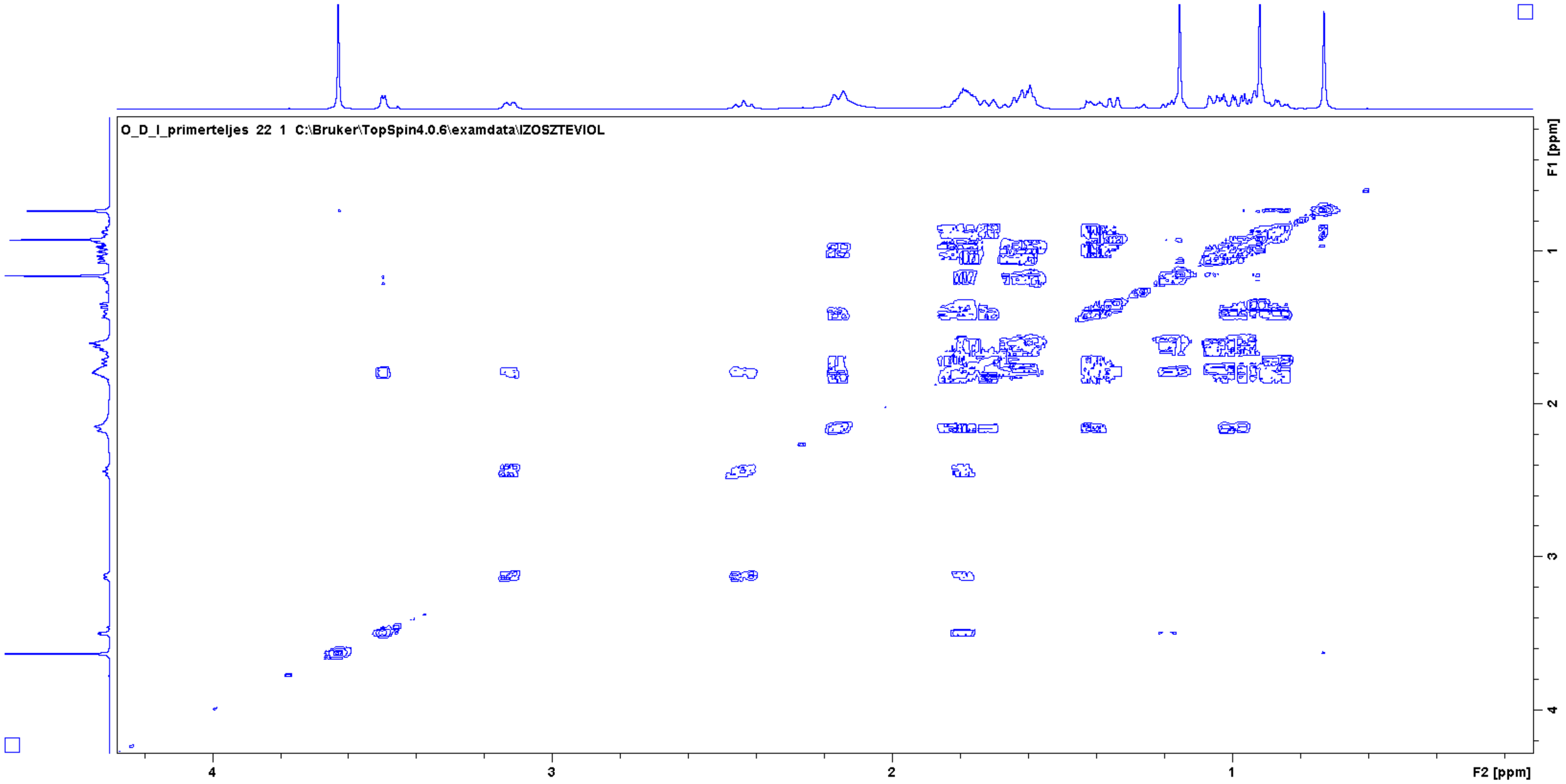

HSQC of compound (4*R*,6a*S*,8*R*,9*S*,11b*S*)-Methyl 7-aminomethyl-8-hydroxy-4,9,11b-trimethyltetradecahydro-6a,9-methanocyclohepta[a]naphthalene-4-carboxylate (**6**)

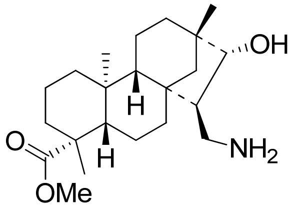

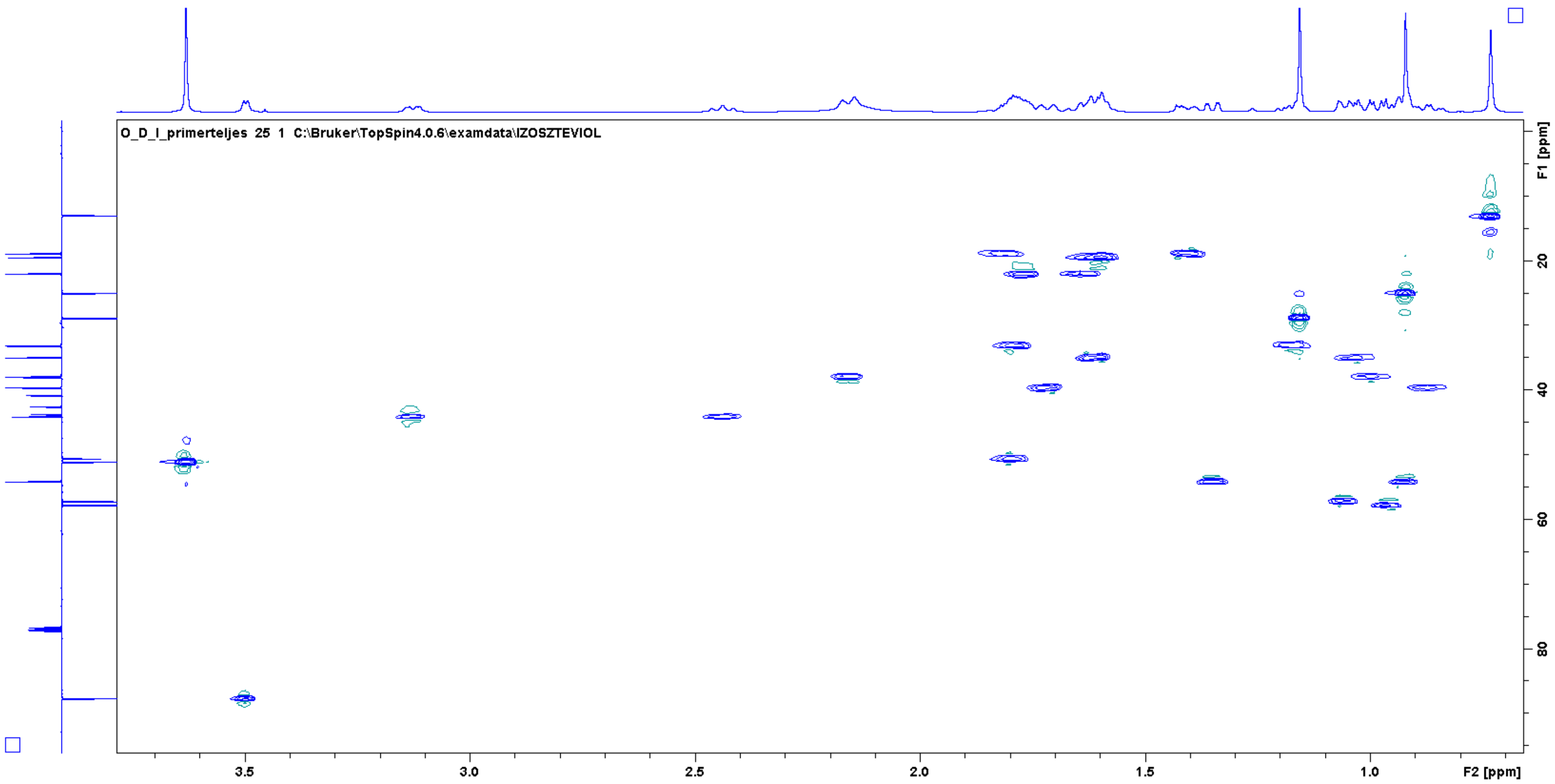

HMBC of compound (4*R*,6a*S*,8*R*,9*S*,11b*S*)-Methyl 7-aminomethyl-8-hydroxy-4,9,11b-trimethyltetradecahydro-6a,9-methanocyclohepta[a]naphthalene-4-carboxylate (**6**)

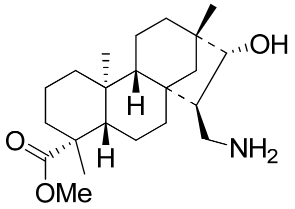

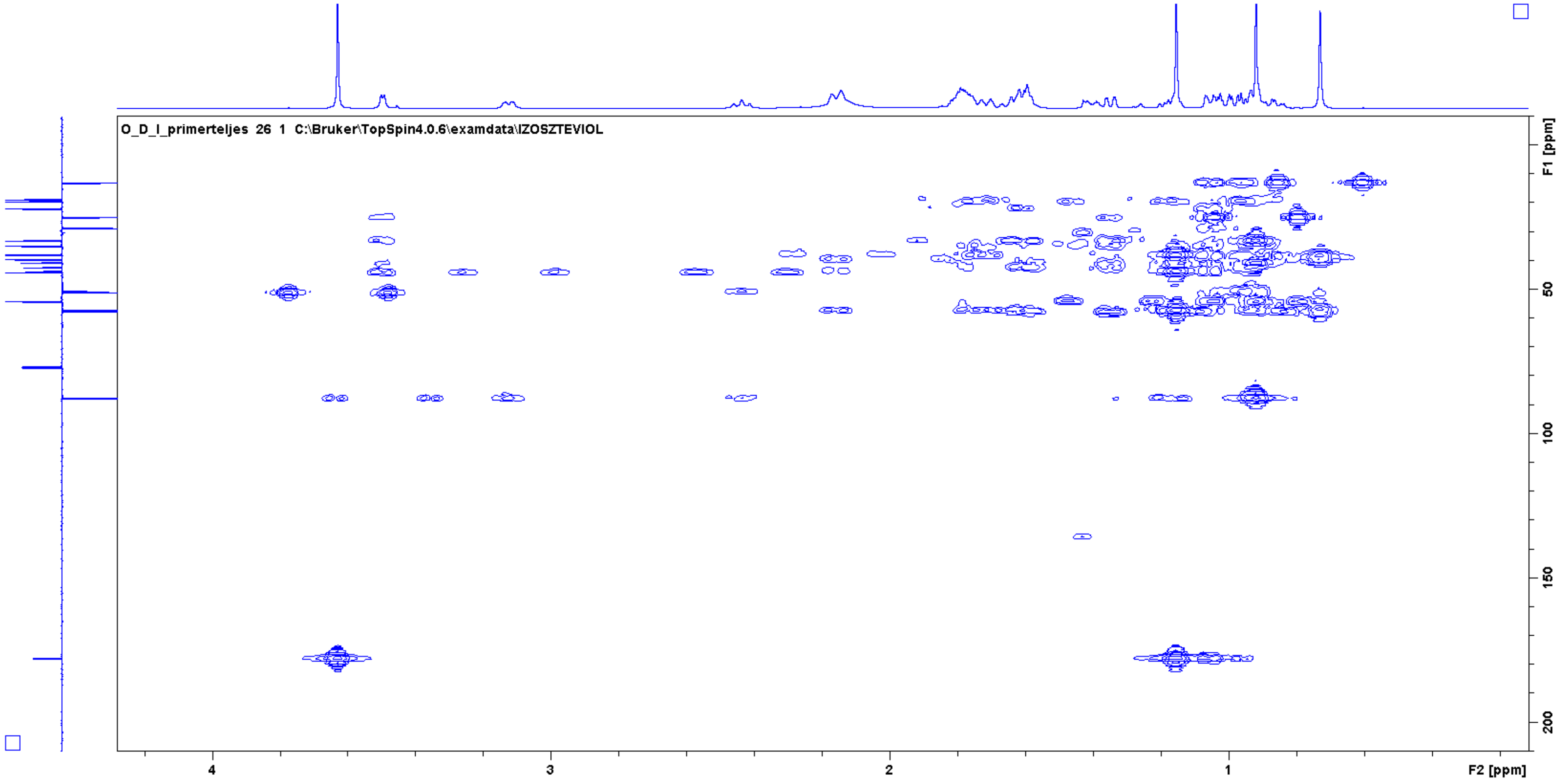

^{1}H-NMR of compound (4*R*,6a*S*,8*R*,9*S*,11b*S*)-Methyl 8-hydroxy-4,9,11b-trimethyl-7-((methylamino)methyl)tetradecahydro-6a,9-methanocyclohepta[a]naphthalene-4-carboxylate (**7**)

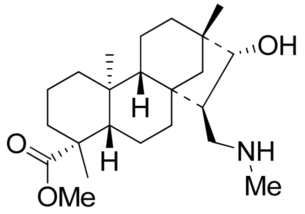

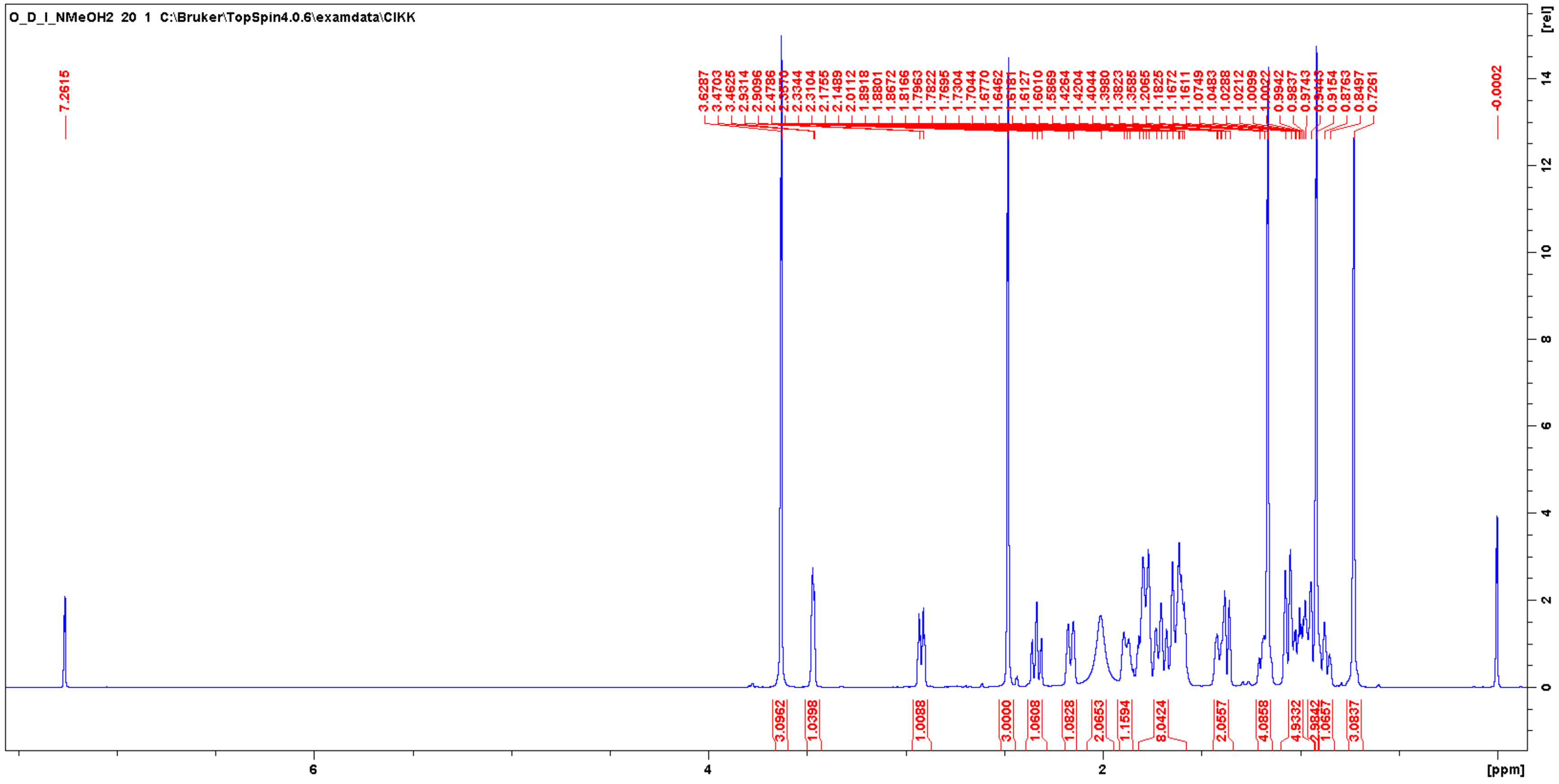

^{13}C-NMR of compound (4*R*,6a*S*,8*R*,9*S*,11b*S*)-Methyl 8-hydroxy-4,9,11b-trimethyl-7-((methylamino)methyl)tetradecahydro-6a,9-methanocyclohepta[a]naphthalene-4-carboxylate (**7**)

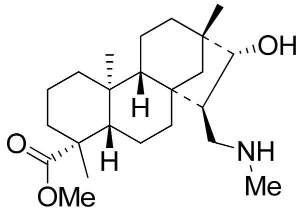

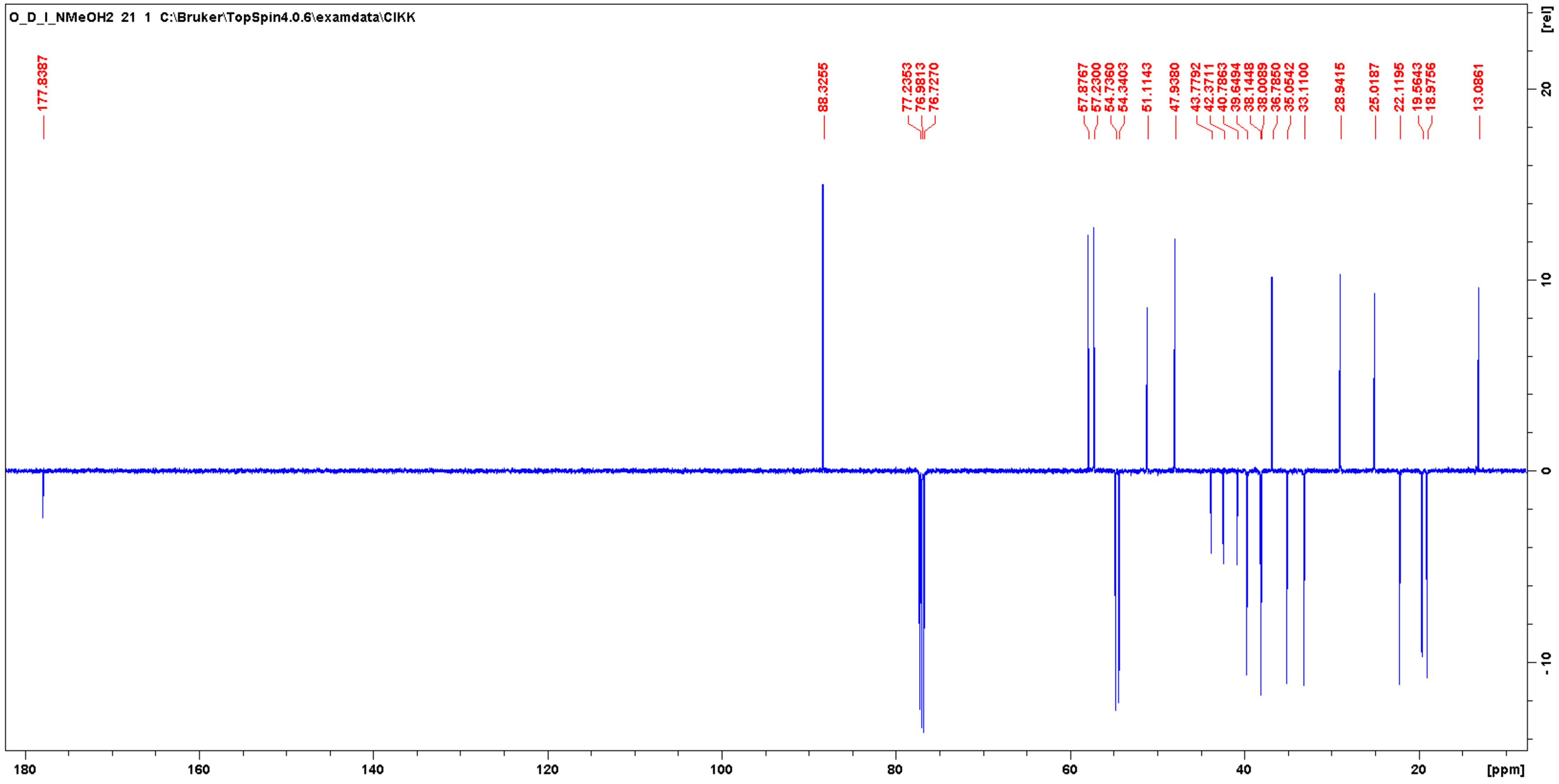

COSY of compound (4*R*,6a*S*,8*R*,9*S*,11b*S*)-Methyl 8-hydroxy-4,9,11b-trimethyl-7-((methylamino)methyl)tetradecahydro-6a,9-methanocyclohepta[a]naphthalene-4-carboxylate (**7**)

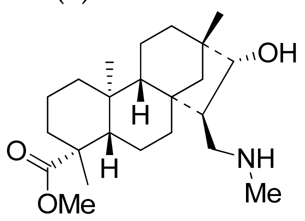

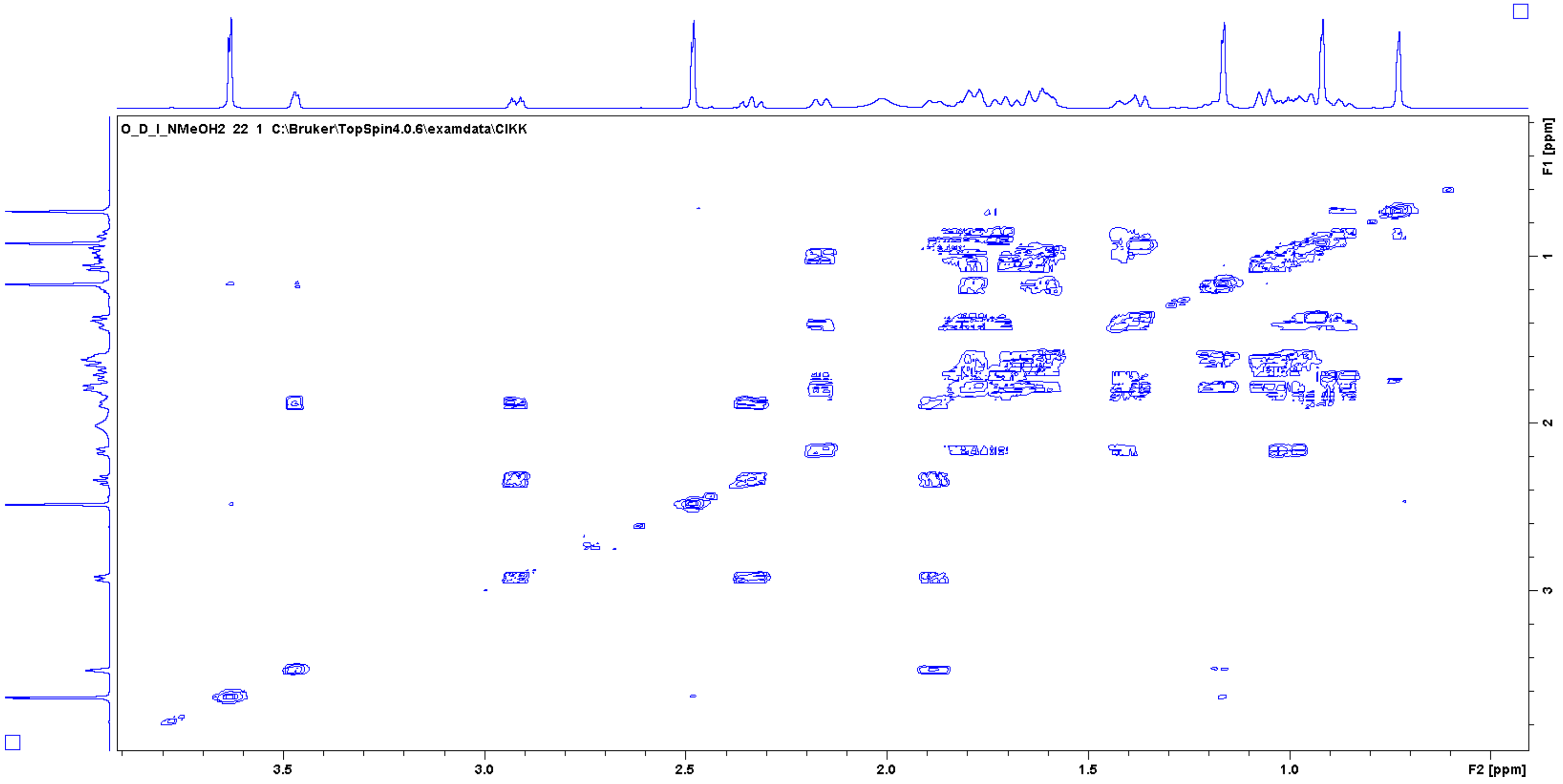

NOESY of compound (4*R*,6a*S*,8*R*,9*S*,11b*S*)-Methyl 8-hydroxy-4,9,11b-trimethyl-7-((methylamino)methyl)tetradecahydro-6a,9-methanocyclohepta[a]naphthalene-4-carboxylate (**7**)

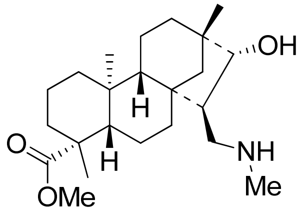

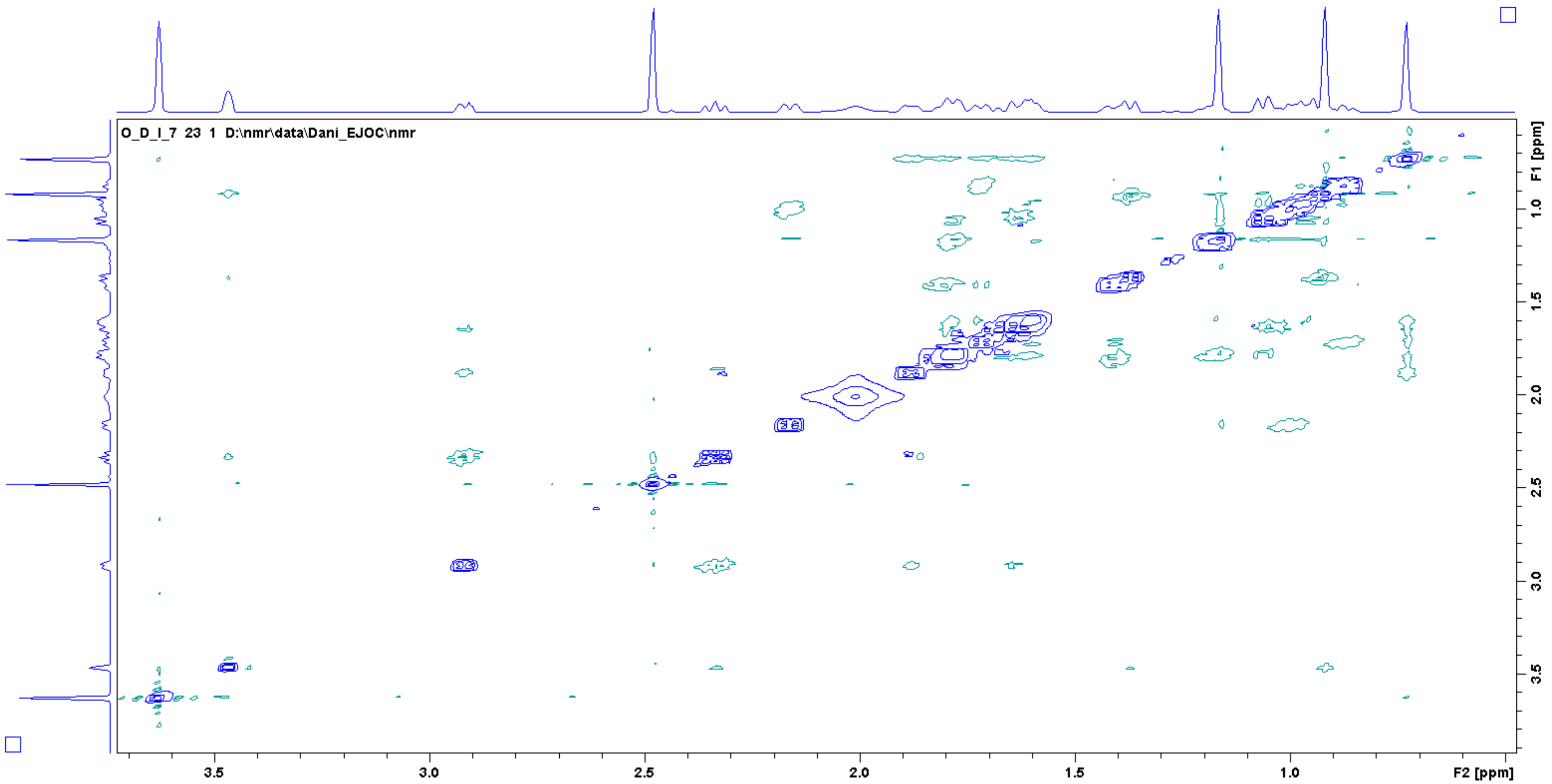

HSQC of compound (4*R*,6a*S*,8*R*,9*S*,11b*S*)-Methyl 8-hydroxy-4,9,11b-trimethyl-7-((methylamino)methyl)tetradecahydro-6a,9-methanocyclohepta[a]naphthalene-4-carboxylate (**7**)

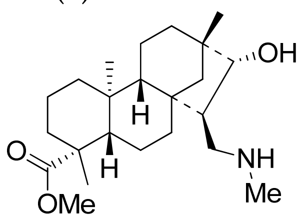

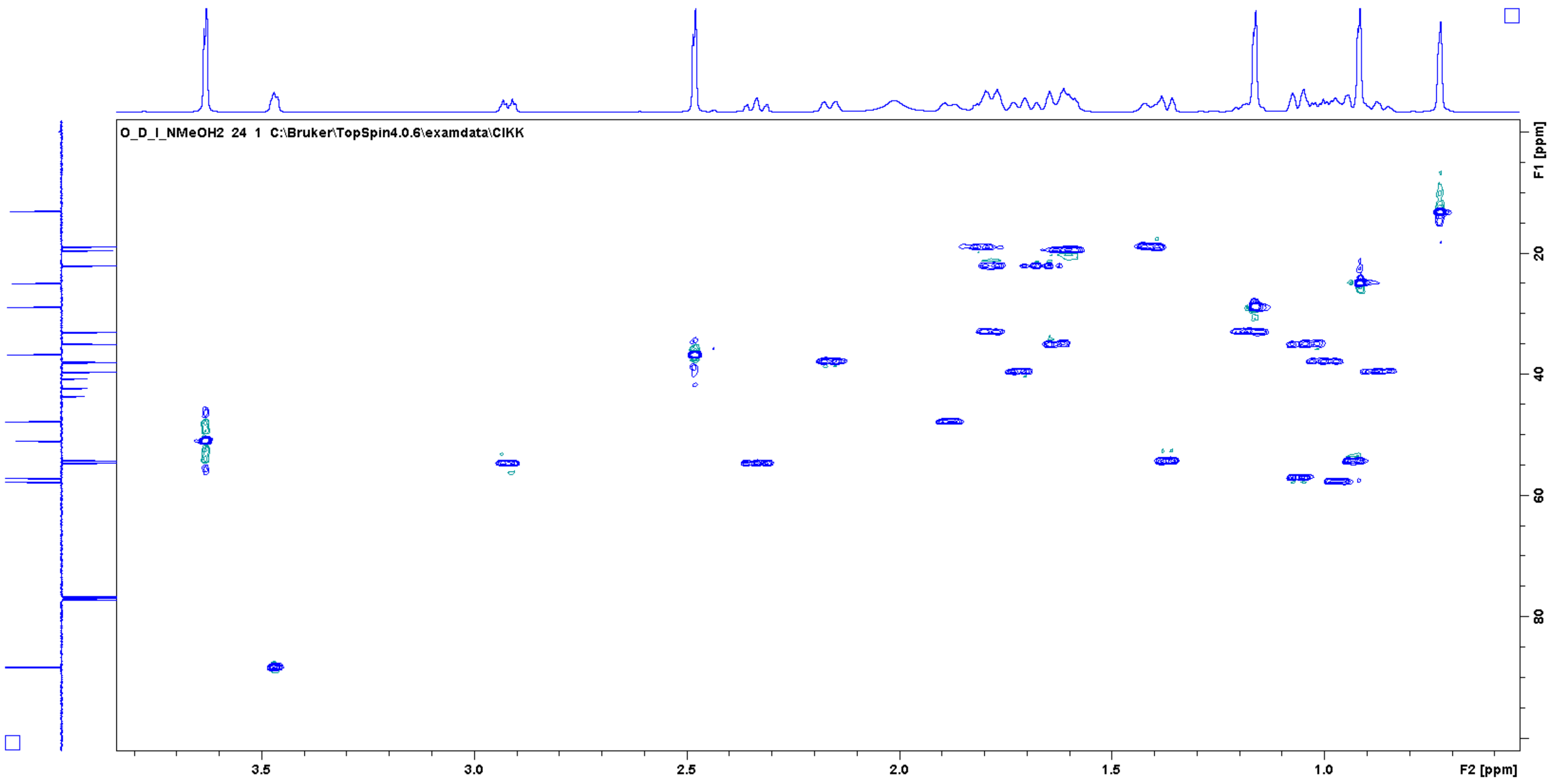

HMBC of compound (4*R*,6a*S*,8*R*,9*S*,11b*S*)-Methyl 8-hydroxy-4,9,11b-trimethyl-7-((methylamino)methyl)tetradecahydro-6a,9-methanocyclohepta[a]naphthalene-4-carboxylate (**7**)

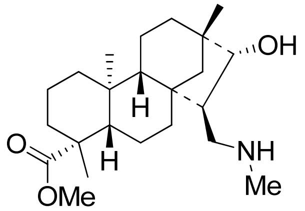

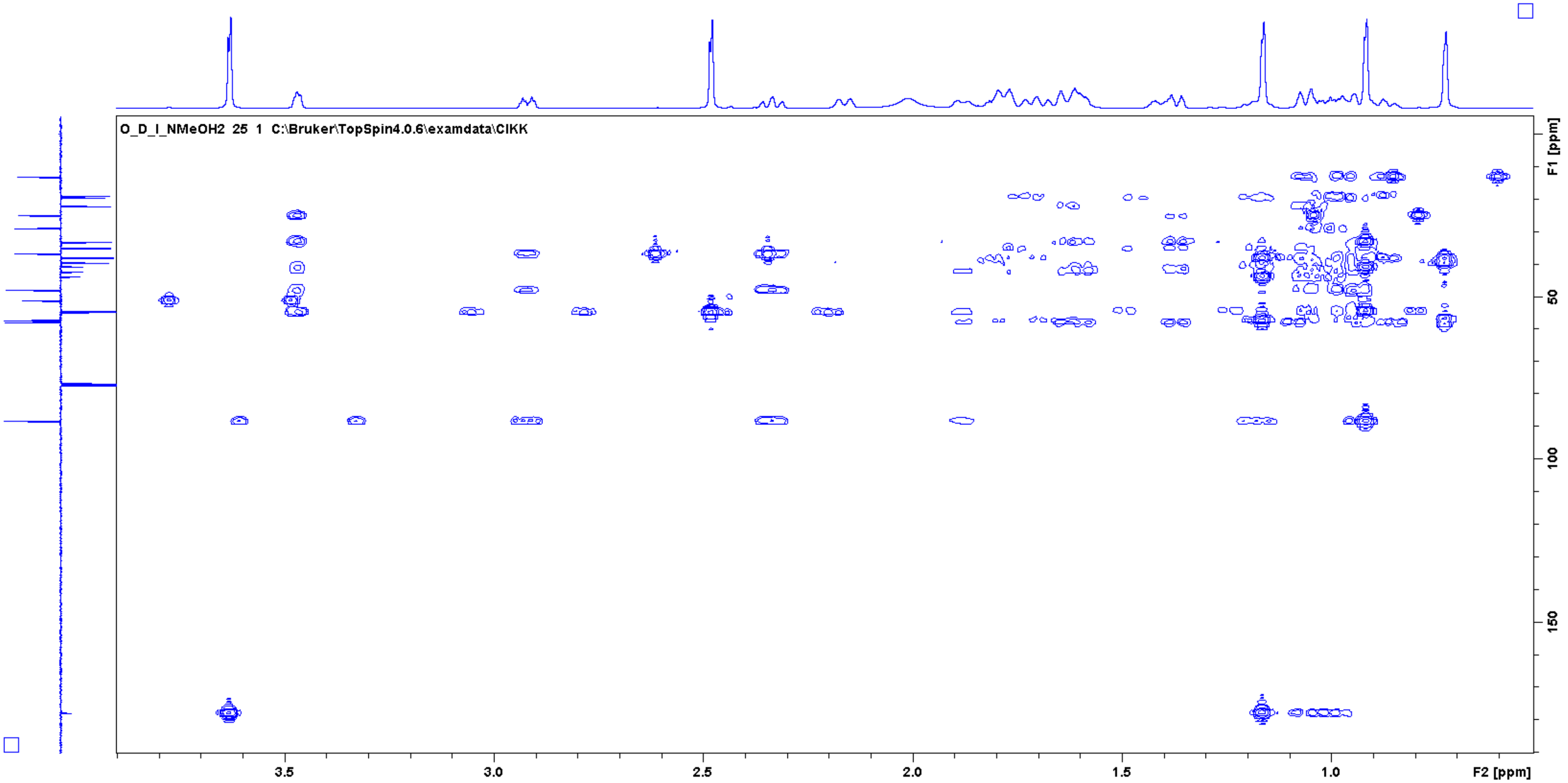

^{1}H-NMR of compound (4*R*,6a*S*,8*R*,9*S*,11b*S*)-Methyl 7-((benzylamino)methyl)-8-hydroxy-4,9,11b-trimethyltetradecahydro-6a,9-methanocyclohepta[a]naphthalene-4-carboxylate (**8**)

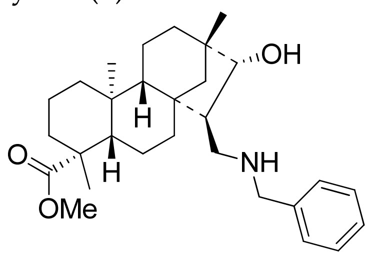

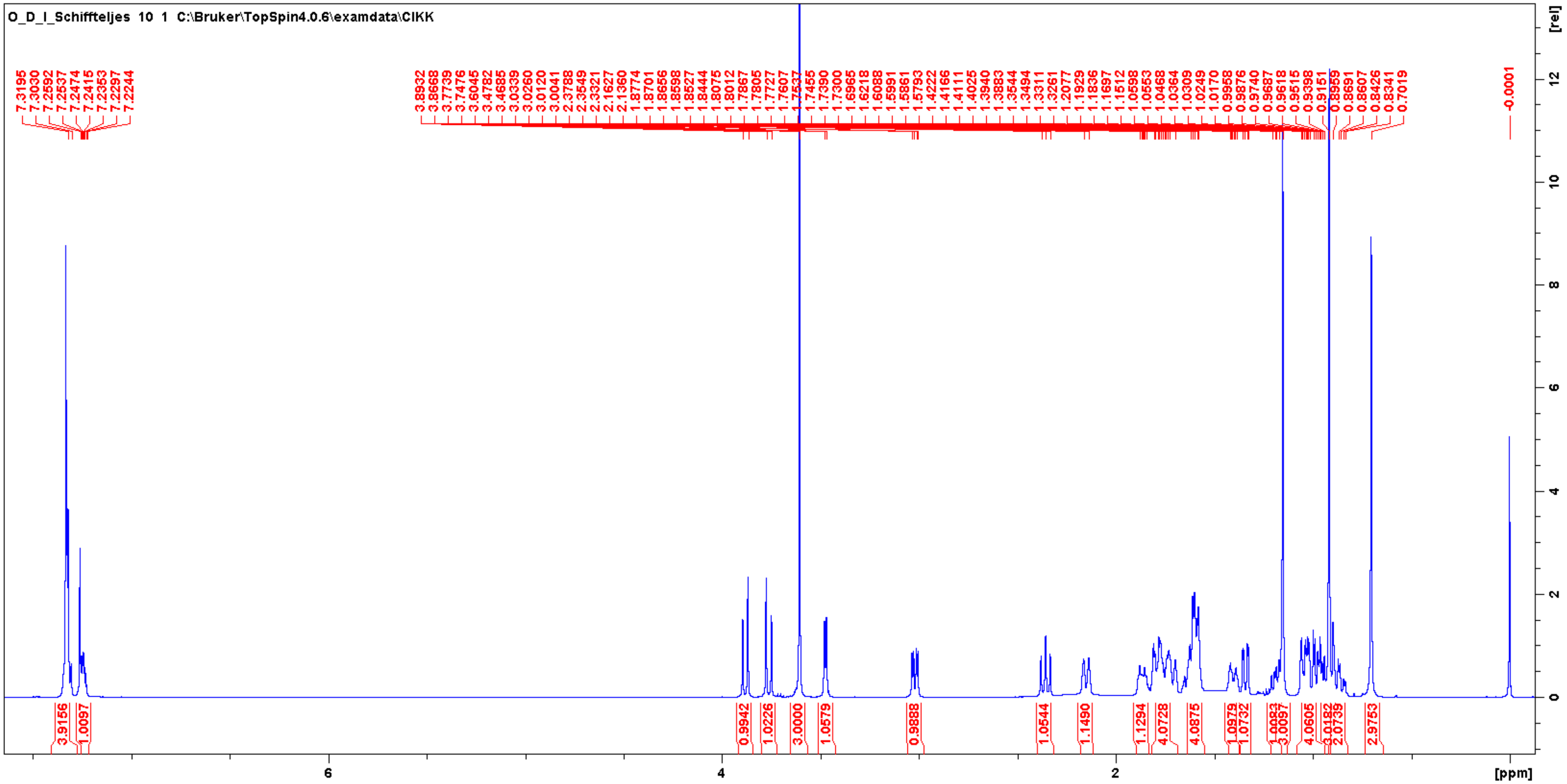

^{13}C-NMR of compound (4*R*,6a*S*,8*R*,9*S*,11b*S*)-Methyl 7-((benzylamino)methyl)-8-hydroxy-4,9,11b-trimethyltetradecahydro-6a,9-methanocyclohepta[a]naphthalene-4-carboxylate (**8**)

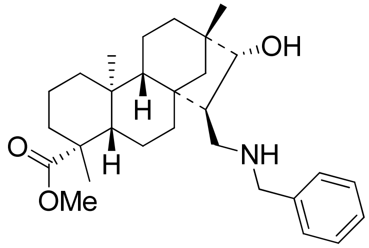

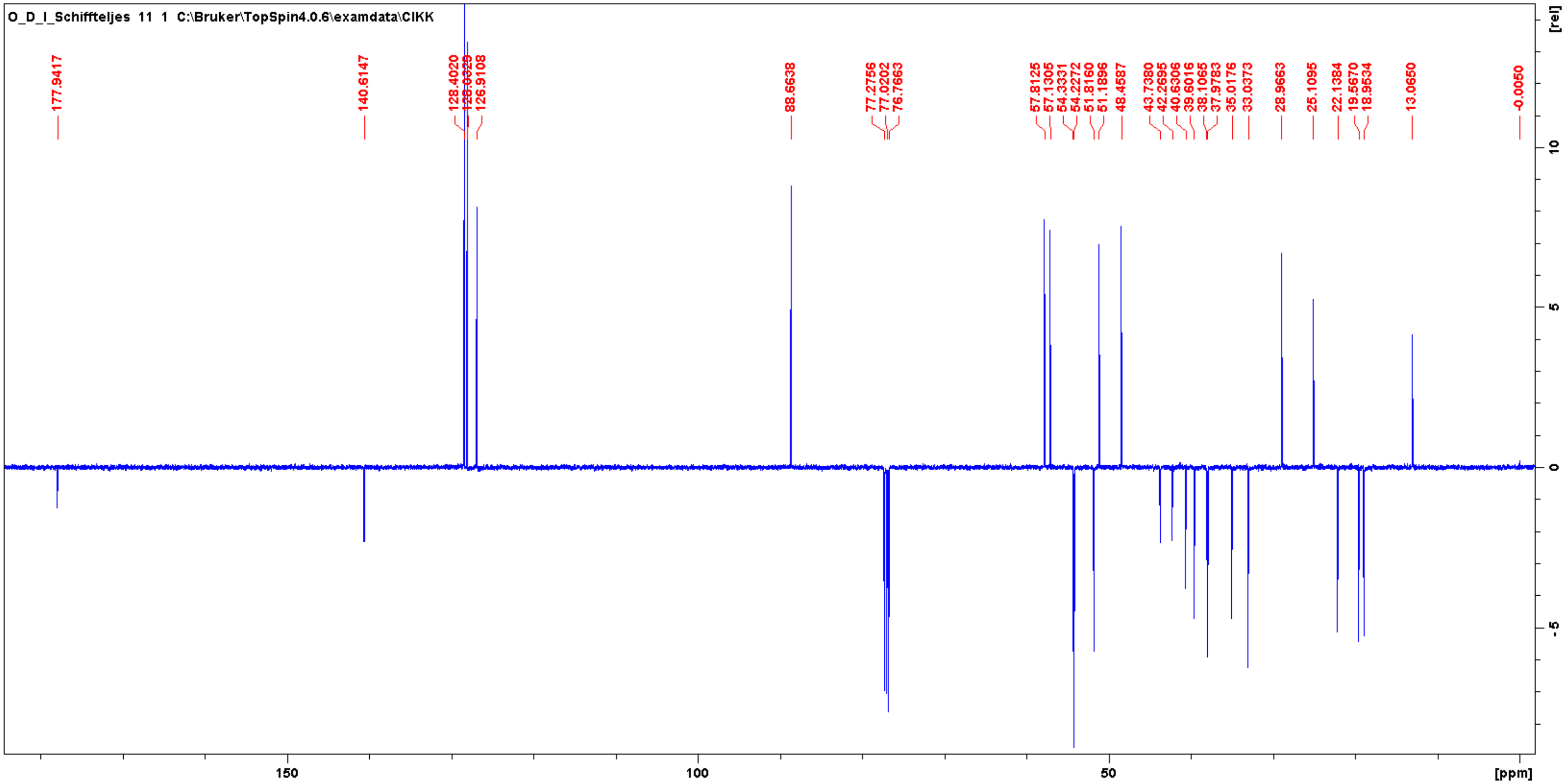

COSY of compound (4*R*,6a*S*,8*R*,9*S*,11b*S*)-Methyl 7-((benzylamino)methyl)-8-hydroxy-4,9,11b-trimethyltetradecahydro-6a,9-methanocyclohepta[a]naphthalene-4-carboxylate (**8**)

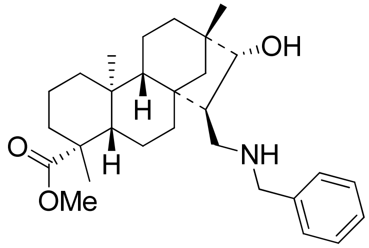

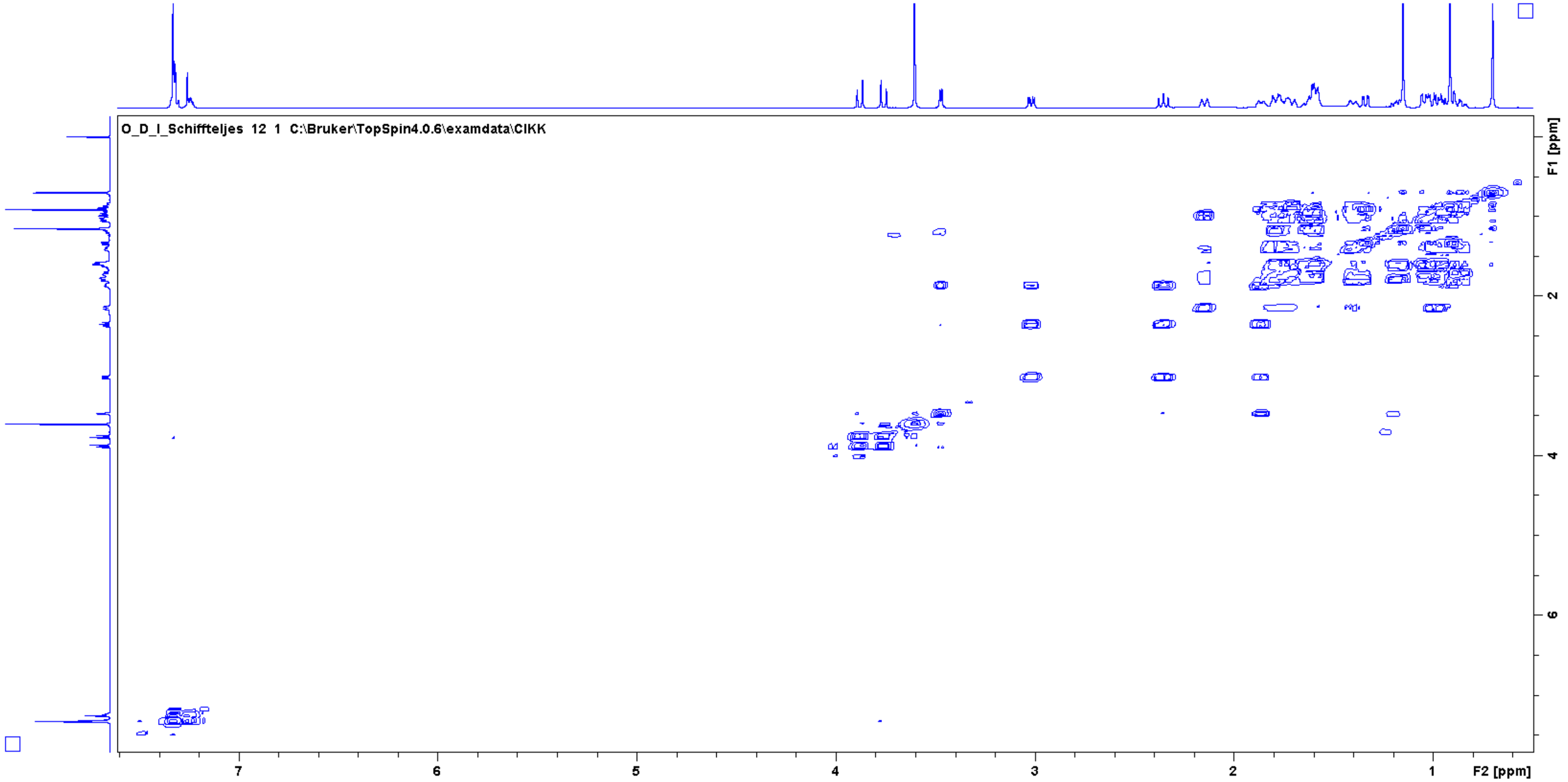

NOESY of compound (4*R*,6a*S*,8*R*,9*S*,11b*S*)-Methyl 7-((benzylamino)methyl)-8-hydroxy-4,9,11b-trimethyltetradecahydro-6a,9-methanocyclohepta[a]naphthalene-4-carboxylate (**8**)

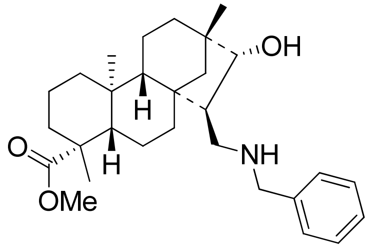

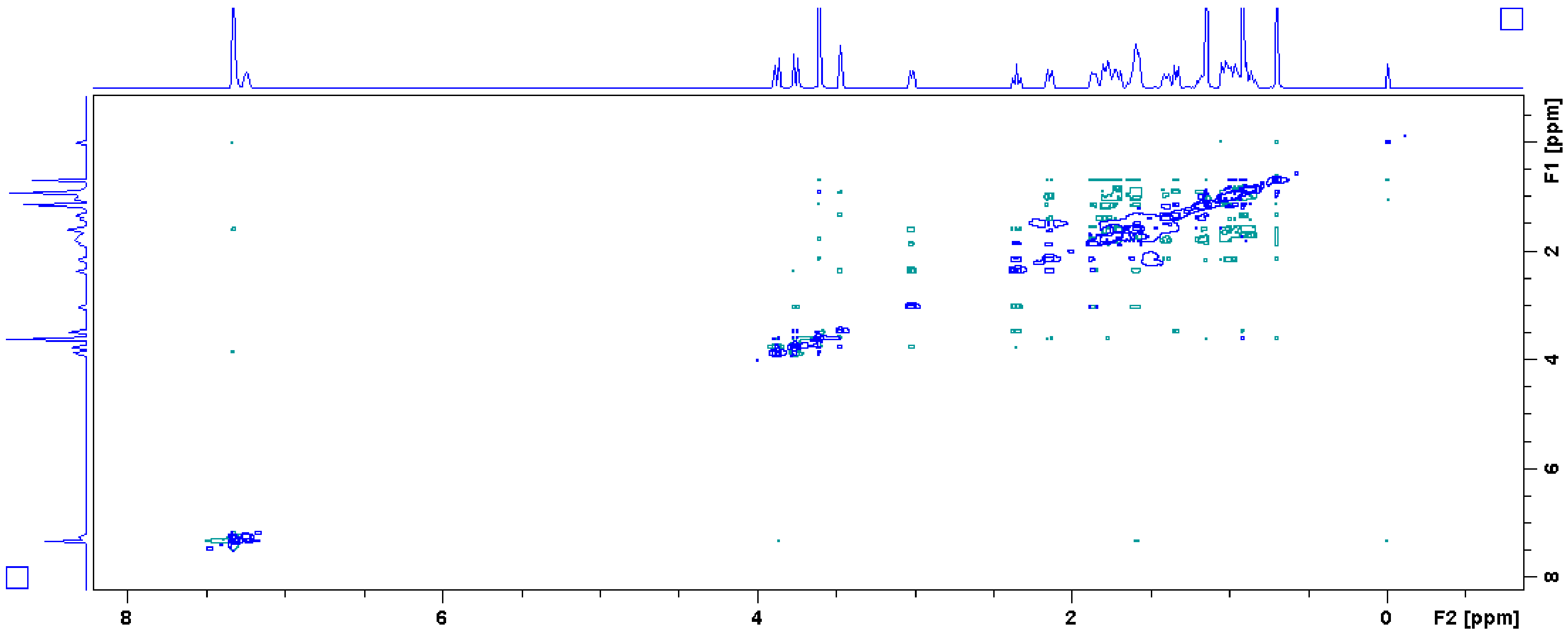

HSQC of compound (4*R*,6a*S*,8*R*,9*S*,11b*S*)-Methyl 7-((benzylamino)methyl)-8-hydroxy-4,9,11b-trimethyltetradecahydro-6a,9-methanocyclohepta[a]naphthalene-4-carboxylate (**8**)

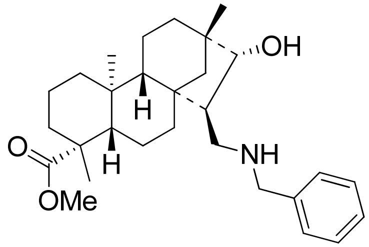

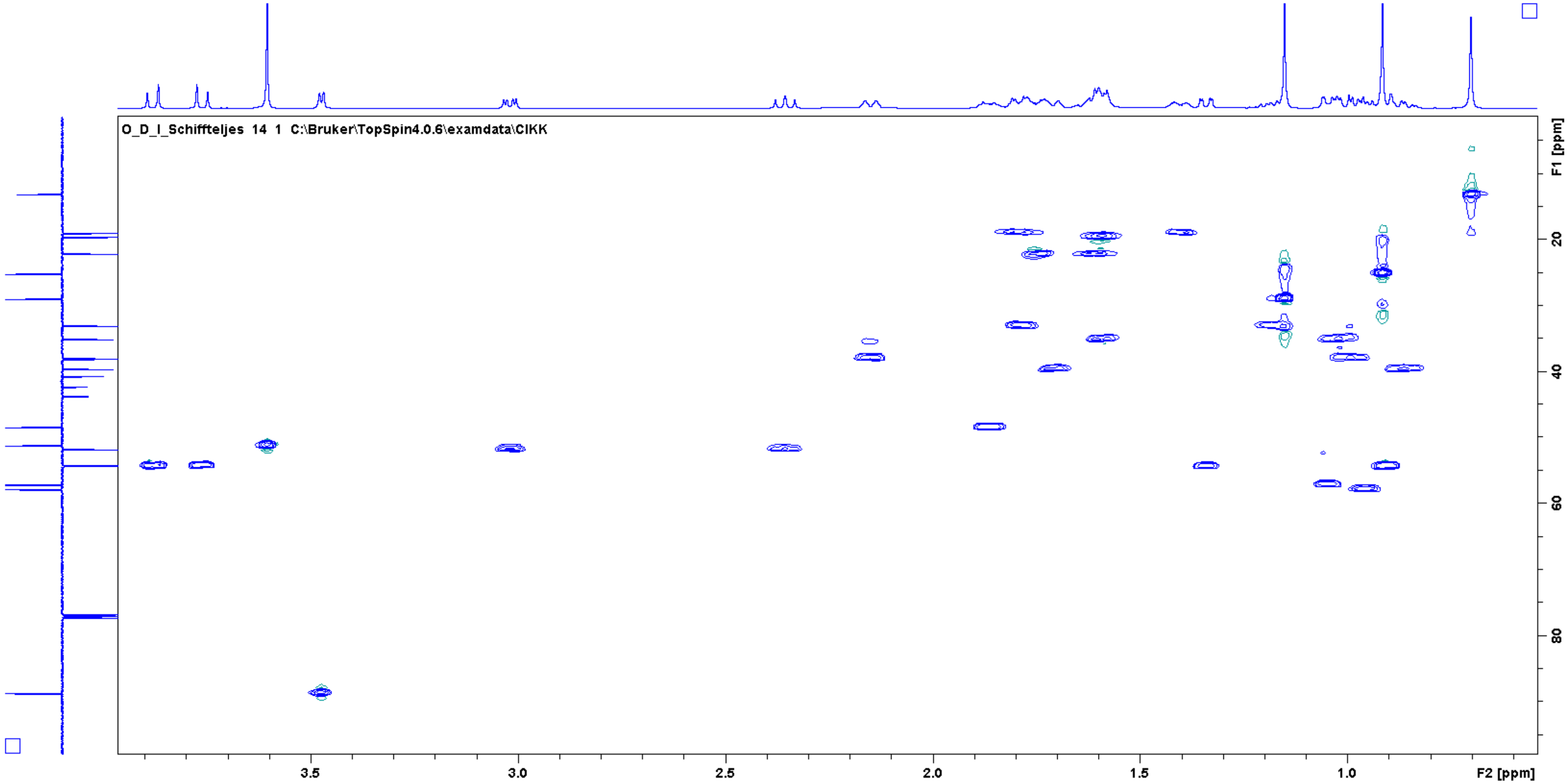

HMBC of compound (4*R*,6a*S*,8*R*,9*S*,11b*S*)-Methyl 7-((benzylamino)methyl)-8-hydroxy-4,9,11b-trimethyltetradecahydro-6a,9-methanocyclohepta[a]naphthalene-4-carboxylate (**8**)

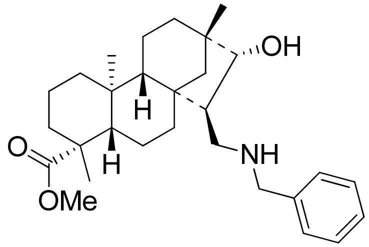

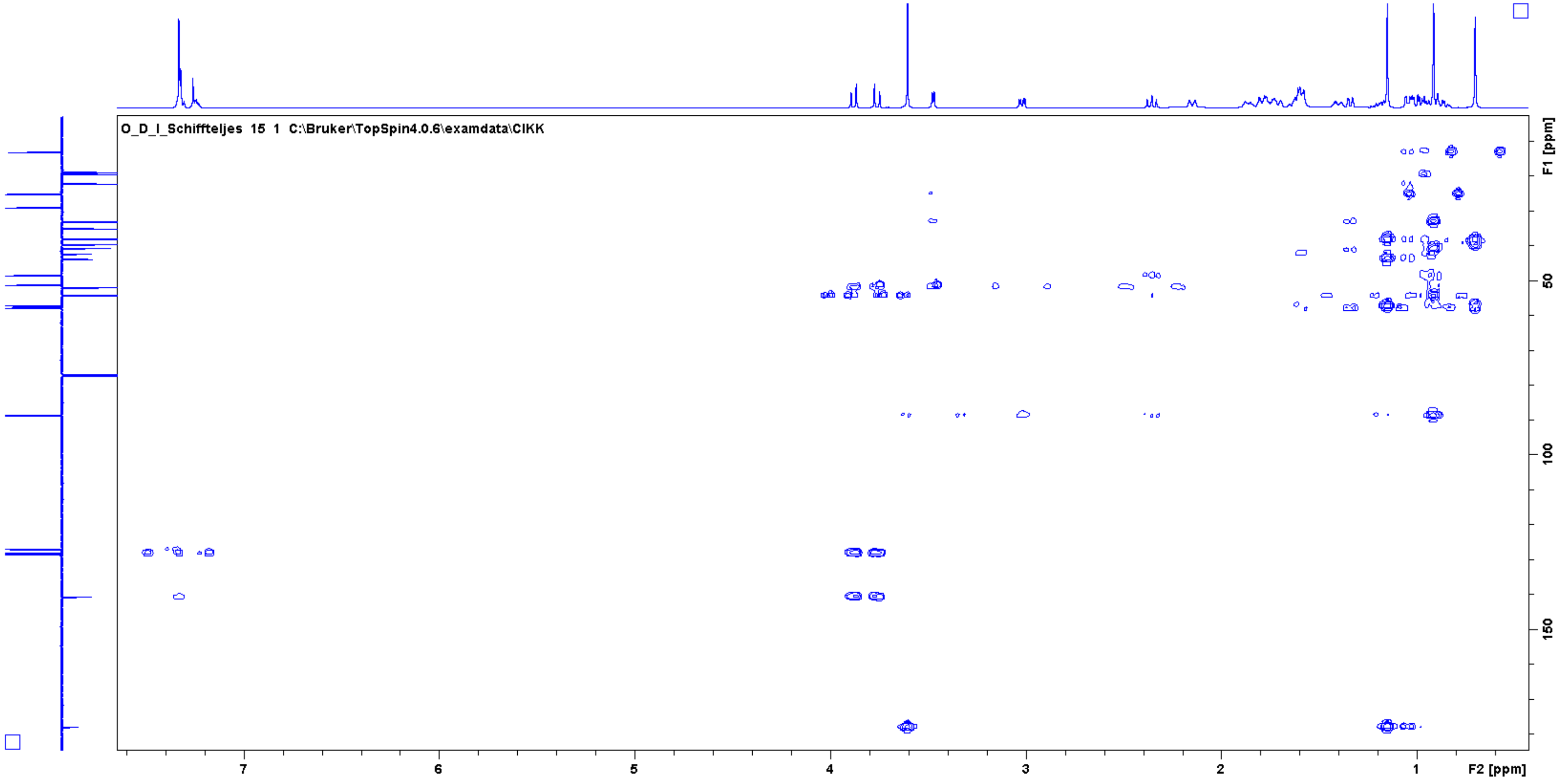

^{1}H-NMR of compound (4*R*,6a*S*,8*R*,9*S*,11b*S*)-Methyl 8-hydroxy-4,9,11b-trimethyl-7-((((*S*)-1-phenylethyl)amino)methyl)tetradecahydro-6a,9-methanocyclohepta[a]naphthalene-4-carboxylate (**9**)

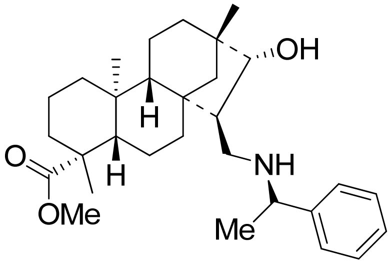

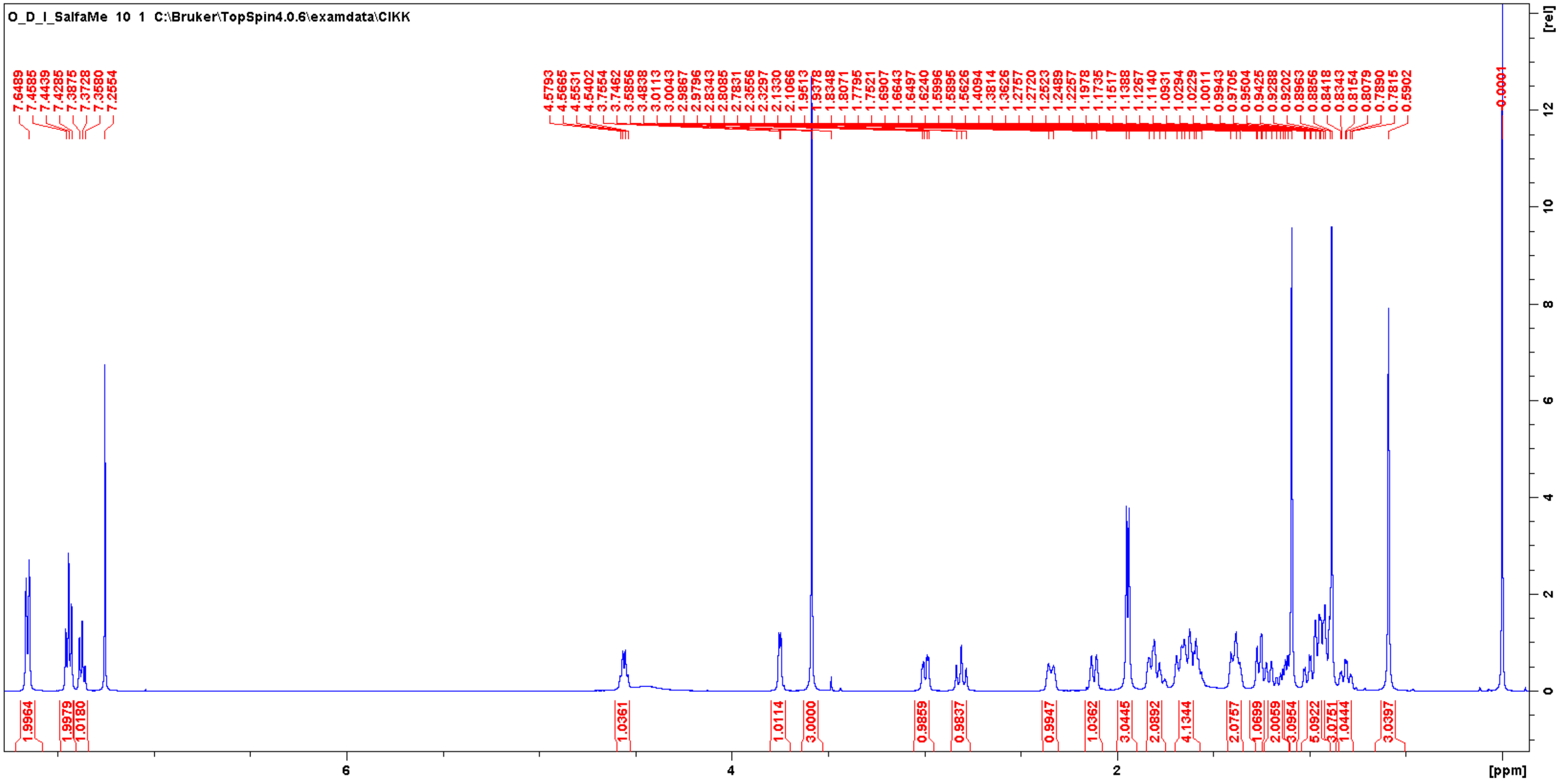

^{13}C-NMR of compound (4*R*,6a*S*,8*R*,9*S*,11b*S*)-Methyl 8-hydroxy-4,9,11b-trimethyl-7-((((*S*)-1-phenylethyl)amino)methyl)tetradecahydro-6a,9-methanocyclohepta[a]naphthalene-4-carboxylate (**9**)

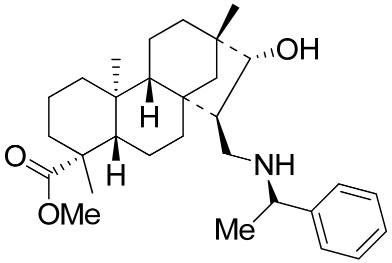

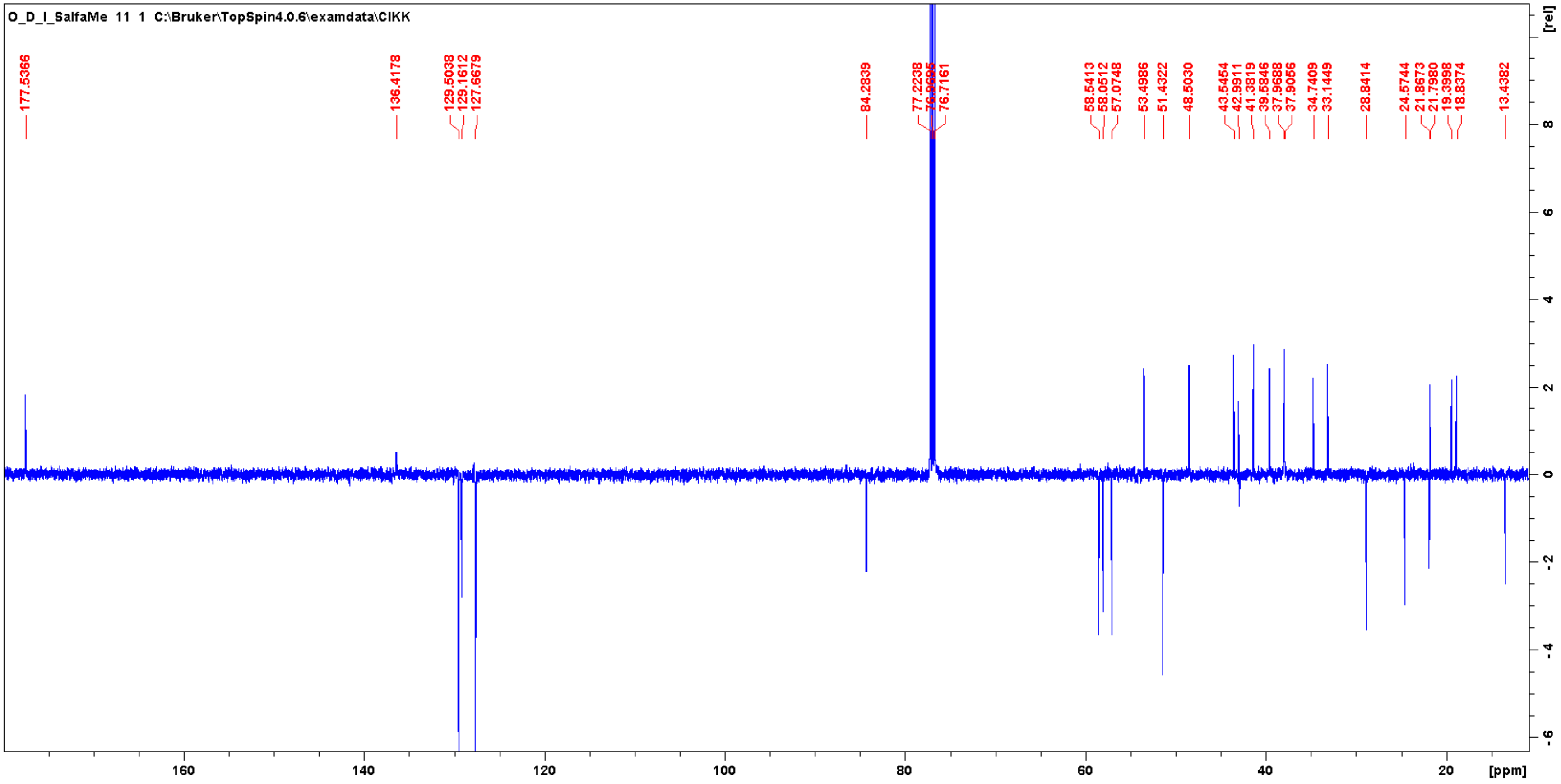

COSY of compound (4*R*,6a*S*,8*R*,9*S*,11b*S*)-Methyl 8-hydroxy-4,9,11b-trimethyl-7-((((*S*)-1-phenylethyl)amino)methyl)tetradecahydro-6a,9-methanocyclohepta[a]naphthalene-4-carboxylate (**9**)

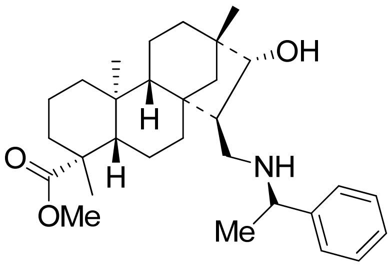

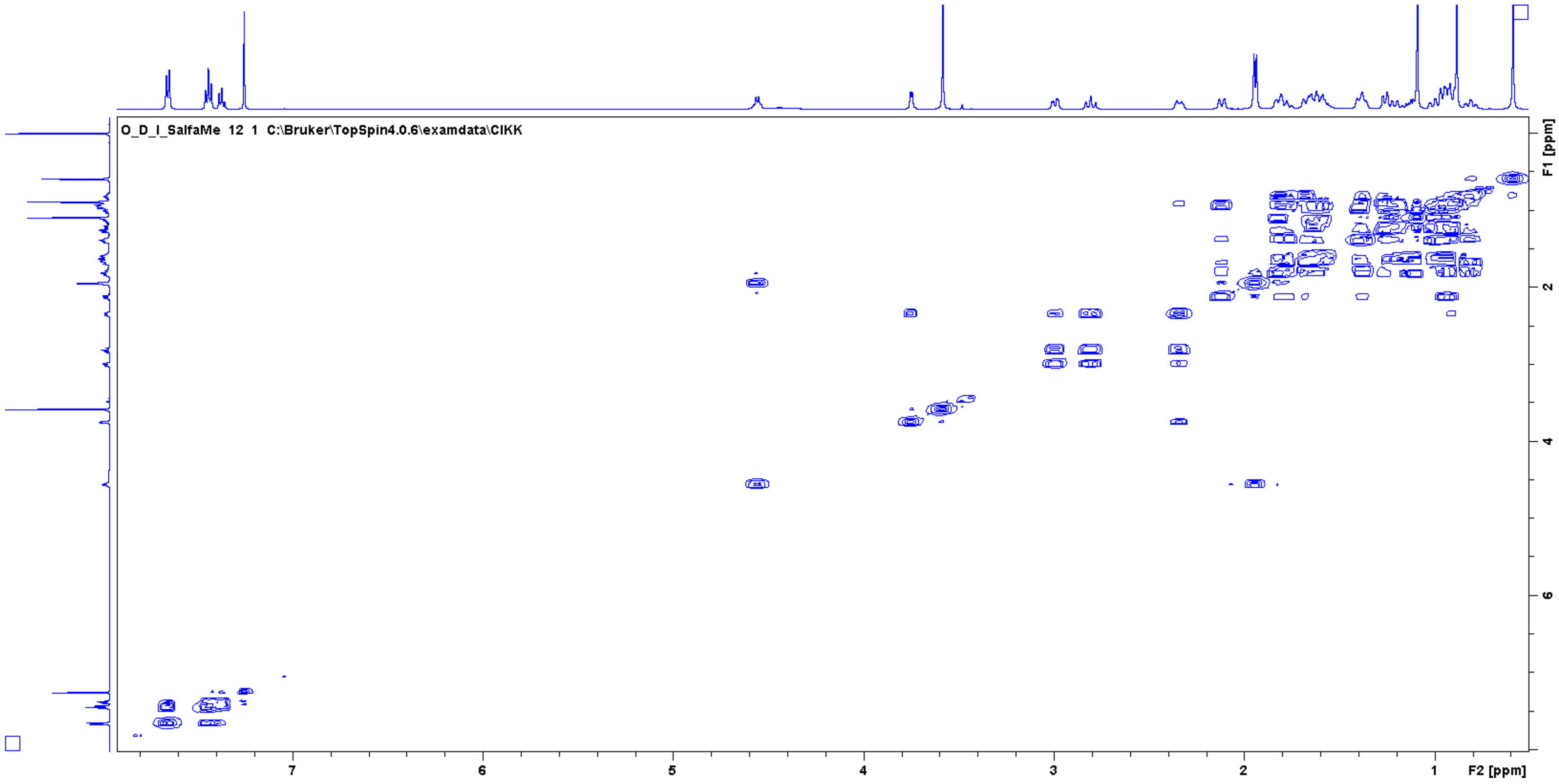

NOESY of compound (4*R*,6a*S*,8*R*,9*S*,11b*S*)-Methyl 8-hydroxy-4,9,11b-trimethyl-7-((((*S*)-1-phenylethyl)amino)methyl)tetradecahydro-6a,9-methanocyclohepta[a]naphthalene-4-carboxylate (**9**)

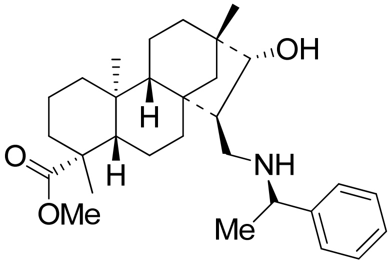

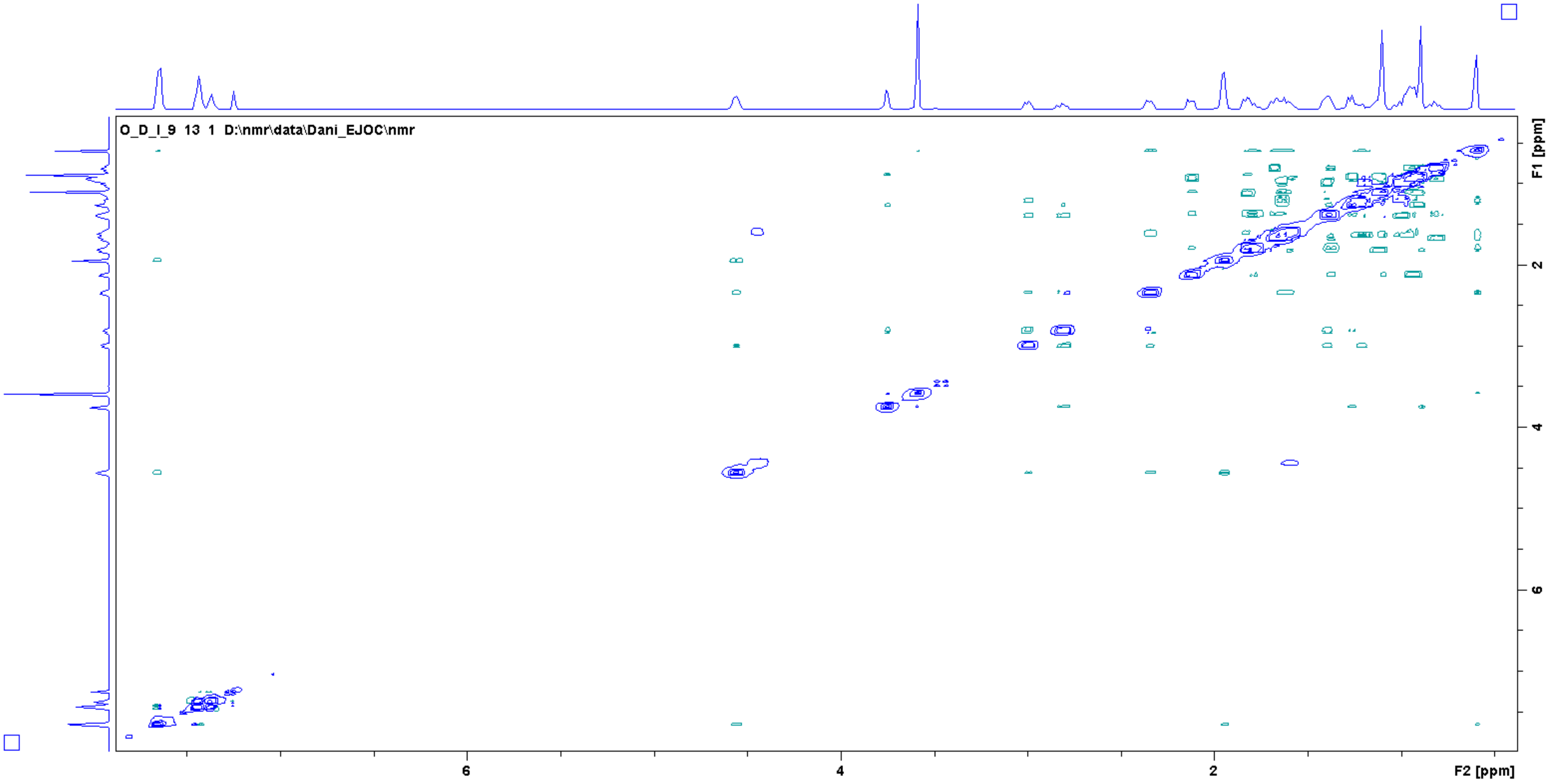

HSQC of compound (4*R*,6a*S*,8*R*,9*S*,11b*S*)-Methyl 8-hydroxy-4,9,11b-trimethyl-7-((((*S*)-1-phenylethyl)amino)methyl)tetradecahydro-6a,9-methanocyclohepta[a]naphthalene-4-carboxylate (**9**)

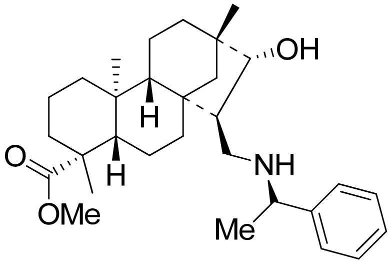

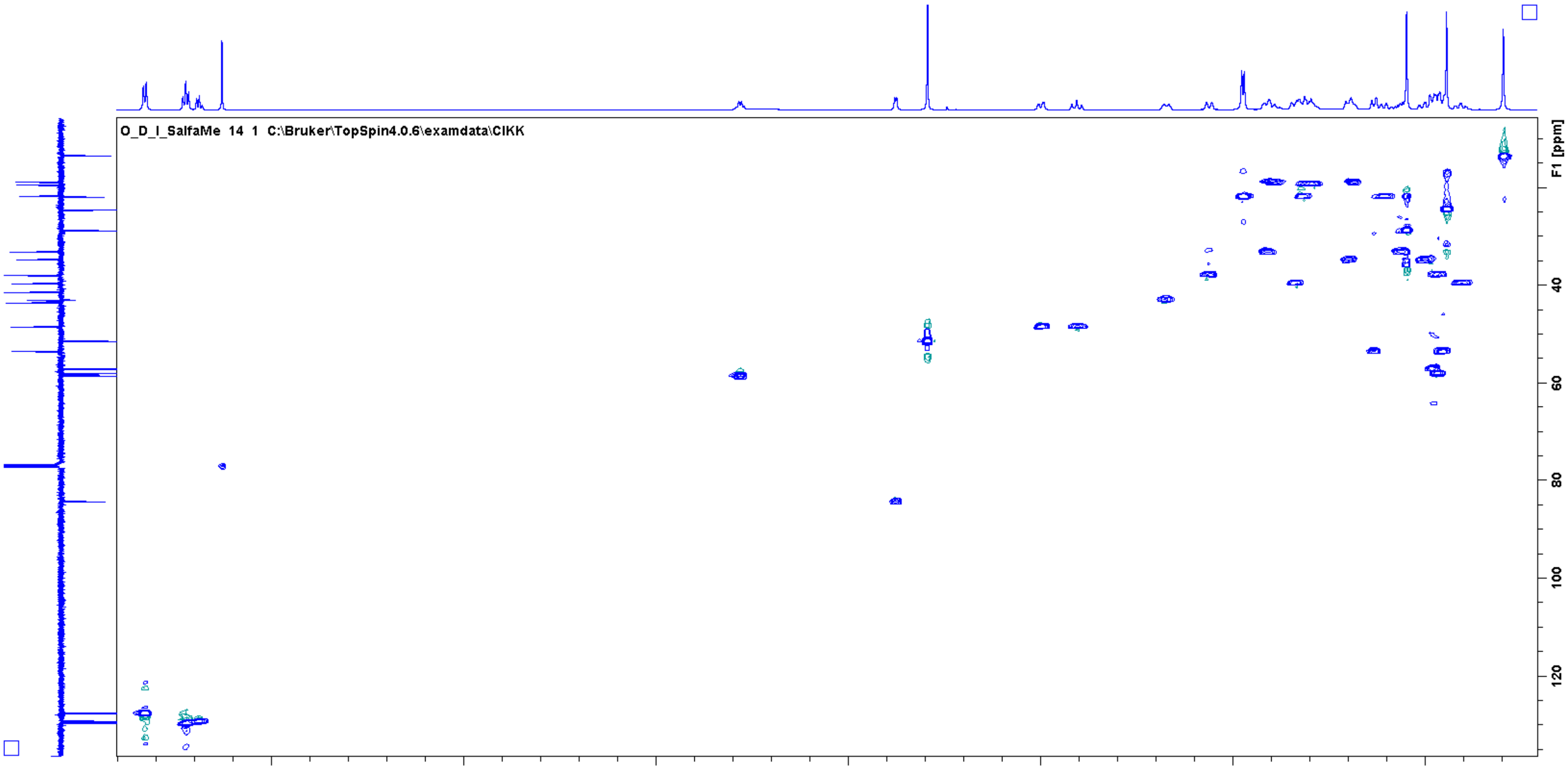

HMBC of compound (4*R*,6a*S*,8*R*,9*S*,11b*S*)-Methyl 8-hydroxy-4,9,11b-trimethyl-7-((((*S*)-1-phenylethyl)amino)methyl)tetradecahydro-6a,9-methanocyclohepta[a]naphthalene-4-carboxylate (**9**)

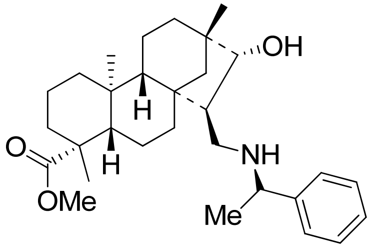

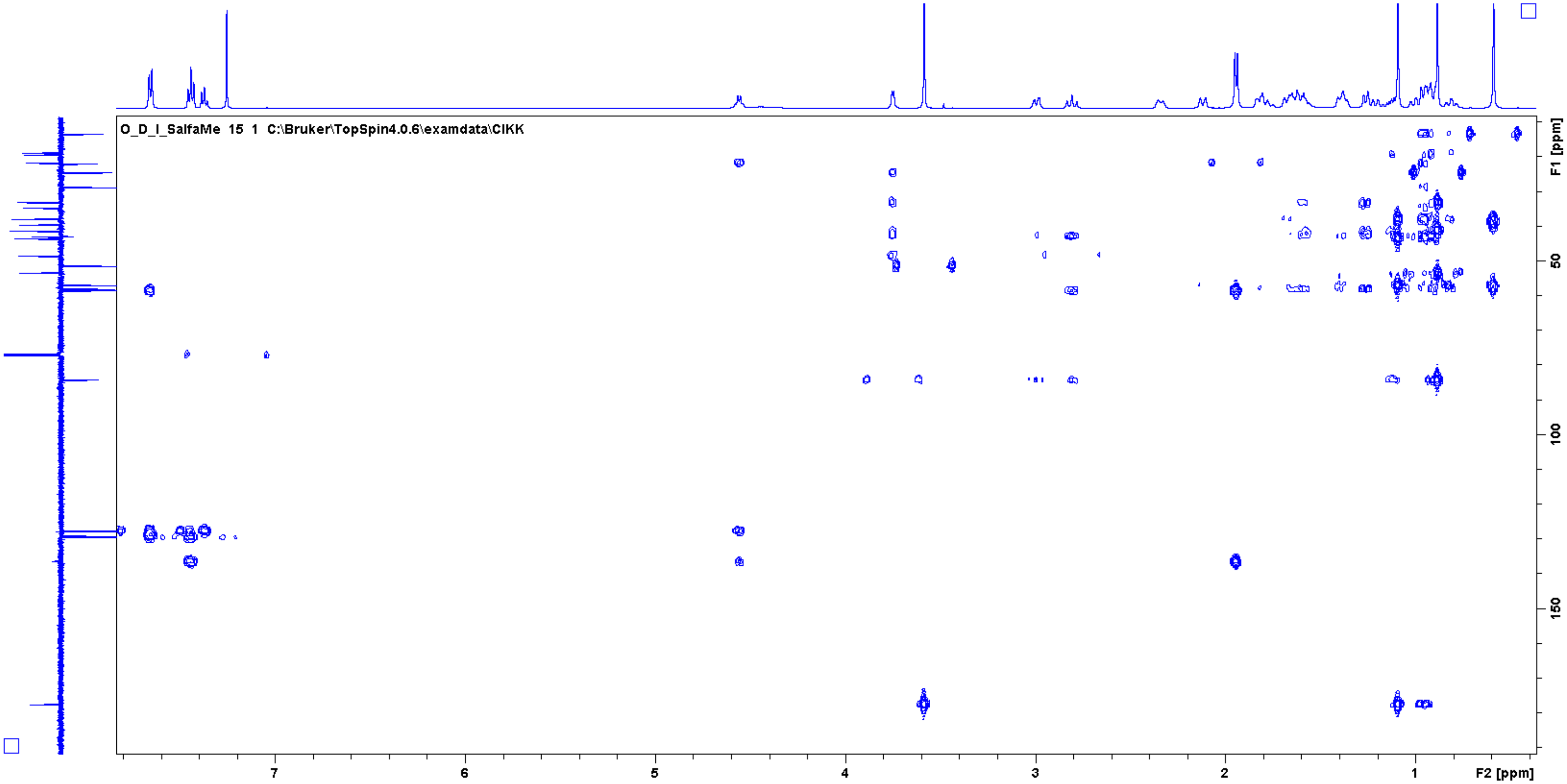

^{1}H-NMR of compound (4*R*,6a*S*,8*R*,9*S*,11b*S*)-Methyl 8-hydroxy-4,9,11b-trimethyl-7-((((*R*)-1-phenylethyl)amino)methyl)tetradecahydro-6a,9-methanocyclohepta[a]naphthalene-4-carboxylate (**10**)

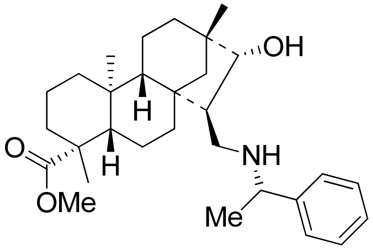

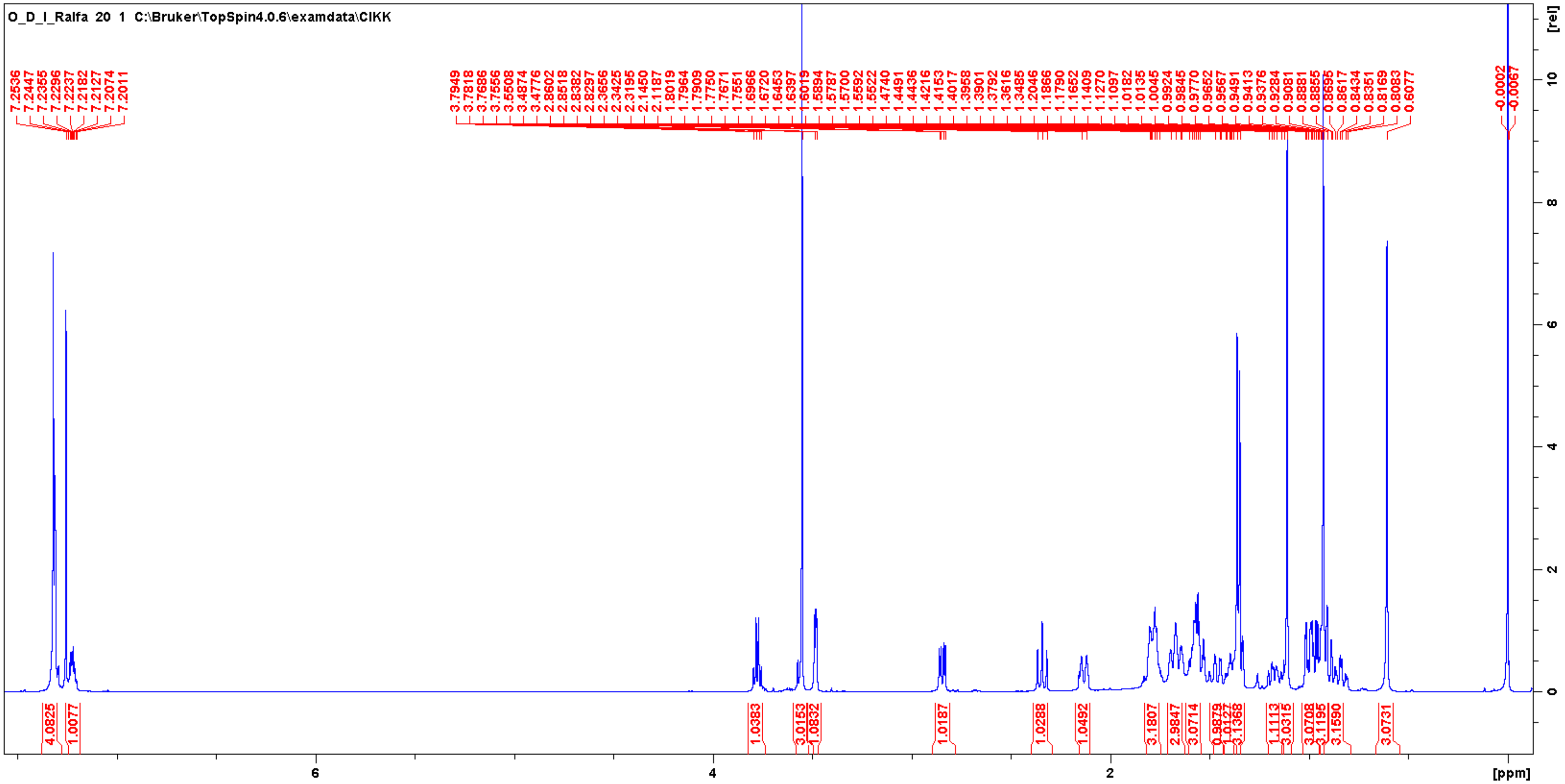

^{13}C-NMR of compound (4*R*,6a*S*,8*R*,9*S*,11b*S*)-Methyl 8-hydroxy-4,9,11b-trimethyl-7-((((*R*)-1-phenylethyl)amino)methyl)tetradecahydro-6a,9-methanocyclohepta[a]naphthalene-4-carboxylate (**10**)

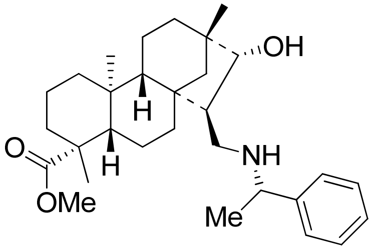

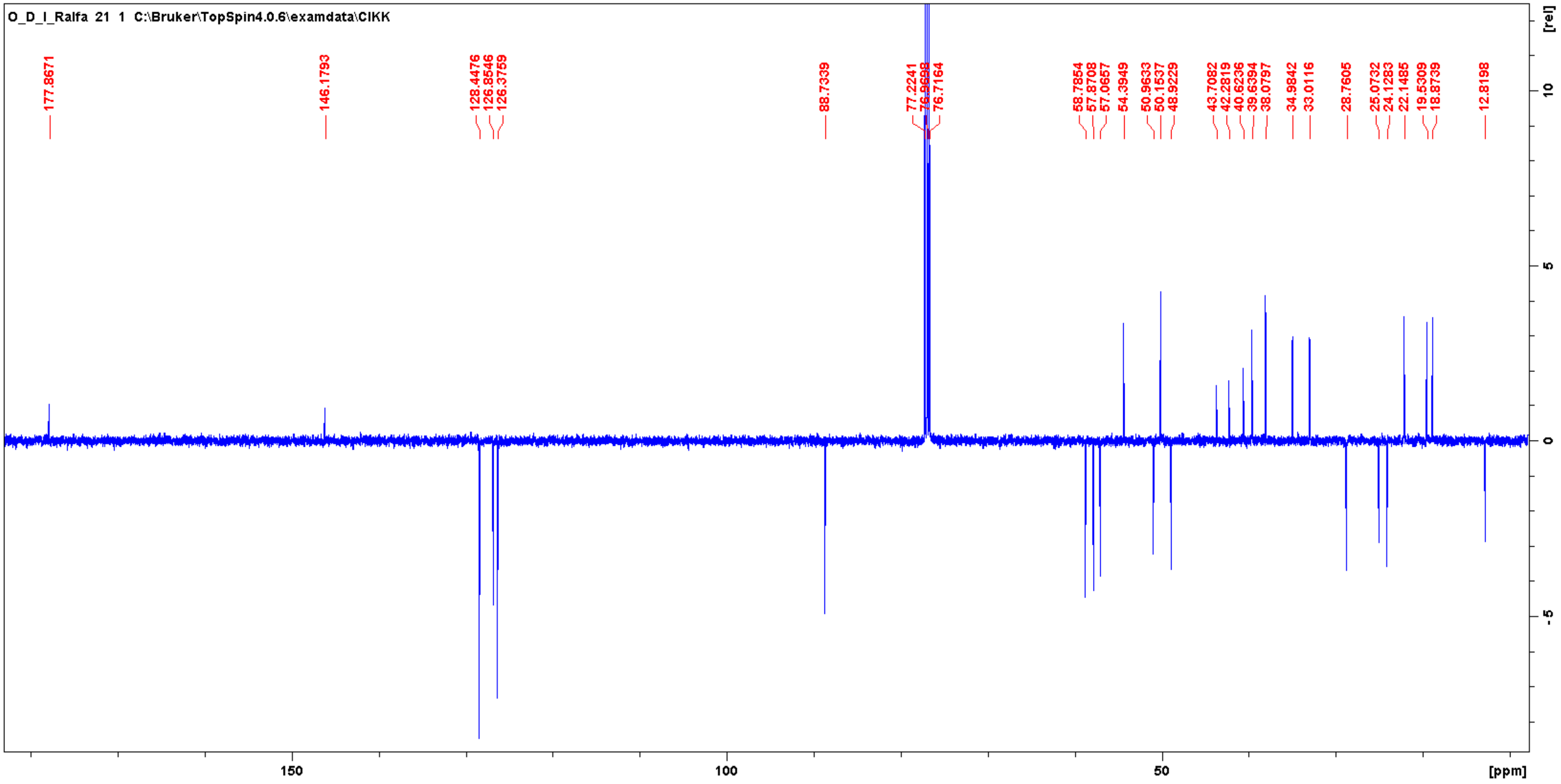

COSY of compound (4*R*,6a*S*,8*R*,9*S*,11b*S*)-Methyl 8-hydroxy-4,9,11b-trimethyl-7-((((*R*)-1-phenylethyl)amino)methyl)tetradecahydro-6a,9-methanocyclohepta[a]naphthalene-4-carboxylate (**10**)

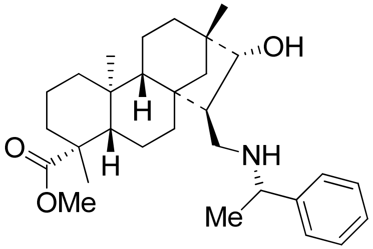

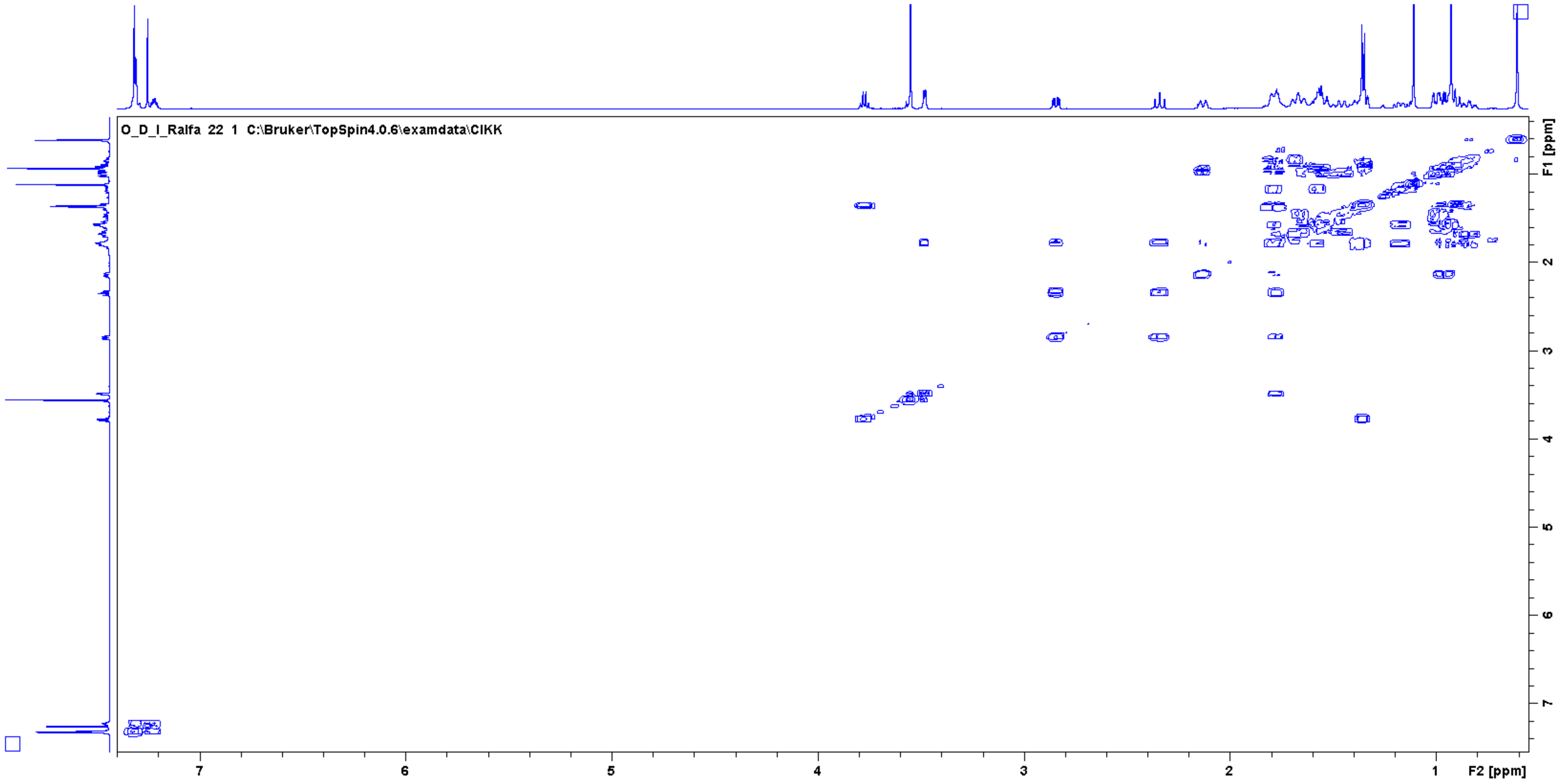

NOESY of compound (4*R*,6a*S*,8*R*,9*S*,11b*S*)-Methyl 8-hydroxy-4,9,11b-trimethyl-7-((((*R*)-1-phenylethyl)amino)methyl)tetradecahydro-6a,9-methanocyclohepta[a]naphthalene-4-carboxylate (**10**)

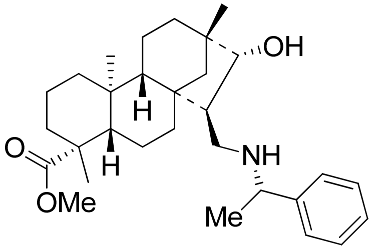

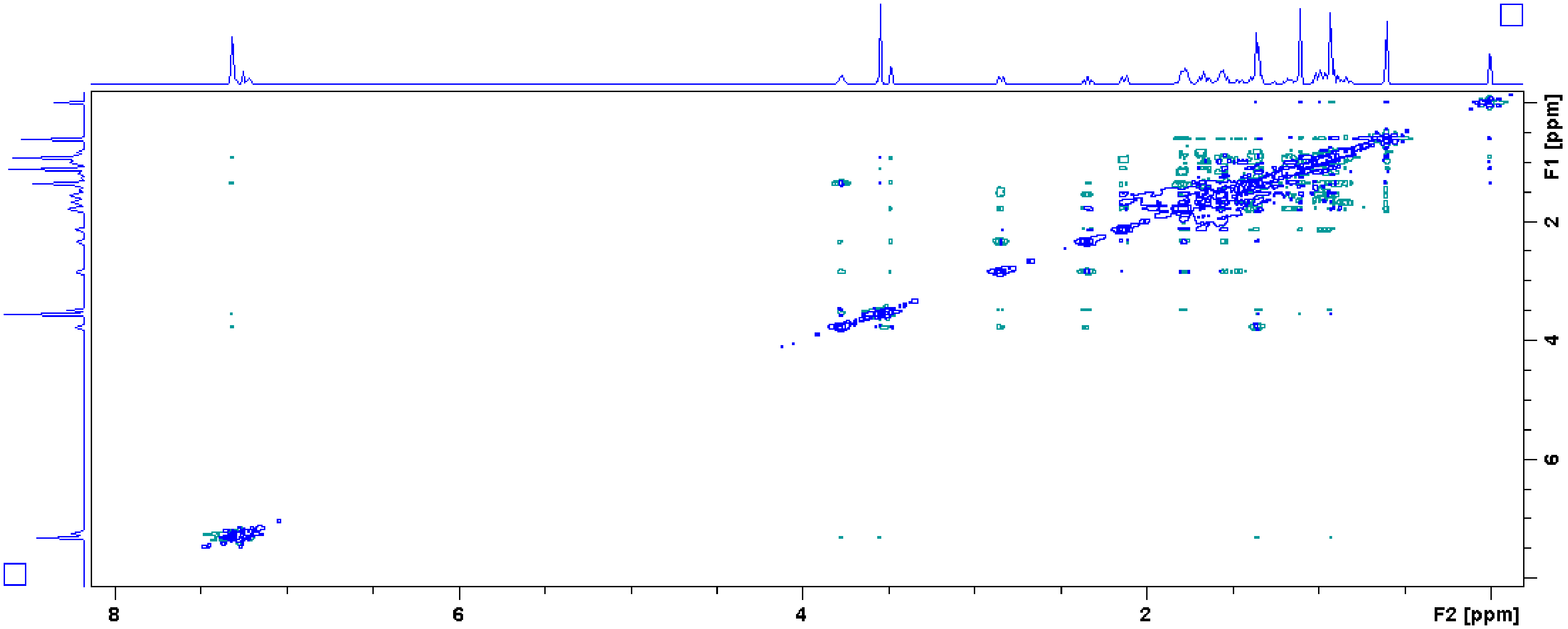

HSQC of compound (4*R*,6a*S*,8*R*,9*S*,11b*S*)-Methyl 8-hydroxy-4,9,11b-trimethyl-7-((((*R*)-1-phenylethyl)amino)methyl)tetradecahydro-6a,9-methanocyclohepta[a]naphthalene-4-carboxylate (**10**)

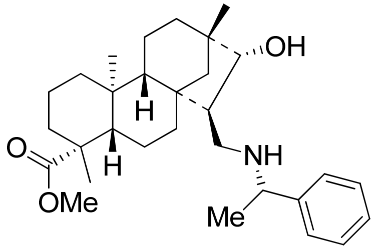

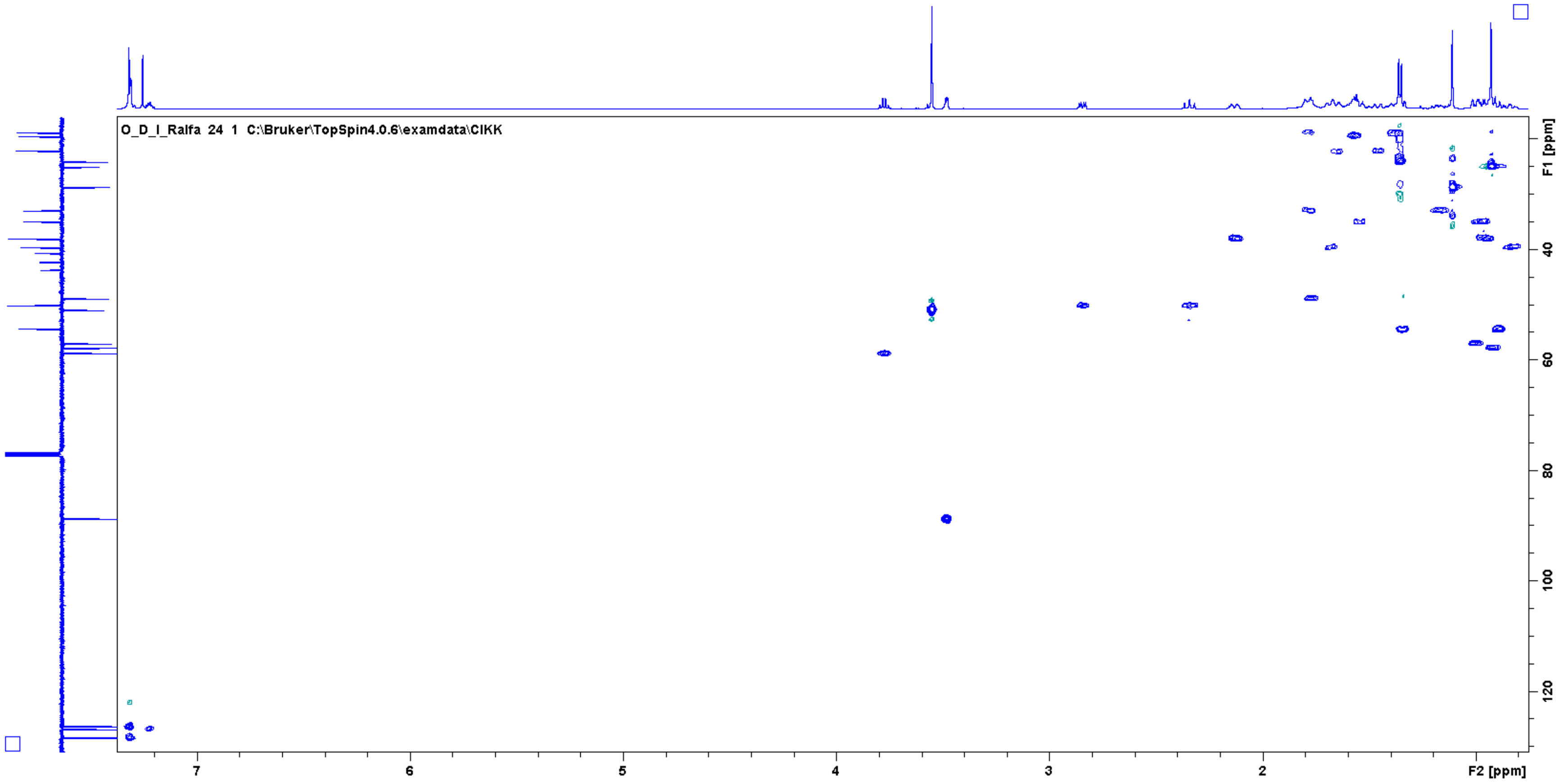

HMBC of compound (4*R*,6a*S*,8*R*,9*S*,11b*S*)-Methyl 8-hydroxy-4,9,11b-trimethyl-7-((((*R*)-1-phenylethyl)amino)methyl)tetradecahydro-6a,9-methanocyclohepta[a]naphthalene-4-carboxylate (**10**)

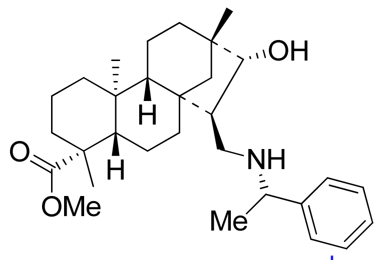

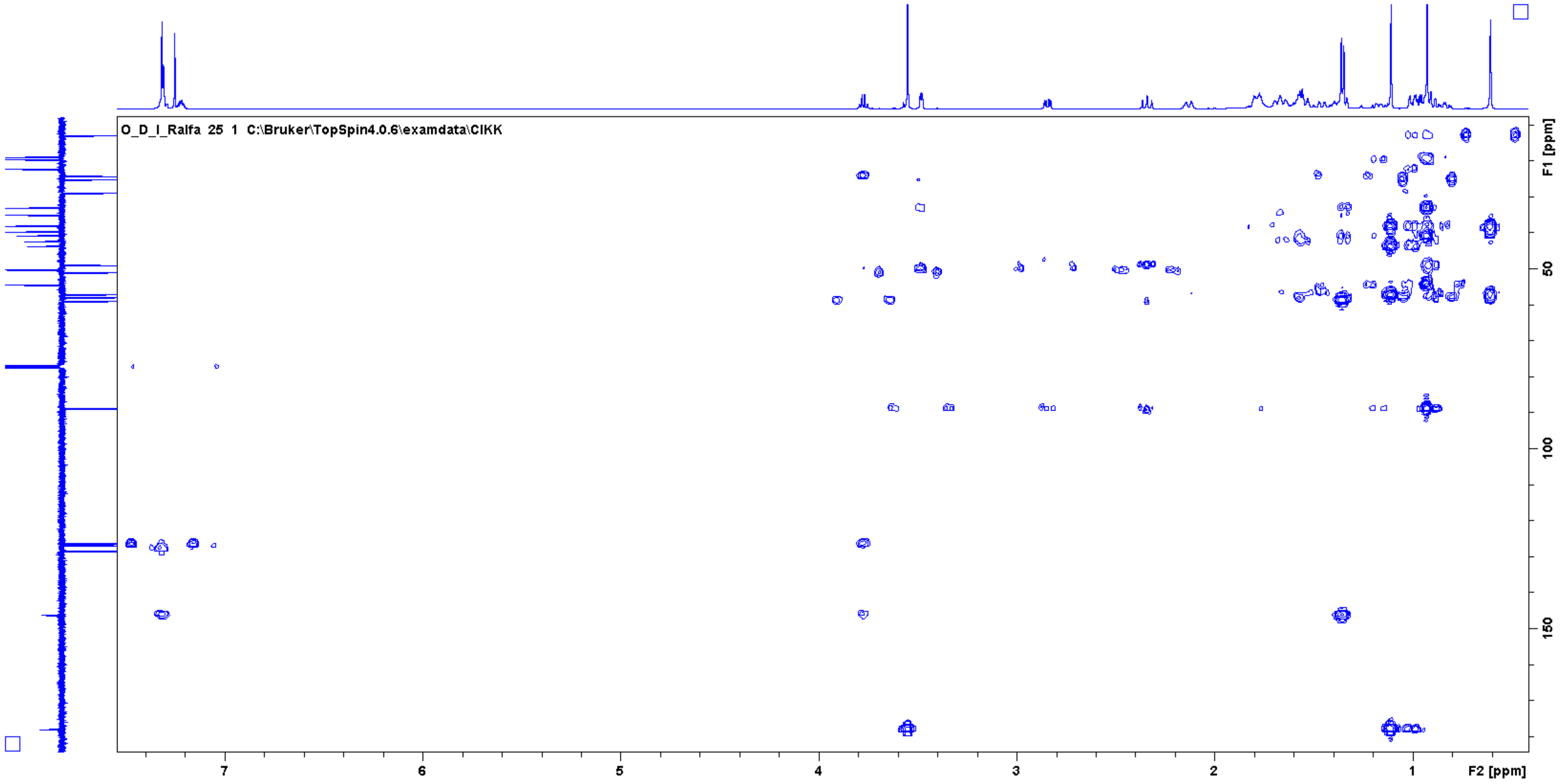

^{1}H-NMR of compound (4*R*,6a*S*,8*R*,9*S*,11b*S*)-Methyl 8-hydroxy-7-(((4-methoxybenzyl)amino)methyl)-4,9,11b-trimethyltetradecahydro-6a,9-methanocyclohepta[a]naphthalene-4-carboxylate (**11**)

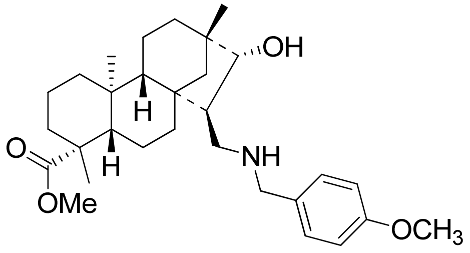

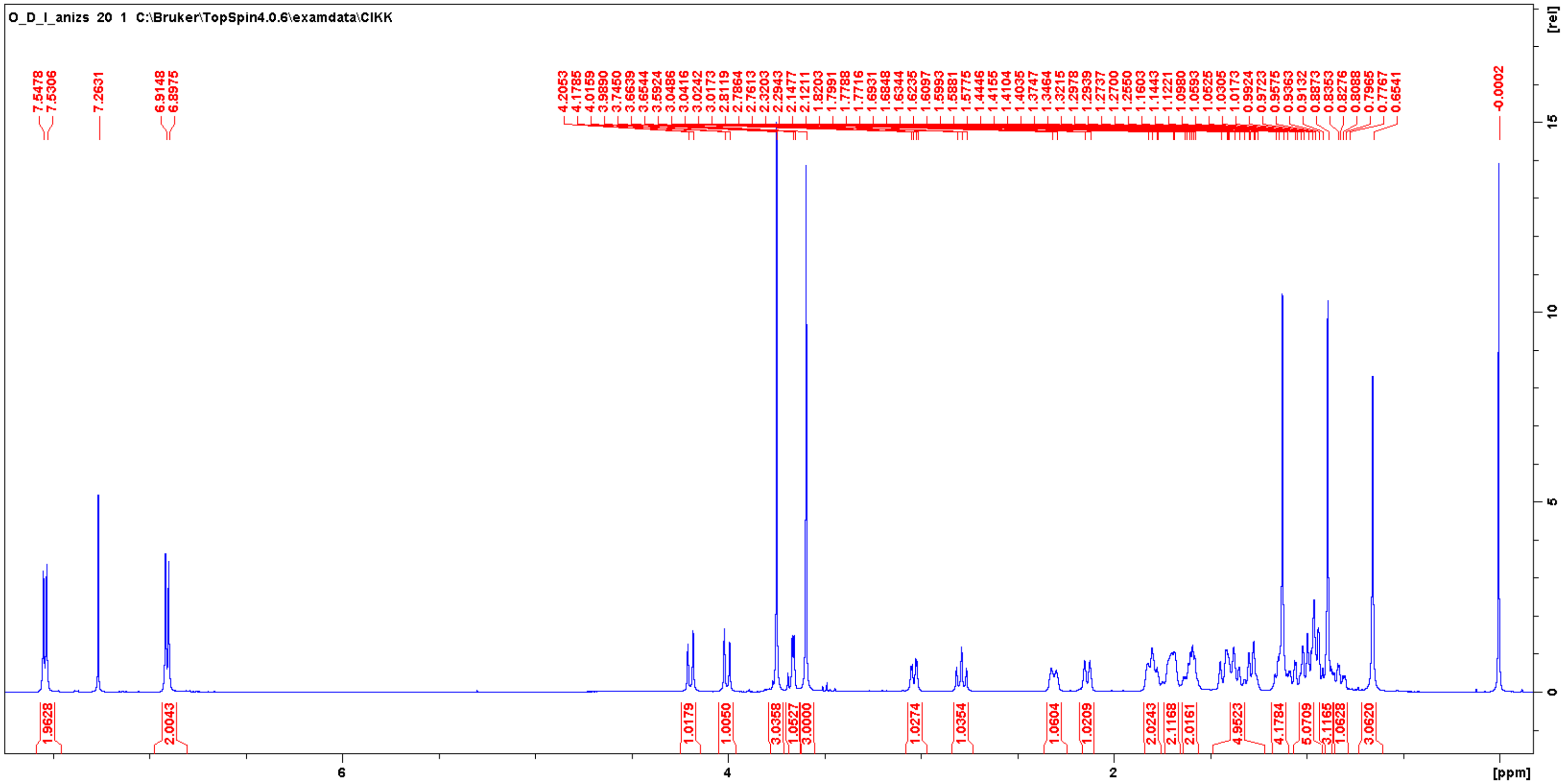

^{13}C-NMR of compound (4*R*,6a*S*,8*R*,9*S*,11b*S*)-Methyl 8-hydroxy-7-(((4-methoxybenzyl)amino)methyl)-4,9,11b-trimethyltetradecahydro-6a,9-methanocyclohepta[a]naphthalene-4-carboxylate (**11**)

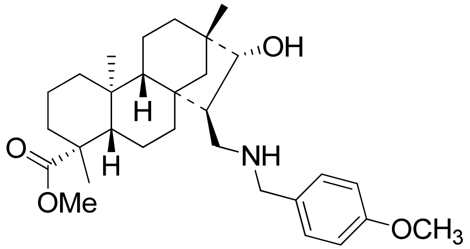

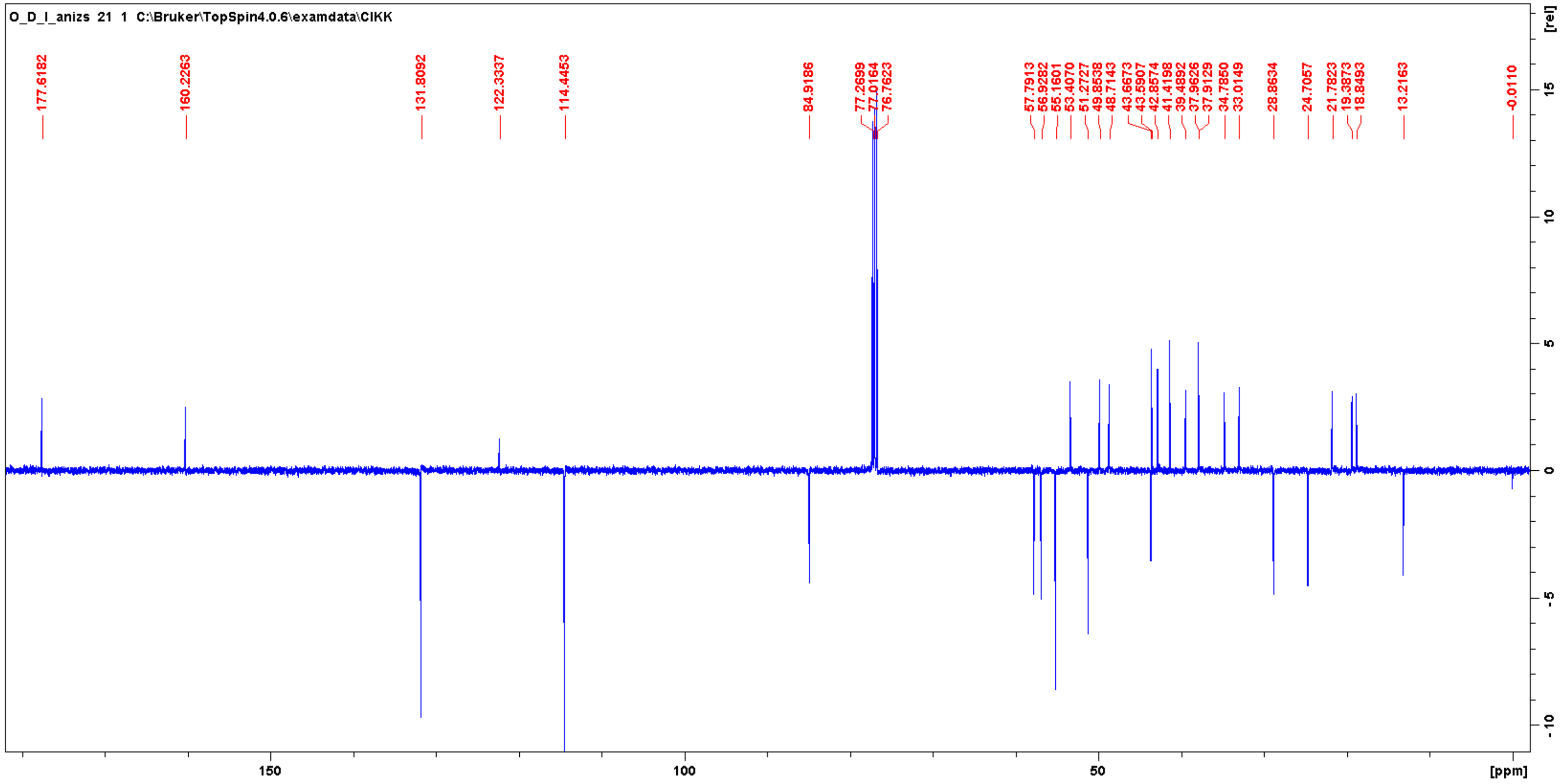

COSY of compound (4*R*,6a*S*,8*R*,9*S*,11b*S*)-Methyl 8-hydroxy-7-(((4-methoxybenzyl)amino)methyl)-4,9,11b-trimethyltetradecahydro-6a,9-methanocyclohepta[a]naphthalene-4-carboxylate (**11**)

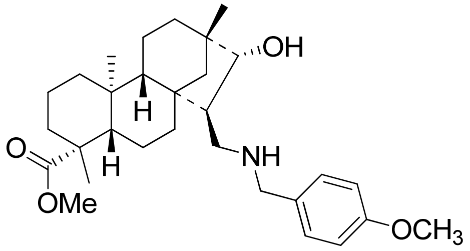

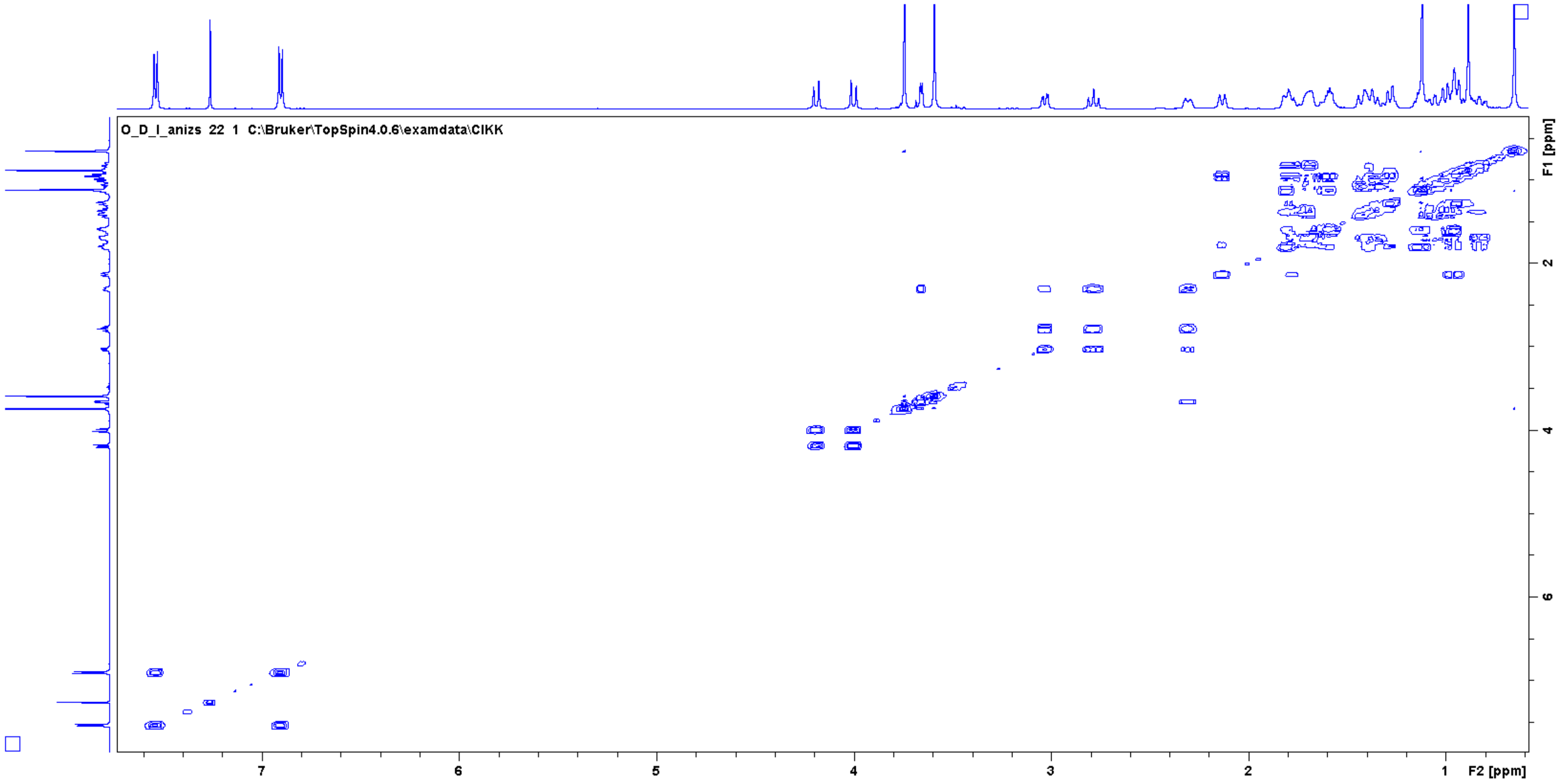

HSQC of compound (4*R*,6a*S*,8*R*,9*S*,11b*S*)-Methyl 8-hydroxy-7-(((4-methoxybenzyl)amino)methyl)-4,9,11b-trimethyltetradecahydro-6a,9-methanocyclohepta[a]naphthalene-4-carboxylate (**11**)

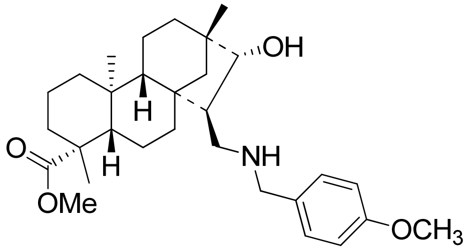

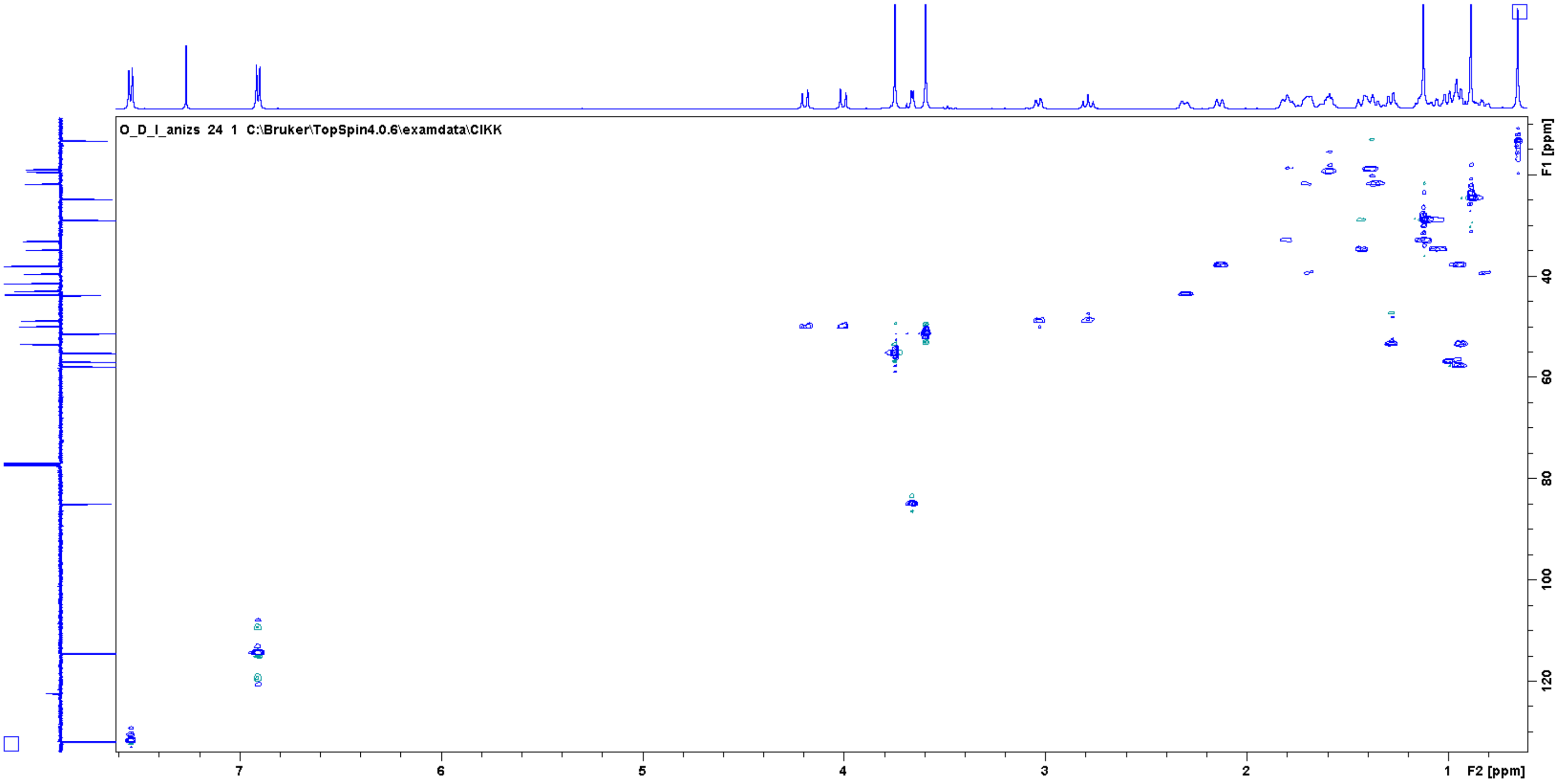

HMBC of compound (4*R*,6a*S*,8*R*,9*S*,11b*S*)-Methyl 8-hydroxy-7-(((4-methoxybenzyl)amino)methyl)-4,9,11b-trimethyltetradecahydro-6a,9-methanocyclohepta[a]naphthalene-4-carboxylate (**11**)

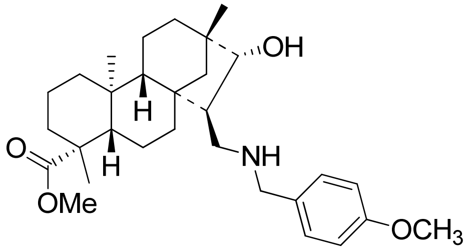

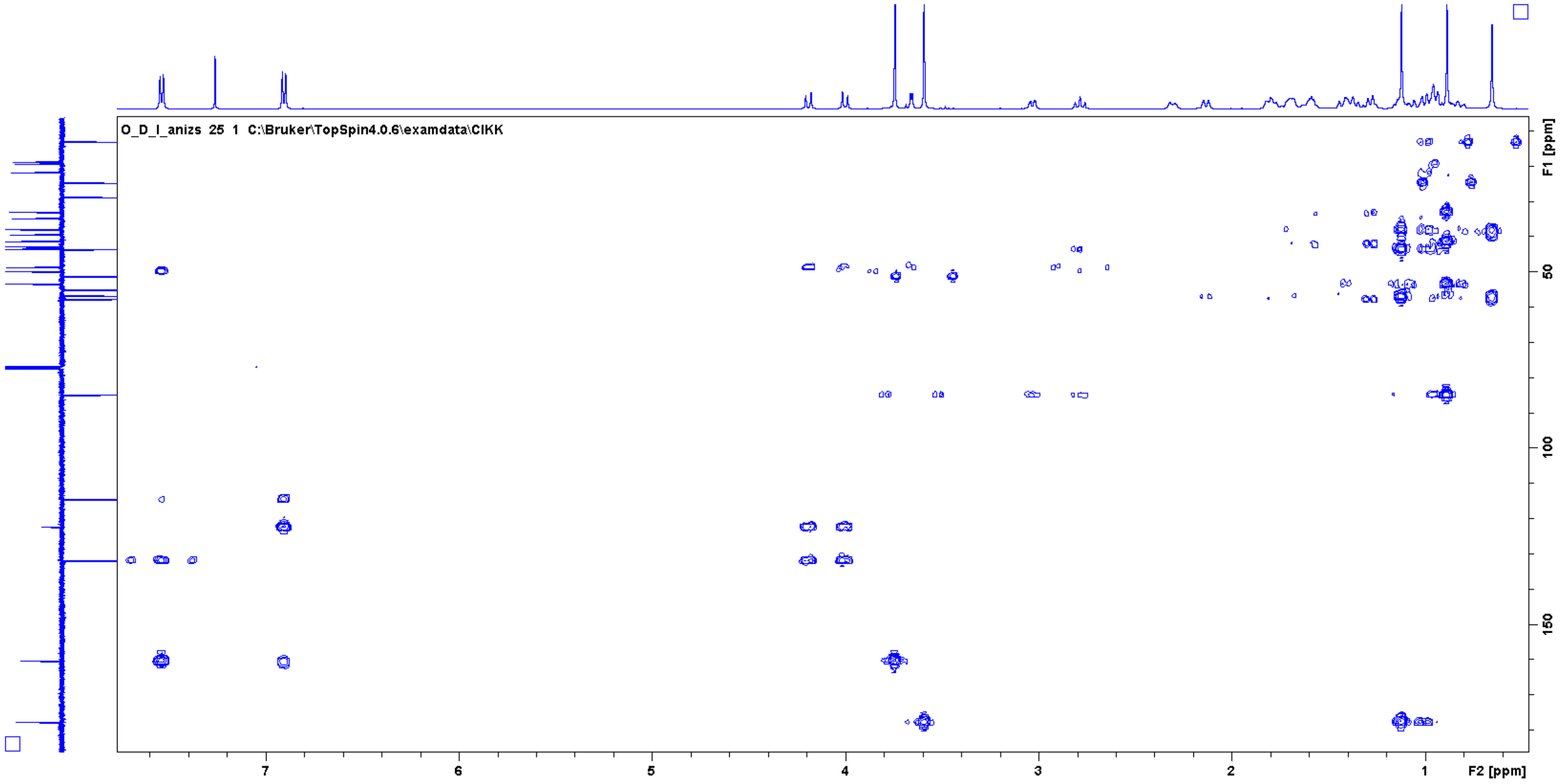

^{1}H-NMR of compound (4*R*,6a*S*,8*R*,9*S*,11b*S*)-Methyl 7-(((4-fluorobenzyl)amino)methyl)-8-hydroxy-4,9,11b-trimethyltetradecahydro-6a,9-methanocyclohepta[a]naphthalene-4-carboxylate (**12**)

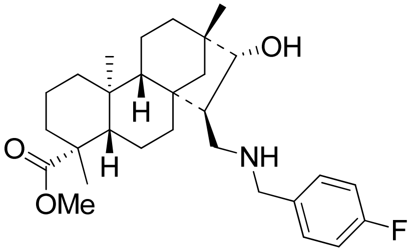

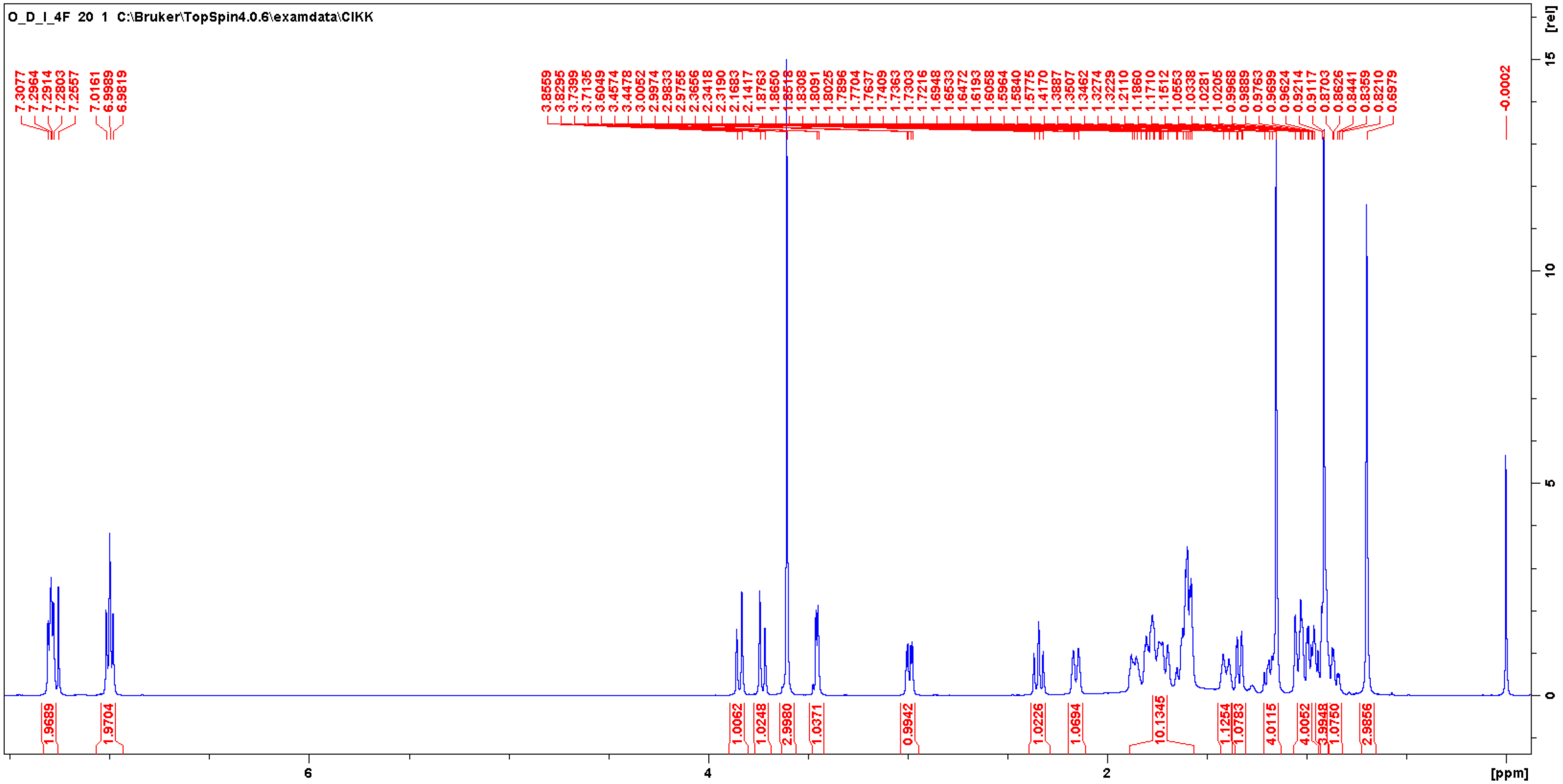

^{13}C-NMR of compound (4*R*,6a*S*,8*R*,9*S*,11b*S*)-Methyl 7-(((4-fluorobenzyl)amino)methyl)-8-hydroxy-4,9,11b-trimethyltetradecahydro-6a,9-methanocyclohepta[a]naphthalene-4-carboxylate (**12**)

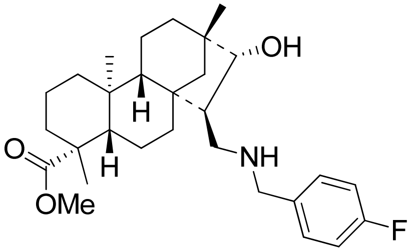

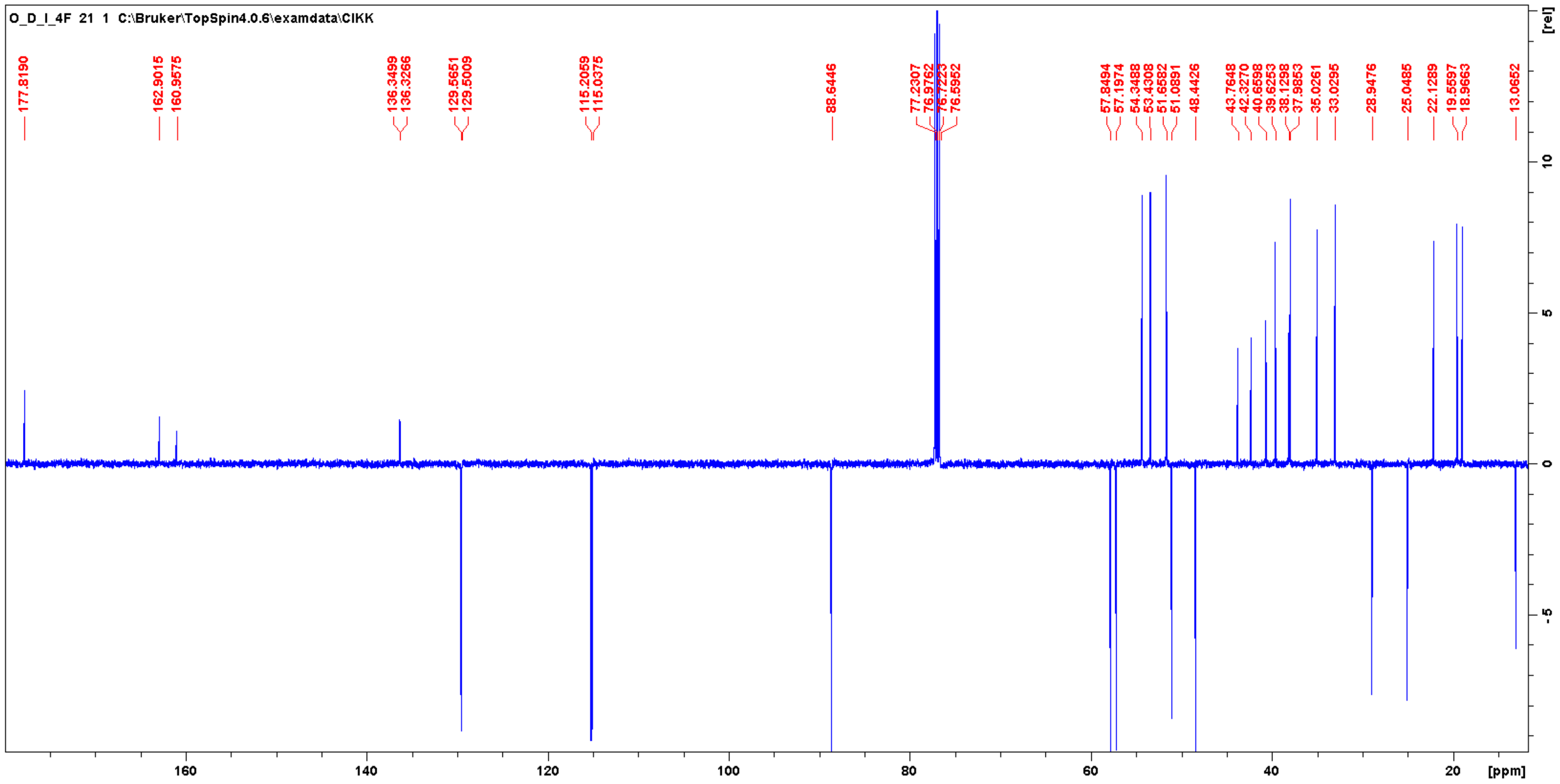

COSY of compound (4*R*,6a*S*,8*R*,9*S*,11b*S*)-Methyl 7-(((4-fluorobenzyl)amino)methyl)-8-hydroxy-4,9,11b-trimethyltetradecahydro-6a,9-methanocyclohepta[a]naphthalene-4-carboxylate (**12**)

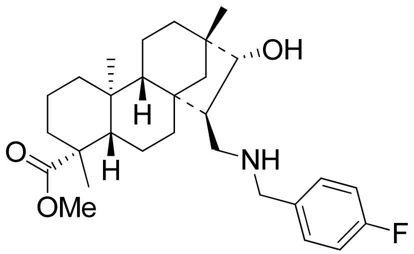
OH
H
H
O
OMe
NH
F

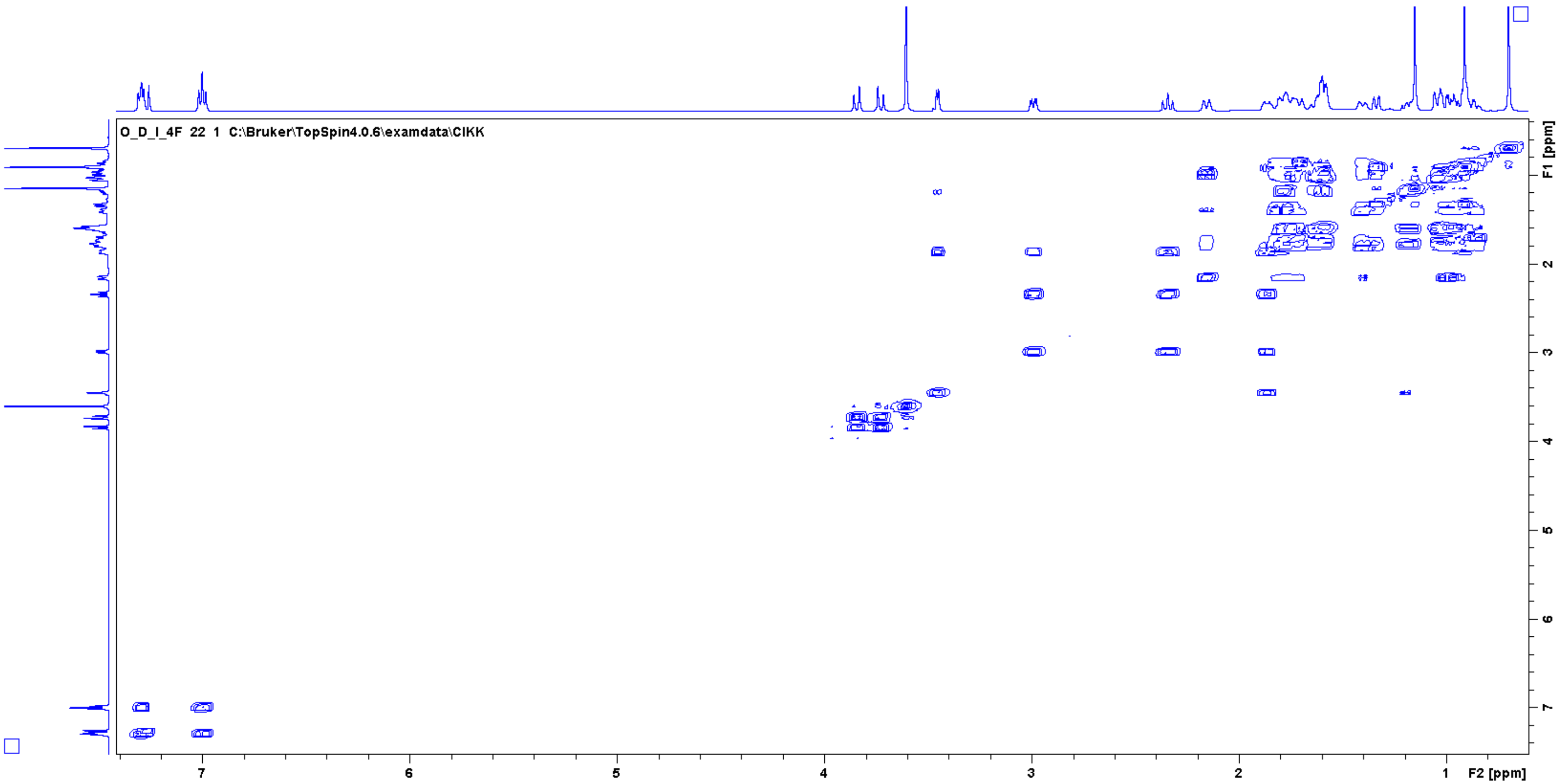
O_D_I_4F 22 1 C:\Bruker\TopSpin4.0.6\examdata\CIKK
F1 [ppm]
F2 [ppm]
7
6
5
4
3
2
1

NOESY of compound (4*R*,6a*S*,8*R*,9*S*,11b*S*)-Methyl 7-(((4-fluorobenzyl)amino)methyl)-8-hydroxy-4,9,11b-trimethyltetradecahydro-6a,9-methanocyclohepta[a]naphthalene-4-carboxylate (**12**)

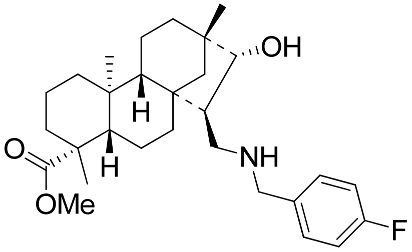

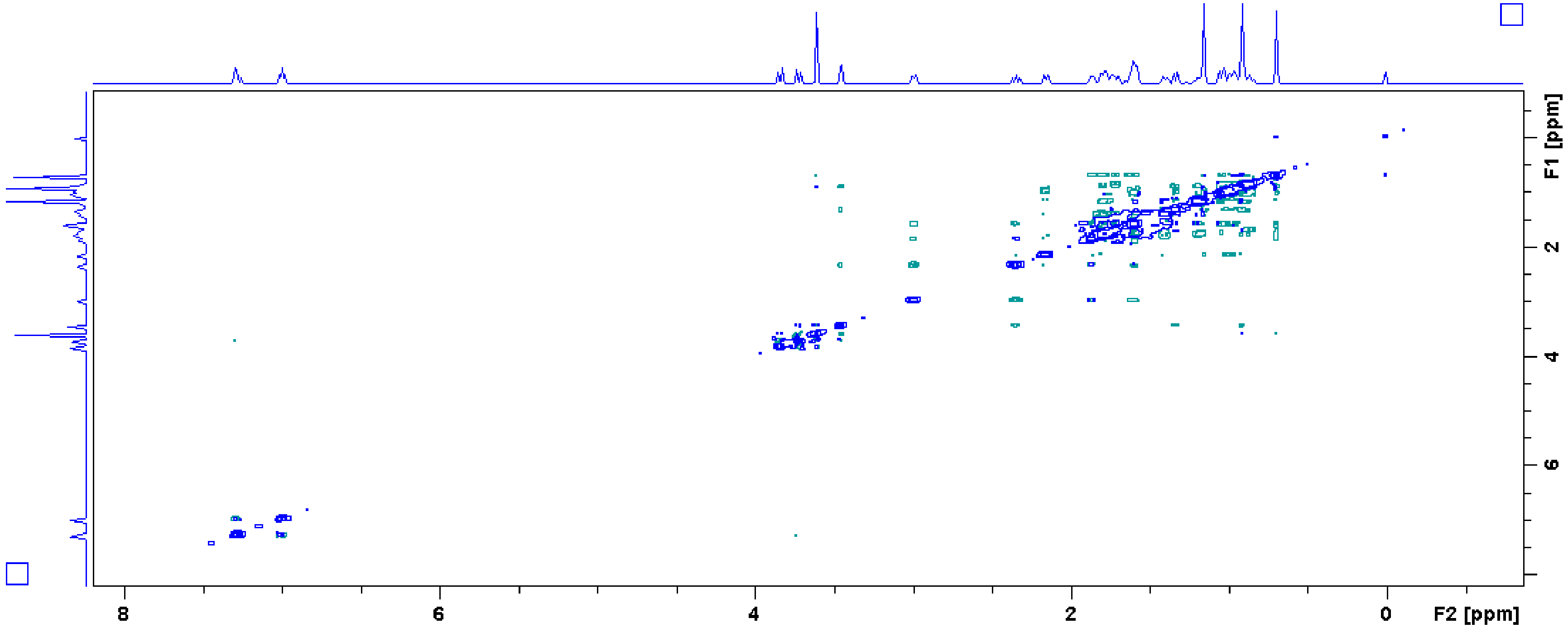

HSQC of compound (4*R*,6a*S*,8*R*,9*S*,11b*S*)-Methyl 7-(((4-fluorobenzyl)amino)methyl)-8-hydroxy-4,9,11b-trimethyltetradecahydro-6a,9-methanocyclohepta[a]naphthalene-4-carboxylate (**12**)

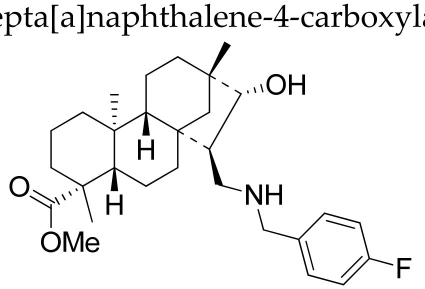

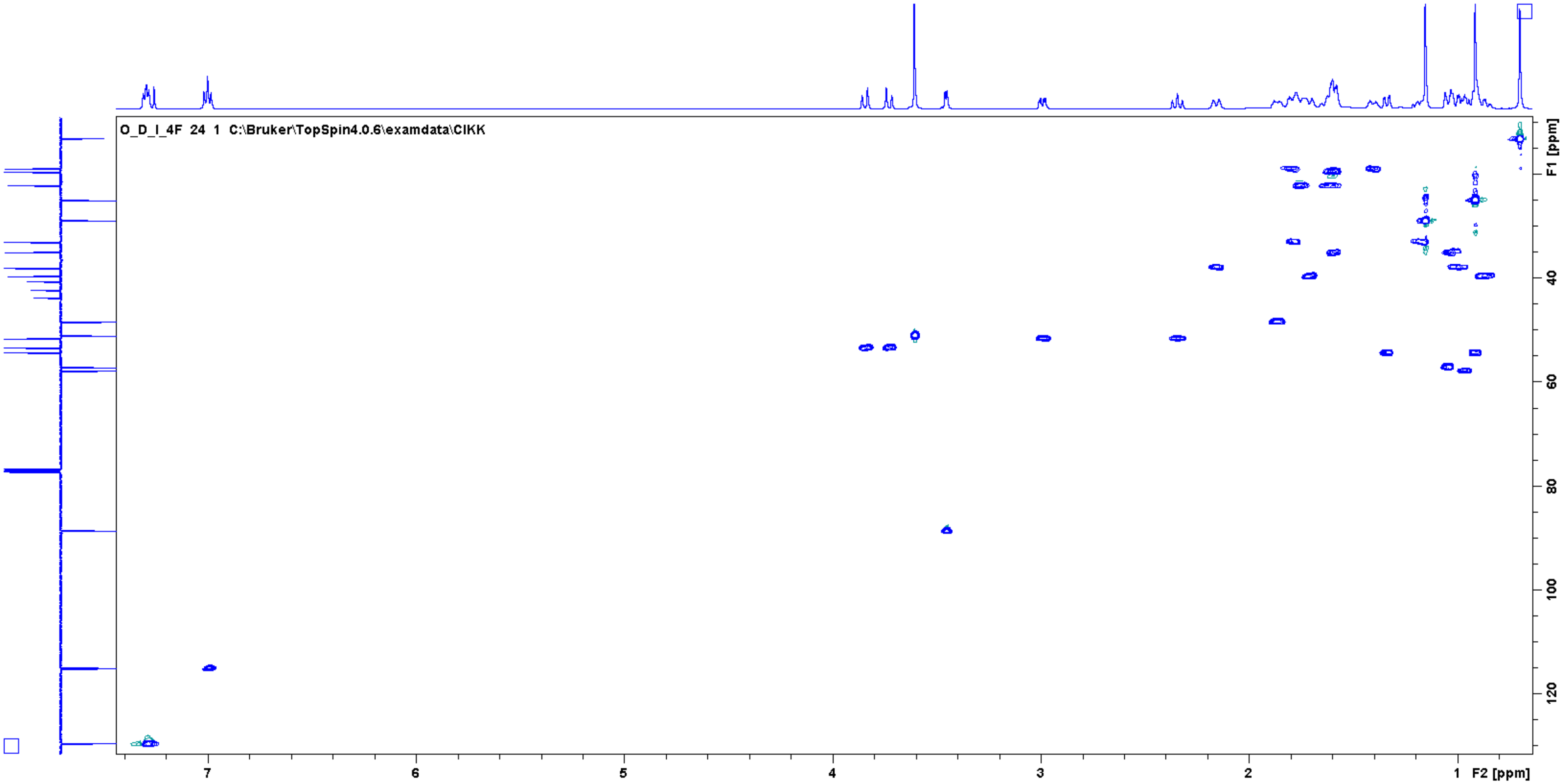

HMBC of compound (4*R*,6a*S*,8*R*,9*S*,11b*S*)-Methyl 7-(((4-fluorobenzyl)amino)methyl)-8-hydroxy-4,9,11b-trimethyltetradecahydro-6a,9-methanocyclohepta[a]naphthalene-4-carboxylate (**12**)

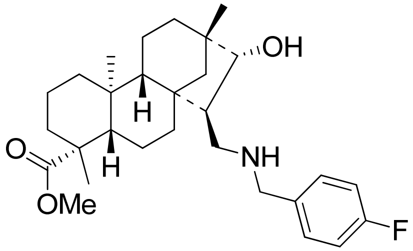

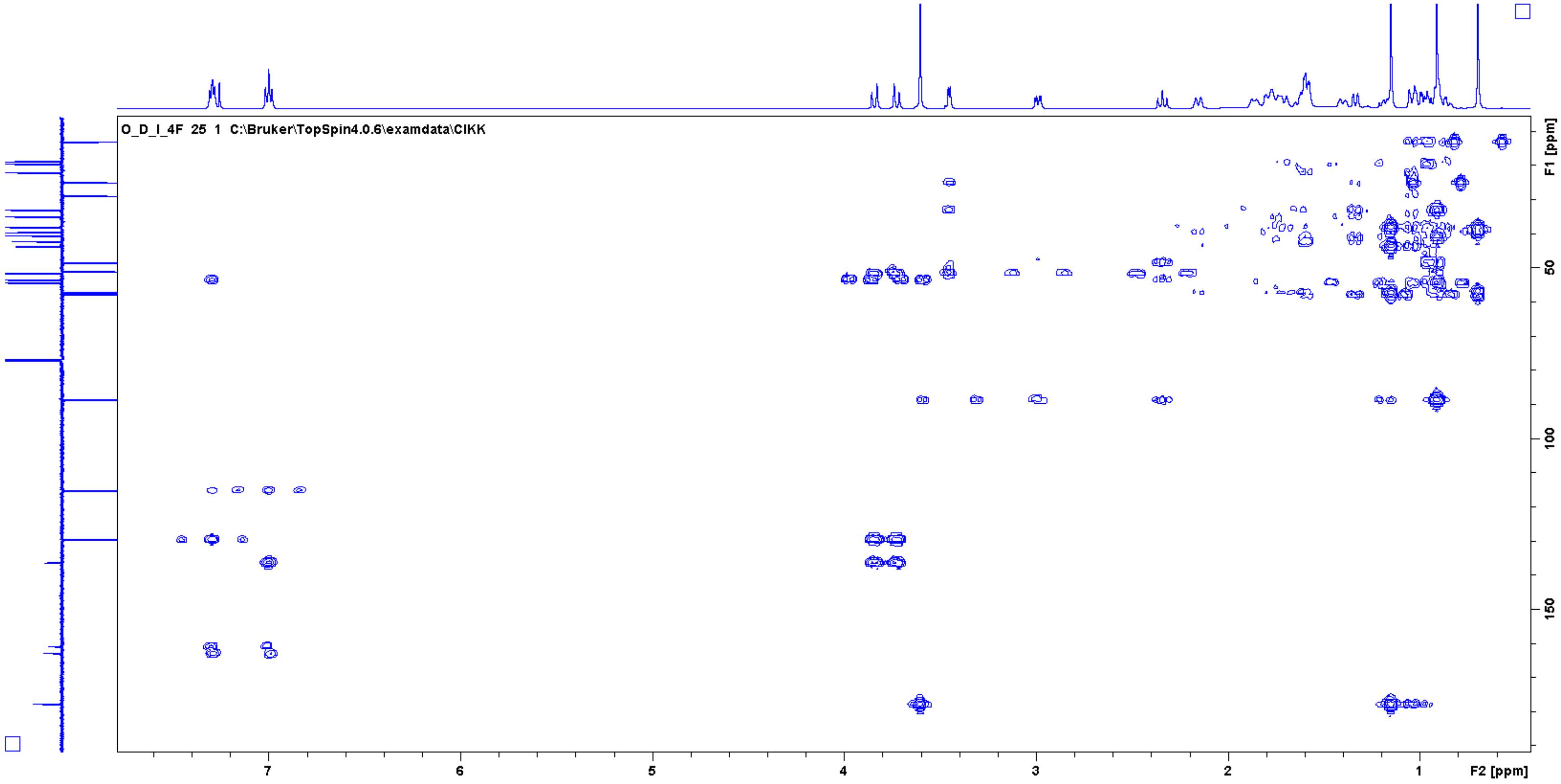

^{19}F-NMR of compound (4*R*,6a*S*,8*R*,9*S*,11b*S*)-Methyl 7-(((4-fluorobenzyl)amino)methyl)-8-hydroxy-4,9,11b-trimethyltetradecahydro-6a,9-methanocyclohepta[a]naphthalene-4-carboxylate (**12**)

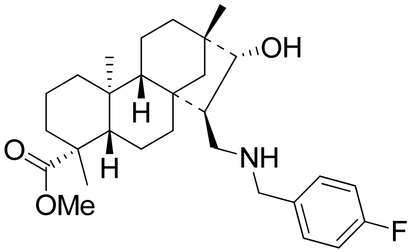
OH
NH
F
O
OMe
H
H

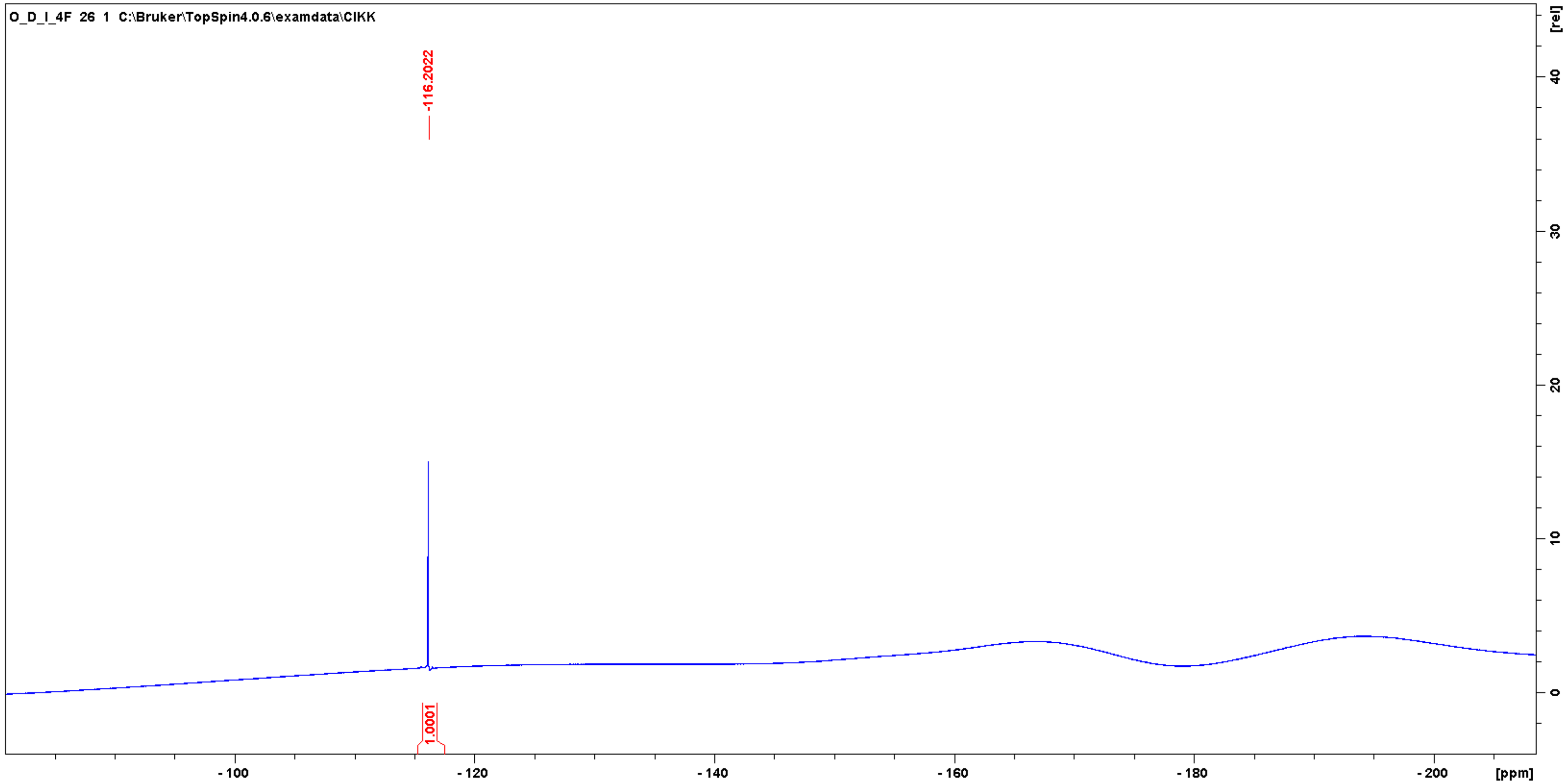
O_D_I_4F 26 1 C:\Bruker\TopSpin4.0.6\examdata\CIKK
-116.2022
1.0001
-100
-120
-140
-160
-180
-200
[ppm]
[rel]
0
10
20
30
40

^{1}H-NMR of compound (4*R*,6a*S*,9*S*,11b*S*)-Methyl 4,9,11b-trimethyl-7-(morpholinomethyl)-8-oxotetradecahydro-6a,9-methanocyclohepta[a]naphthalene-4-carboxylate (**14**)

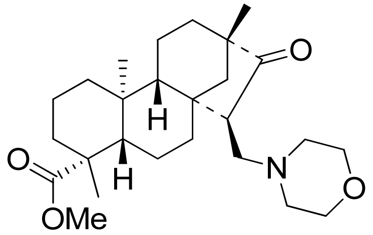

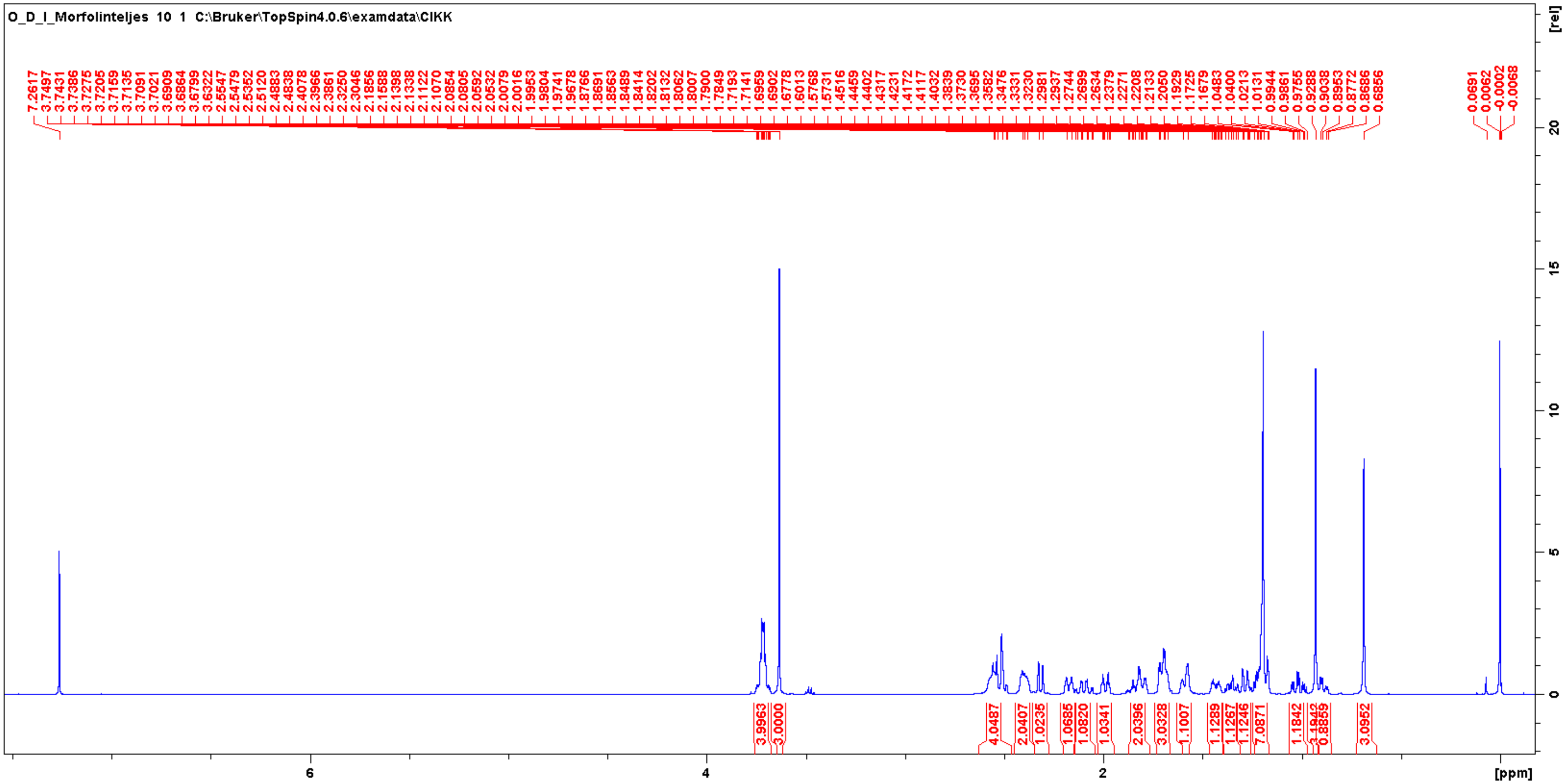

^{13}C-NMR of compound (4*R*,6a*S*,9*S*,11b*S*)-Methyl 4,9,11b-trimethyl-7-(morpholinomethyl)-8-oxotetradecahydro-6a,9-methanocyclohepta[a]naphthalene-4-carboxylate (**14**)

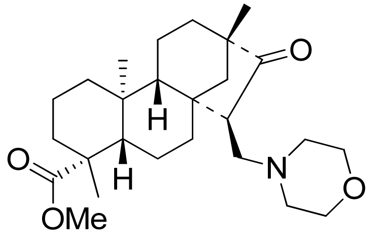

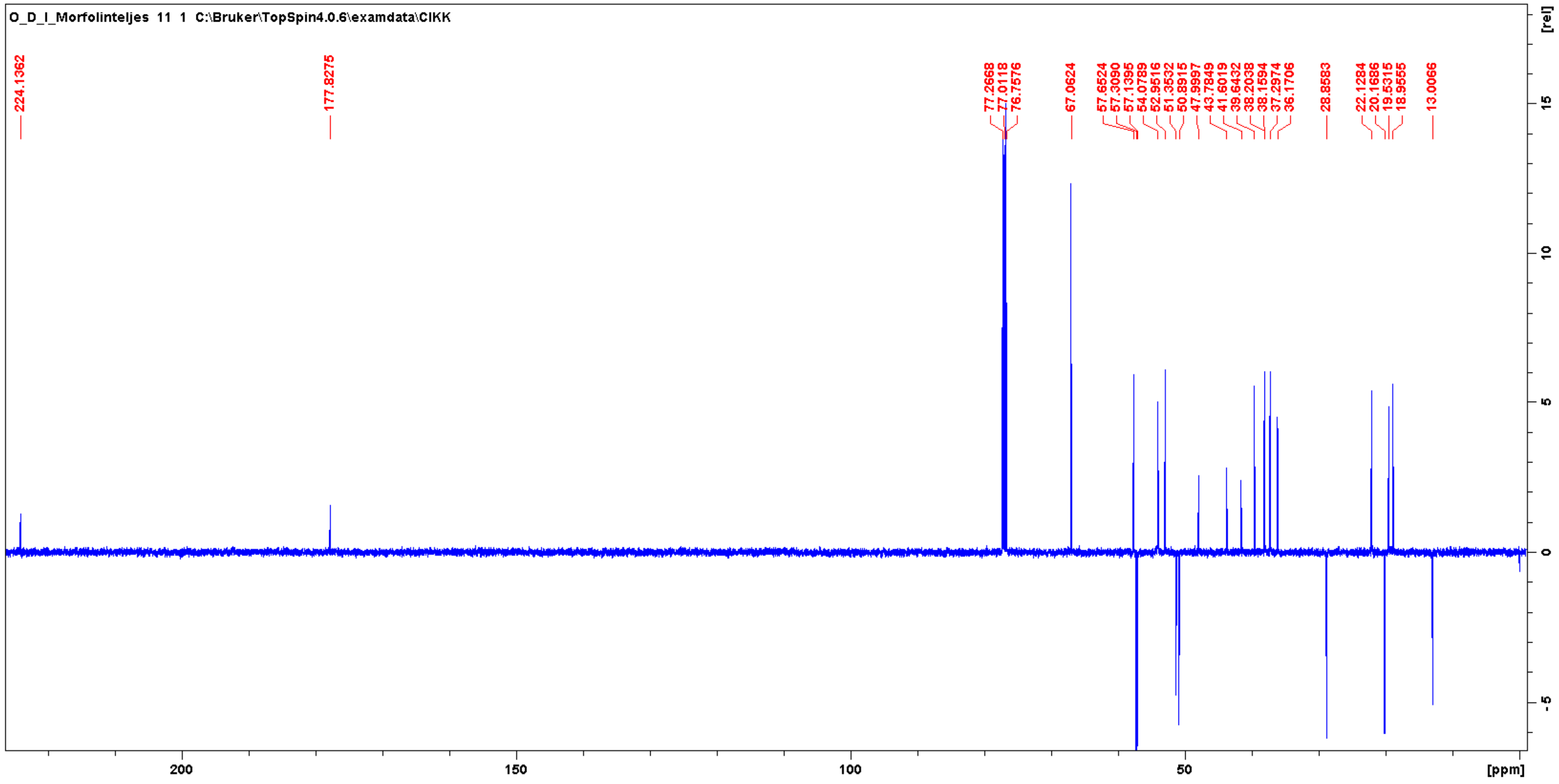

COSY of compound (4*R*,6a*S*,9*S*,11b*S*)-Methyl 4,9,11b-trimethyl-7-(morpholinomethyl)-8-oxotetradecahydro-6a,9-methanocyclohepta[a]naphthalene-4-carboxylate (**14**)

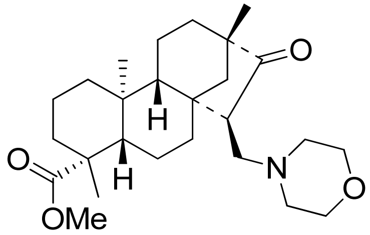

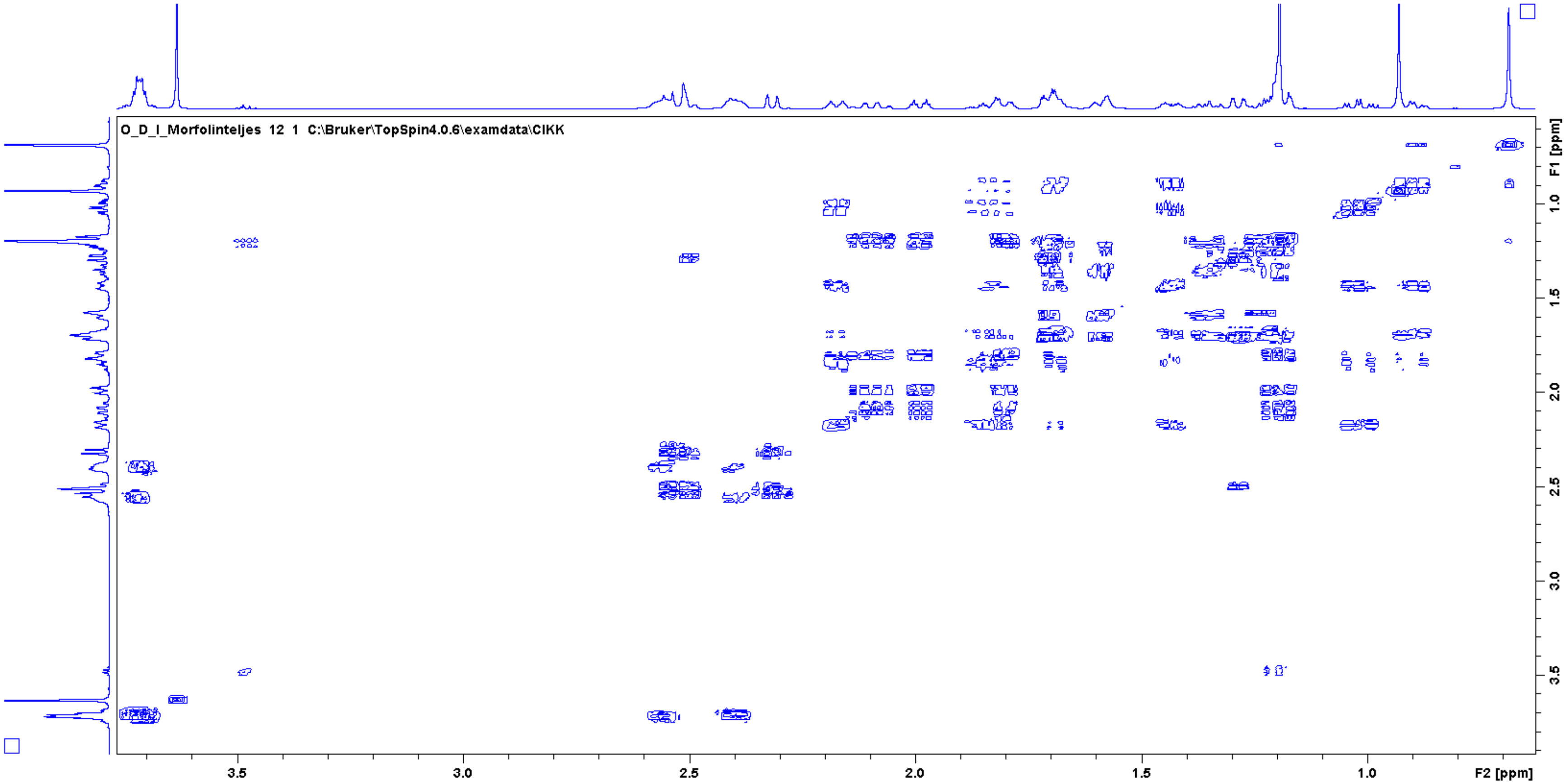

NOESY of compound (4*R*,6a*S*,9*S*,11b*S*)-Methyl 4,9,11b-trimethyl-7-(morpholinomethyl)-8-oxotetradecahydro-6a,9-methanocyclohepta[a]naphthalene-4-carboxylate (**14**)

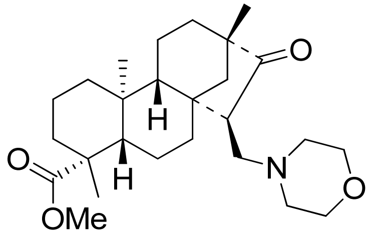

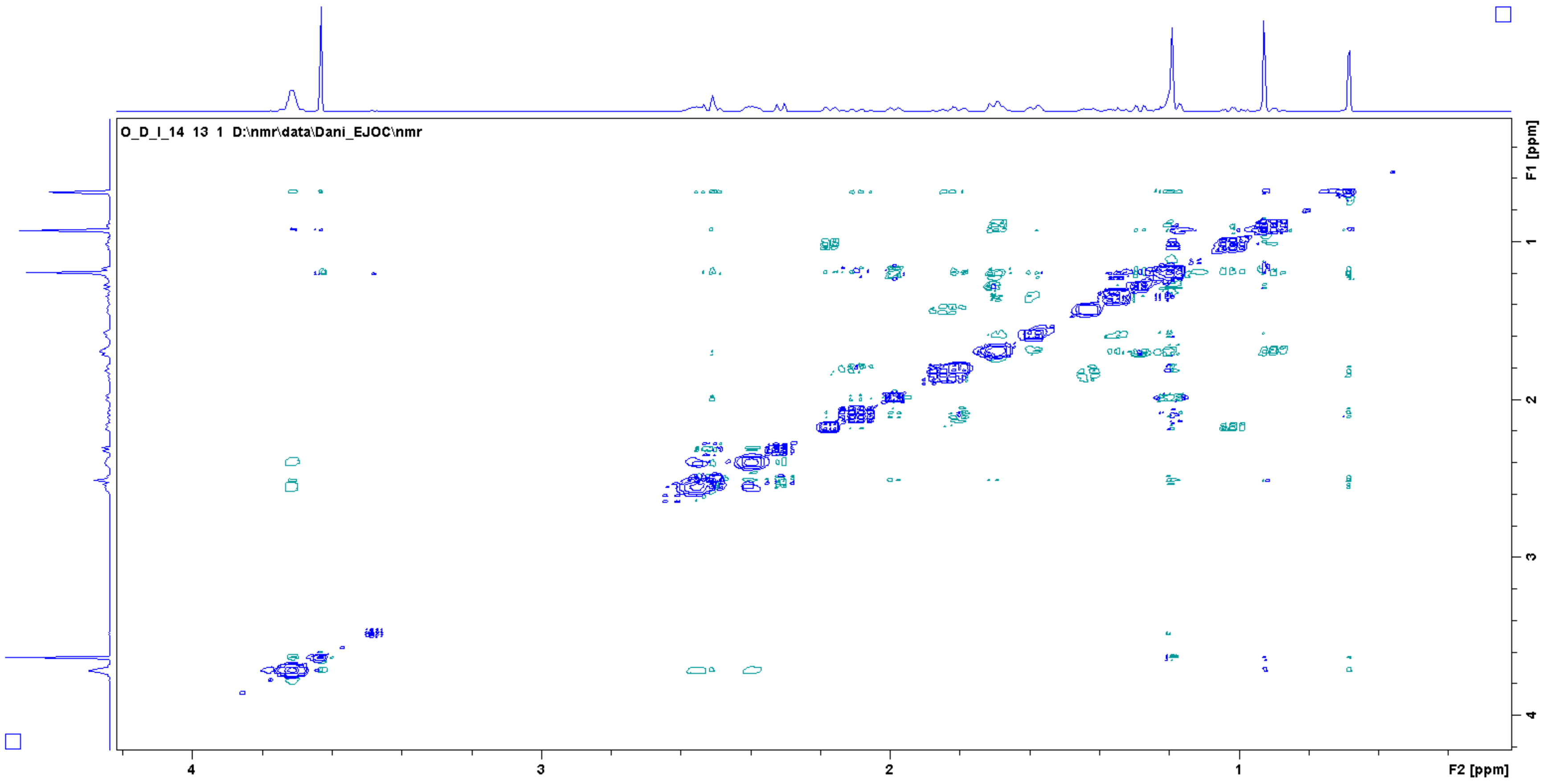

HSQC of compound (4*R*,6a*S*,9*S*,11b*S*)-Methyl 4,9,11b-trimethyl-7-(morpholinomethyl)-8-oxotetradecahydro-6a,9-methanocyclohepta[a]naphthalene-4-carboxylate (**14**)

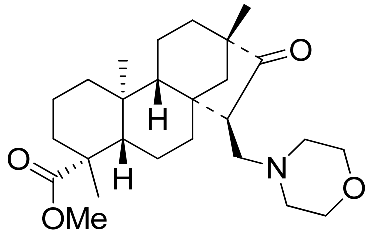

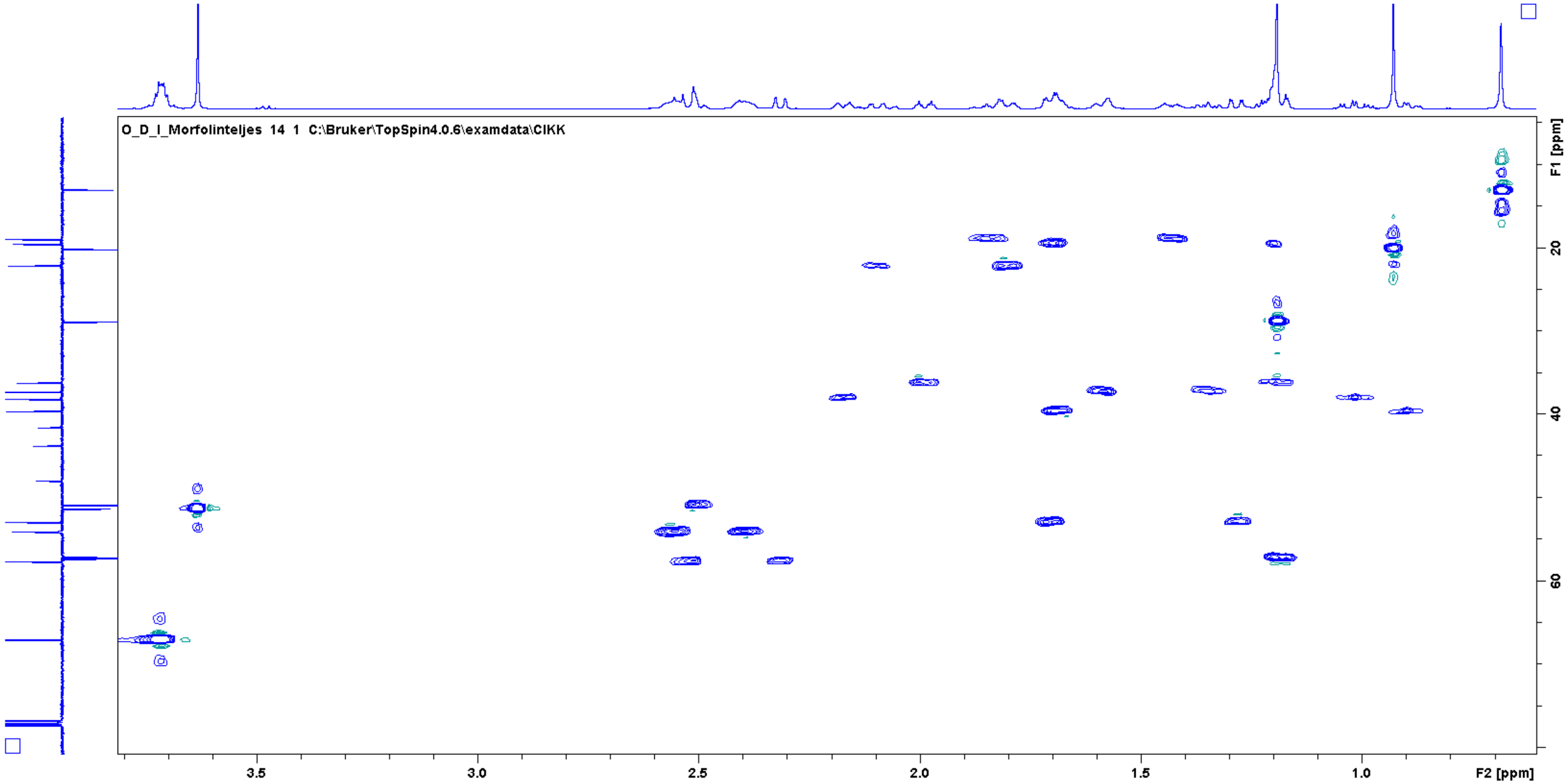

HMBC of compound (4*R*,6a*S*,9*S*,11b*S*)-Methyl 4,9,11b-trimethyl-7-(morpholinomethyl)-8-oxotetradecahydro-6a,9-methanocyclohepta[a]naphthalene-4-carboxylate (**14**)

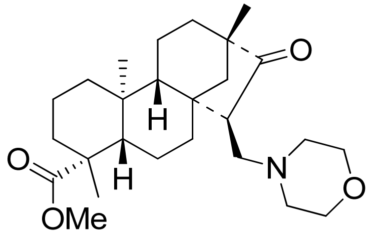

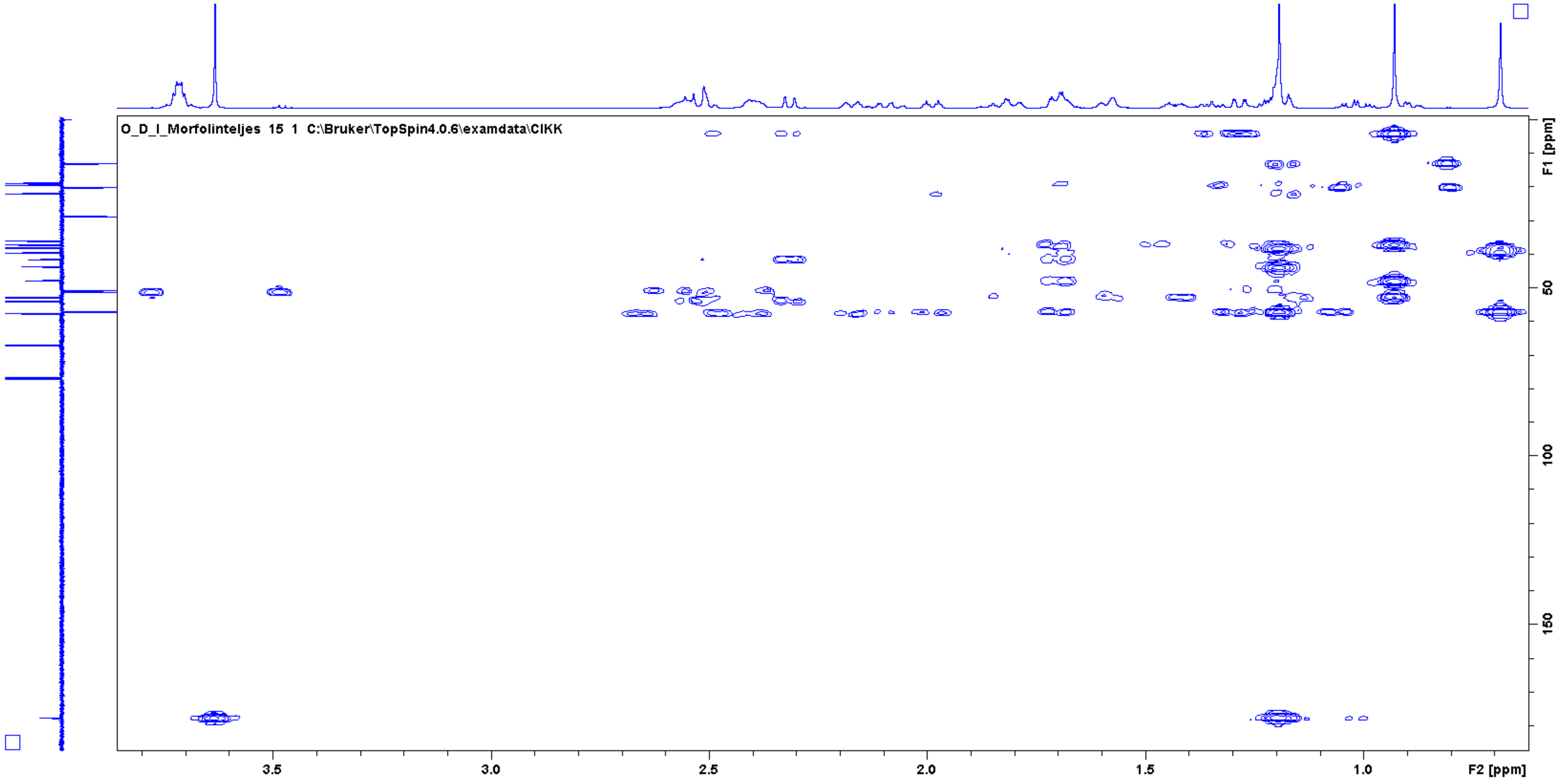

^{1}H-NMR of compound (4*R*,6a*S*,9*S*,11b*S*)-Methyl 7-((benzyl(methyl)amino)methyl)-4,9,11b-trimethyl-8-oxotetradecahydro-6a,9-methanocyclohepta[a]naphthalene-4-carboxylate (**15**)

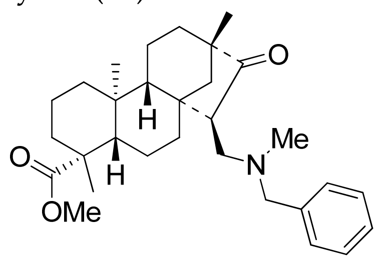

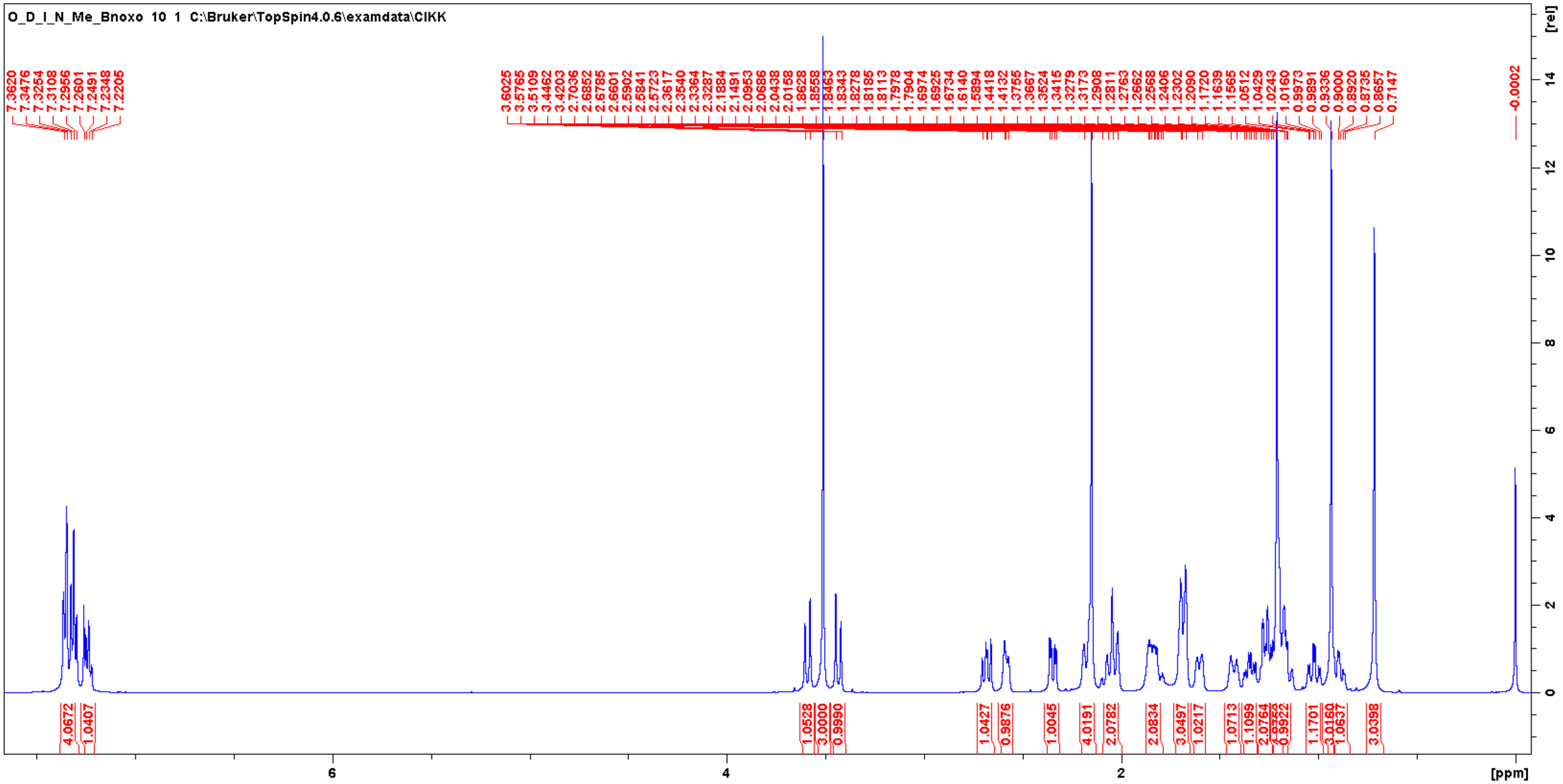

^{13}C-NMR of compound (4*R*,6a*S*,9*S*,11b*S*)-Methyl 7-((benzyl(methyl)amino)methyl)-4,9,11b-trimethyl-8-oxotetradecahydro-6a,9-methanocyclohepta[a]naphthalene-4-carboxylate (**15**)

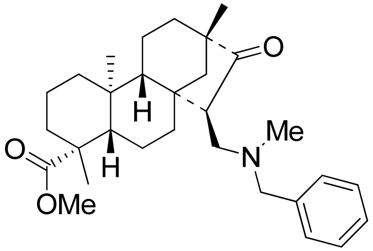

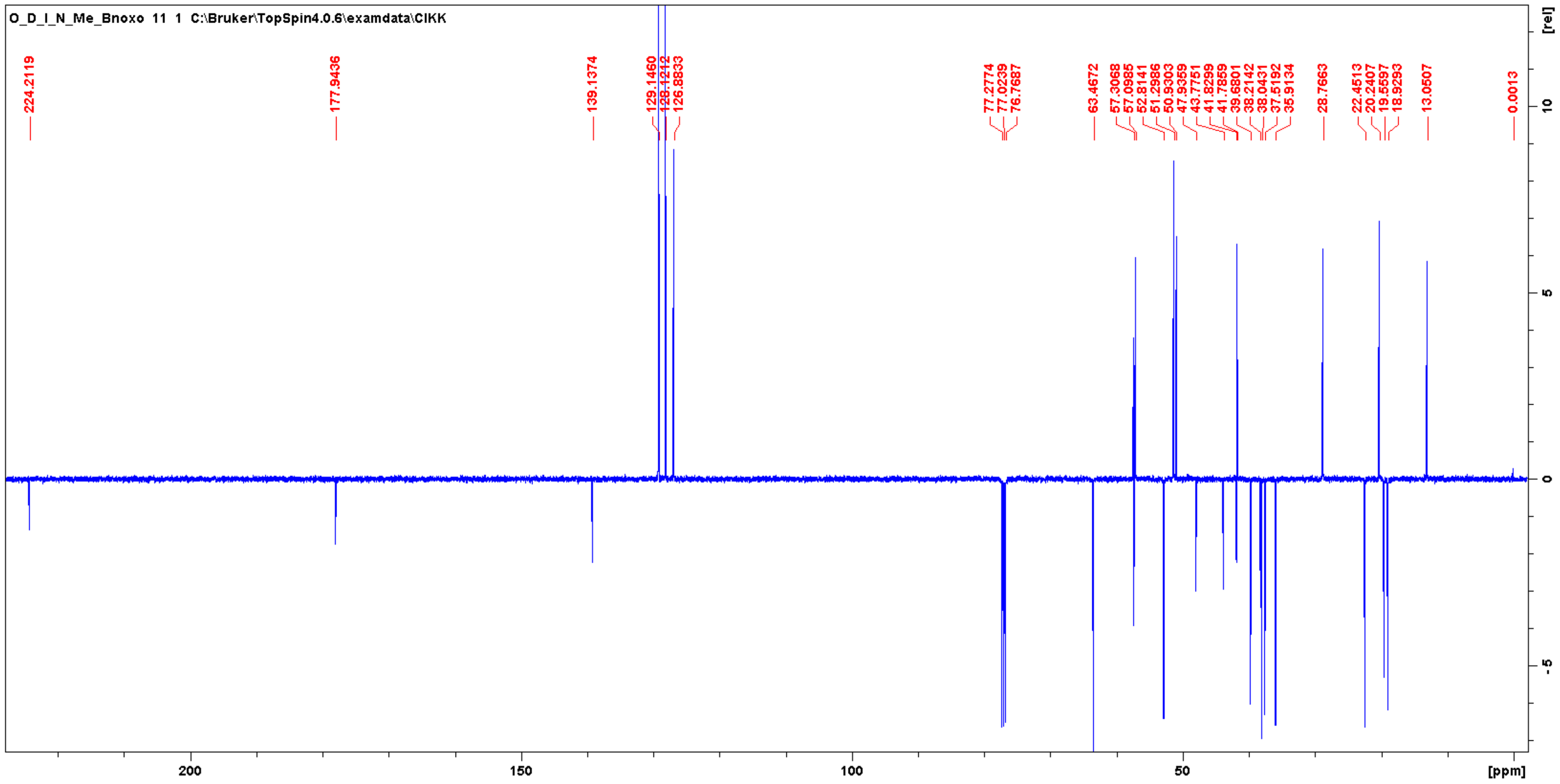

COSY of compound (4*R*,6a*S*,9*S*,11b*S*)-Methyl 7-((benzyl(methyl)amino)methyl)-4,9,11b-trimethyl-8-oxotetradecahydro-6a,9-methanocyclohepta[a]naphthalene-4-carboxylate (**15**)

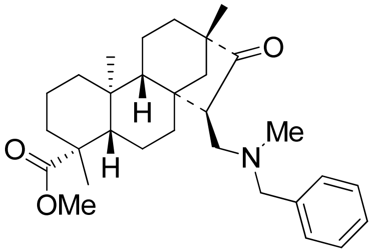
O
H
Me
N
H
O
OMe

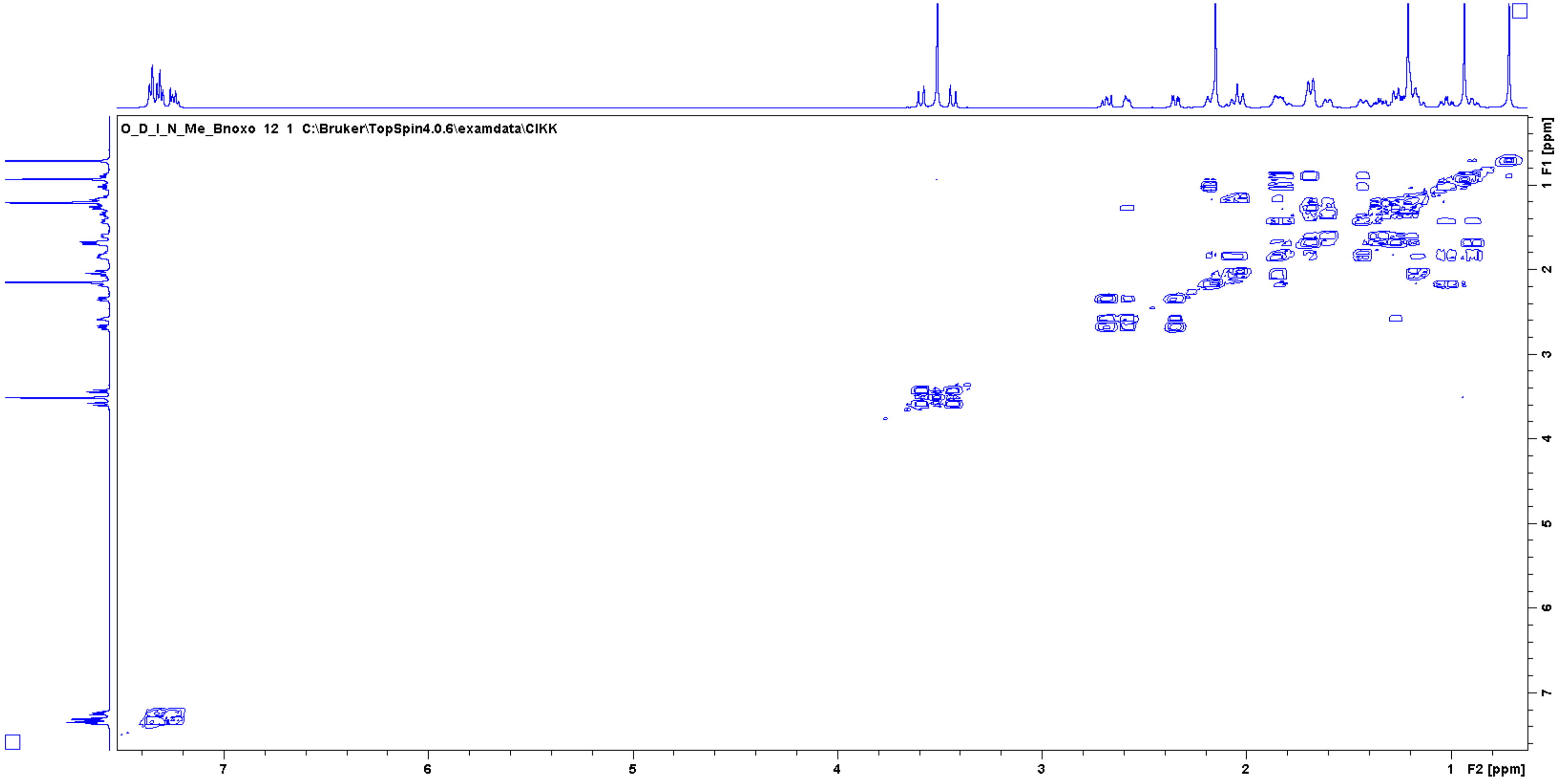
O_D_I_N_Me_Bnoxo 12 1 C:\Bruker\TopSpin4.0.6\examdata\CIKK
F1 [ppm]
F2 [ppm]

NOESY of compound (4*R*,6a*S*,9*S*,11b*S*)-Methyl 7-((benzyl(methyl)amino)methyl)-4,9,11b-trimethyl-8-oxotetradecahydro-6a,9-methanocyclohepta[a]naphthalene-4-carboxylate (**15**)

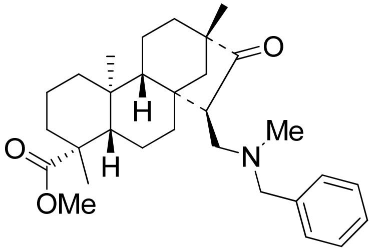

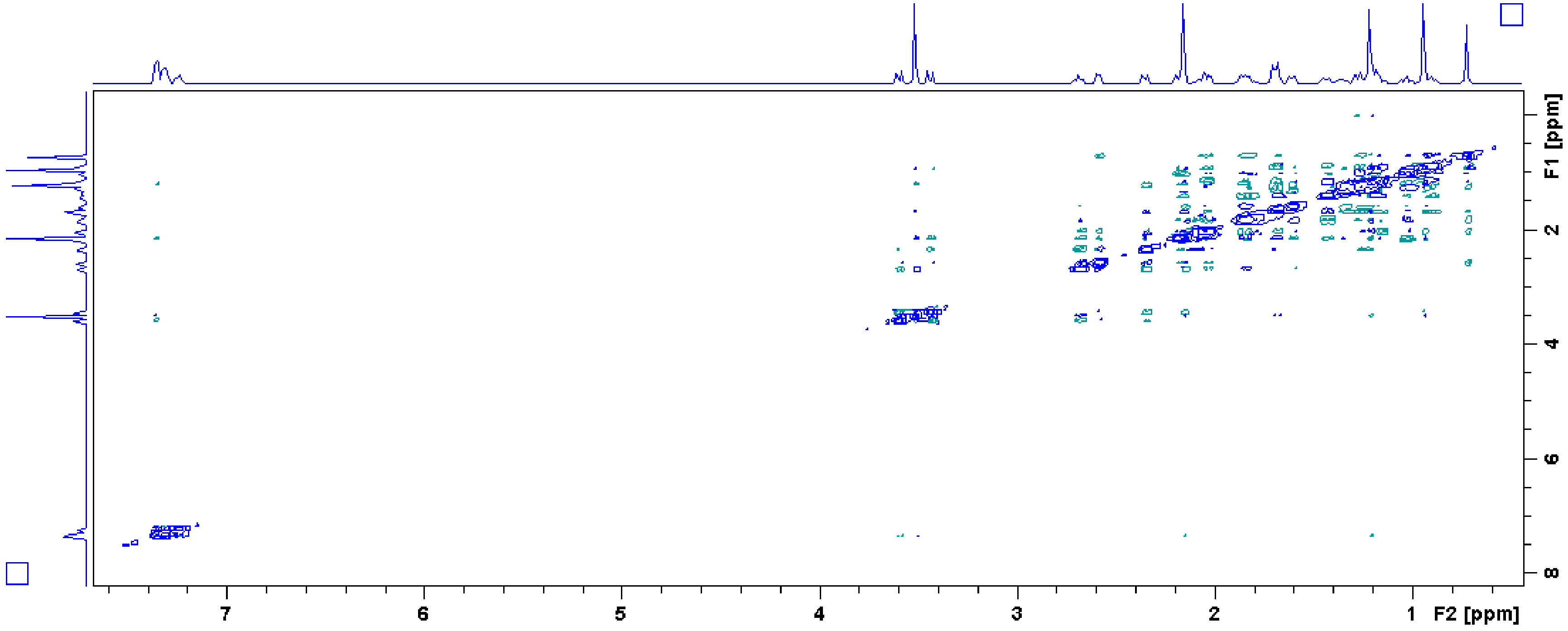

HSQC of compound (4*R*,6a*S*,9*S*,11b*S*)-Methyl 7-((benzyl(methyl)amino)methyl)-4,9,11b-trimethyl-8-oxotetradecahydro-6a,9-methanocyclohepta[a]naphthalene-4-carboxylate (**15**)

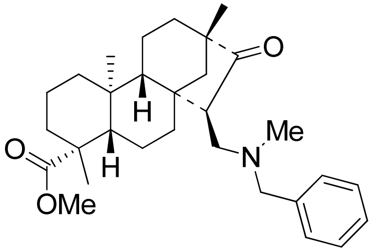

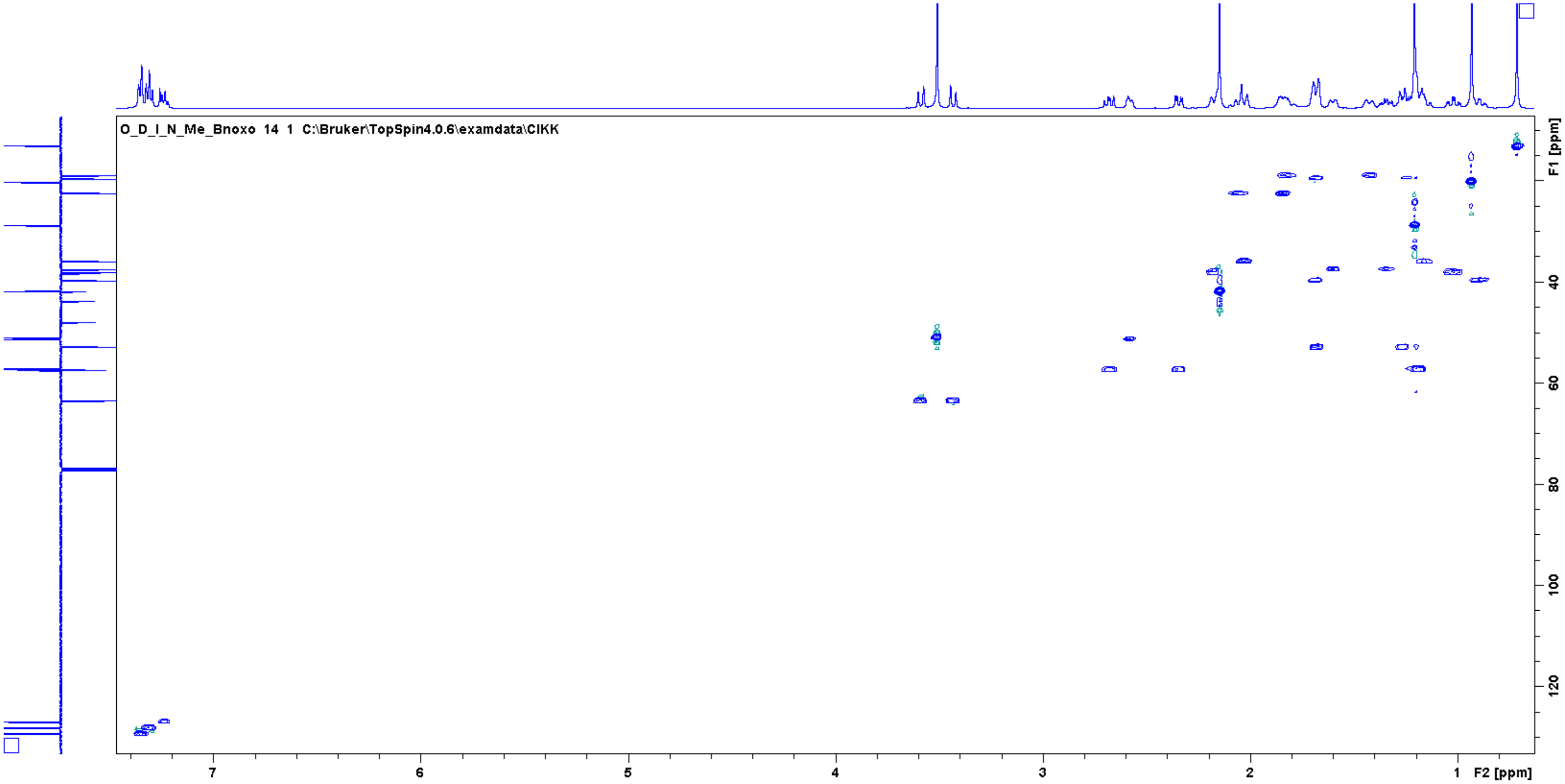

HMBC of compound (4*R*,6a*S*,9*S*,11b*S*)-Methyl 7-((benzyl(methyl)amino)methyl)-4,9,11b-trimethyl-8-oxotetradecahydro-6a,9-methanocyclohepta[a]naphthalene-4-carboxylate (**15**)

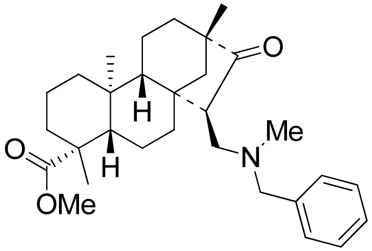

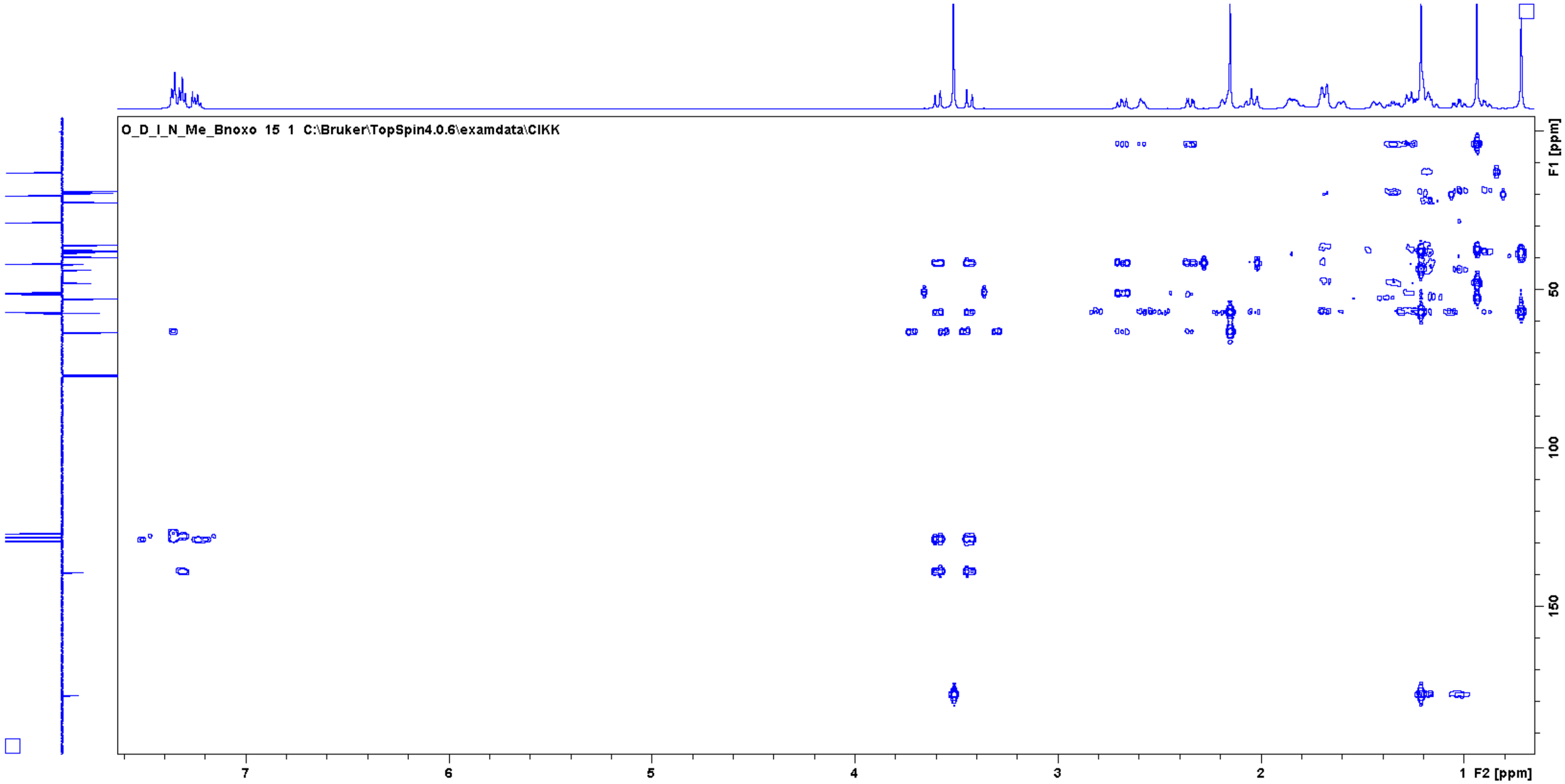

^{1}H-NMR of compound (4*R*,6a*S*,9*S*,11b*S*)-Methyl 4,9,11b-trimethyl-8-oxo-7-(pyrrolidin-1-ylmethyl)tetradecahydro-6a,9-methanocyclohepta[a]naphthalene-4-carboxylate (**16**)

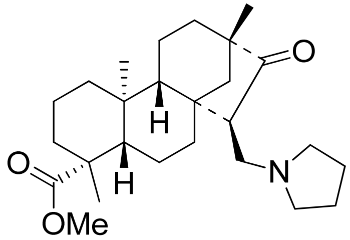

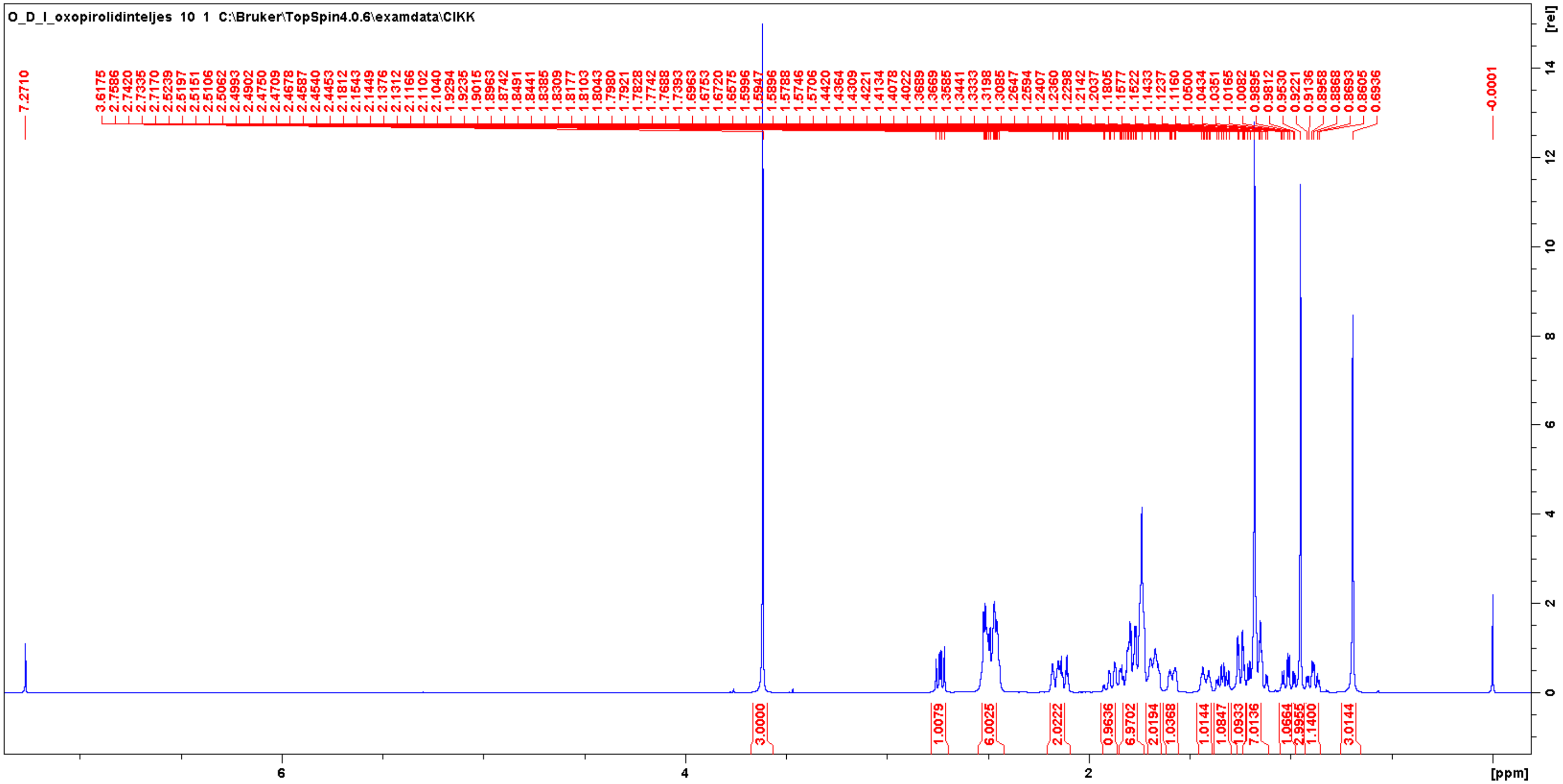

^{13}C-NMR of compound (4*R*,6a*S*,9*S*,11b*S*)-Methyl 4,9,11b-trimethyl-8-oxo-7-(pyrrolidin-1-ylmethyl)tetradecahydro-6a,9-methanocyclohepta[a]naphthalene-4-carboxylate (**16**)

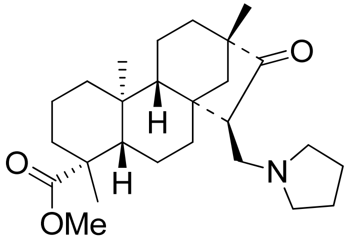

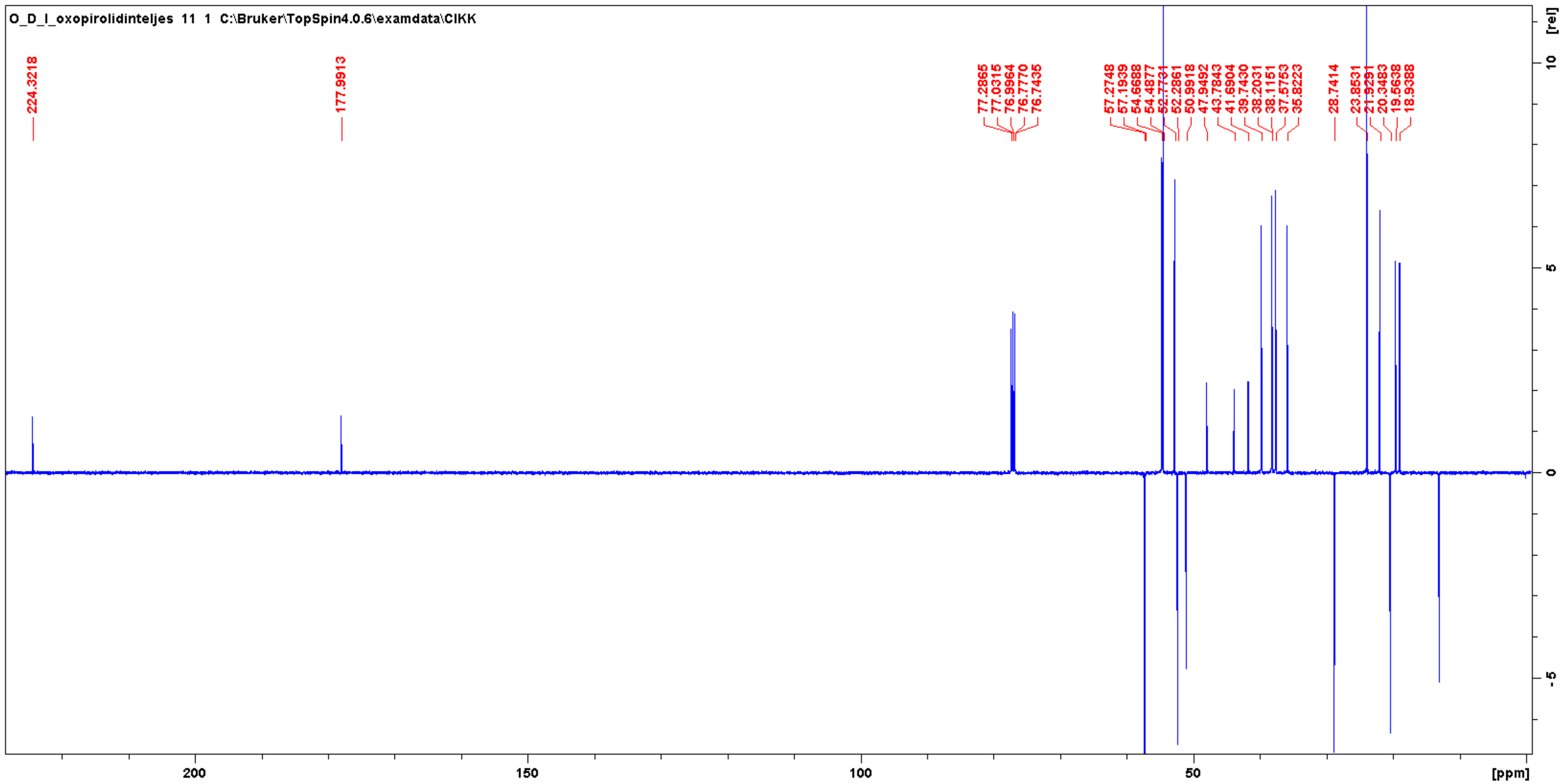

COSY of compound (4*R*,6a*S*,9*S*,11b*S*)-Methyl 4,9,11b-trimethyl-8-oxo-7-(pyrrolidin-1-ylmethyl)tetradecahydro-6a,9-methanocyclohepta[a]naphthalene-4-carboxylate (**16**)

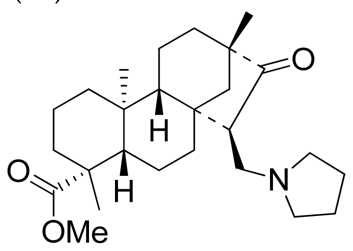

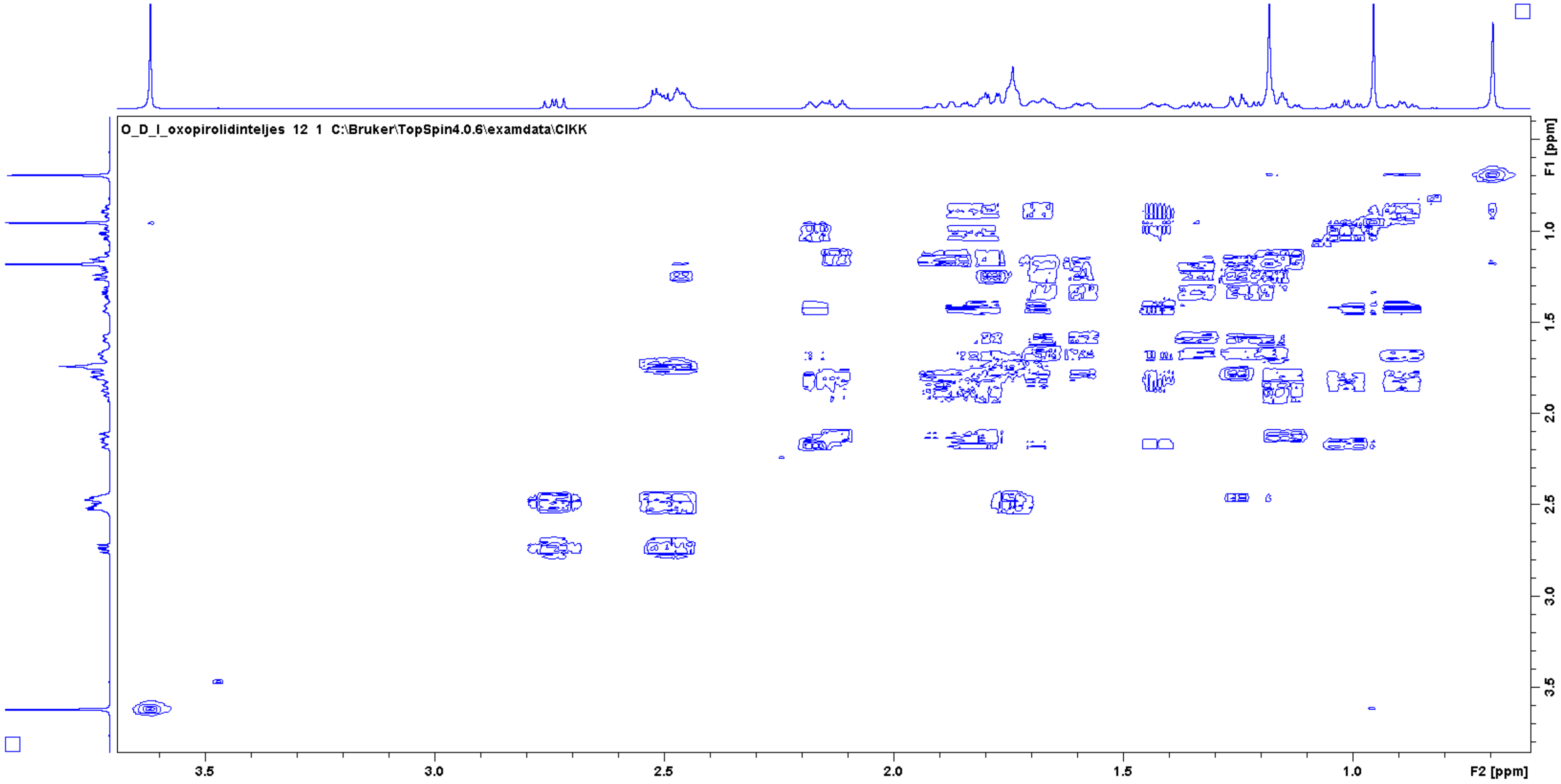

HSQC of compound (4*R*,6a*S*,9*S*,11b*S*)-Methyl 4,9,11b-trimethyl-8-oxo-7-(pyrrolidin-1-ylmethyl)tetradecahydro-6a,9-methanocyclohepta[a]naphthalene-4-carboxylate (**16**)

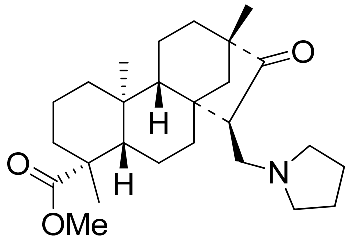

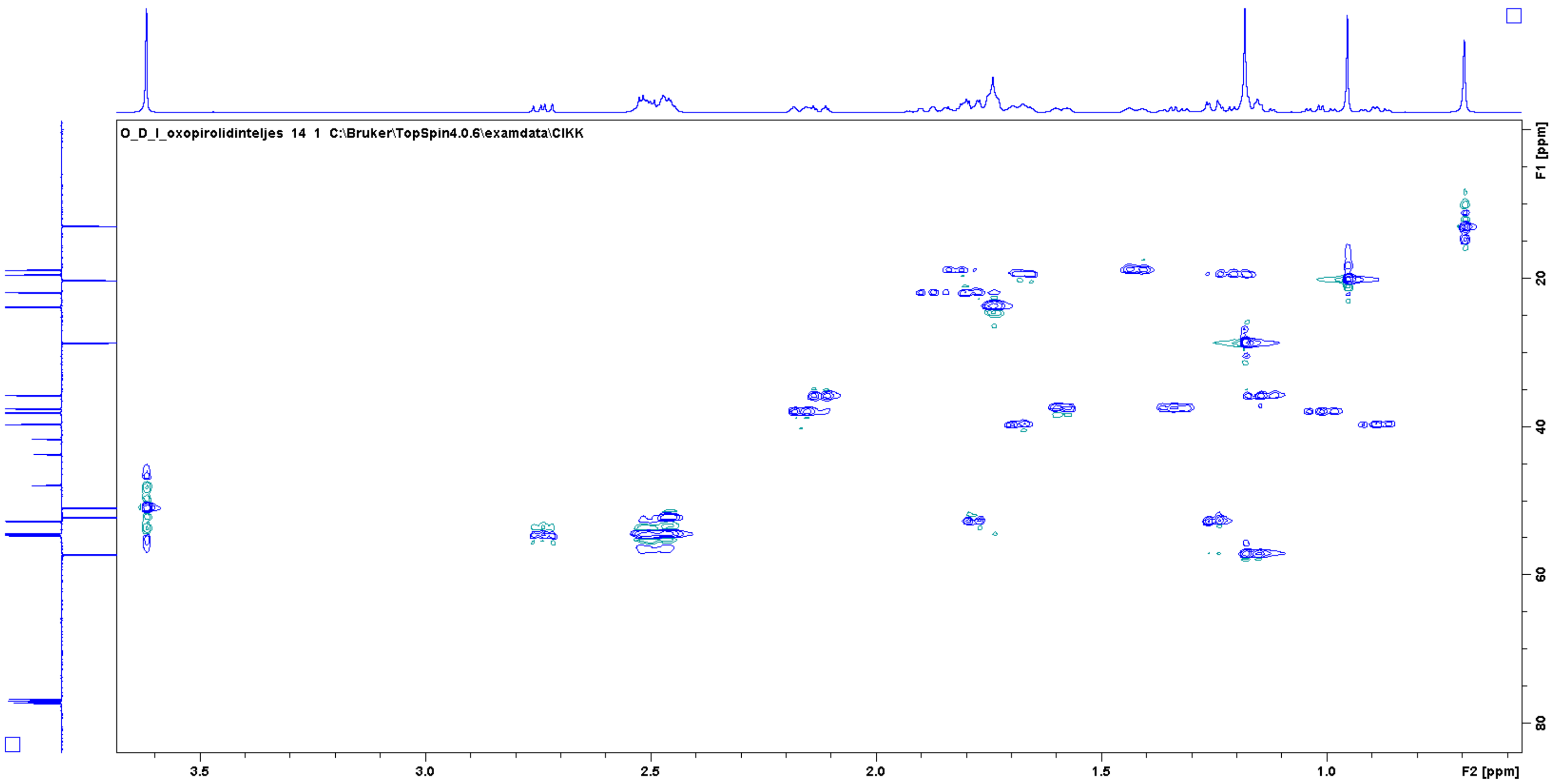

HMBC of compound (4*R*,6a*S*,9*S*,11b*S*)-Methyl 4,9,11b-trimethyl-8-oxo-7-(pyrrolidin-1-ylmethyl)tetradecahydro-6a,9-methanocyclohepta[a]naphthalene-4-carboxylate (**16**)

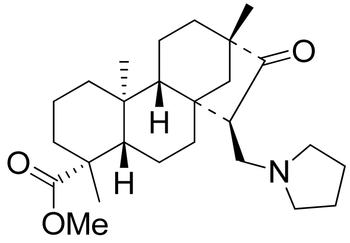

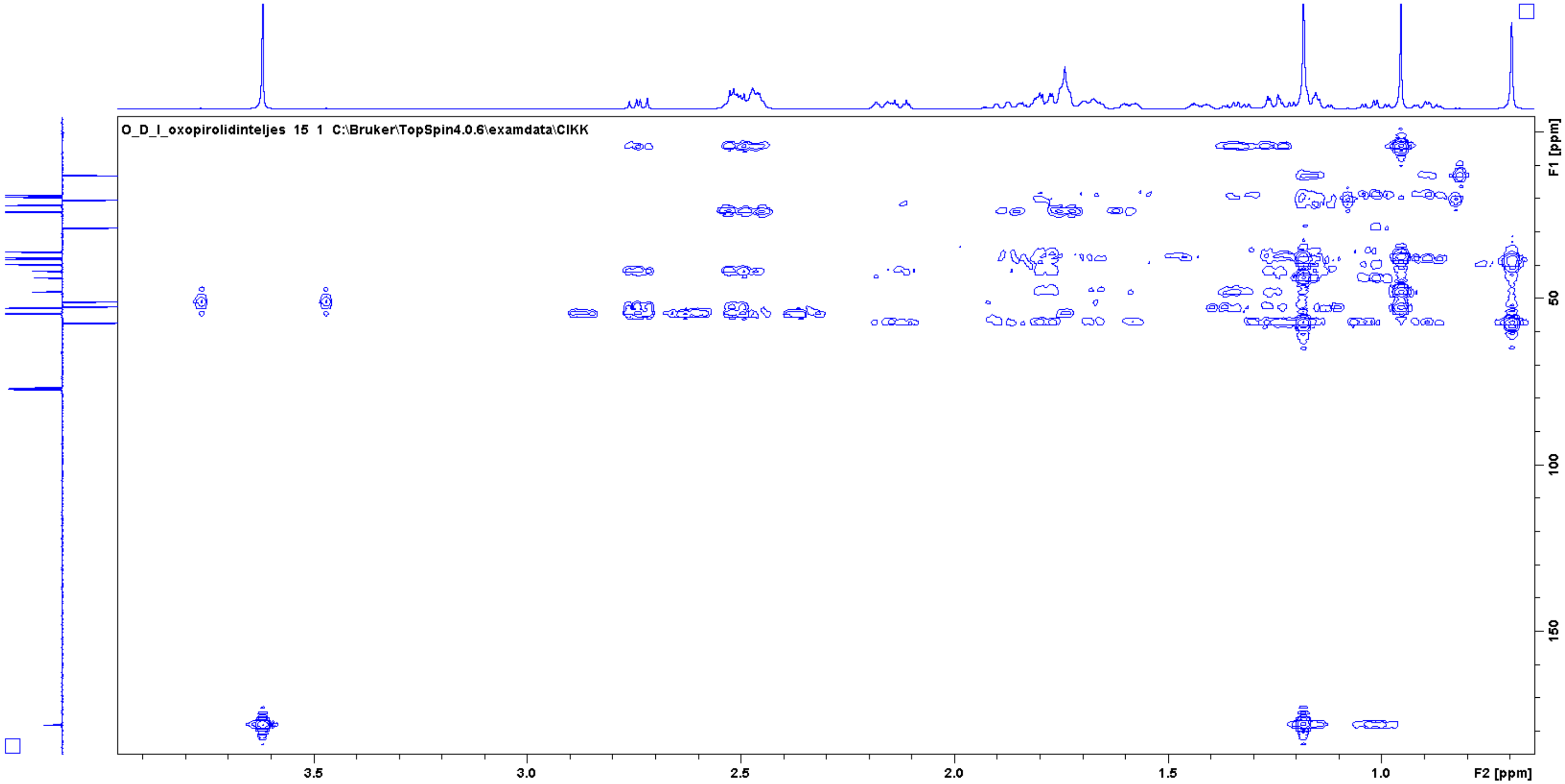

^{1}H-NMR of compound (4*R*,6a*S*,9*S*,11b*S*)-Methyl 7-((dimethylamino)methyl)-4,9,11b-trimethyl-8-oxotetradecahydro-6a,9-methanocyclohepta[a]naphthalene-4-carboxylate (**17**)

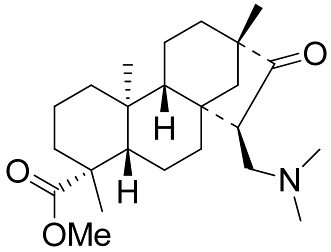

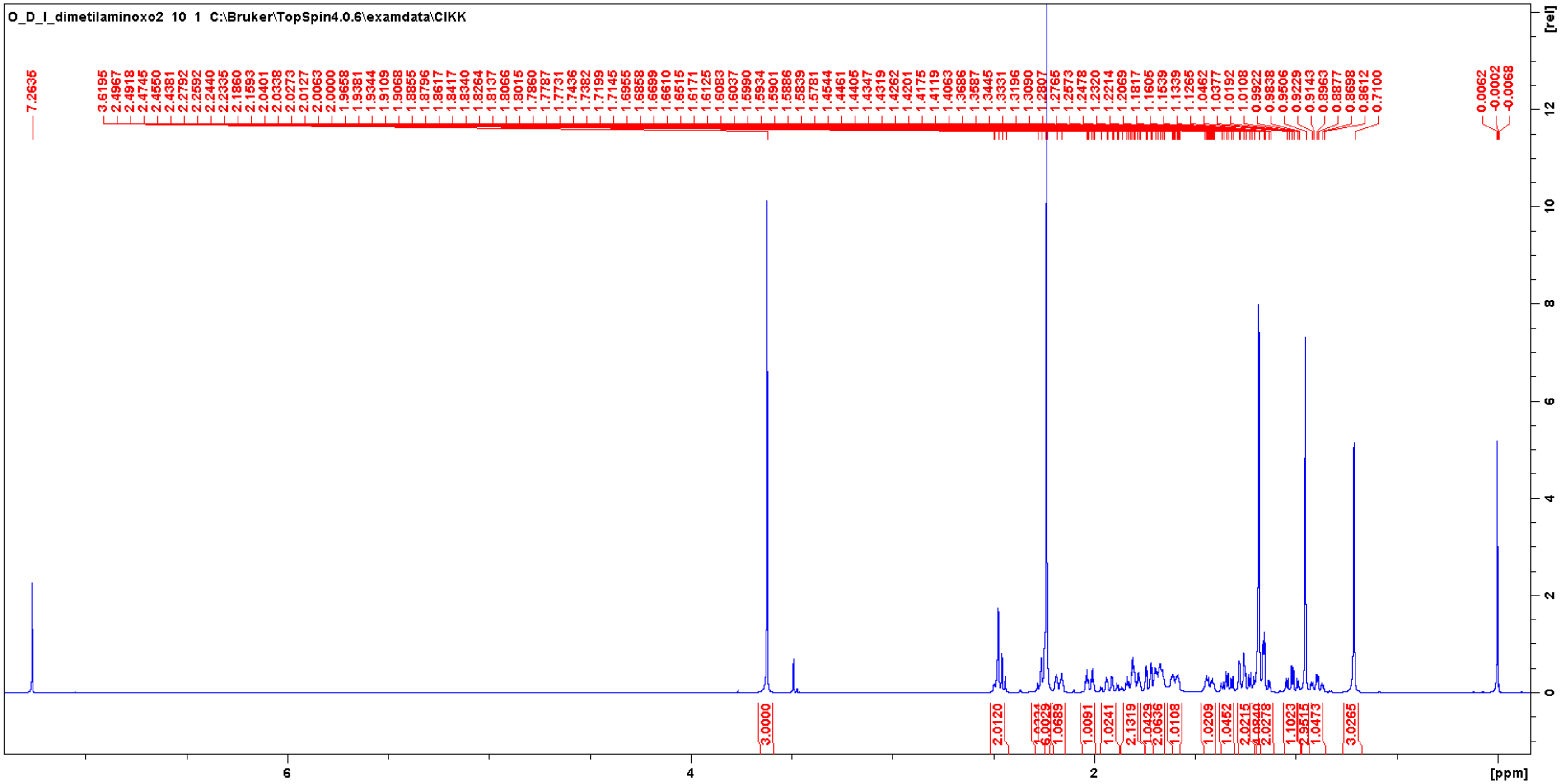

^{13}C-NMR of compound (4*R*,6a*S*,9*S*,11b*S*)-Methyl 7-((dimethylamino)methyl)-4,9,11b-trimethyl-8-oxotetradecahydro-6a,9-methanocyclohepta[a]naphthalene-4-carboxylate (**17**)

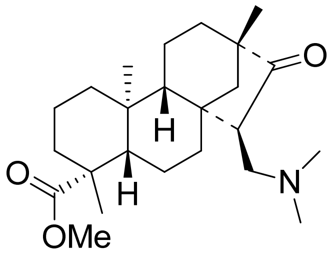

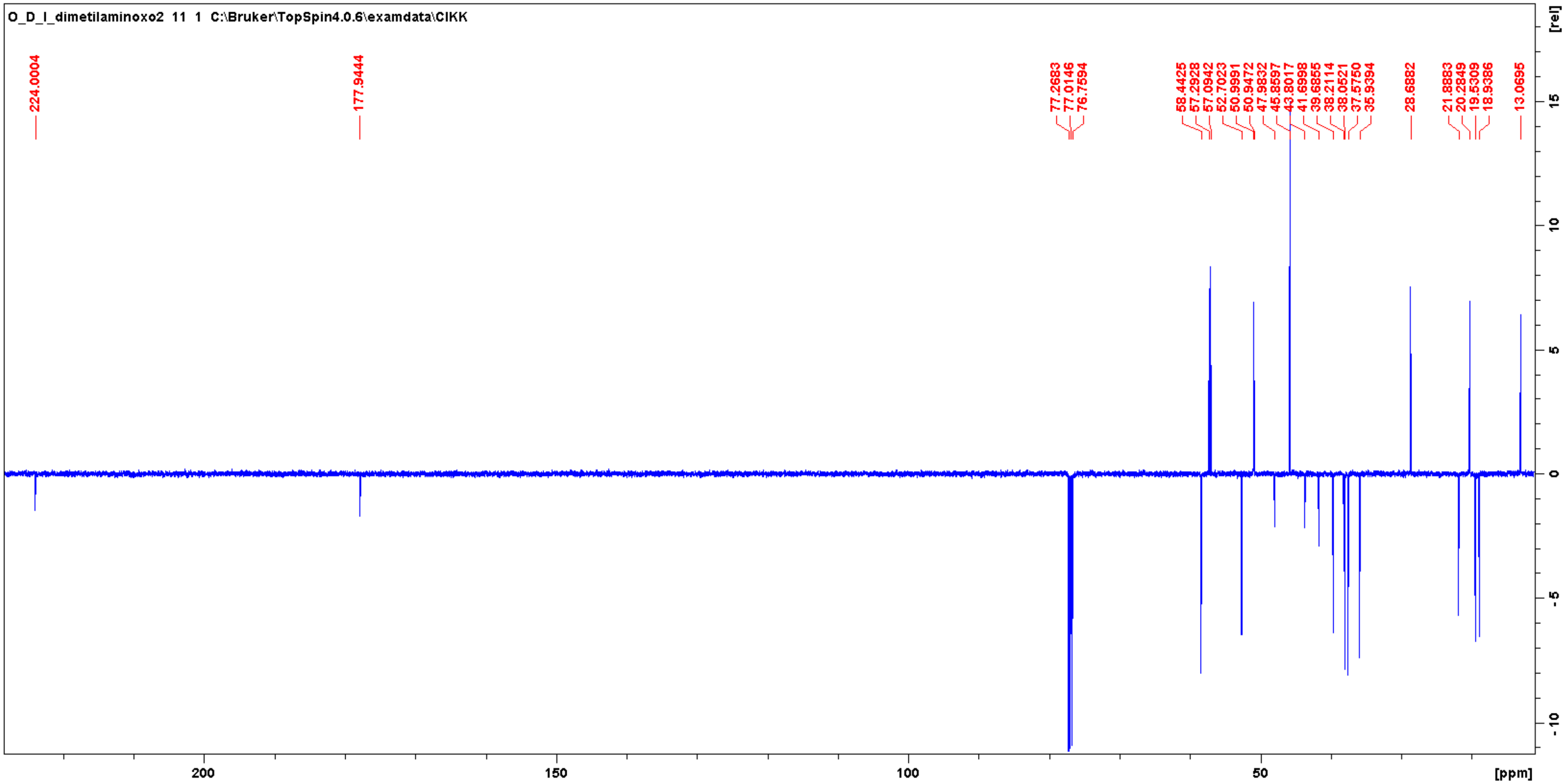

COSY of compound (4*R*,6a*S*,9*S*,11b*S*)-Methyl 7-((dimethylamino)methyl)-4,9,11b-trimethyl-8-oxotetradecahydro-6a,9-methanocyclohepta[a]naphthalene-4-carboxylate (**17**)

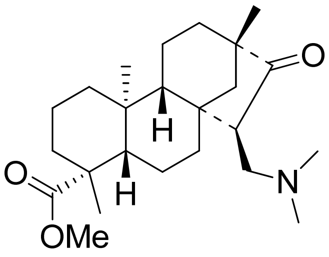

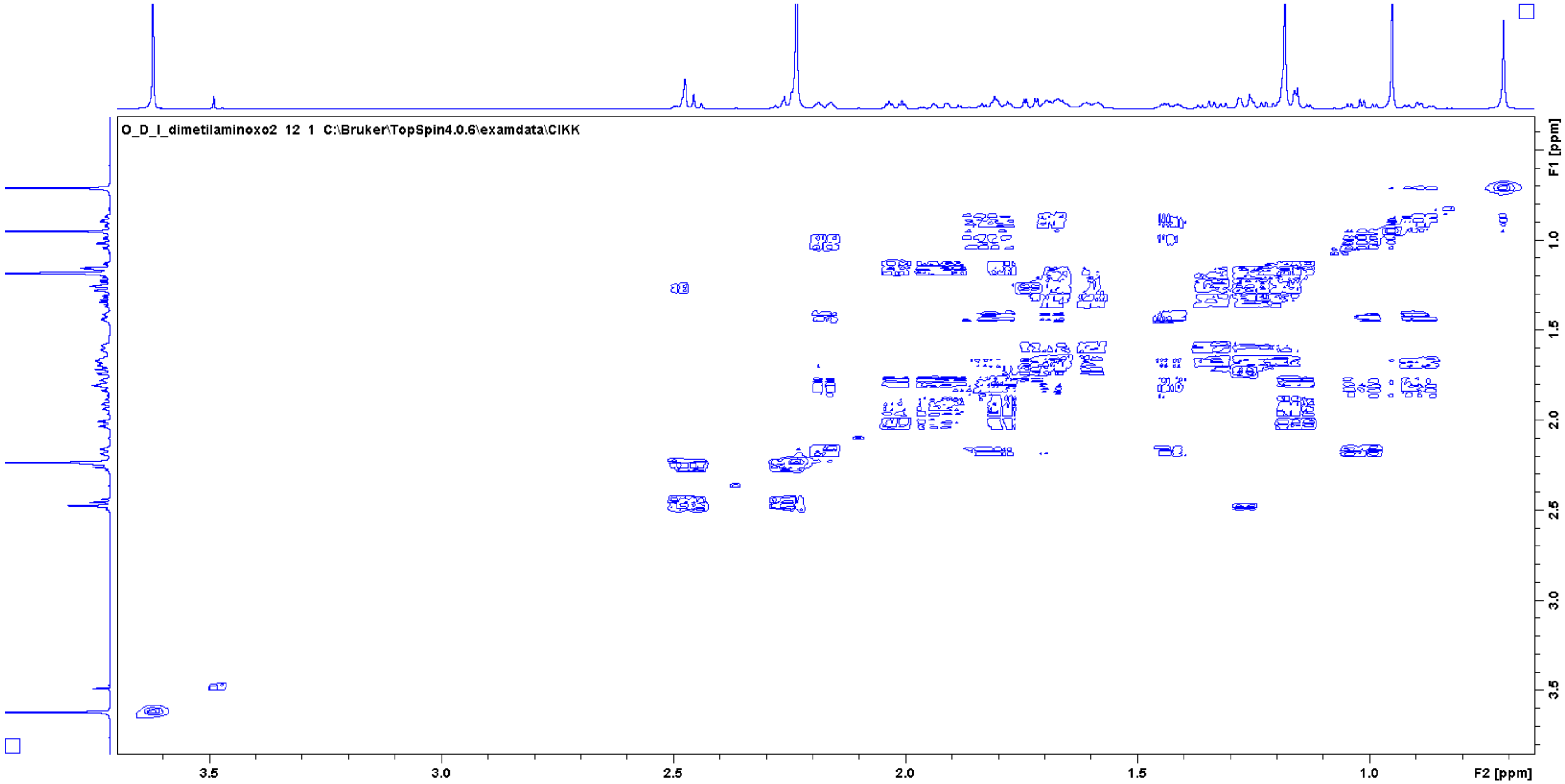

NOESY of compound (4*R*,6a*S*,9*S*,11b*S*)-Methyl 7-((dimethylamino)methyl)-4,9,11b-trimethyl-8-oxotetradecahydro-6a,9-methanocyclohepta[a]naphthalene-4-carboxylate (**17**)

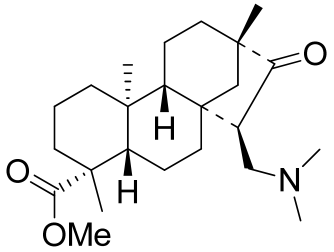

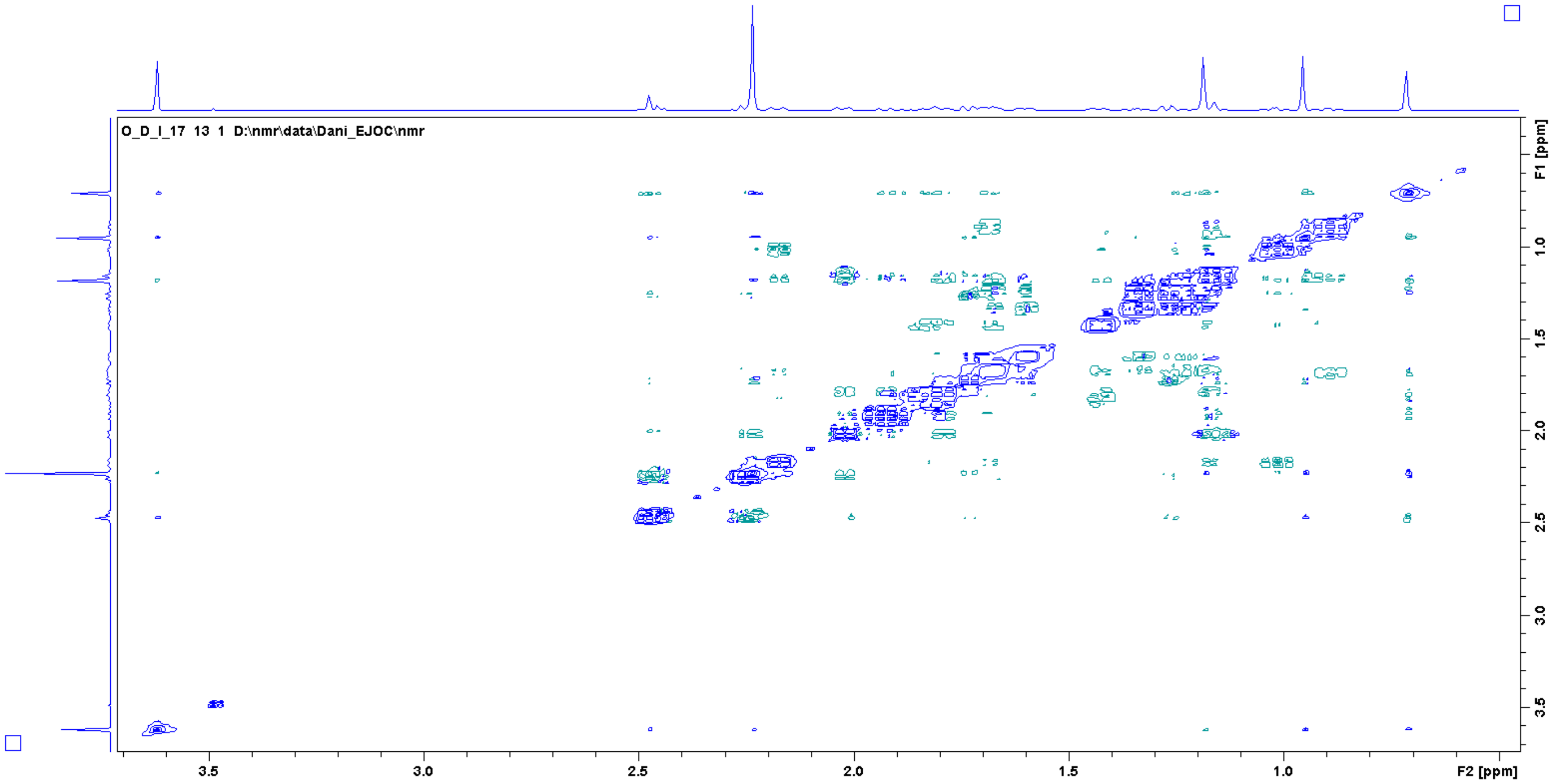

HSQC of compound (4*R*,6a*S*,9*S*,11b*S*)-Methyl 7-((dimethylamino)methyl)-4,9,11b-trimethyl-8-oxotetradecahydro-6a,9-methanocyclohepta[a]naphthalene-4-carboxylate (**17**)

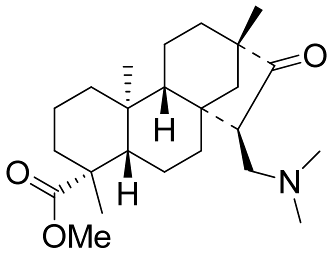

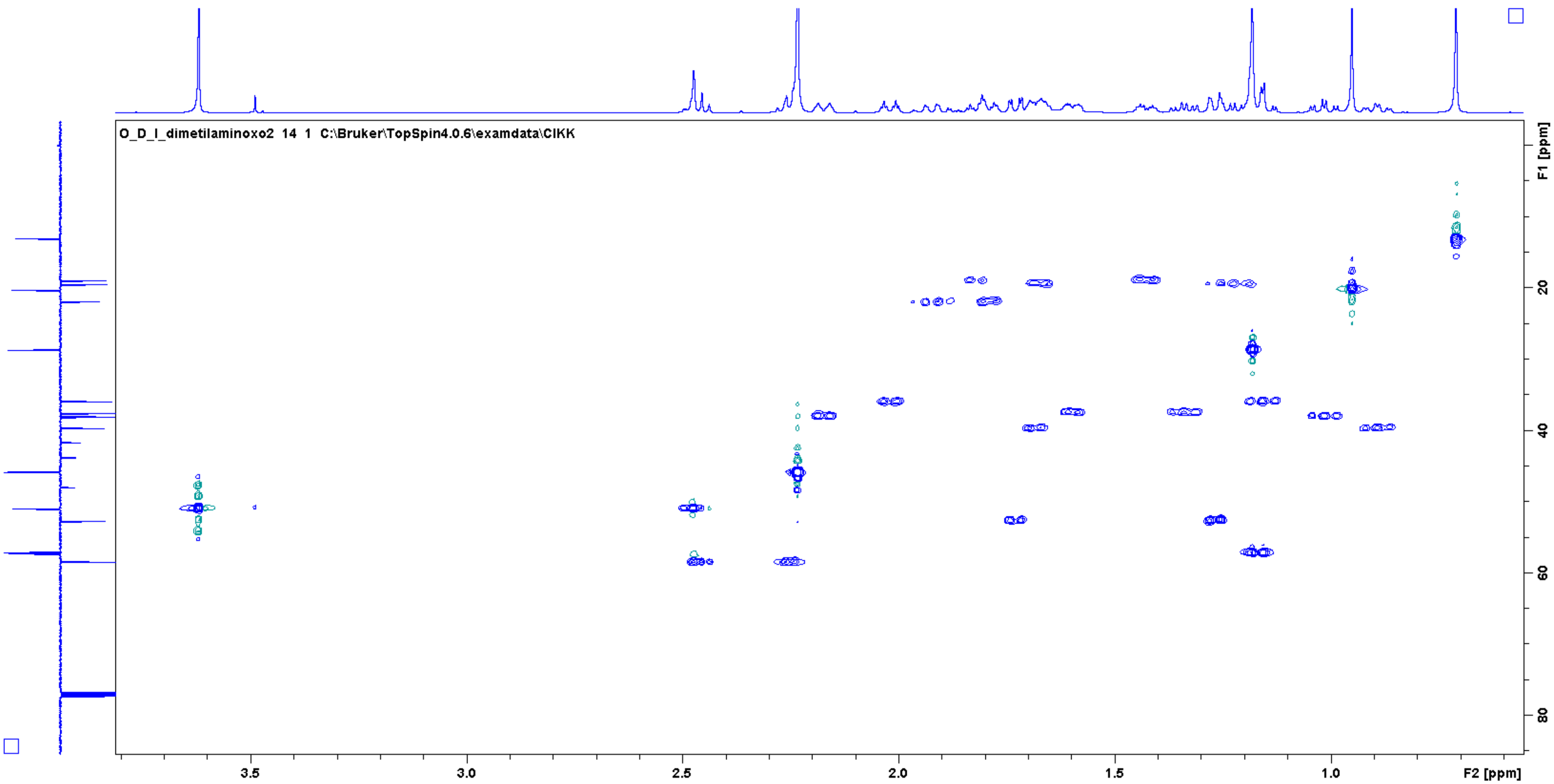

HMBC of compound (4*R*,6a*S*,9*S*,11b*S*)-Methyl 7-((dimethylamino)methyl)-4,9,11b-trimethyl-8-oxotetradecahydro-6a,9-methanocyclohepta[a]naphthalene-4-carboxylate (**17**)

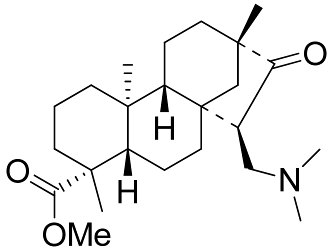

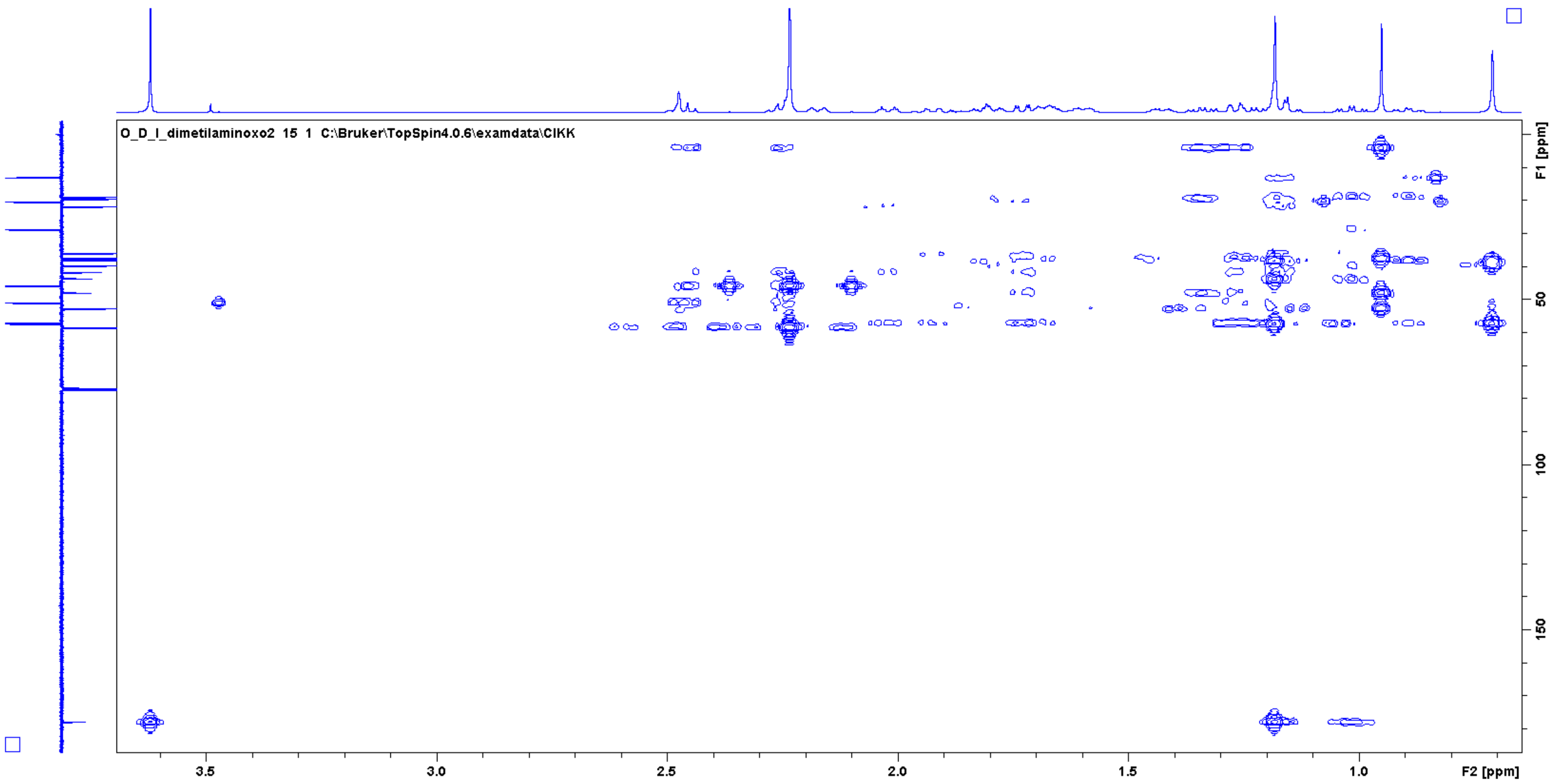

^{1}H-NMR of compound (4*R*,6a*S*,9*S*,11b*S*)-Methyl 7-((diethylamino)methyl)-4,9,11b-trimethyl-8-oxotetradecahydro-6a,9-methanocyclohepta[a]naphthalene-4-carboxylate (**18**)

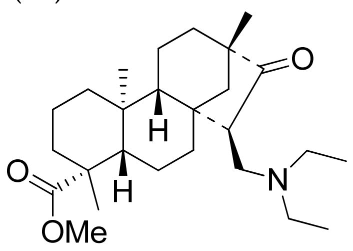

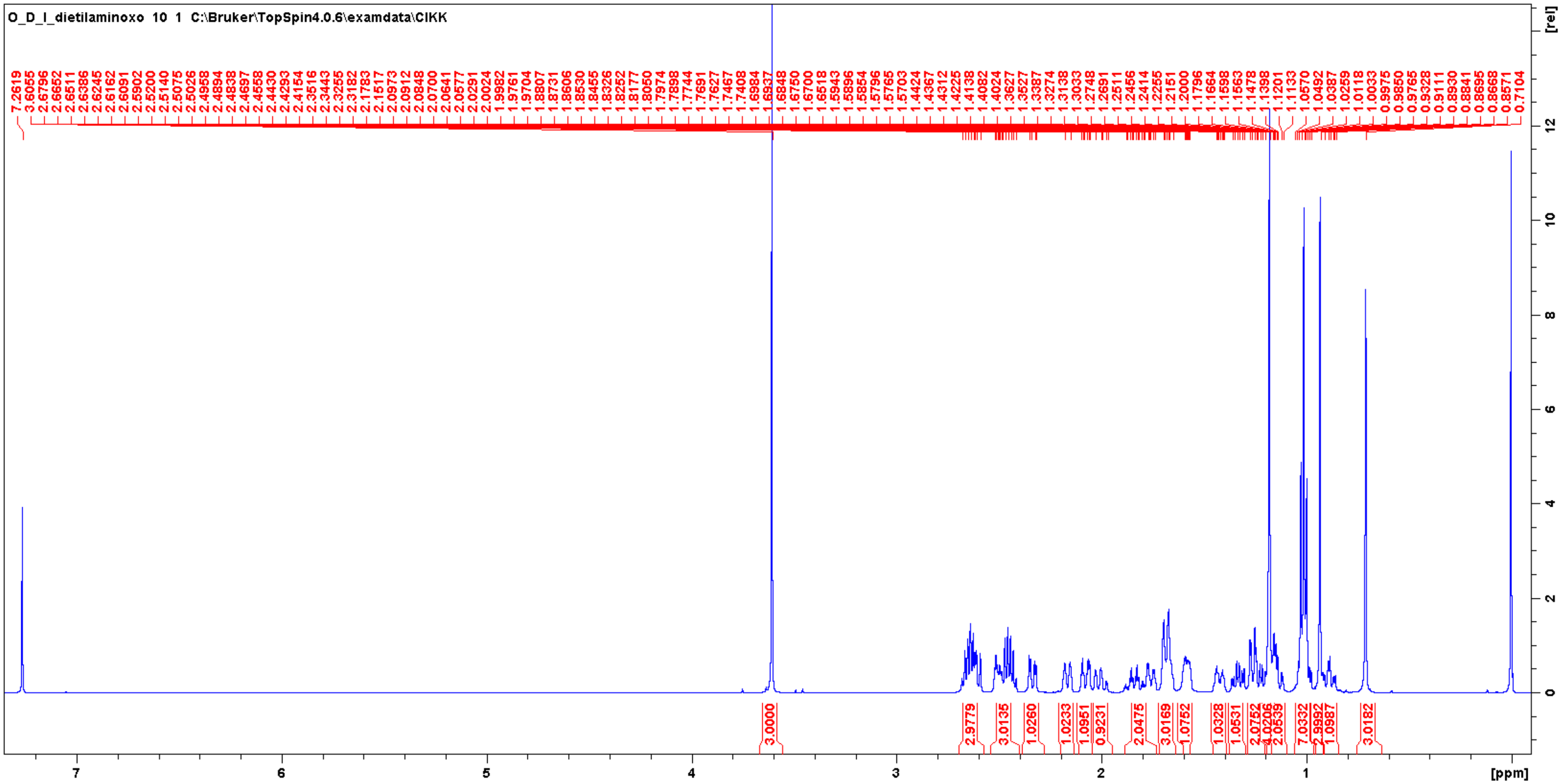

^{13}C-NMR of compound (4*R*,6a*S*,9*S*,11b*S*)-Methyl 7-((diethylamino)methyl)-4,9,11b-trimethyl-8-oxotetradecahydro-6a,9-methanocyclohepta[a]naphthalene-4-carboxylate (**18**)

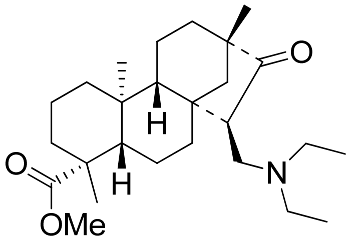

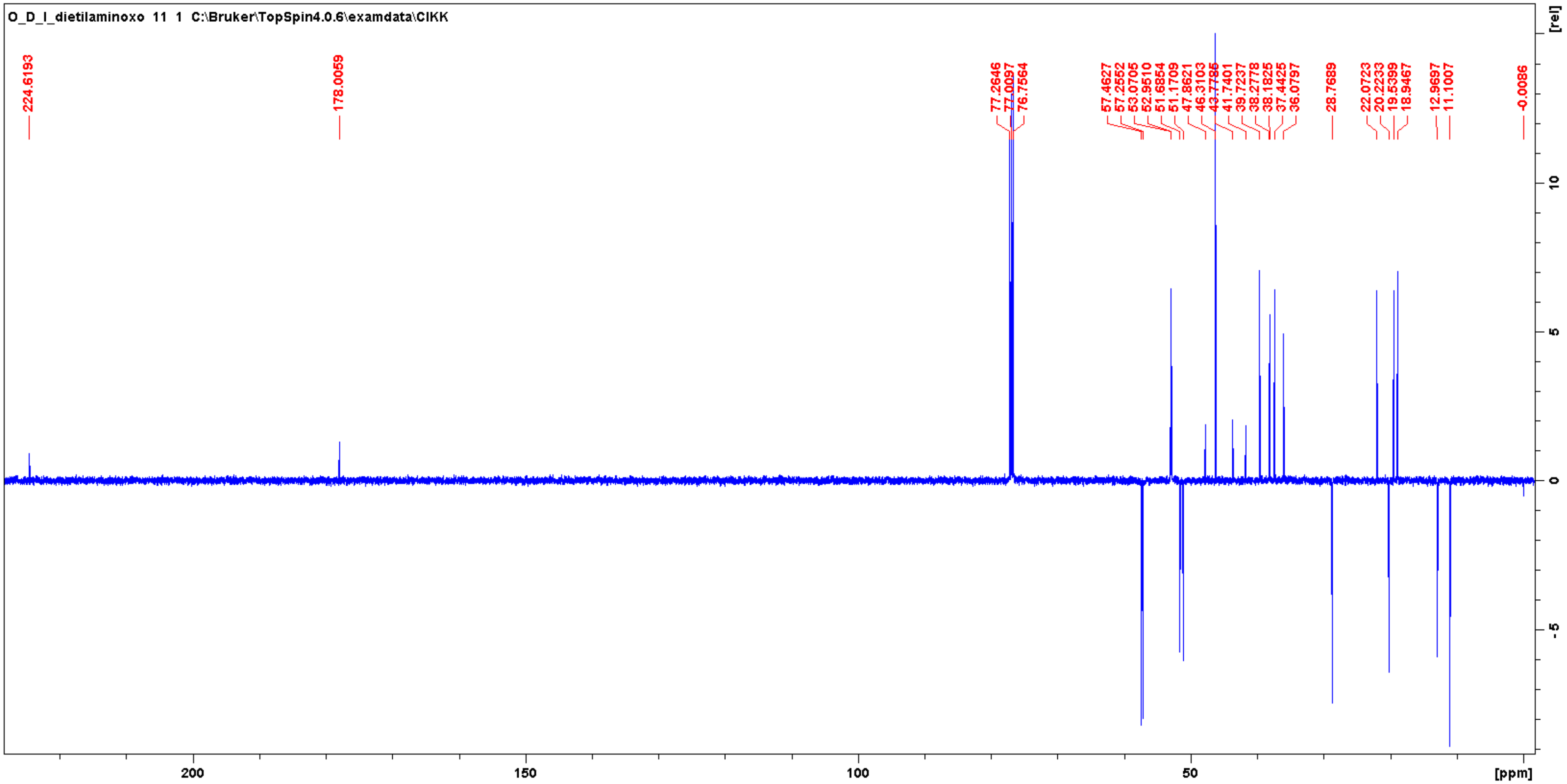

COSY of compound (4*R*,6a*S*,9*S*,11b*S*)-Methyl 7-((diethylamino)methyl)-4,9,11b-trimethyl-8-oxotetradecahydro-6a,9-methanocyclohepta[a]naphthalene-4-carboxylate (**18**)

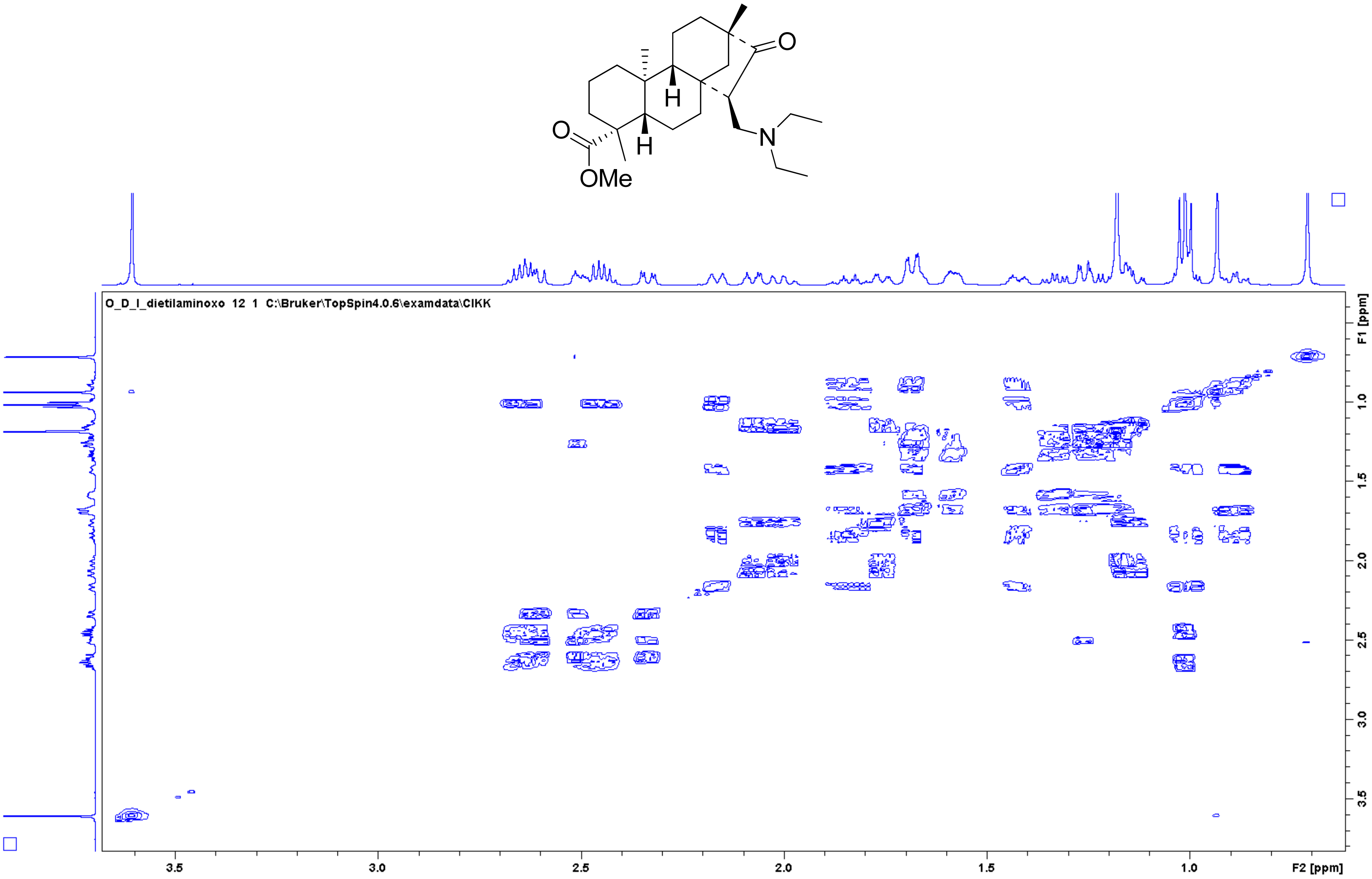

NOESY of compound (4*R*,6a*S*,9*S*,11b*S*)-Methyl 7-((diethylamino)methyl)-4,9,11b-trimethyl-8-oxotetradecahydro-6a,9-methanocyclohepta[a]naphthalene-4-carboxylate (**18**)

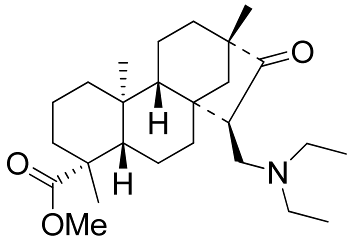

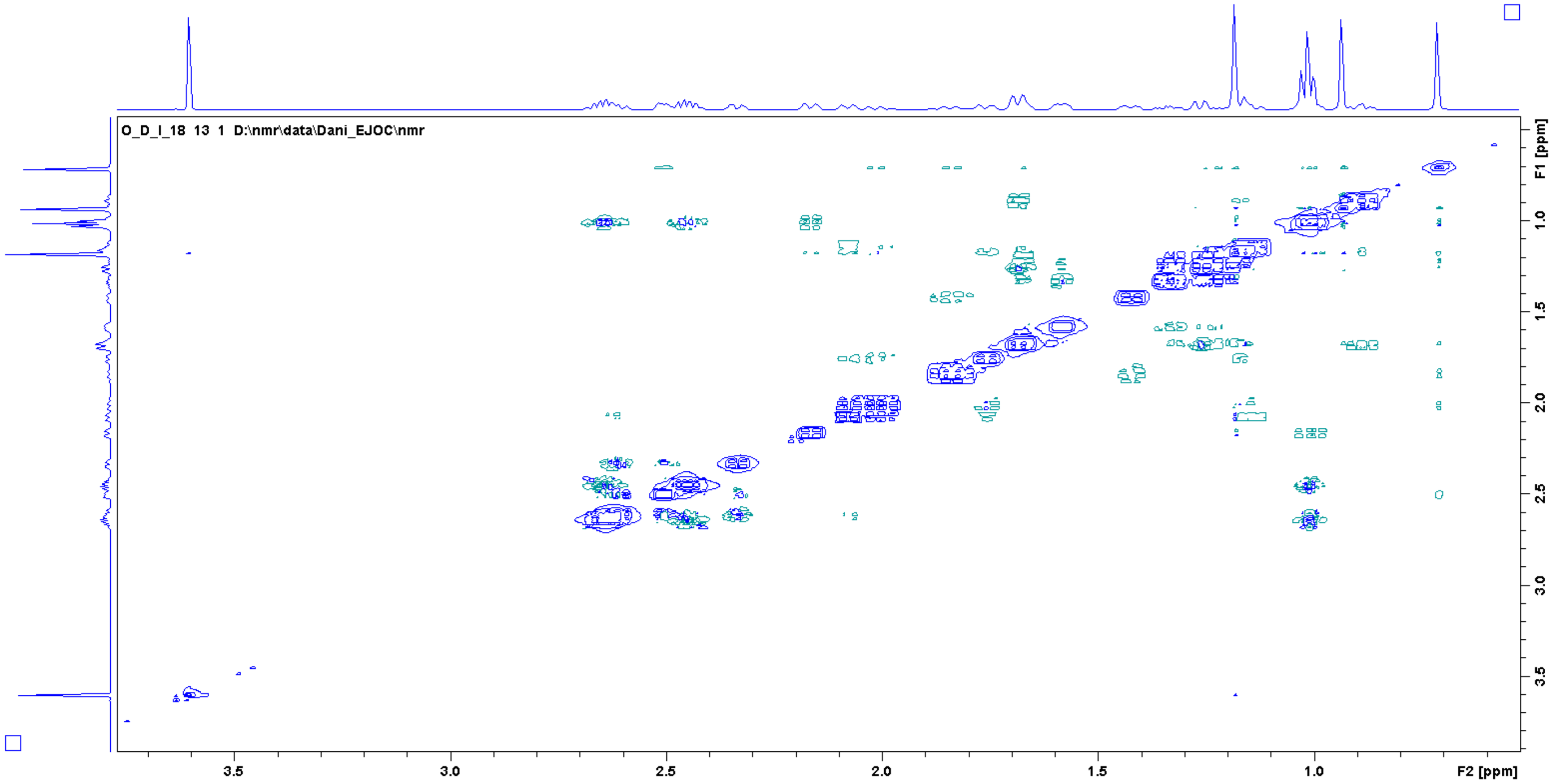

HSQC of compound (4*R*,6a*S*,9*S*,11b*S*)-Methyl 7-((diethylamino)methyl)-4,9,11b-trimethyl-8-oxotetradecahydro-6a,9-methanocyclohepta[a]naphthalene-4-carboxylate (**18**)

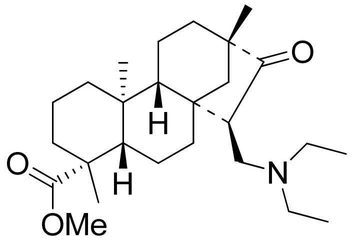

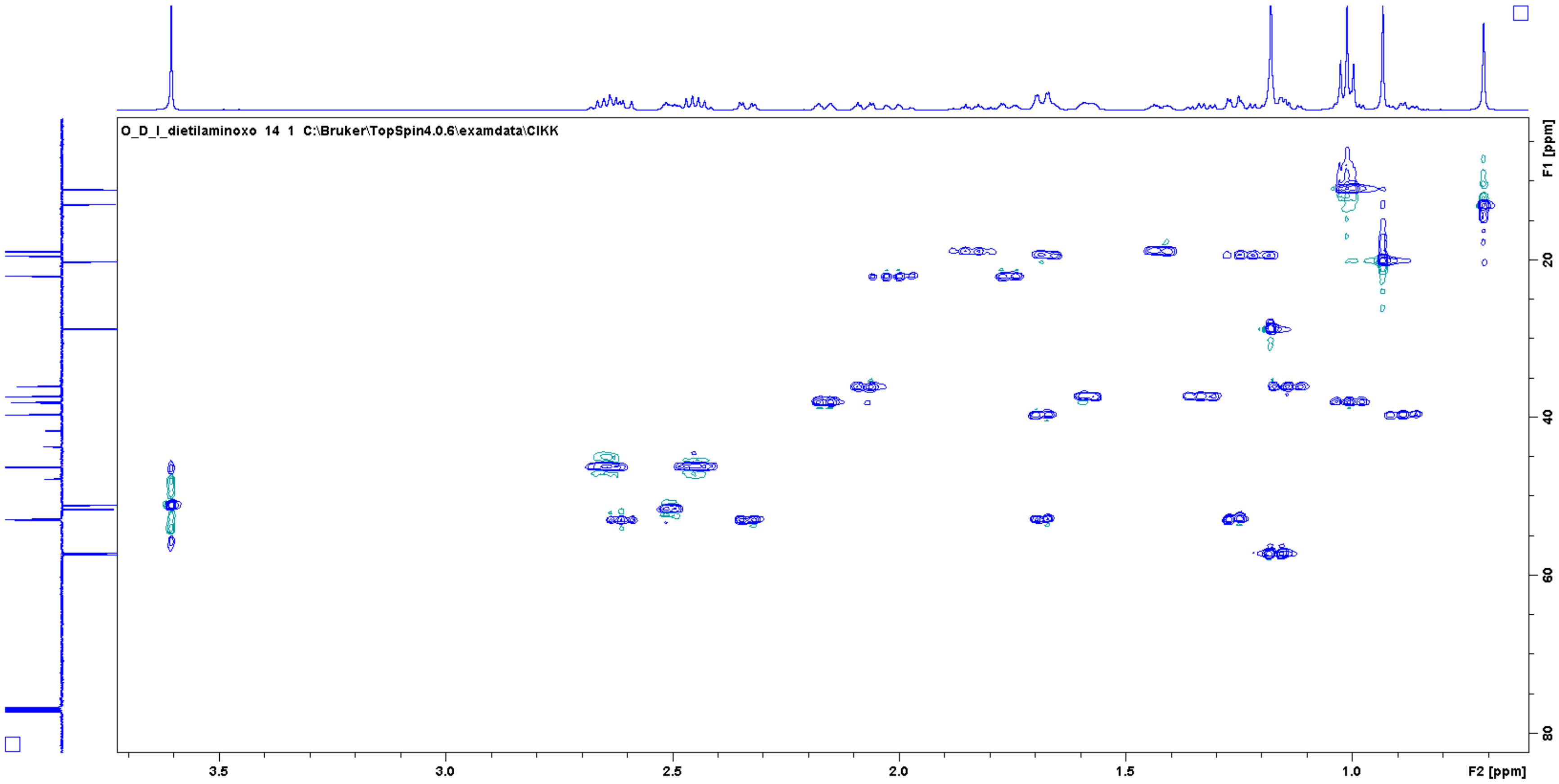

HMBC of compound (4*R*,6a*S*,9*S*,11b*S*)-Methyl 7-((diethylamino)methyl)-4,9,11b-trimethyl-8-oxotetradecahydro-6a,9-methanocyclohepta[a]naphthalene-4-carboxylate (**18**)

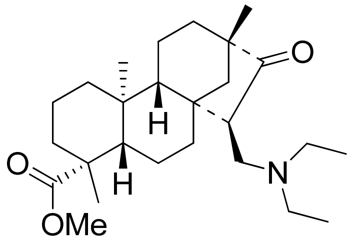

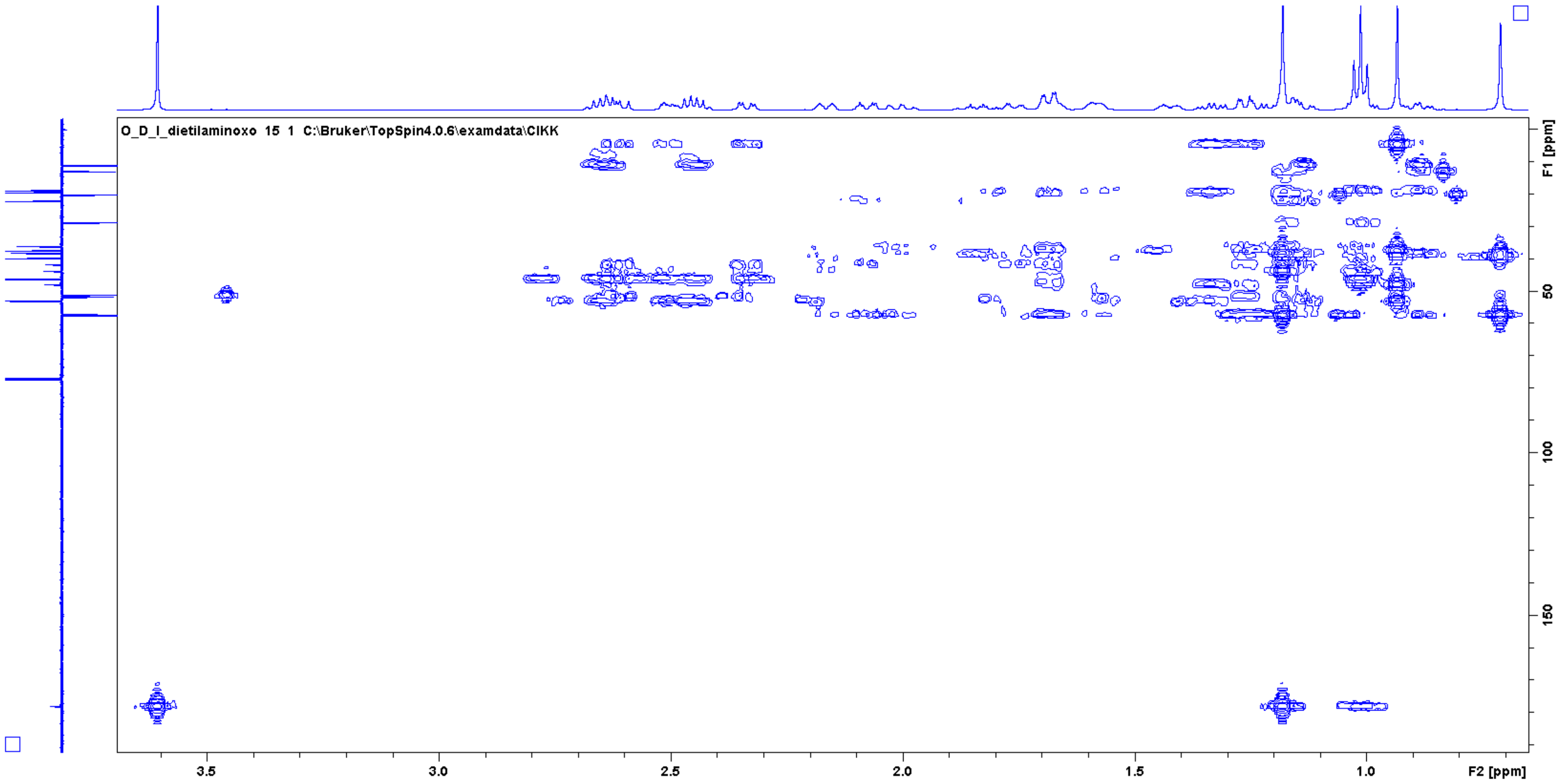

^{1}H-NMR of compound (4*R*,6a*S*,9*S*,11b*S*)-Methyl 8-hydroxy-4,9,11b-trimethyl-7-(morpholinomethyl)tetradecahydro-6a,9 methanocyclohepta[a]naphthalene-4-carboxylate (**19a**)

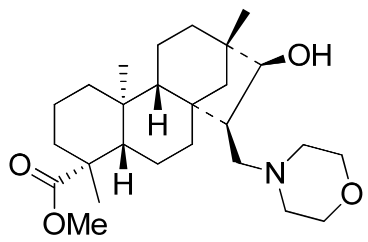

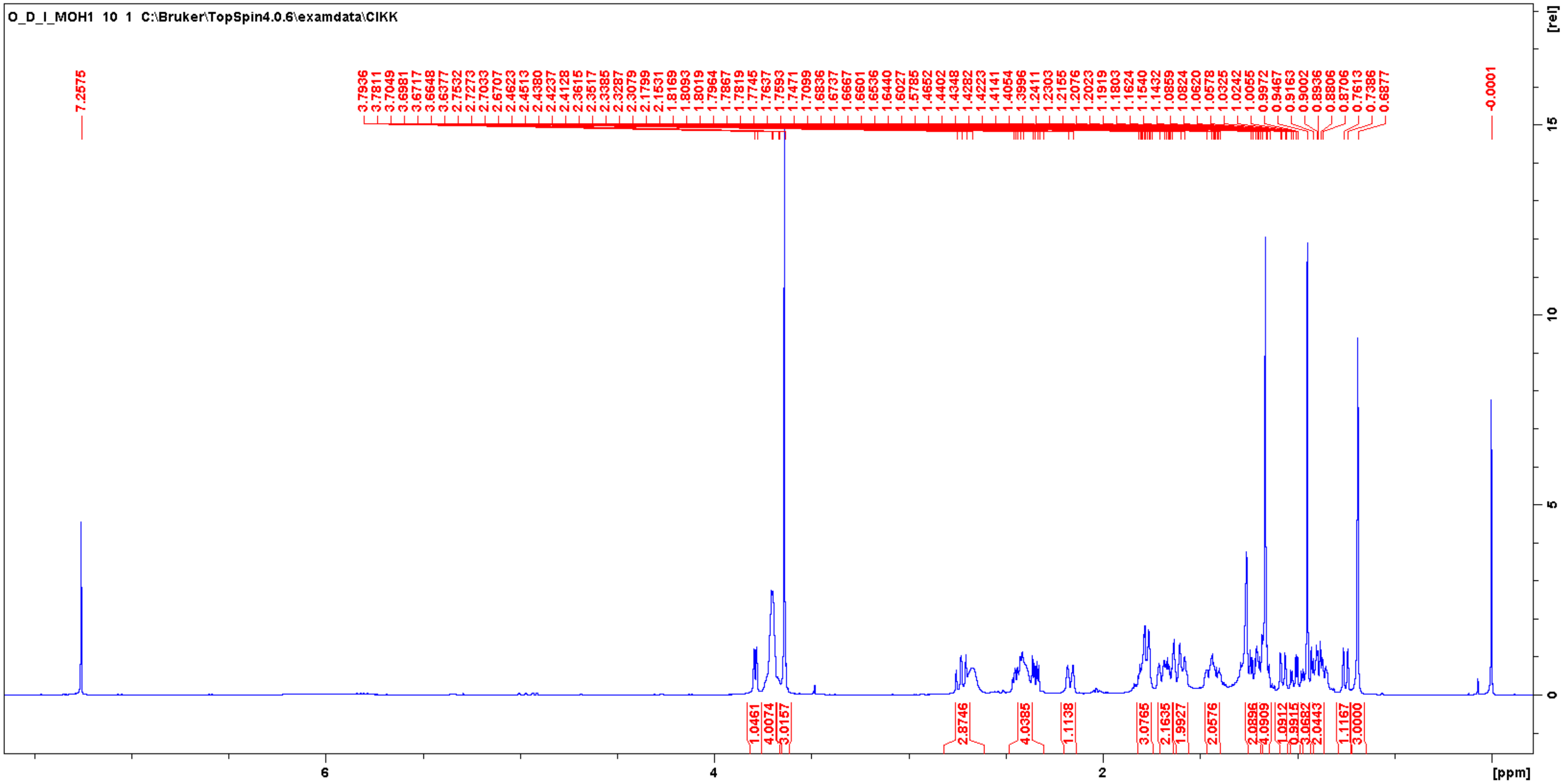

^{13}C-NMR of compound (4*R*,6a*S*,9*S*,11b*S*)-Methyl 8-hydroxy-4,9,11b-trimethyl-7-(morpholinomethyl)tetradecahydro-6a,9 methanocyclohepta[a]naphthalene-4-carboxylate (**19a**)

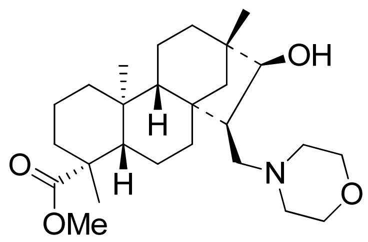

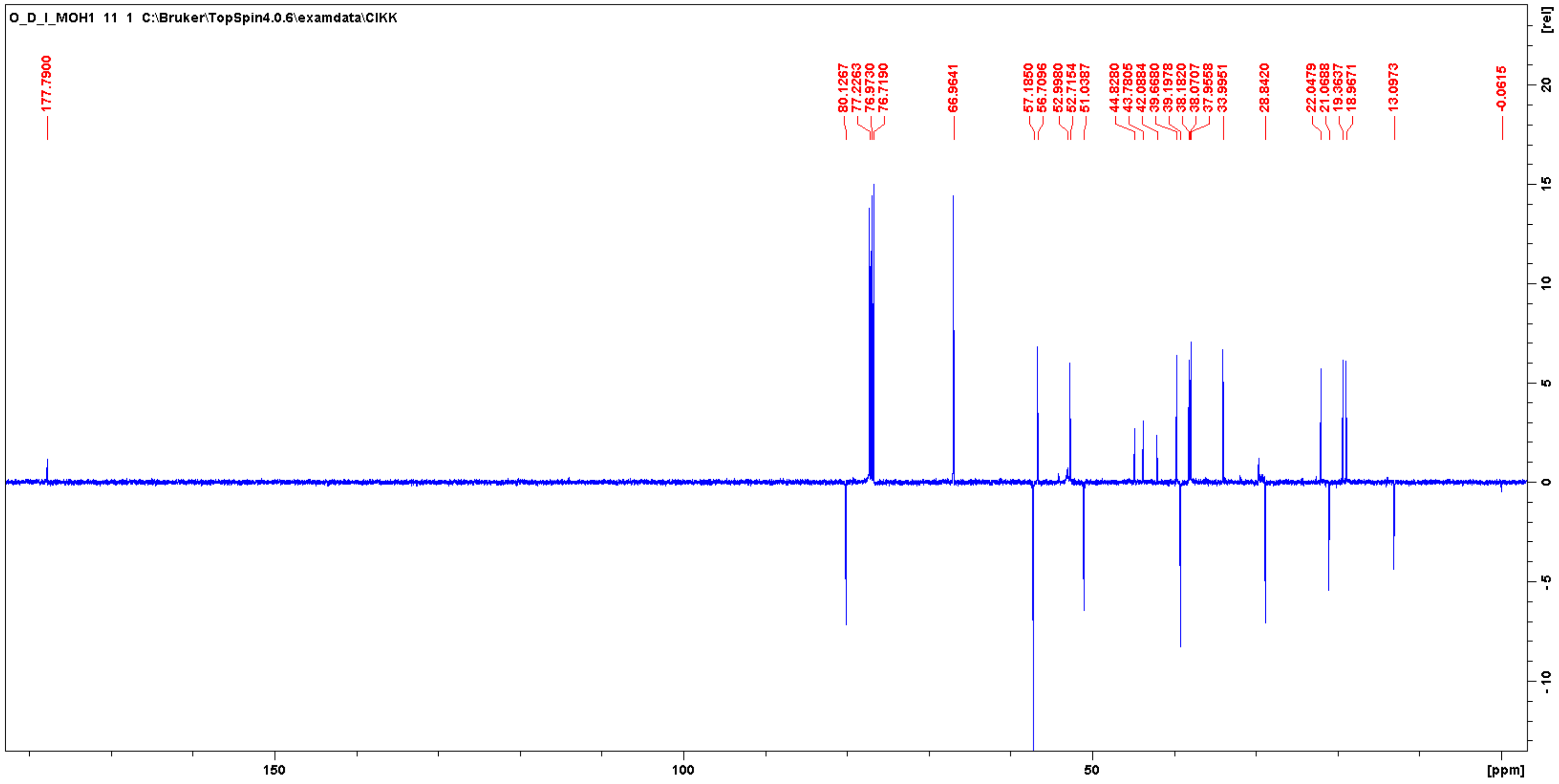

COSY of compound (4*R*,6a*S*,9*S*,11b*S*)-Methyl 8-hydroxy-4,9,11b-trimethyl-7-(morpholinomethyl)tetradecahydro-6a,9 methanocyclohepta[a]naphthalene-4-carboxylate (**19a**)

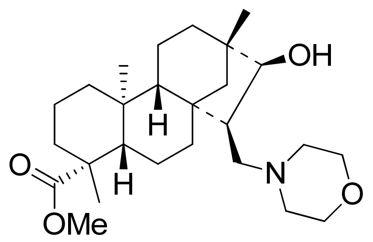

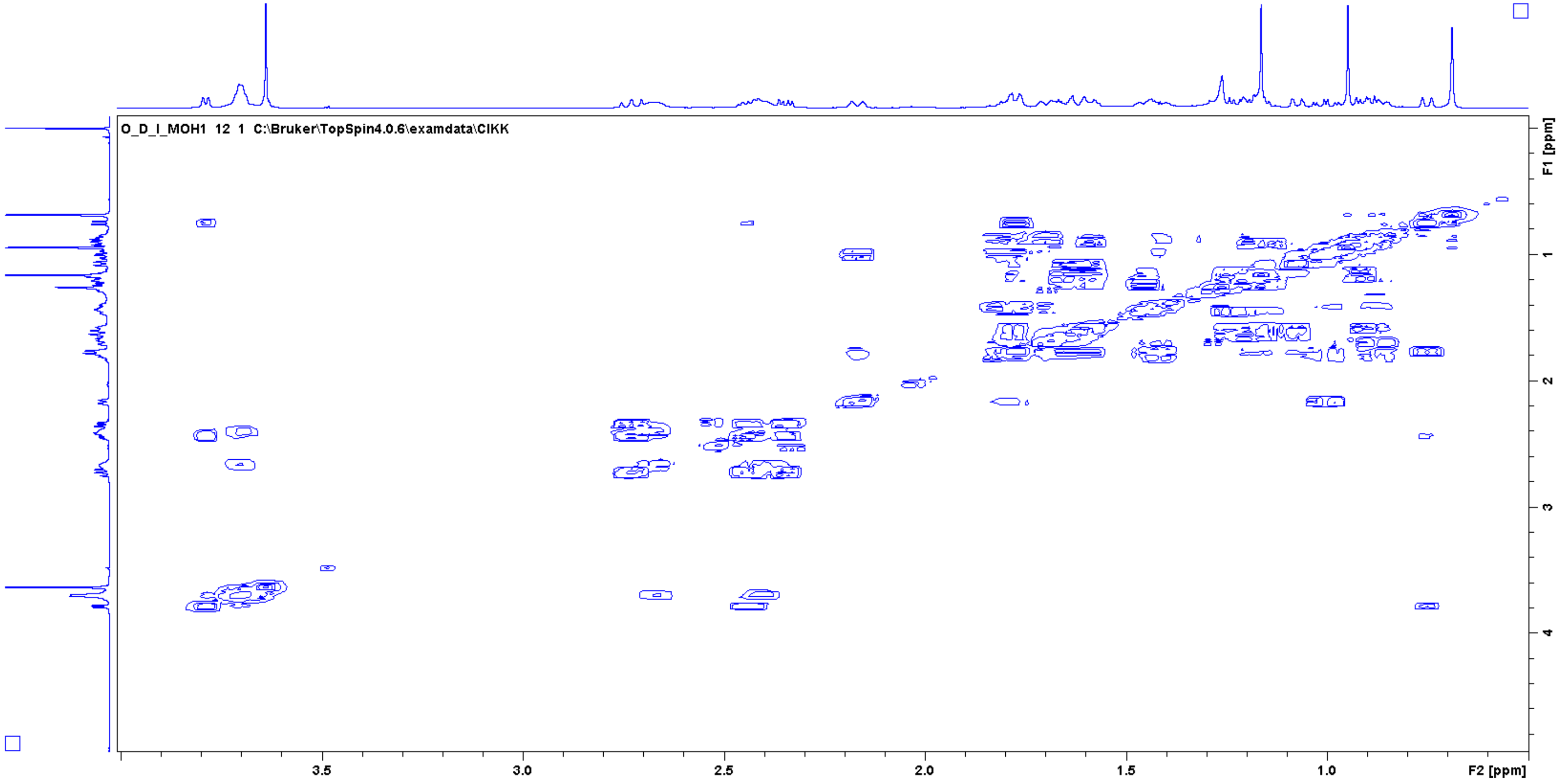

HSQC of compound (4*R*,6a*S*,9*S*,11b*S*)-Methyl 8-hydroxy-4,9,11b-trimethyl-7-(morpholinomethyl)tetradecahydro-6a,9 methanocyclohepta[a]naphthalene-4-carboxylate (**19a**)

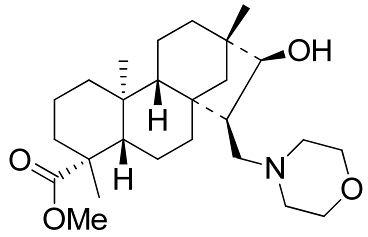

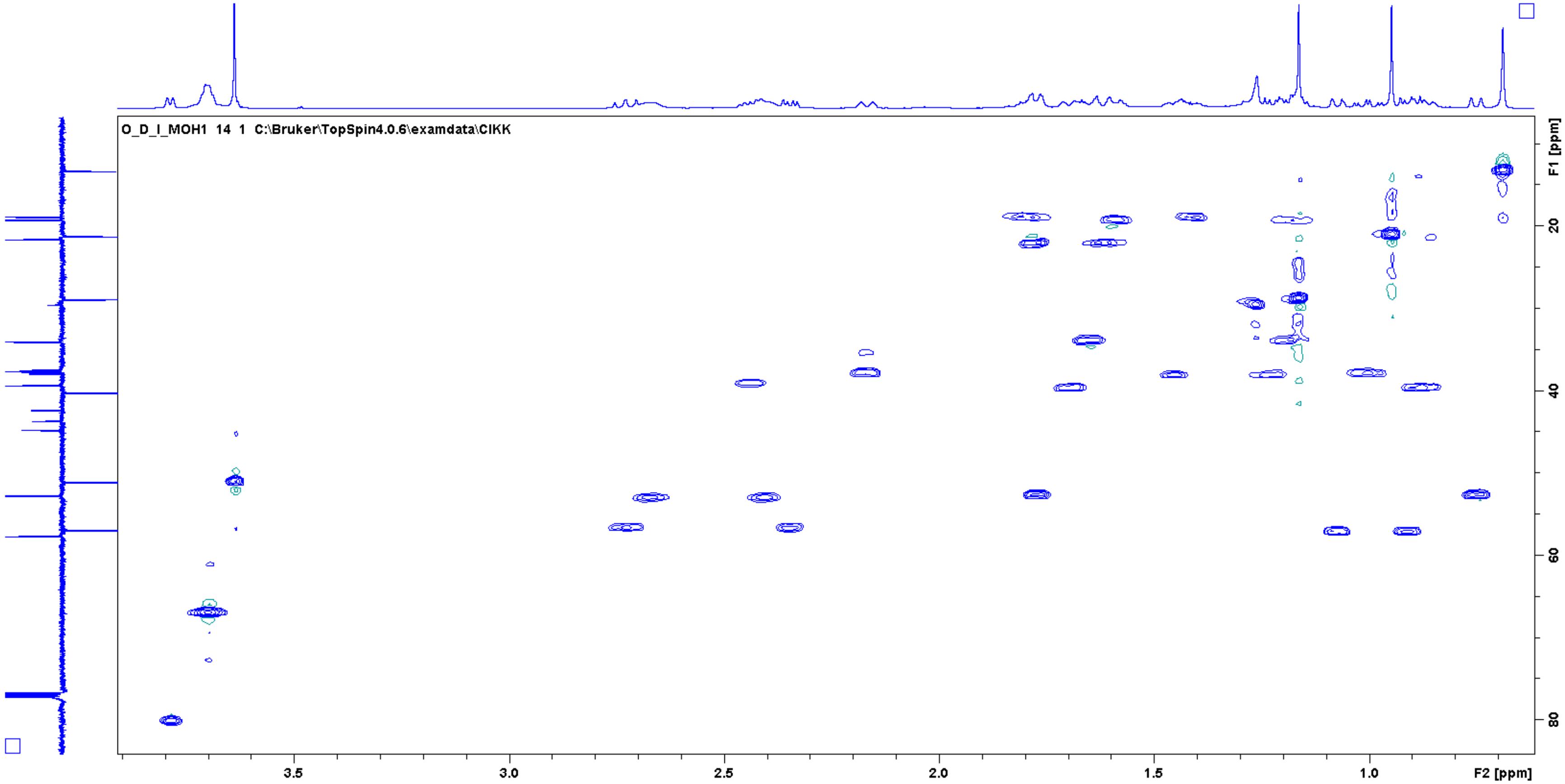

HMBC of compound (4*R*,6a*S*,9*S*,11b*S*)-Methyl 8-hydroxy-4,9,11b-trimethyl-7-(morpholinomethyl)tetradecahydro-6a,9 methanocyclohepta[a]naphthalene-4-carboxylate (**19a**)

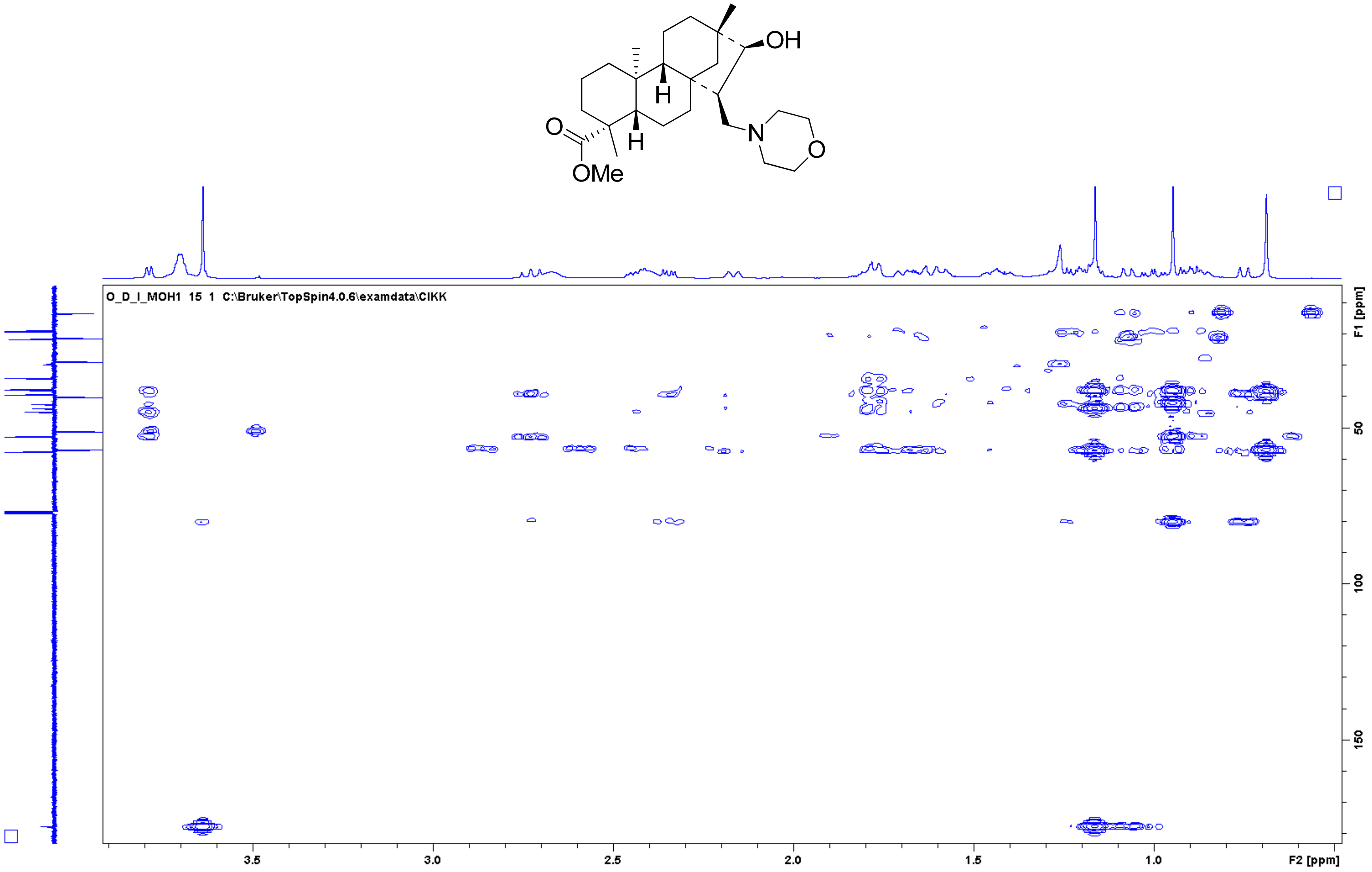

^{1}H-NMR of compound (4*R*,6a*S*,8*R*,9*S*,11b*S*)-Methyl 8-hydroxy-4,9,11b-trimethyl-7-(morpholinomethyl)tetradecahydro-6a,9-methanocyclohepta[a]naphthalene-4-carboxylate (**19b**)

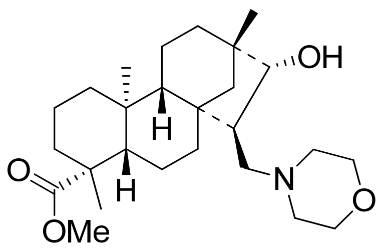

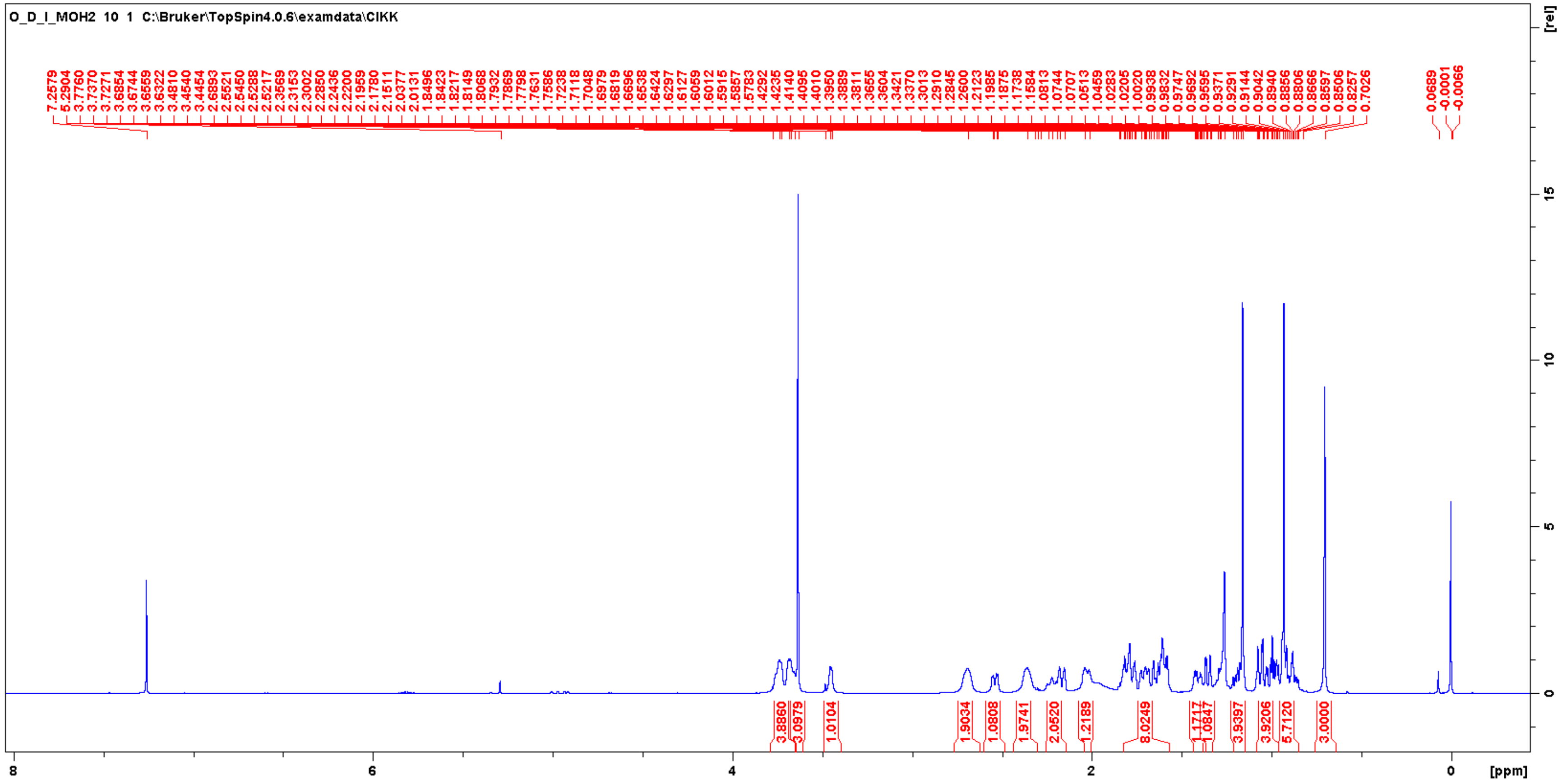

^{13}C-NMR of compound (4*R*,6a*S*,8*R*,9*S*,11b*S*)-Methyl 8-hydroxy-4,9,11b-trimethyl-7-(morpholinomethyl)tetradecahydro-6a,9-methanocyclohepta[a]naphthalene-4-carboxylate (**19b**)

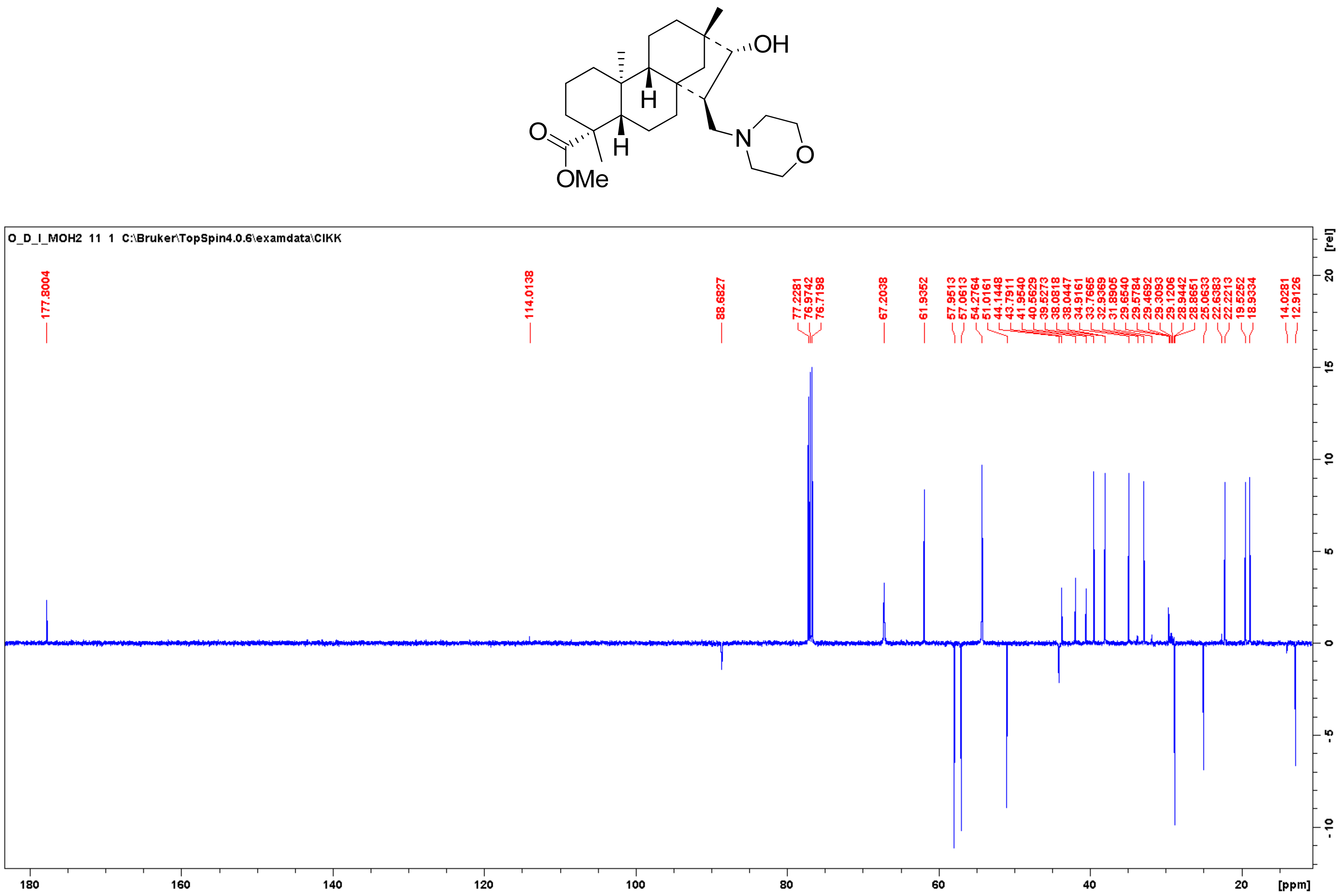

COSY of compound (4*R*,6a*S*,8*R*,9*S*,11b*S*)-Methyl 8-hydroxy-4,9,11b-trimethyl-7-(morpholinomethyl)tetradecahydro-6a,9-methanocyclohepta[a]naphthalene-4-carboxylate (**19b**)

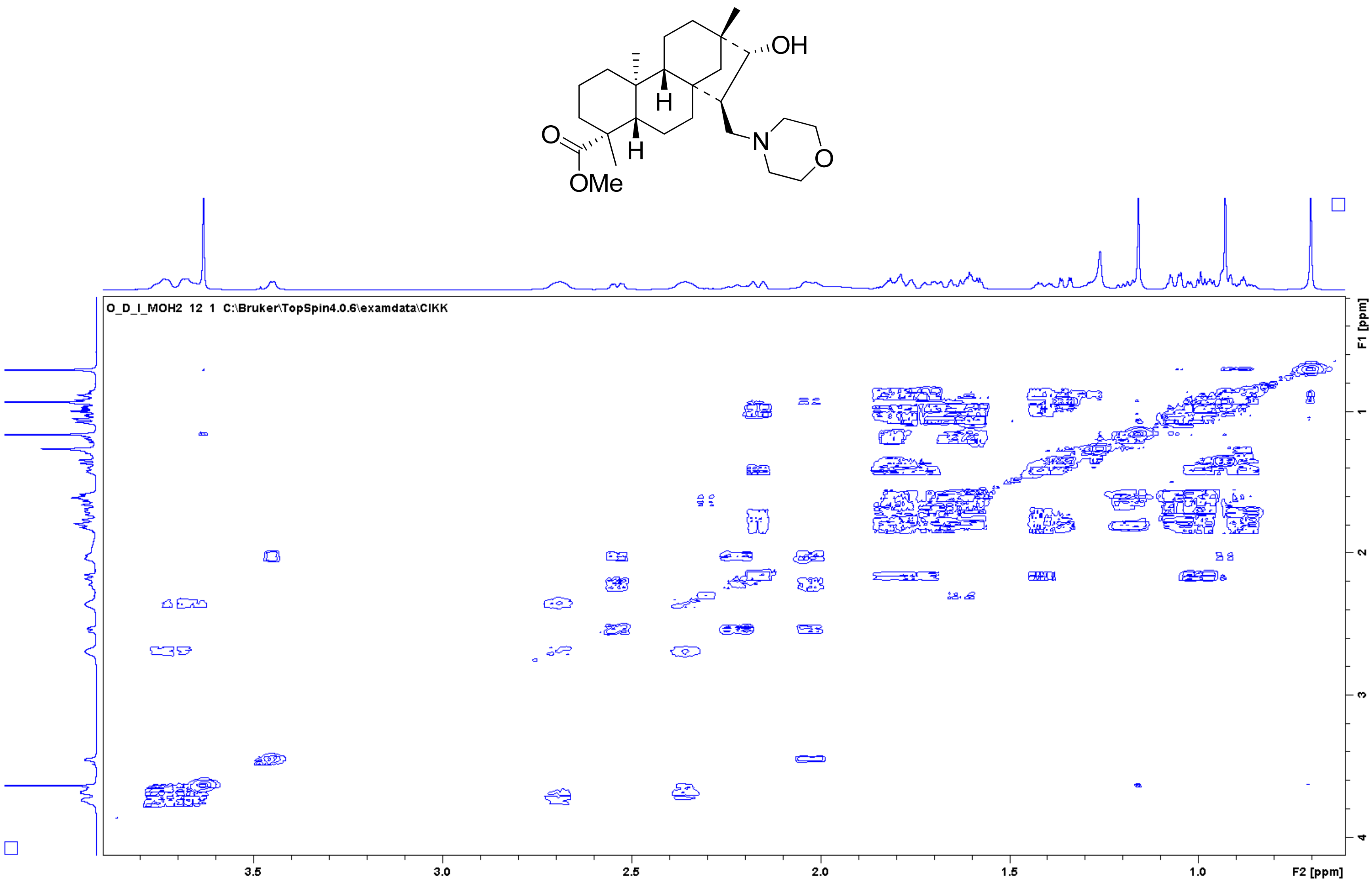

HSQC of compound (4*R*,6a*S*,8*R*,9*S*,11b*S*)-Methyl 8-hydroxy-4,9,11b-trimethyl-7-(morpholinomethyl)tetradecahydro-6a,9-methanocyclohepta[a]naphthalene-4-carboxylate (**19b**)

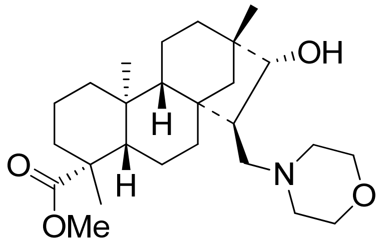

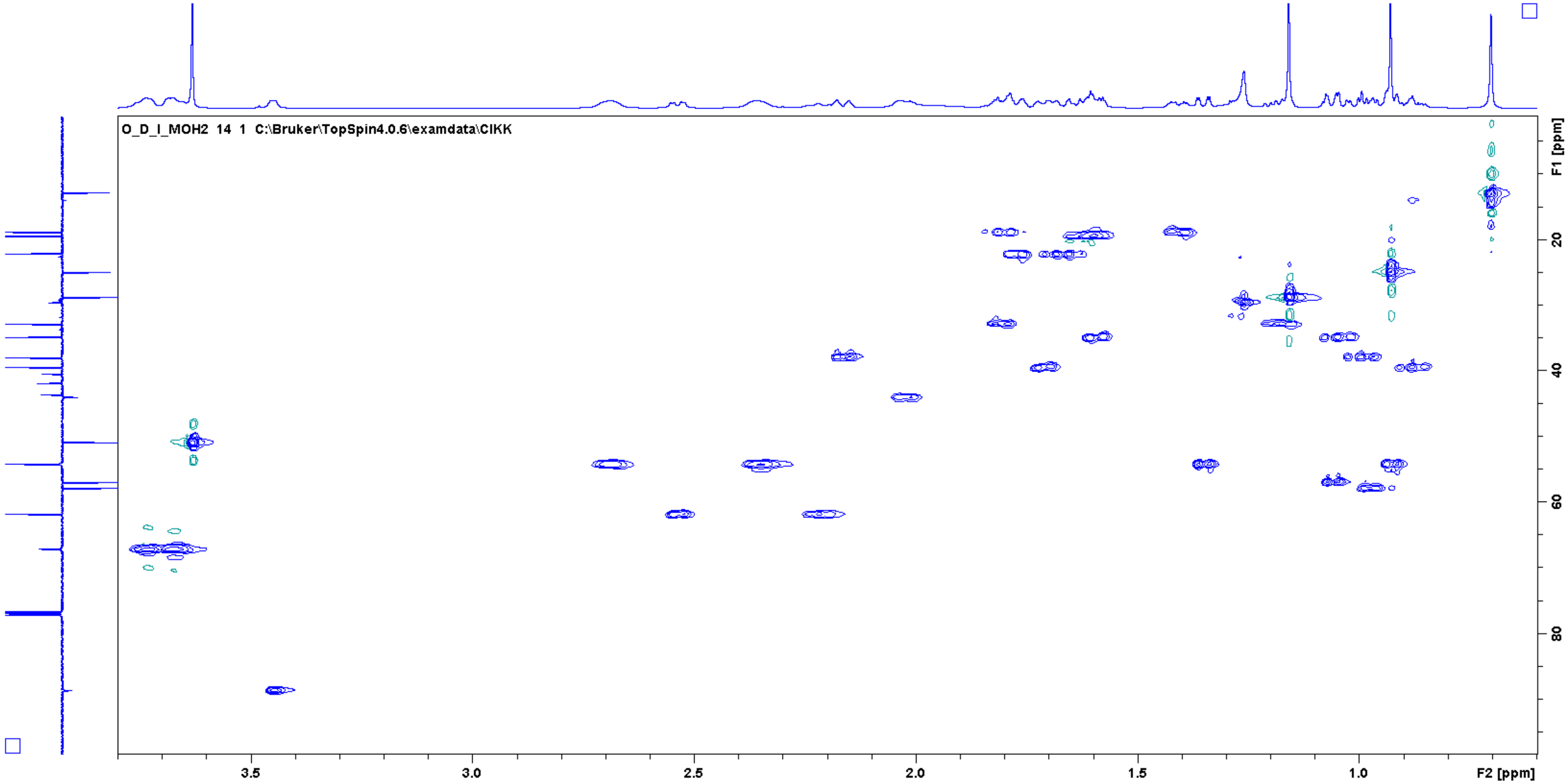

HMBC of compound (4*R*,6a*S*,8*R*,9*S*,11b*S*)-Methyl 8-hydroxy-4,9,11b-trimethyl-7-(morpholinomethyl)tetradecahydro-6a,9-methanocyclohepta[a]naphthalene-4-carboxylate (**19b**)

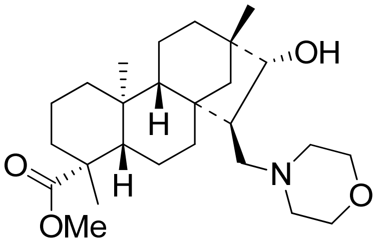

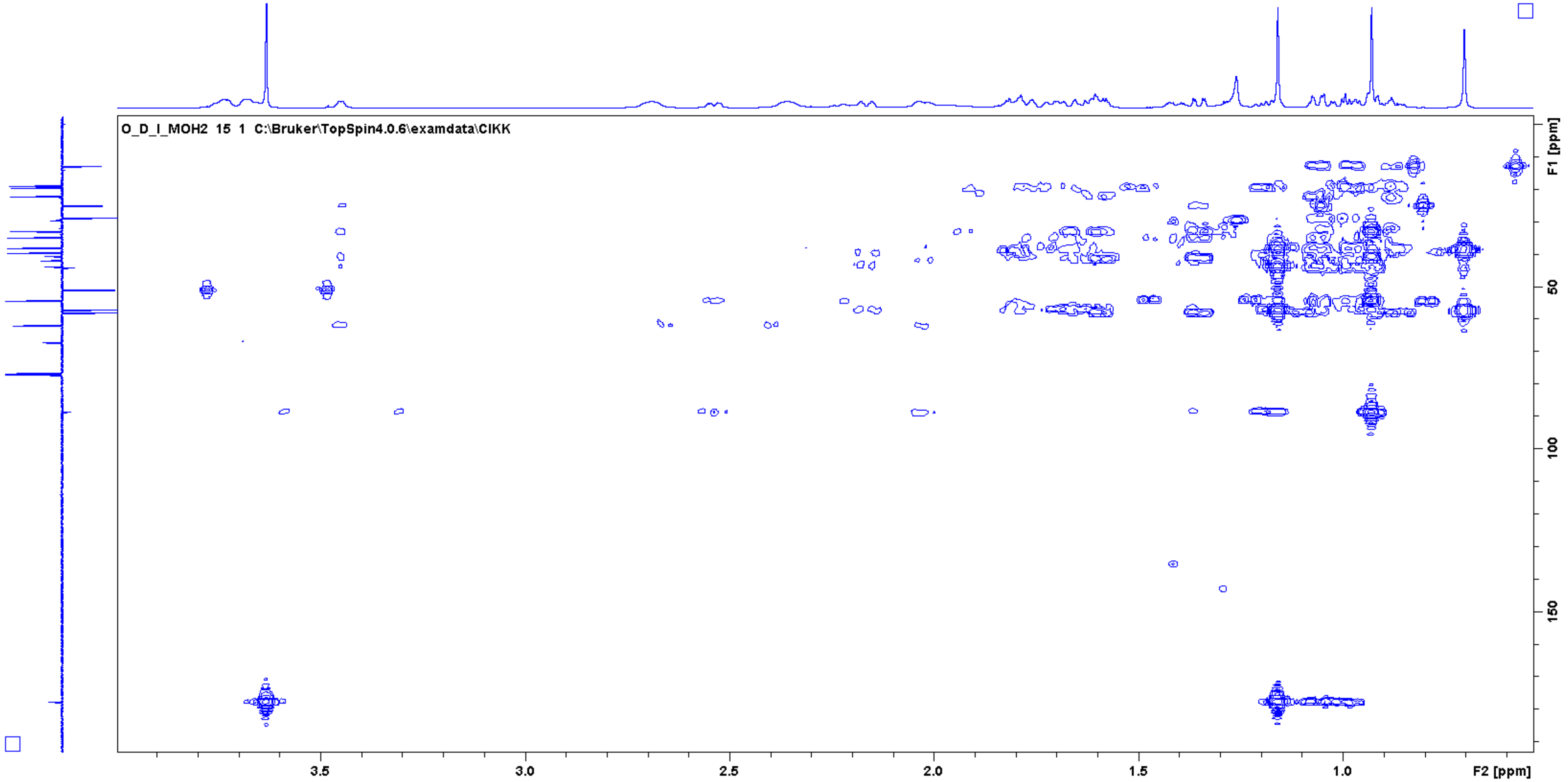

^{1}H-NMR of compound (4*R*,6a*S*,9*S*,11b*S*)-Methyl 7-((benzyl(methyl)amino)methyl)-8-hydroxy-4,9,11b-trimethyltetradecahydro-6a,9-methanocyclohepta[a]naphthalene-4-carboxylate (**20a**)

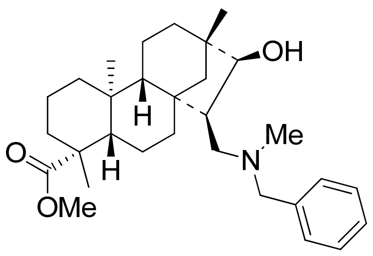

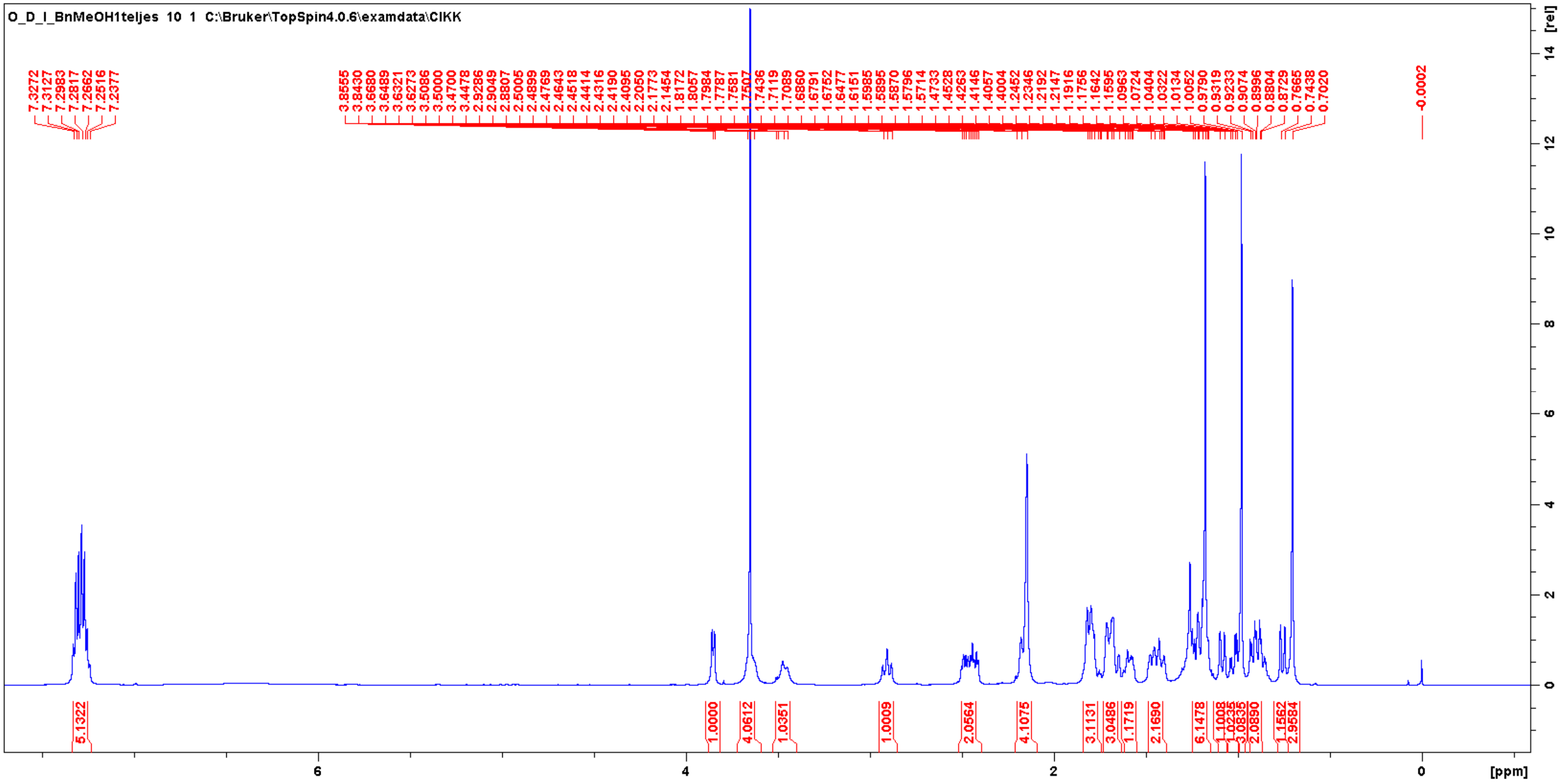

^{13}C-NMR of compound (4*R*,6a*S*,9*S*,11b*S*)-Methyl 7-((benzyl(methyl)amino)methyl)-8-hydroxy-4,9,11b-trimethyltetradecahydro-6a,9-methanocyclohepta[a]naphthalene-4-carboxylate (**20a**)

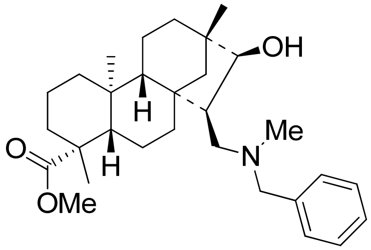

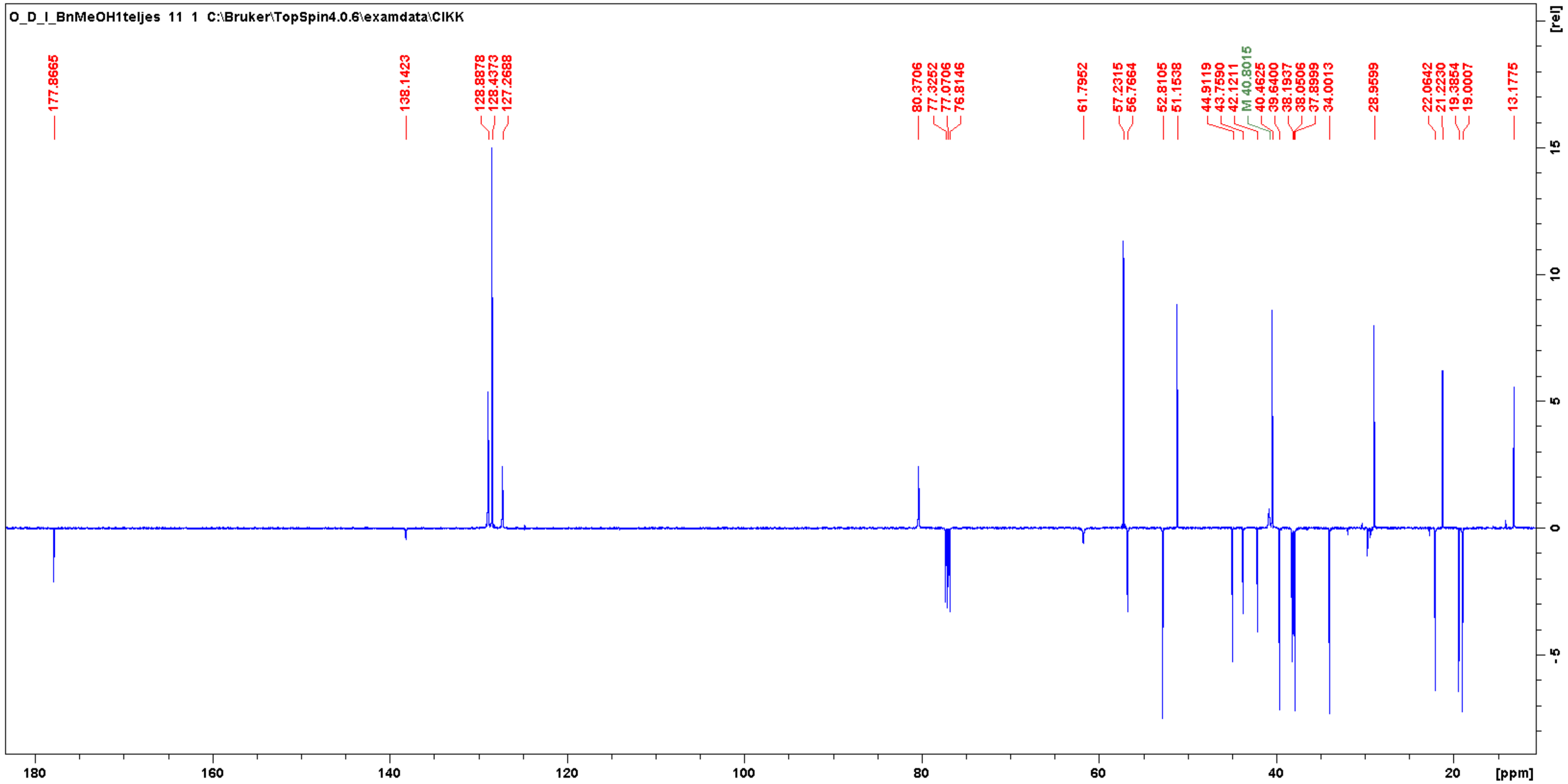

COSY of compound (4*R*,6a*S*,9*S*,11b*S*)-Methyl 7-((benzyl(methyl)amino)methyl)-8-hydroxy-4,9,11b-trimethyltetradecahydro-6a,9-methanocyclohepta[a]naphthalene-4-carboxylate (**20a**)

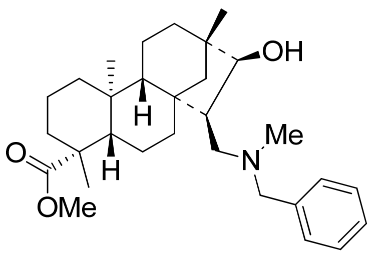

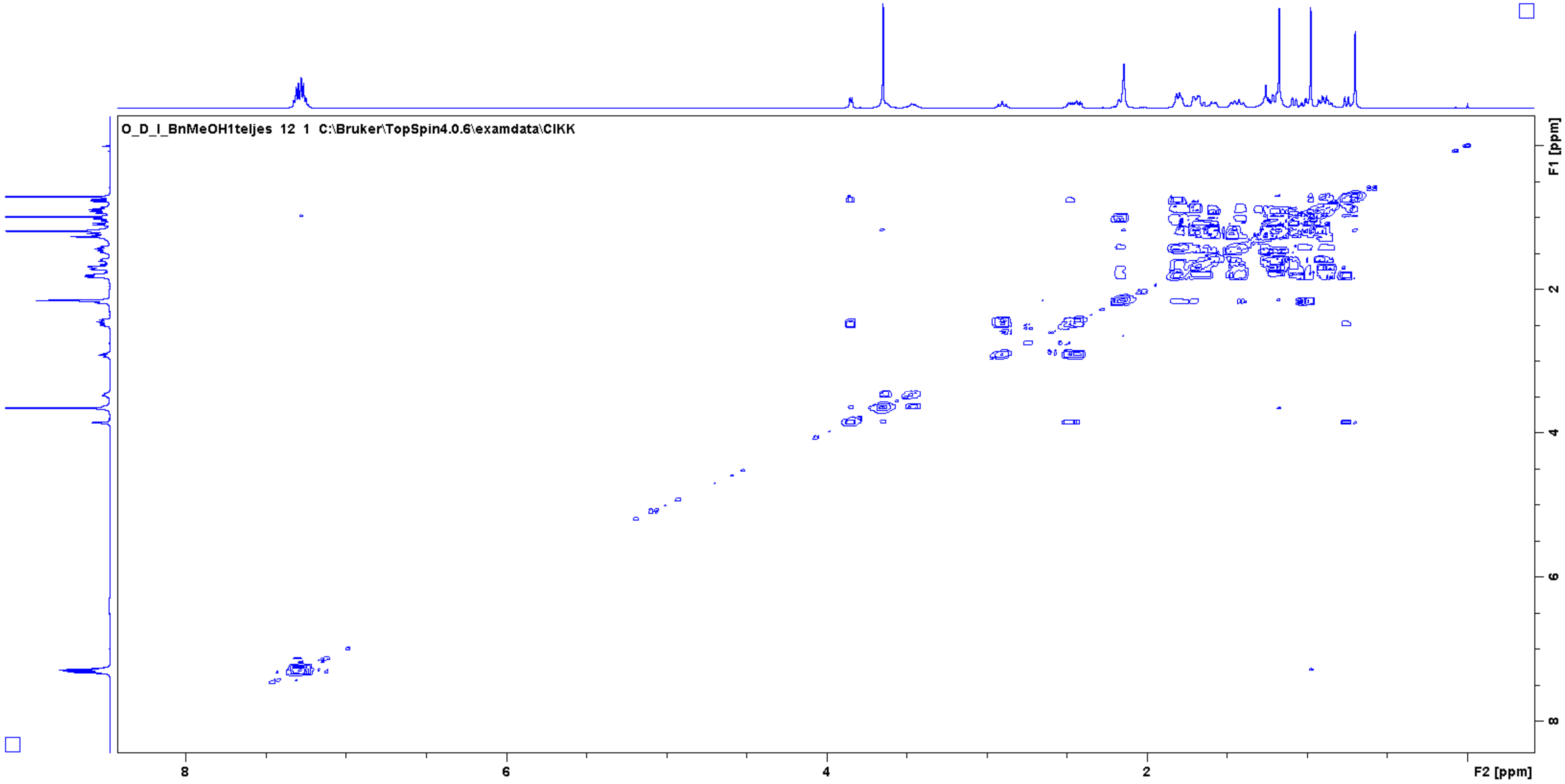

NOESY of compound (4*R*,6a*S*,9*S*,11b*S*)-Methyl 7-((benzyl(methyl)amino)methyl)-8-hydroxy-4,9,11b-trimethyltetradecahydro-6a,9-methanocyclohepta[a]naphthalene-4-carboxylate (**20a**)

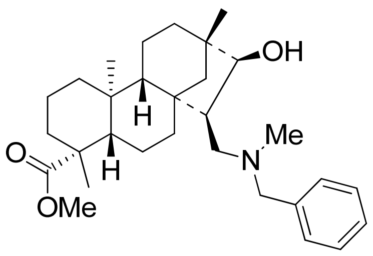

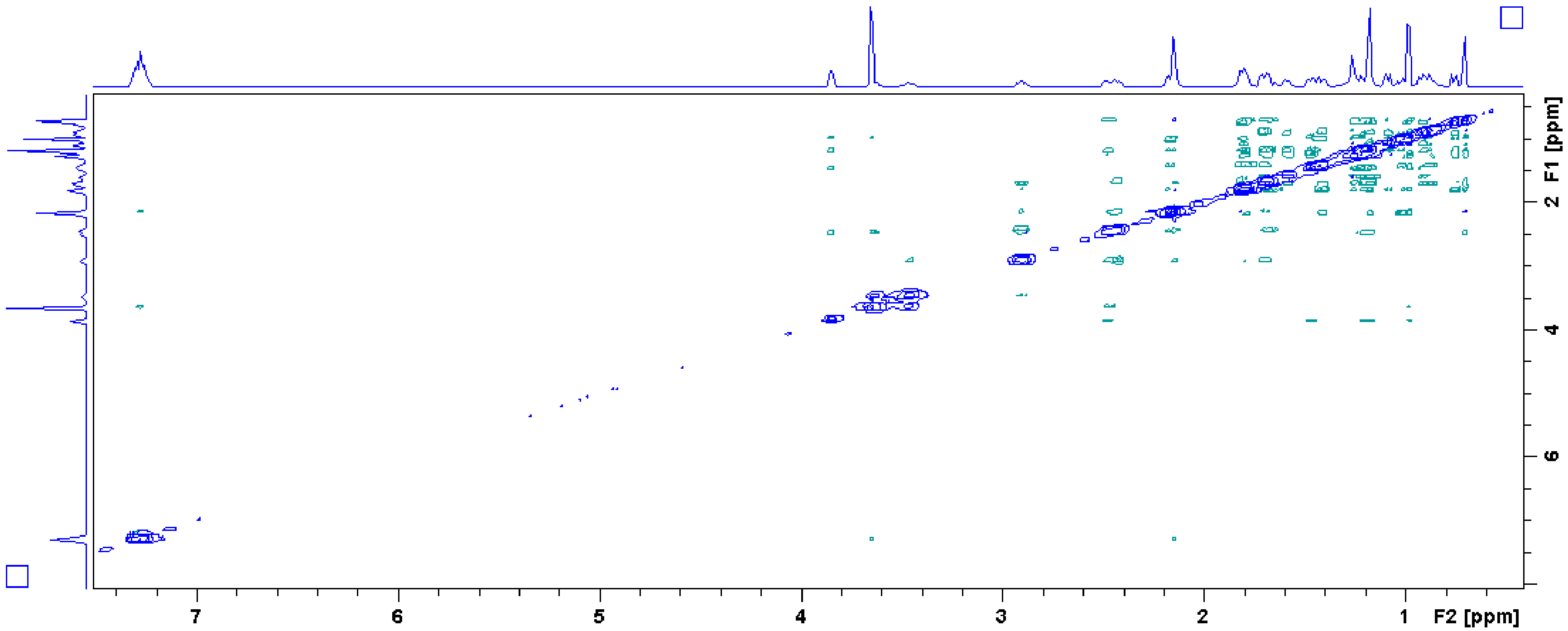

HSQC of compound (4*R*,6a*S*,9*S*,11b*S*)-Methyl 7-((benzyl(methyl)amino)methyl)-8-hydroxy-4,9,11b-trimethyltetradecahydro-6a,9-methanocyclohepta[a]naphthalene-4-carboxylate (**20a**)

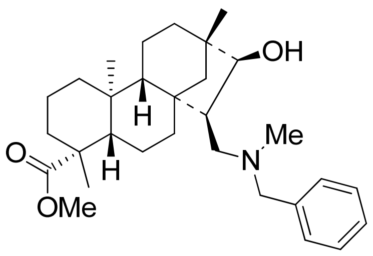

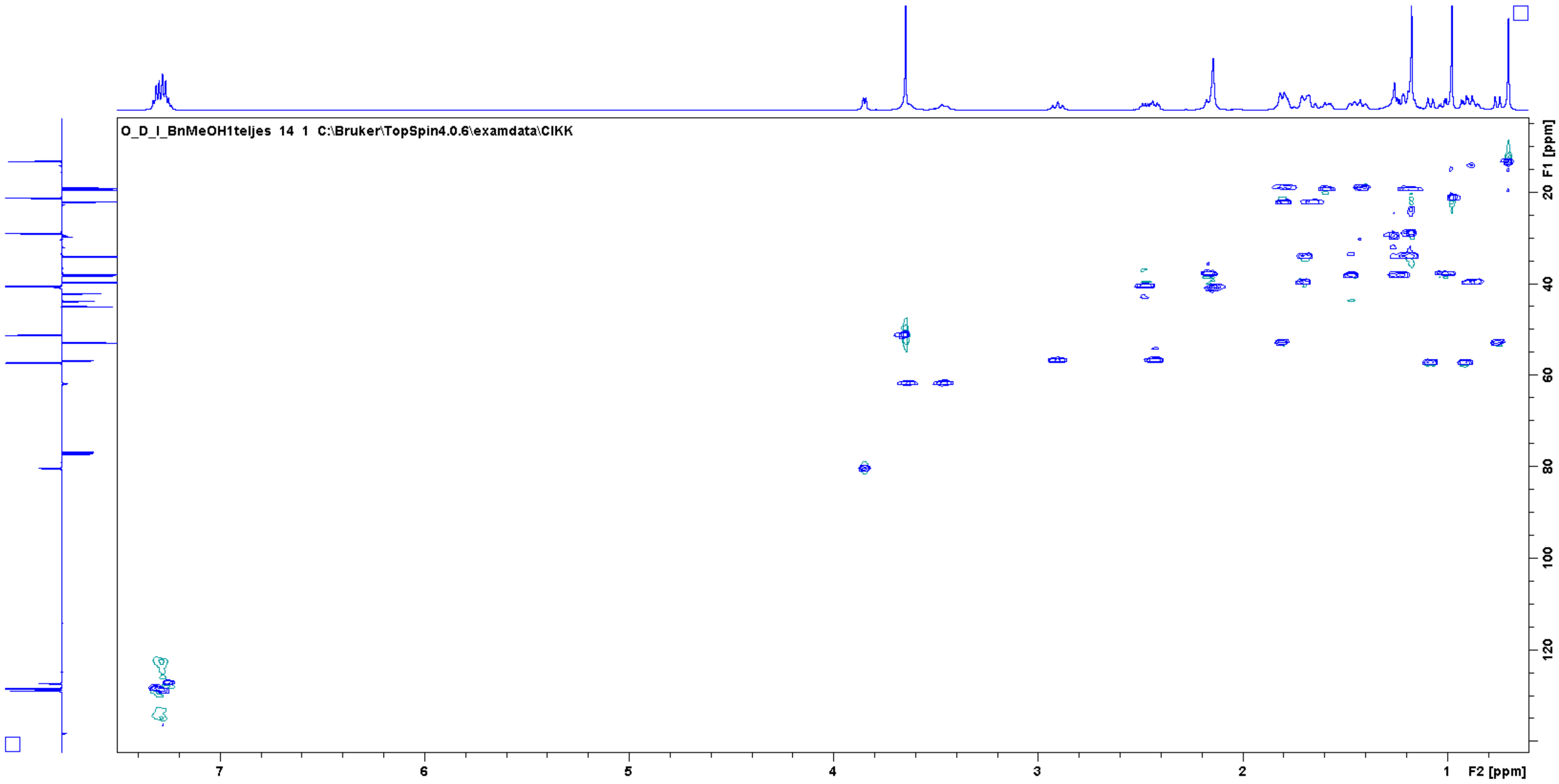

HMBC of compound (4*R*,6a*S*,9*S*,11b*S*)-Methyl 7-((benzyl(methyl)amino)methyl)-8-hydroxy-4,9,11b-trimethyltetradecahydro-6a,9-methanocyclohepta[a]naphthalene-4-carboxylate (**20a**)

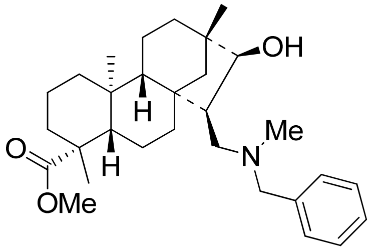

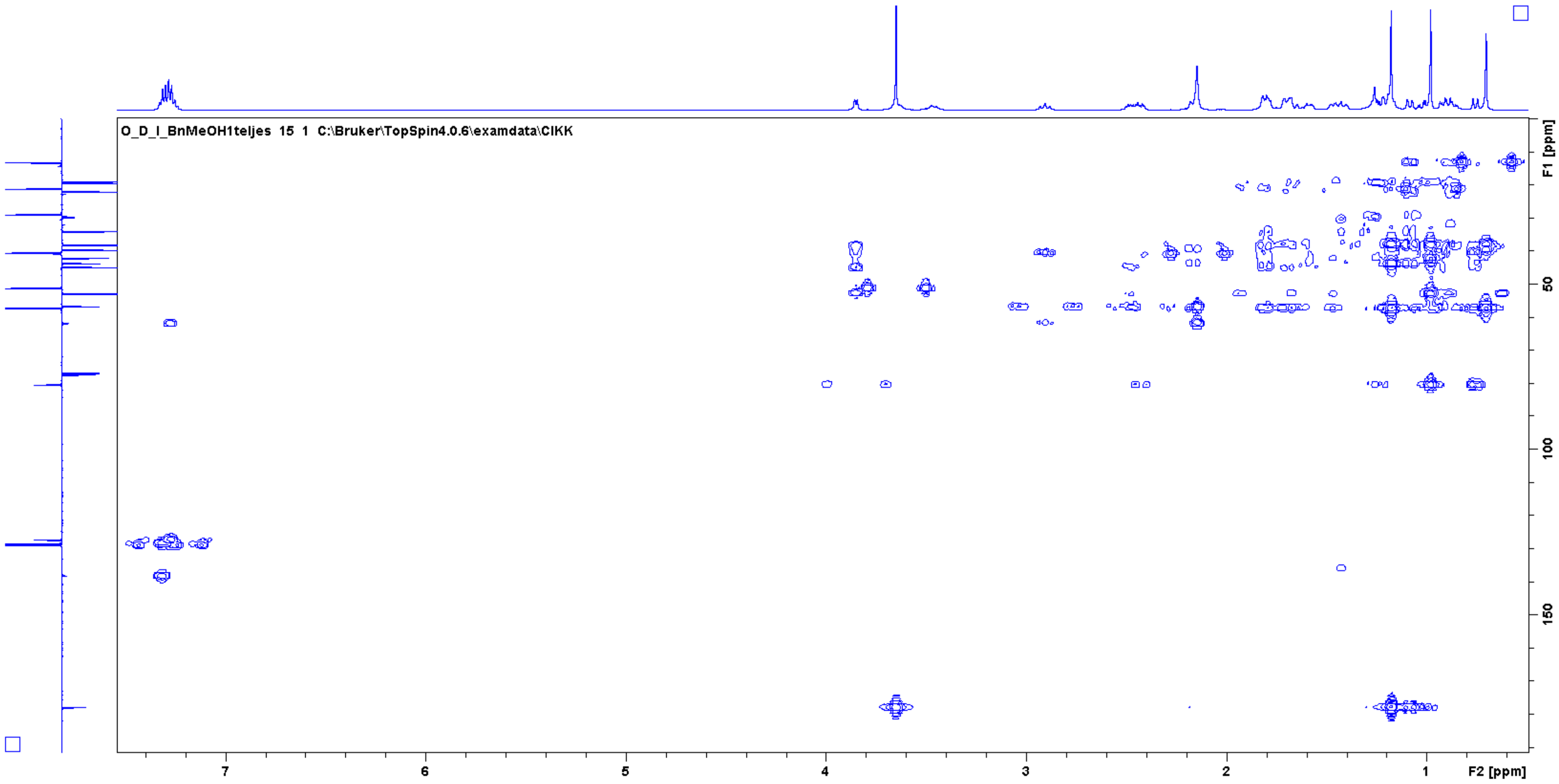

^{1}H-NMR of compound (4*R*,6a*S*,8*R*,9*S*,11b*S*)-Methyl 7-((benzyl(methyl)amino)methyl)-8-hydroxy-4,9,11b-trimethyltetradecahydro-6a,9-methanocyclohepta[a]naphthalene-4-carboxylate (**20b**)

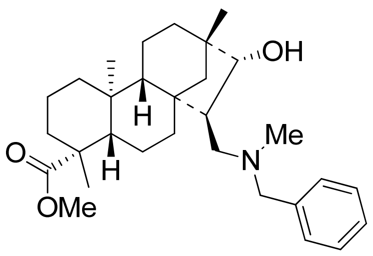

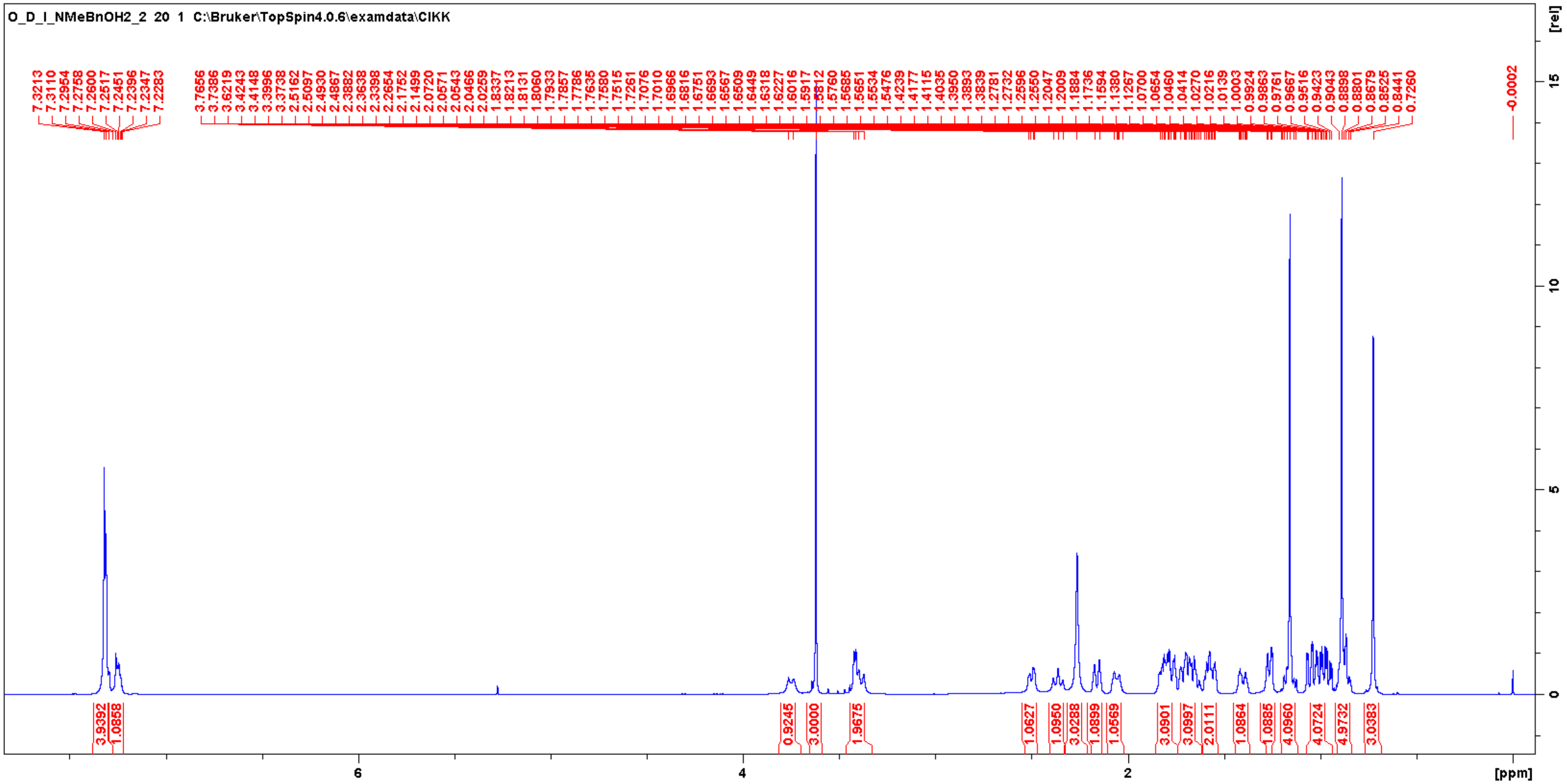

^{13}C-NMR of compound (4*R*,6a*S*,8*R*,9*S*,11b*S*)-Methyl 7-((benzyl(methyl)amino)methyl)-8-hydroxy-4,9,11b-trimethyltetradecahydro-6a,9-methanocyclohepta[a]naphthalene-4-carboxylate (**20b**)

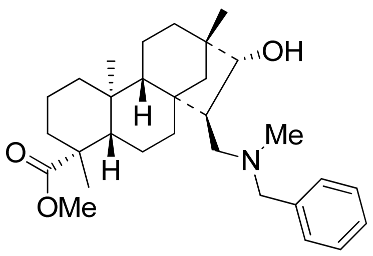

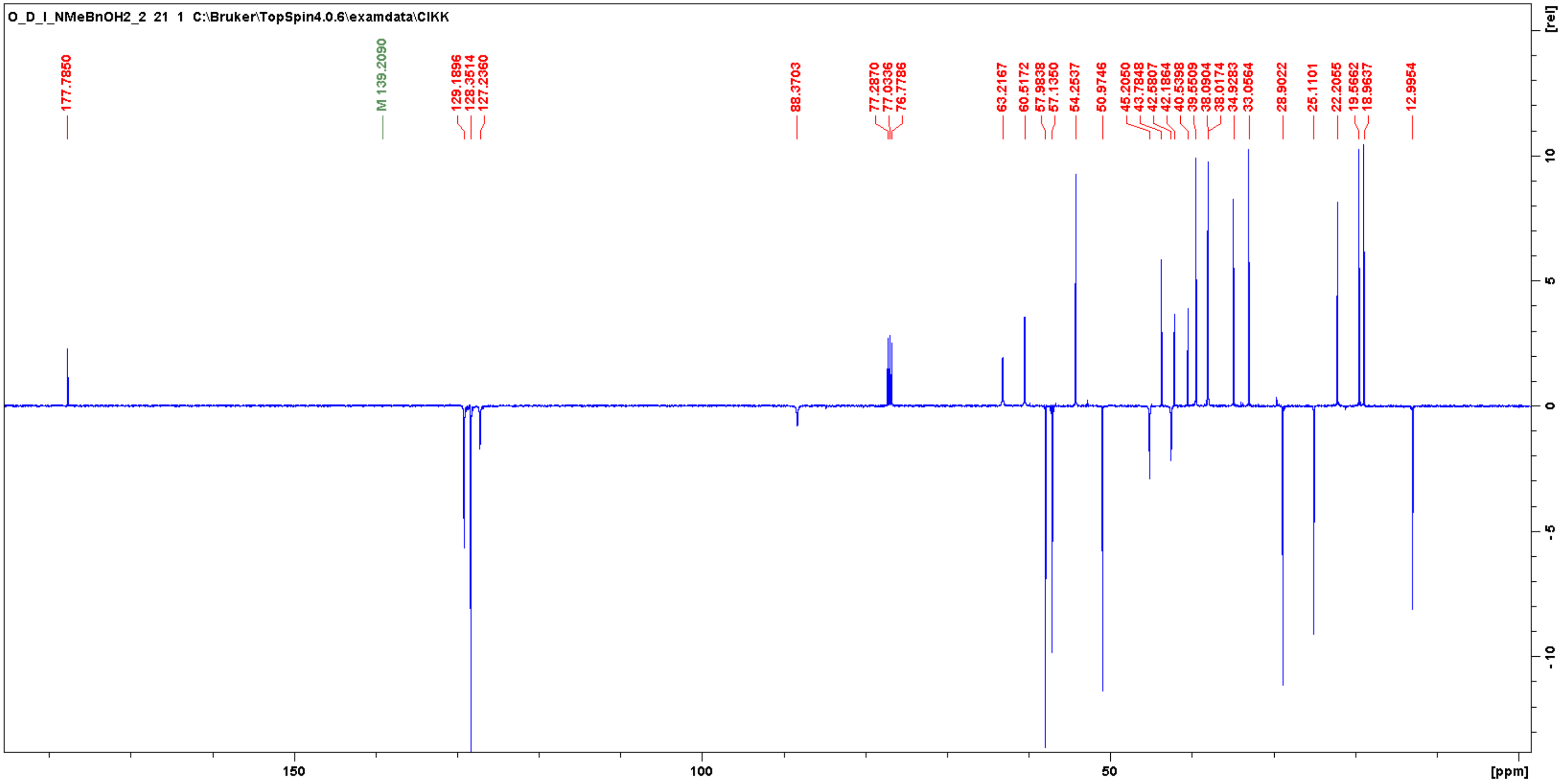

COSY of compound (4*R*,6a*S*,8*R*,9*S*,11b*S*)-Methyl 7-((benzyl(methyl)amino)methyl)-8-hydroxy-4,9,11b-trimethyltetradecahydro-6a,9-methanocyclohepta[a]naphthalene-4-carboxylate (**20b**)

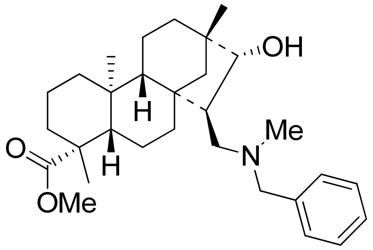

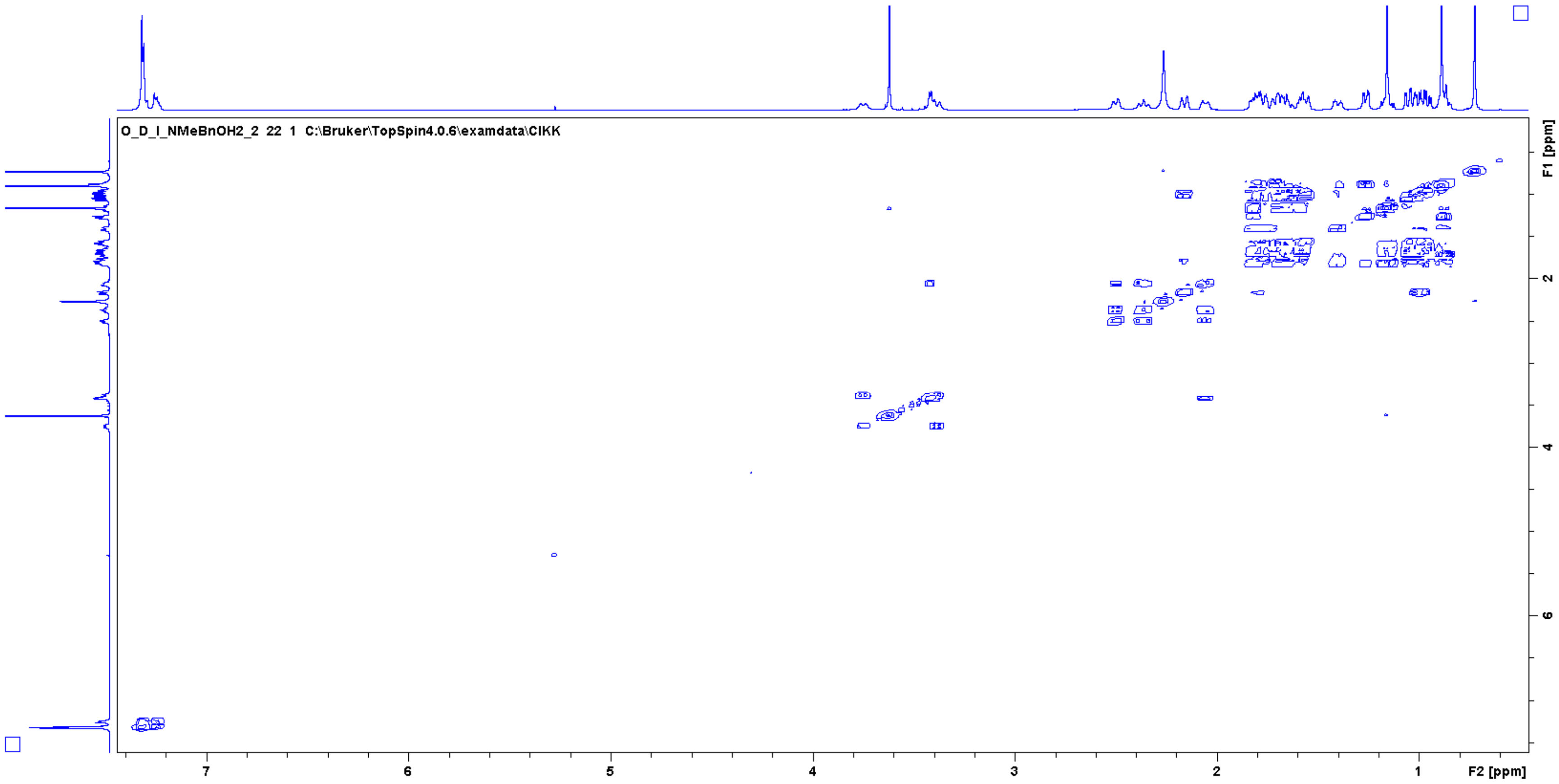

NOESY of compound (4*R*,6a*S*,8*R*,9*S*,11b*S*)-Methyl 7-((benzyl(methyl)amino)methyl)-8-hydroxy-4,9,11b-trimethyltetradecahydro-6a,9-methanocyclohepta[a]naphthalene-4-carboxylate (**20b**)

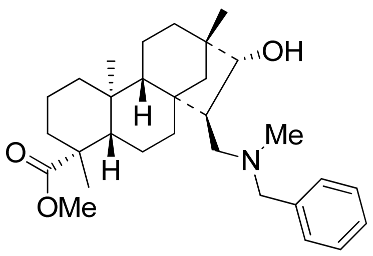

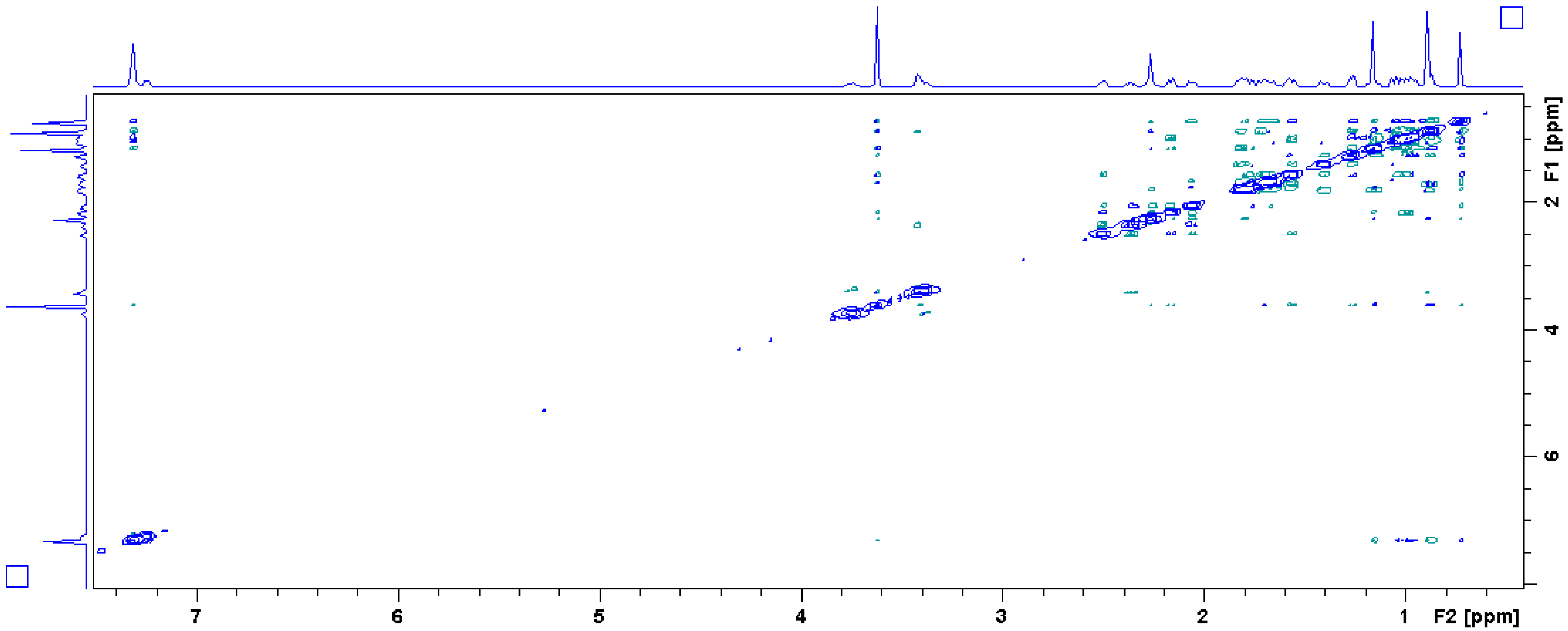

HSQC of compound (4*R*,6a*S*,8*R*,9*S*,11b*S*)-Methyl 7-((benzyl(methyl)amino)methyl)-8-hydroxy-4,9,11b-trimethyltetradecahydro-6a,9-methanocyclohepta[a]naphthalene-4-carboxylate (**20b**)

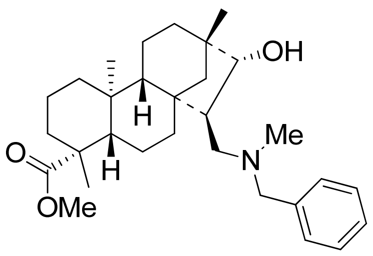

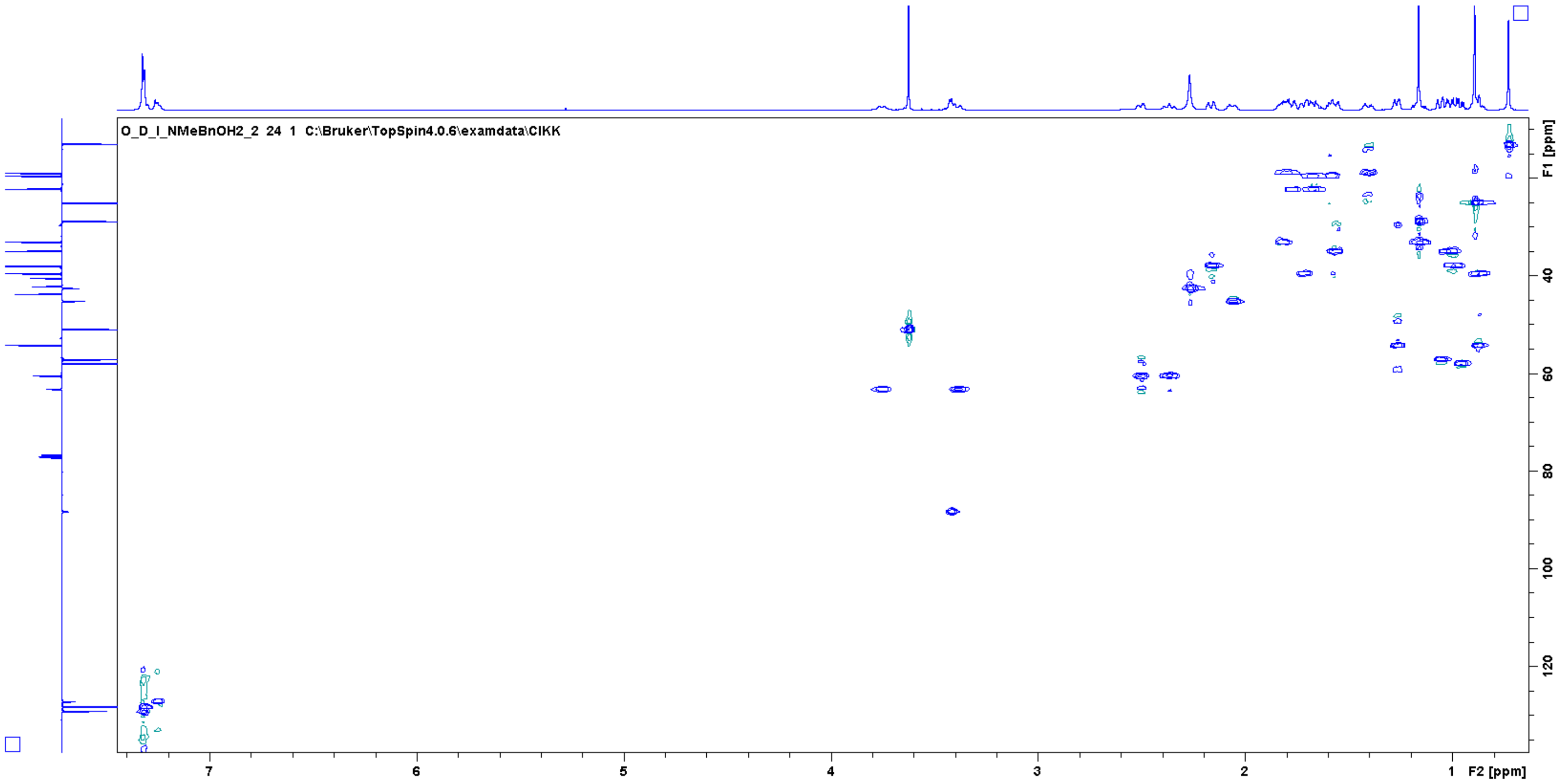

HMBC of compound (4*R*,6a*S*,8*R*,9*S*,11b*S*)-Methyl 7-((benzyl(methyl)amino)methyl)-8-hydroxy-4,9,11b-trimethyltetradecahydro-6a,9-methanocyclohepta[a]naphthalene-4-carboxylate (**20b**)

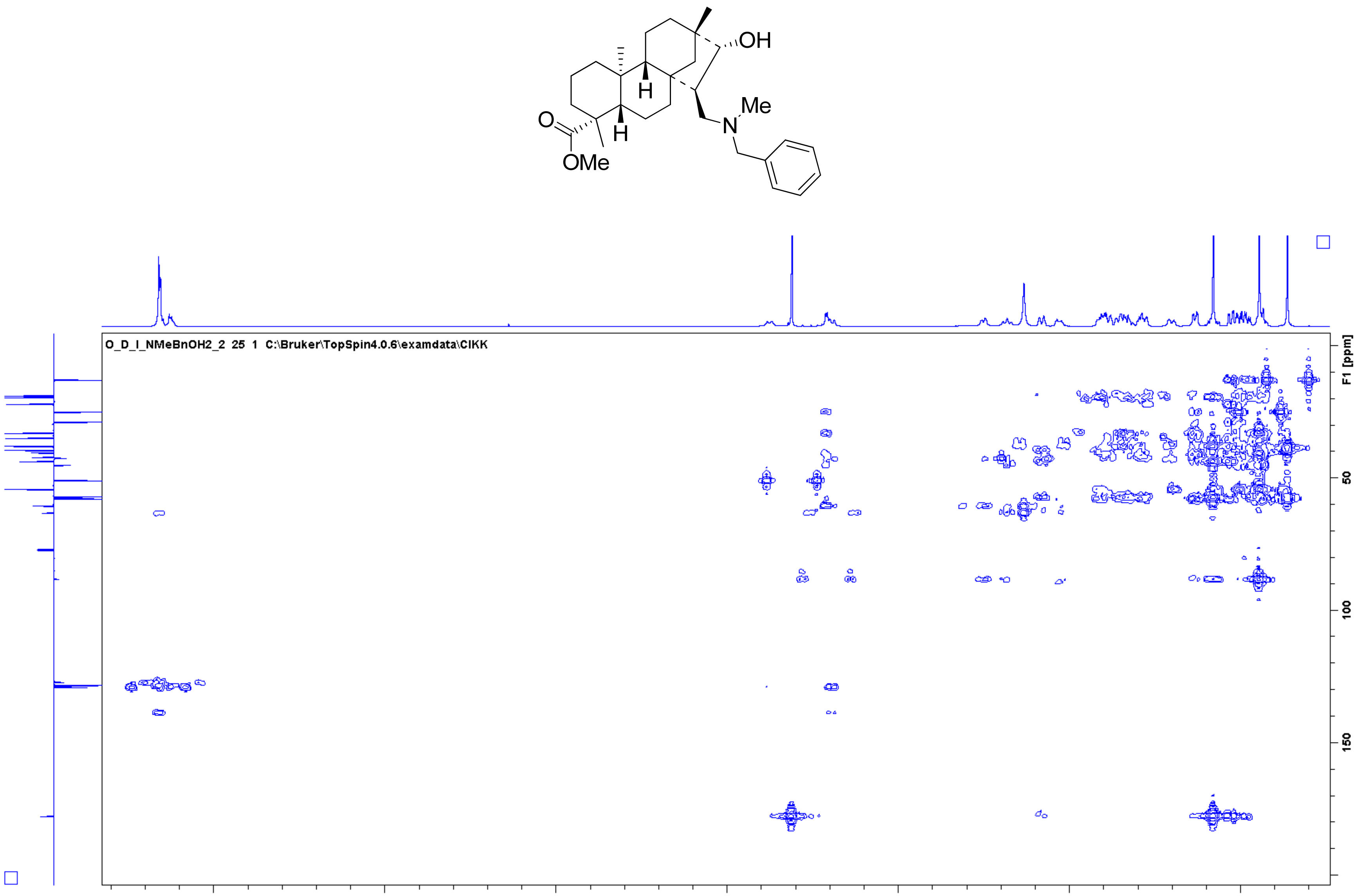

^{1}H-NMR of compound (4*R*,6a*S*,8*R*,9*S*,11b*S*)-Methyl 8-hydroxy-4,9,11b-trimethyl-7-(pyrrolidin-1-ylmethyl)tetradecahydro-6a,9-methanocyclohepta[a]naphthalene-4-carboxylate (**21**)

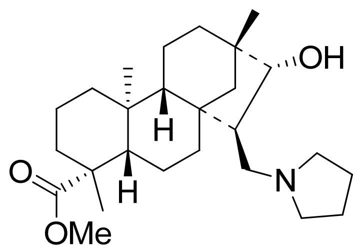

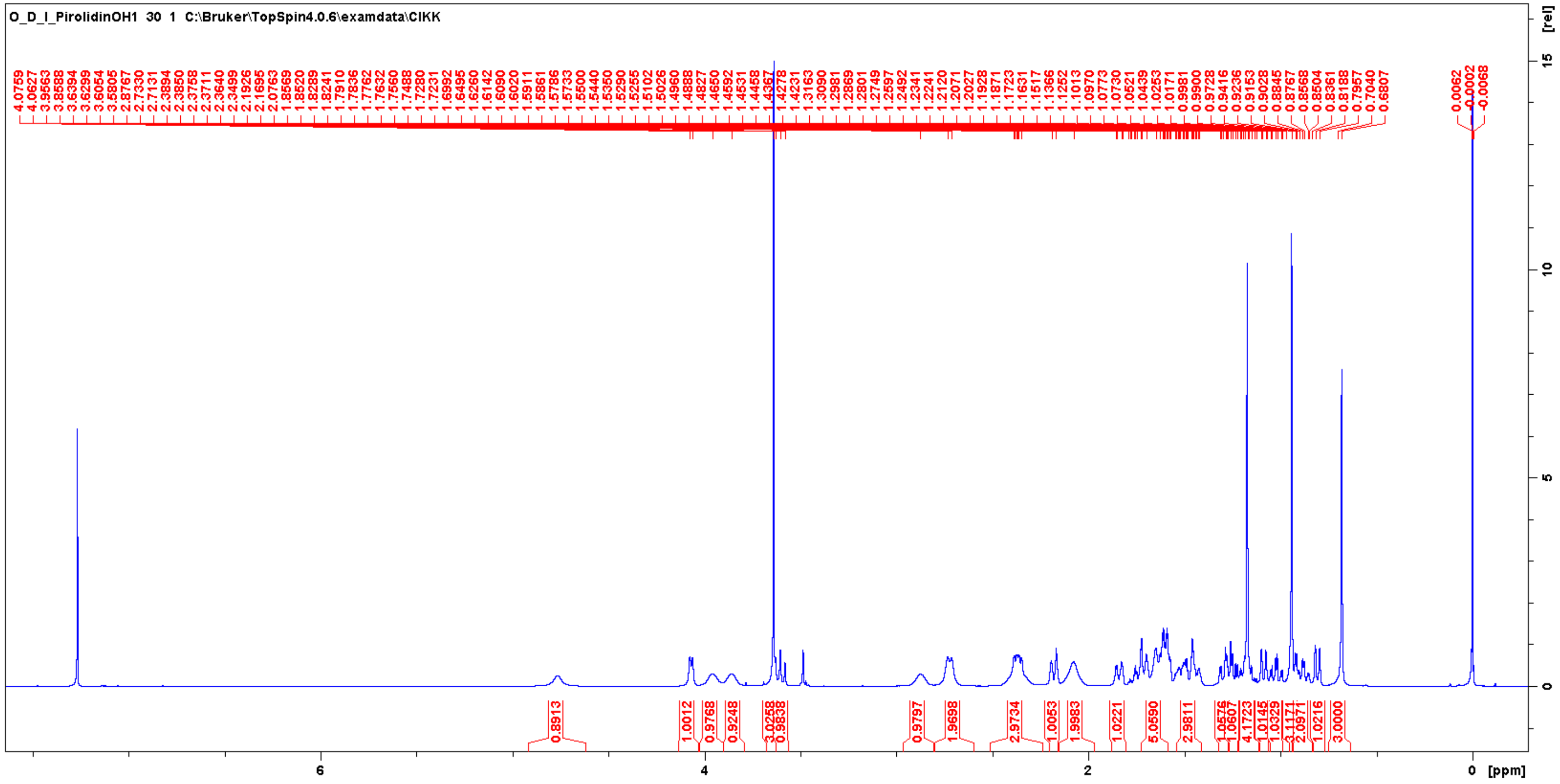

^{13}C-NMR of compound (4*R*,6a*S*,8*R*,9*S*,11b*S*)-Methyl 8-hydroxy-4,9,11b-trimethyl-7-(pyrrolidin-1-ylmethyl)tetradecahydro-6a,9-methanocyclohepta[a]naphthalene-4-carboxylate (**21**)

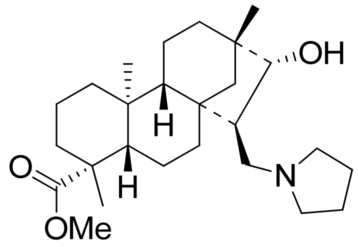

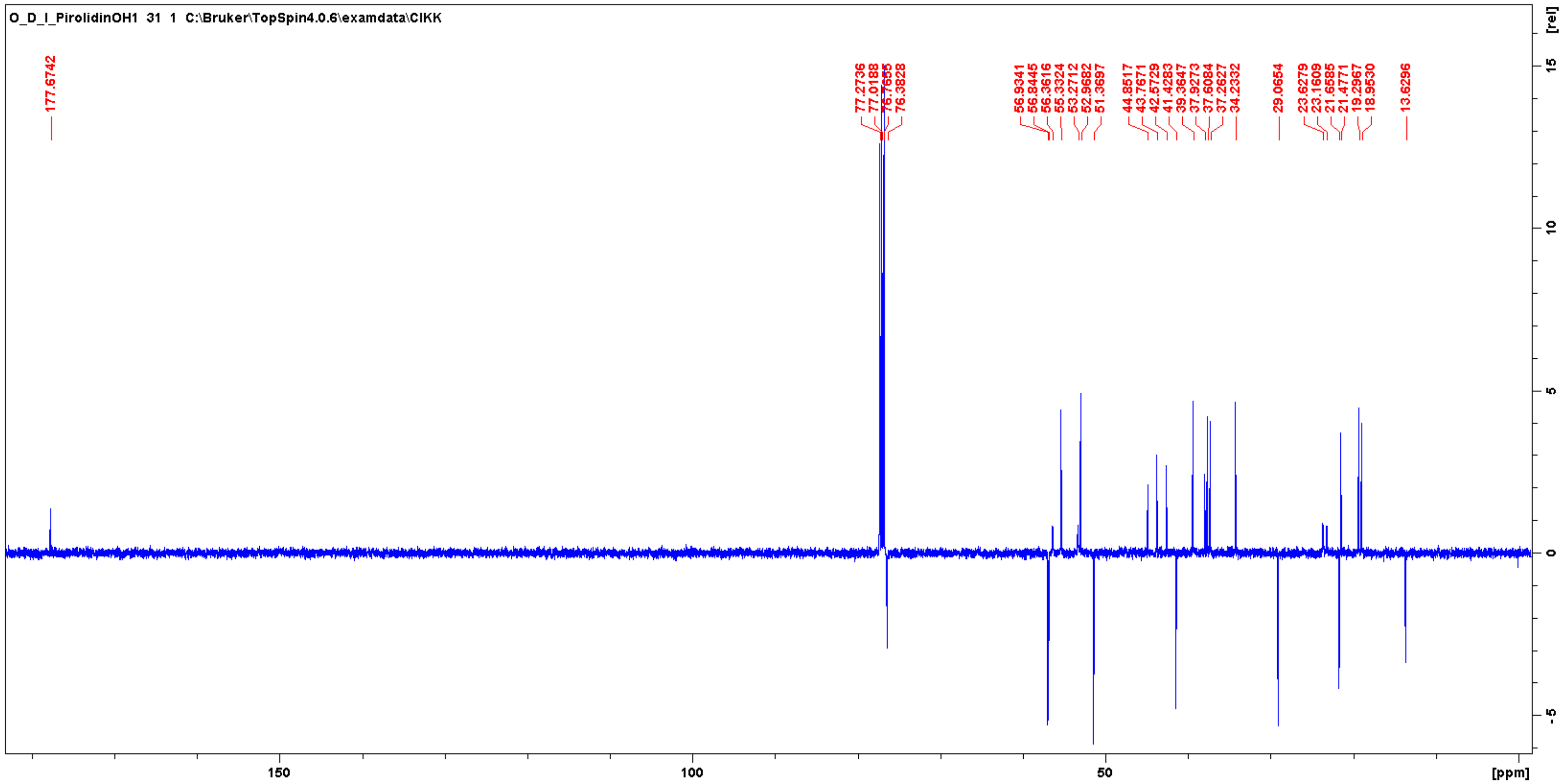

COSY of compound (4*R*,6a*S*,8*R*,9*S*,11b*S*)-Methyl 8-hydroxy-4,9,11b-trimethyl-7-(pyrrolidin-1-ylmethyl)tetradecahydro-6a,9-methanocyclohepta[a]naphthalene-4-carboxylate (**21**)

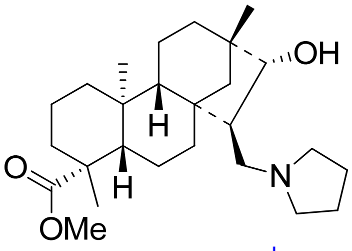

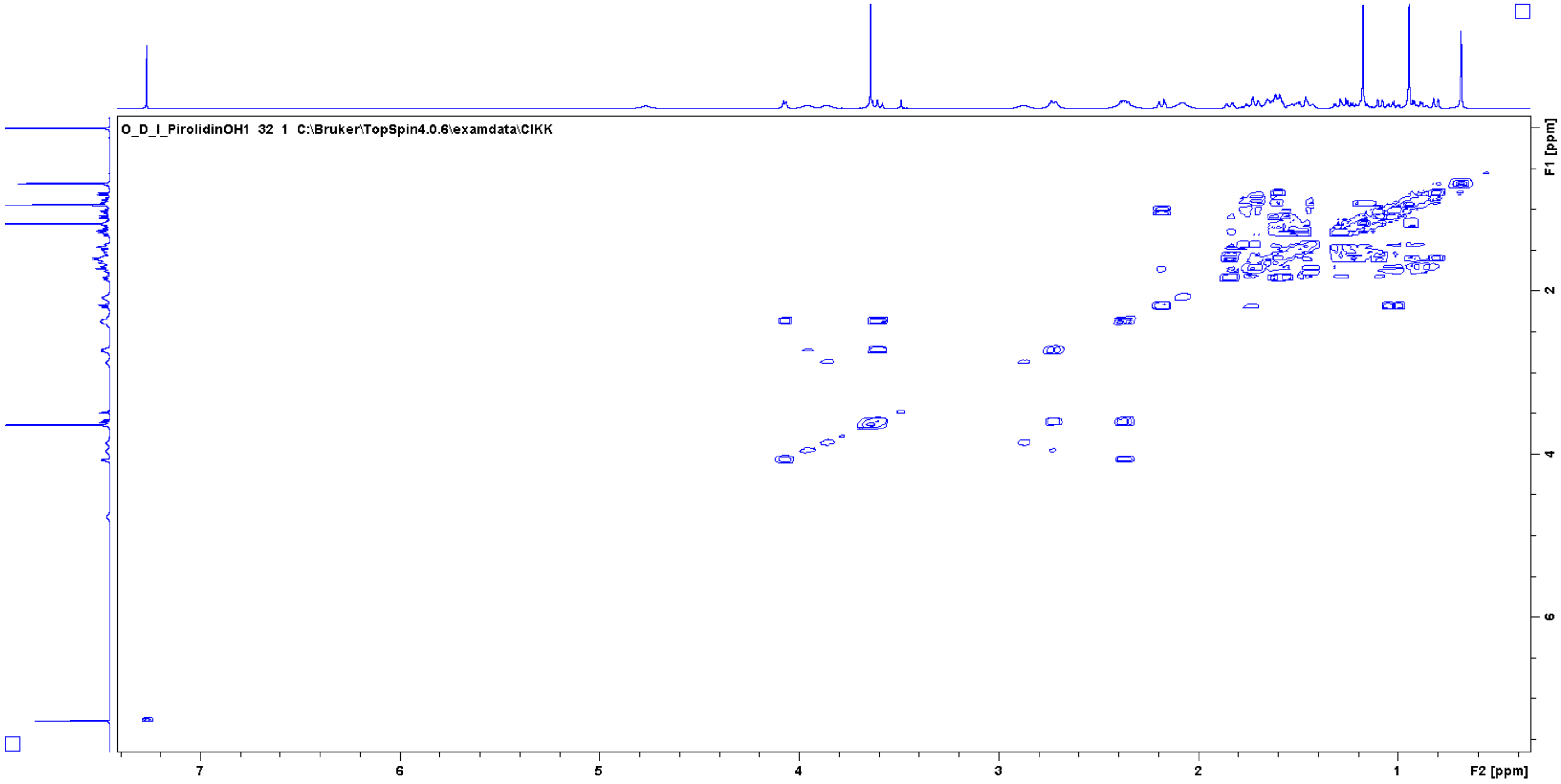

NOESY of compound (4*R*,6a*S*,8*R*,9*S*,11b*S*)-Methyl 8-hydroxy-4,9,11b-trimethyl-7-(pyrrolidin-1-ylmethyl)tetradecahydro-6a,9-methanocyclohepta[a]naphthalene-4-carboxylate (**21**)

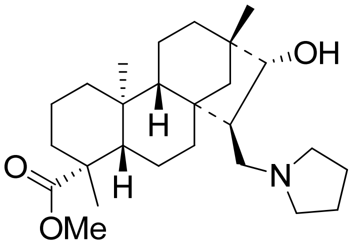

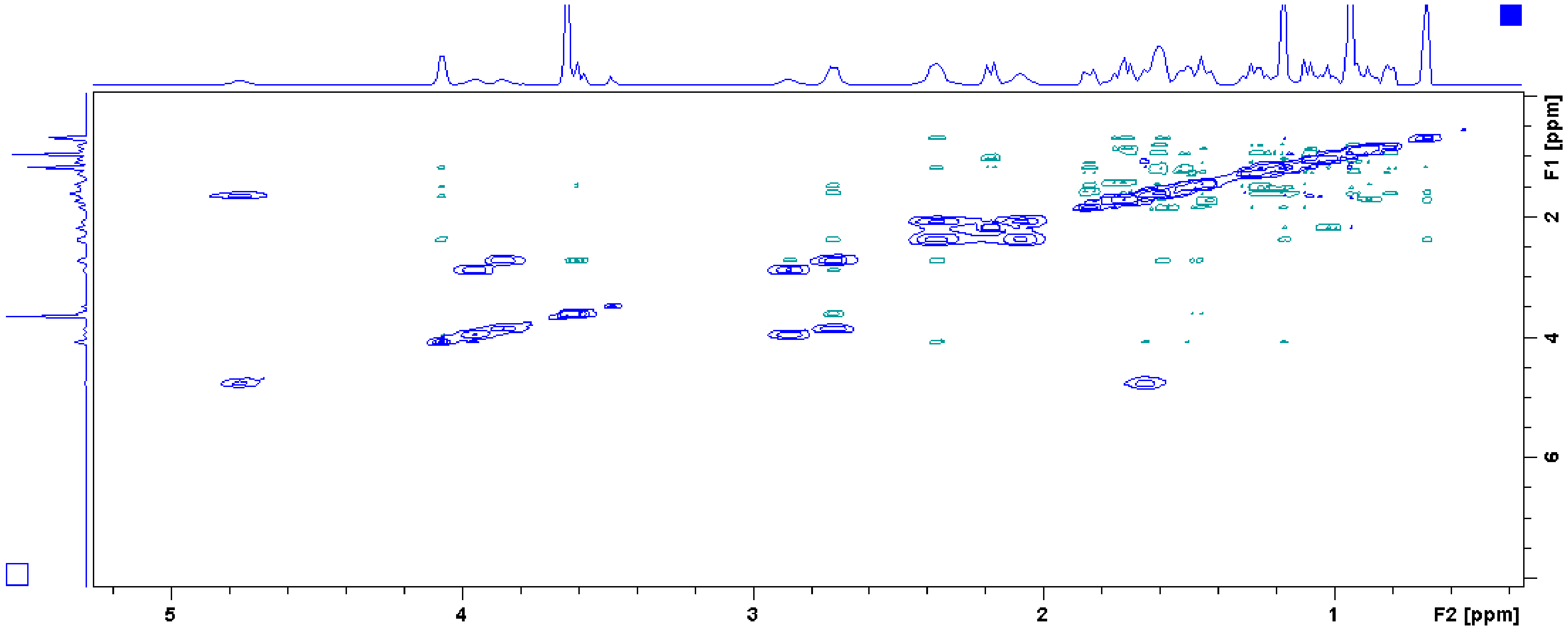

HSQC of compound (4*R*,6a*S*,8*R*,9*S*,11b*S*)-Methyl 8-hydroxy-4,9,11b-trimethyl-7-(pyrrolidin-1-ylmethyl)tetradecahydro-6a,9-methanocyclohepta[a]naphthalene-4-carboxylate (**21**)

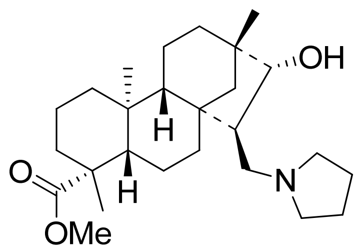

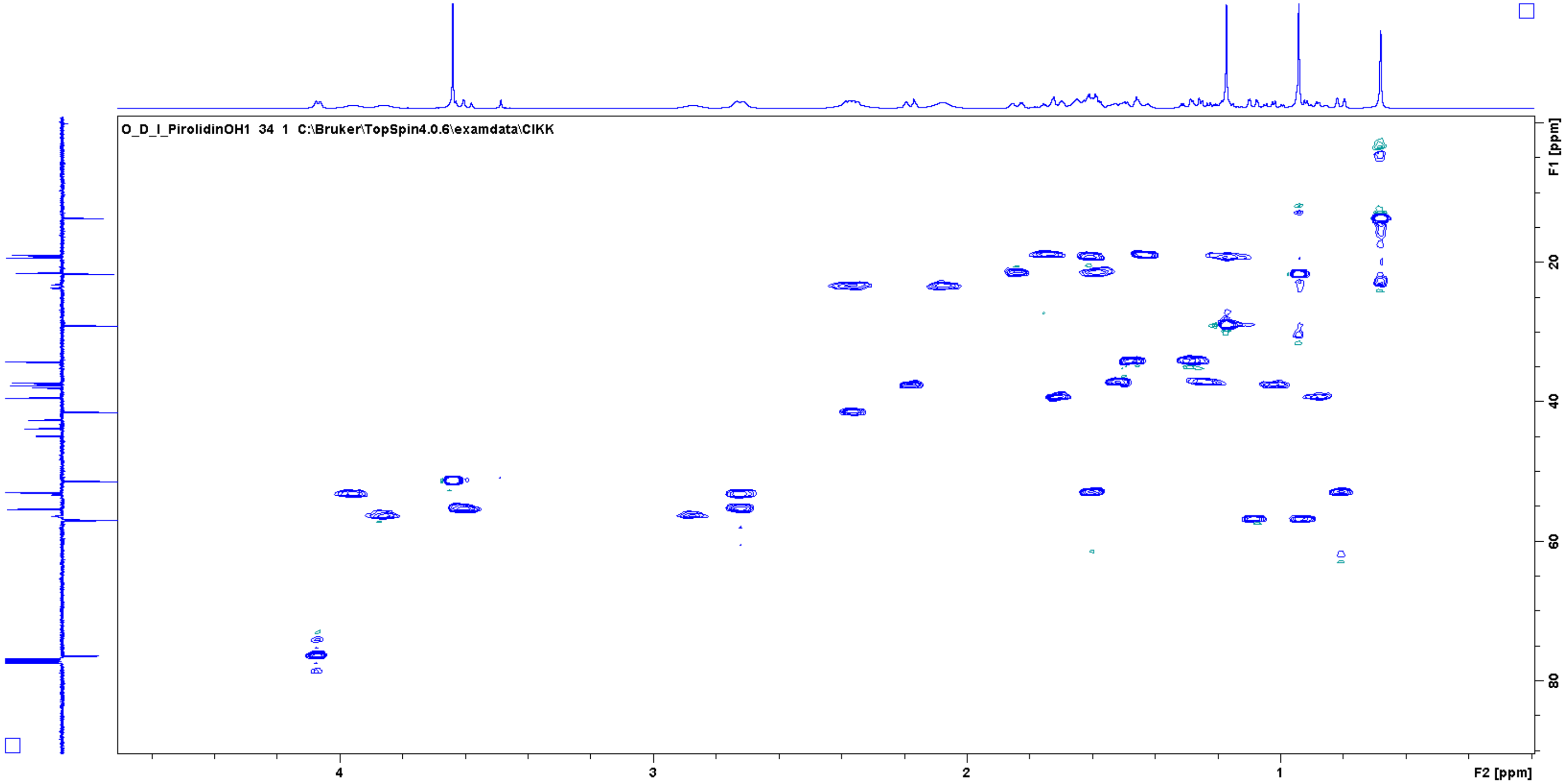

HMBC of compound (4*R*,6a*S*,8*R*,9*S*,11b*S*)-Methyl 8-hydroxy-4,9,11b-trimethyl-7-(pyrrolidin-1-ylmethyl)tetradecahydro-6a,9-methanocyclohepta[a]naphthalene-4-carboxylate (**21**)

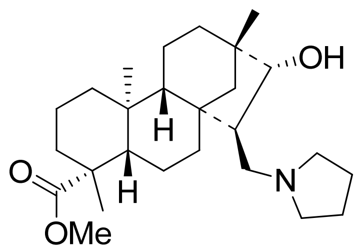

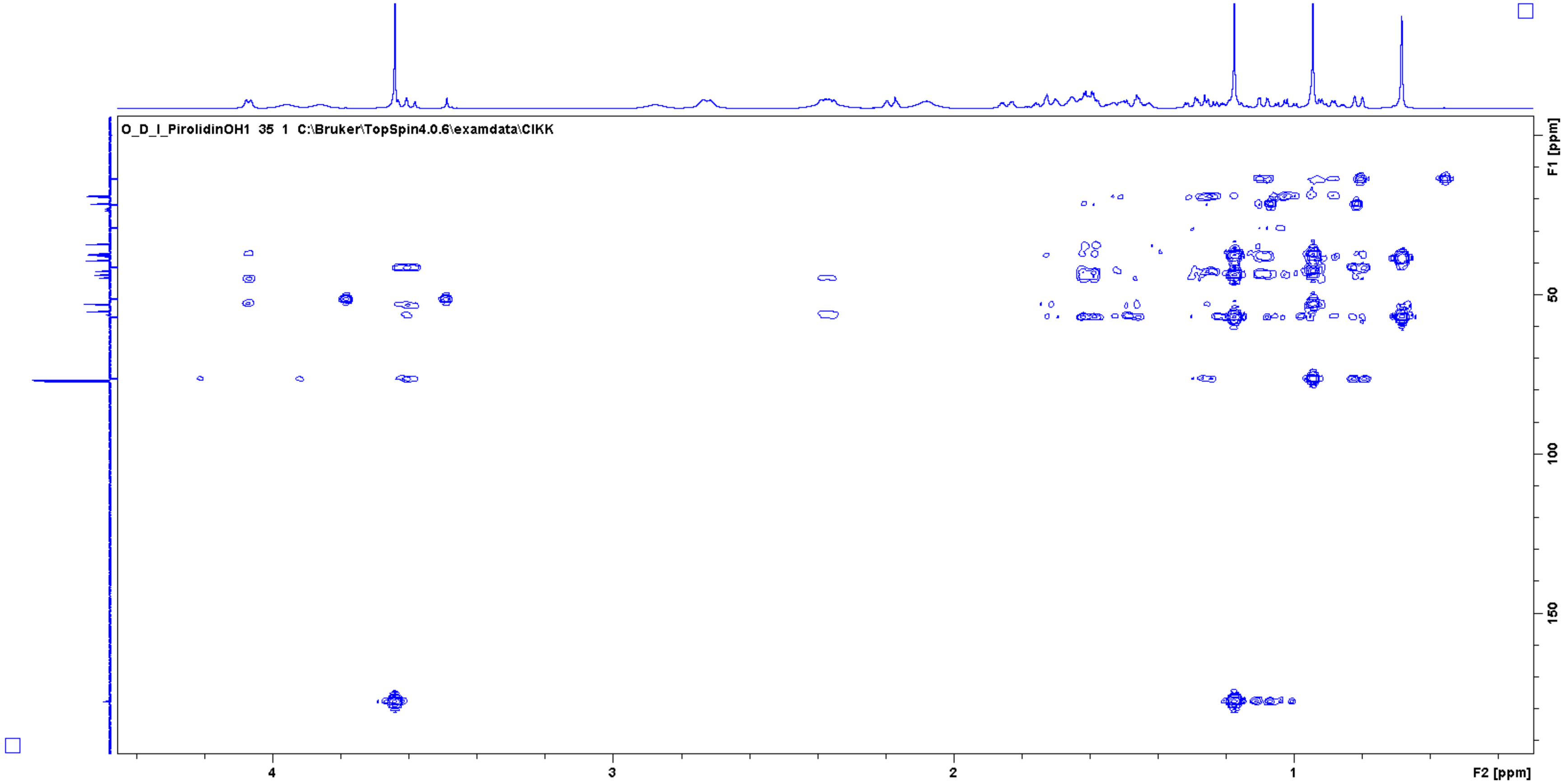

^{1}H-NMR of compound (4*R*,6a*S*,8*R*,9*S*,11b*S*)-Methyl 7-((dimethylamino)methyl)-8-hydroxy-4,9,11b-trimethyltetradecahydro-6a,9-methanocyclohepta[a]naphthalene-4-carboxylate (**22**)

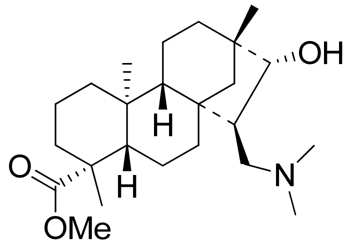

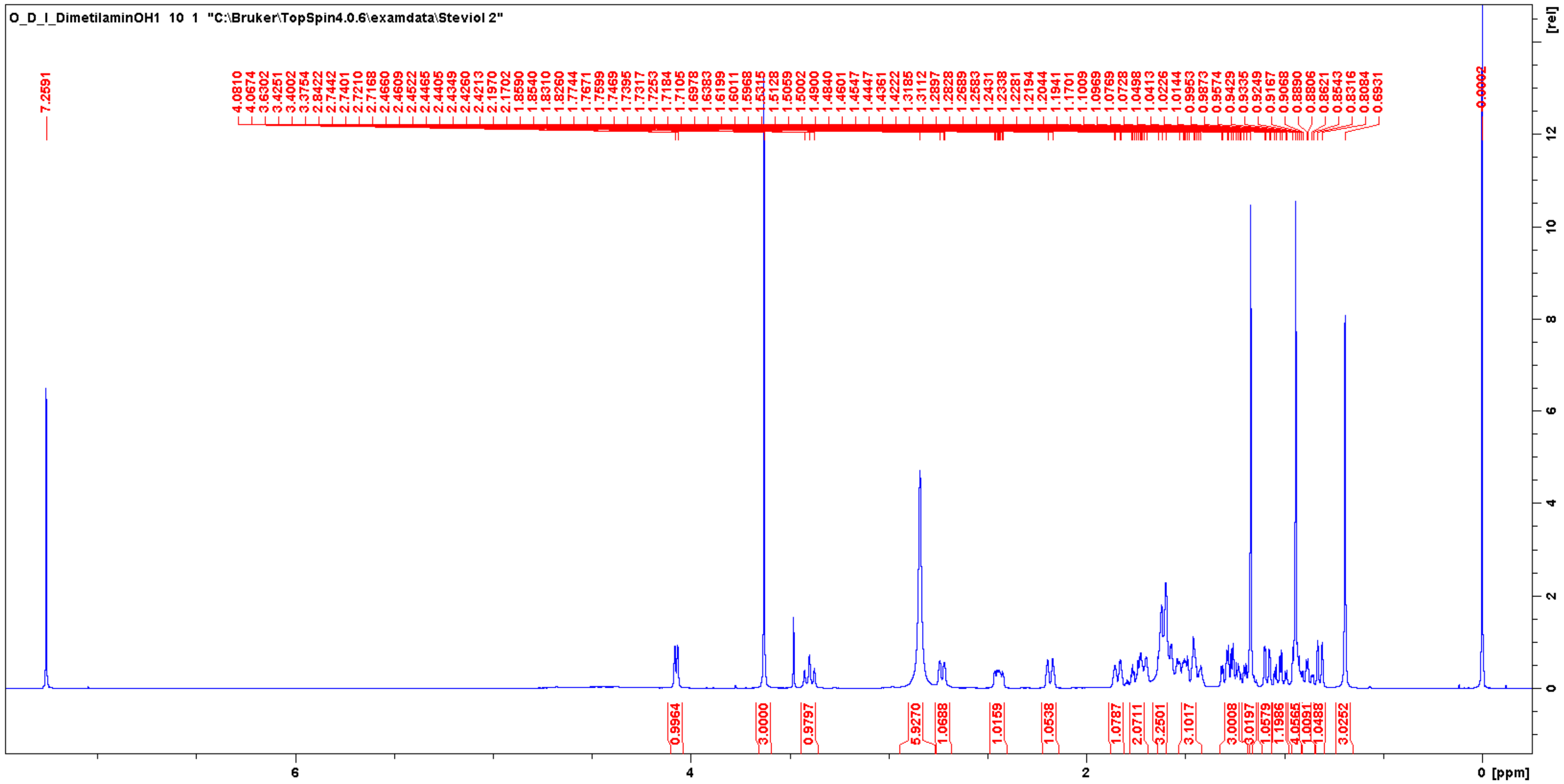

^{13}C-NMR of compound (4*R*,6a*S*,8*R*,9*S*,11b*S*)-Methyl 7-((dimethylamino)methyl)-8-hydroxy-4,9,11b-trimethyltetradecahydro-6a,9-methanocyclohepta[a]naphthalene-4-carboxylate (**22**)

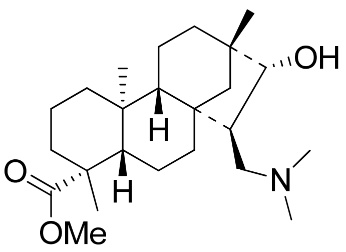

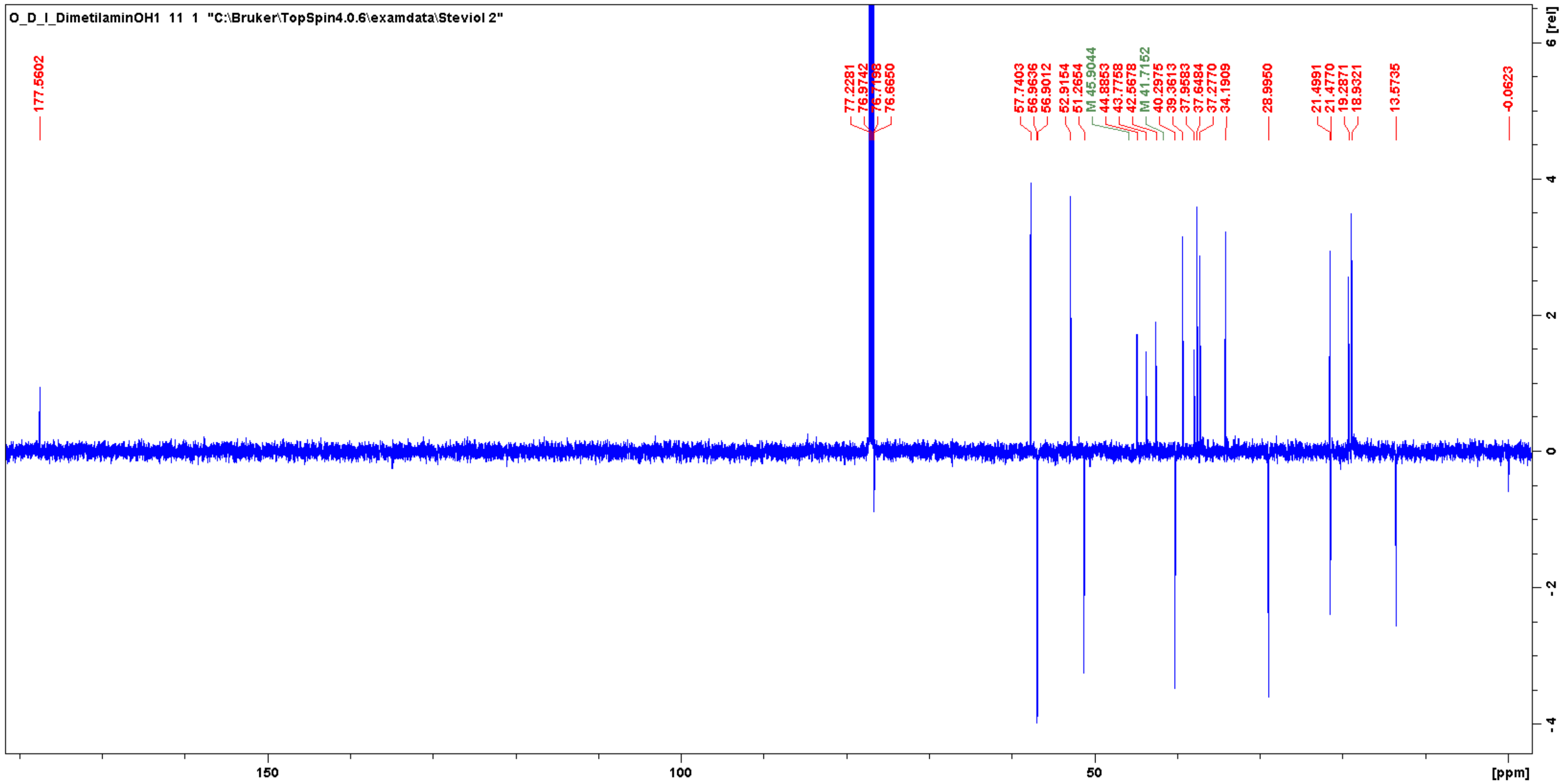

COSY of compound (4*R*,6a*S*,8*R*,9*S*,11b*S*)-Methyl 7-((dimethylamino)methyl)-8-hydroxy-4,9,11b-trimethyltetradecahydro-6a,9-methanocyclohepta[a]naphthalene-4-carboxylate (**22**)

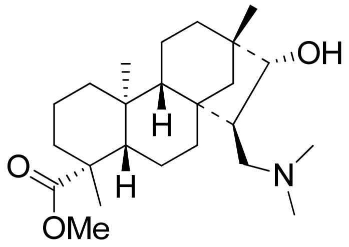

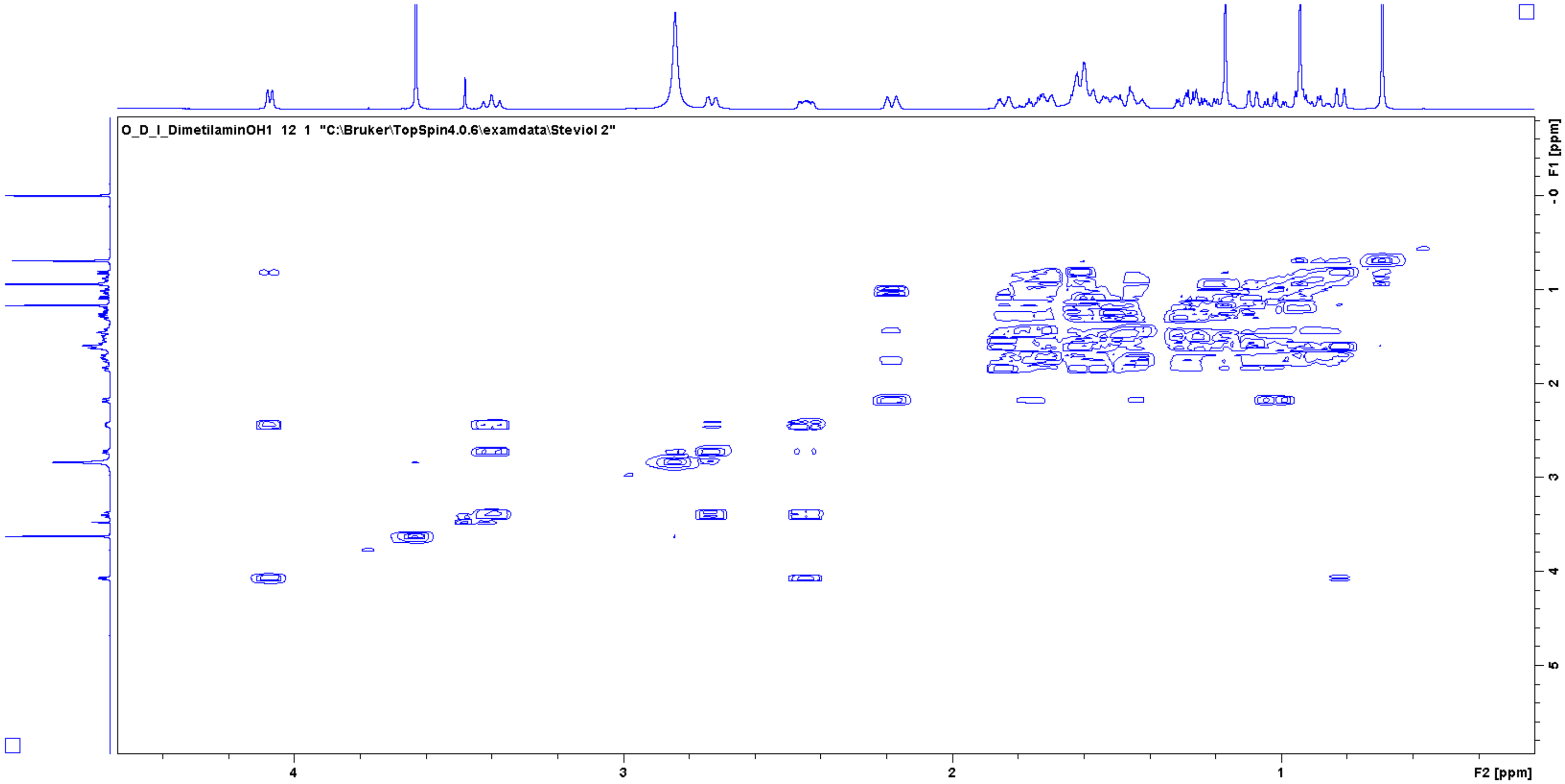

NOESY of compound (4*R*,6a*S*,8*R*,9*S*,11b*S*)-Methyl 7-((dimethylamino)methyl)-8-hydroxy-4,9,11b-trimethyltetradecahydro-6a,9-methanocyclohepta[a]naphthalene-4-carboxylate (**22**)

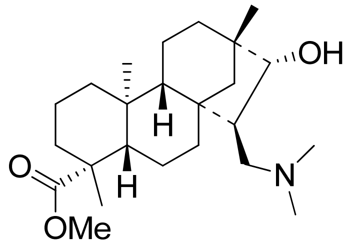

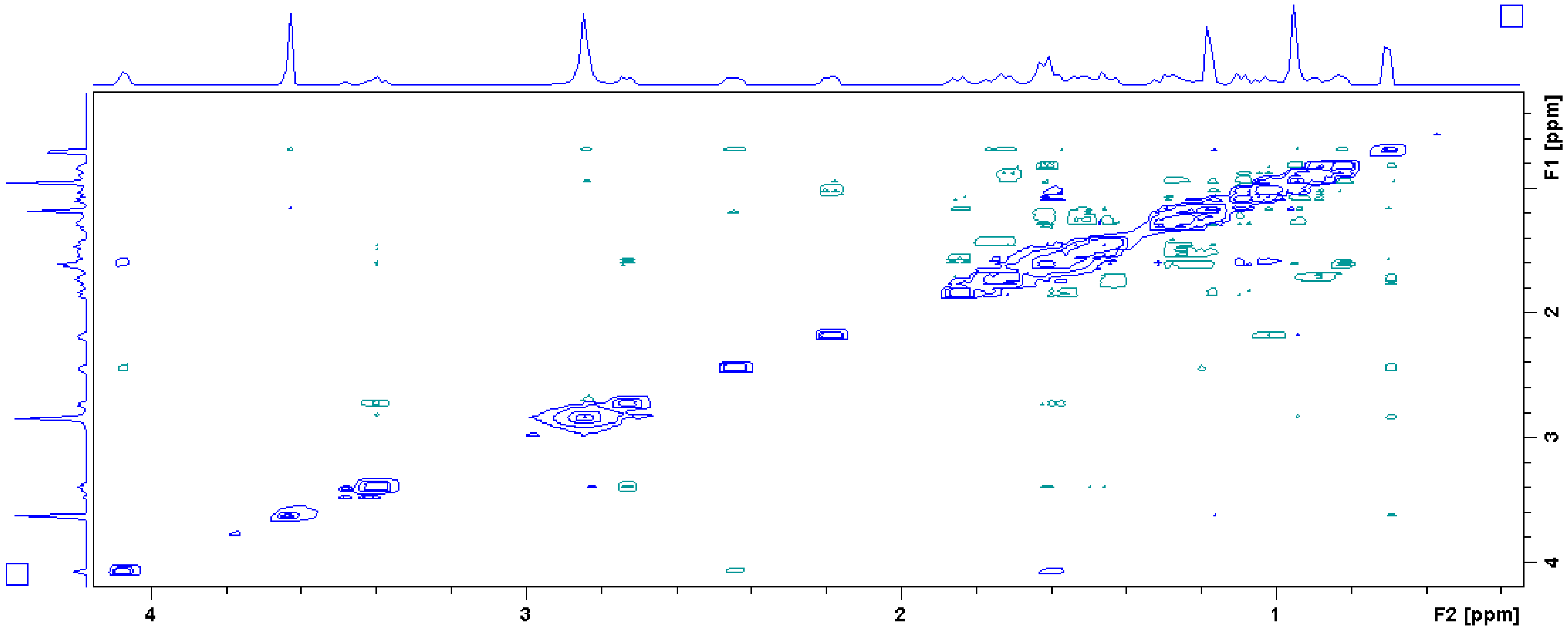

HSQC of compound (4*R*,6a*S*,8*R*,9*S*,11b*S*)-Methyl 7-((dimethylamino)methyl)-8-hydroxy-4,9,11b-trimethyltetradecahydro-6a,9-methanocyclohepta[a]naphthalene-4-carboxylate (**22**)

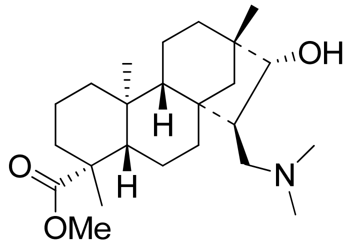

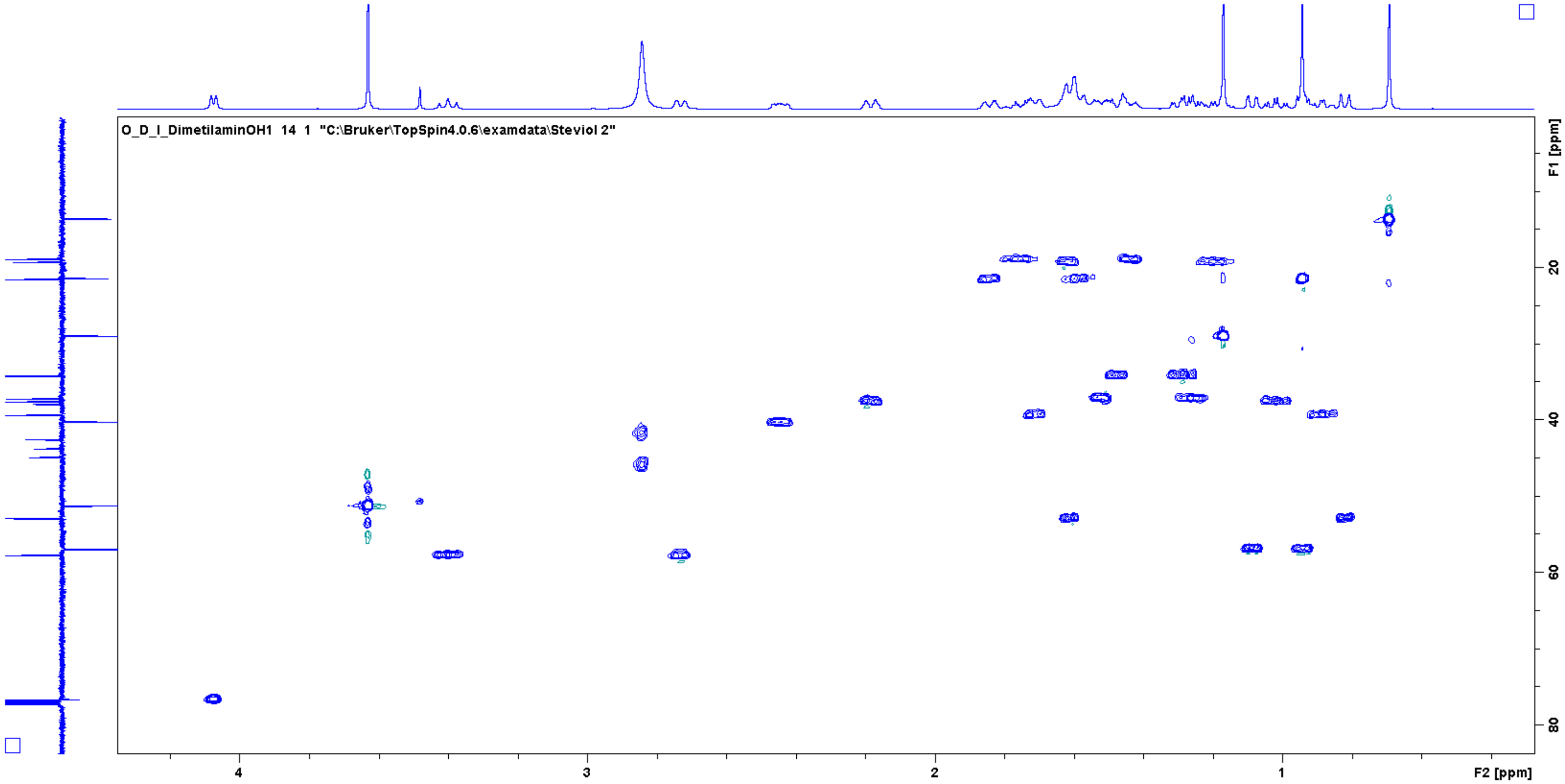

HMBC of compound (4*R*,6a*S*,8*R*,9*S*,11b*S*)-Methyl 7-((dimethylamino)methyl)-8-hydroxy-4,9,11b-trimethyltetradecahydro-6a,9-methanocyclohepta[a]naphthalene-4-carboxylate (**22**)

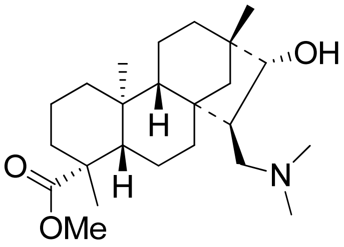

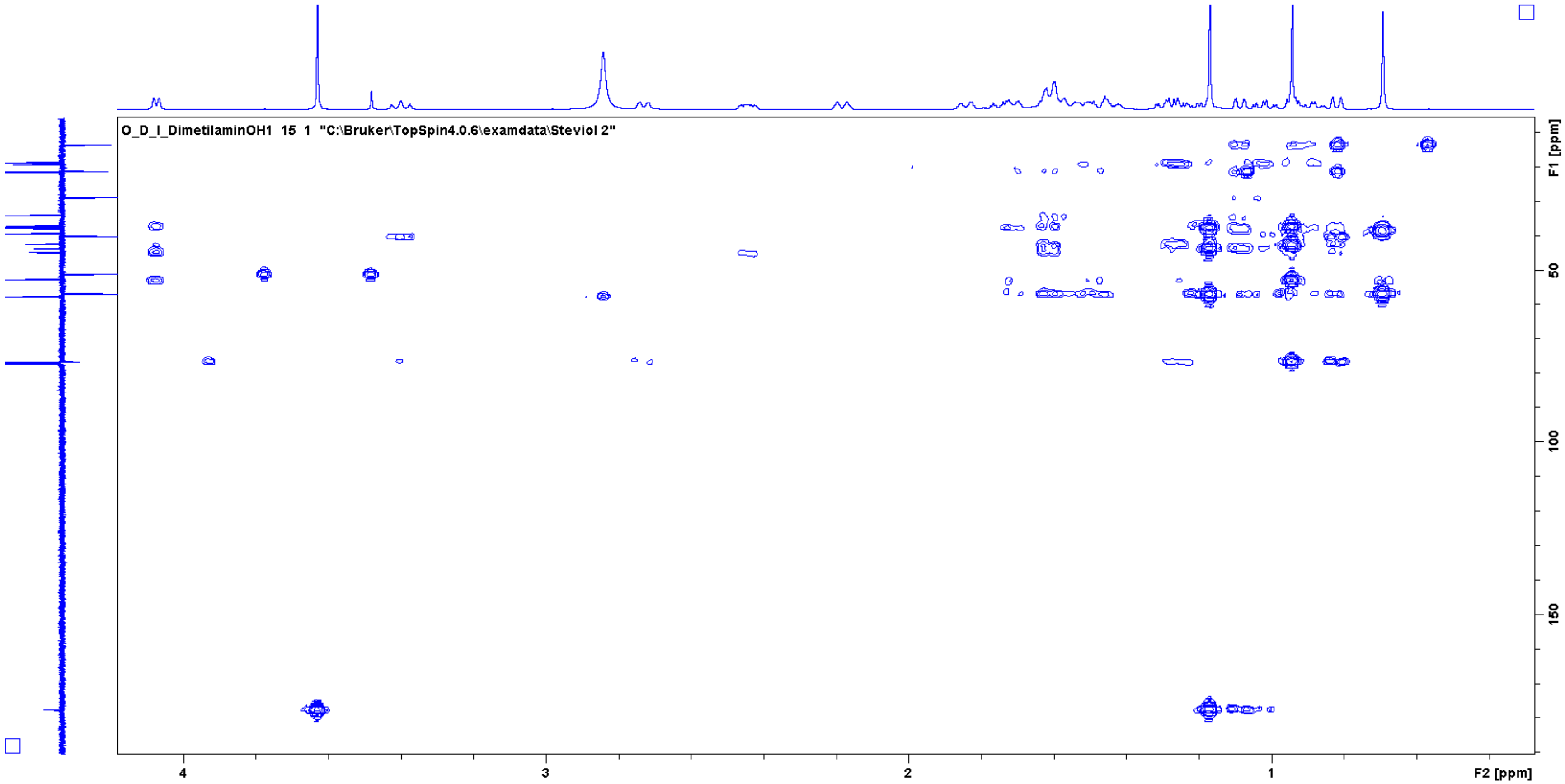

^{1}H-NMR of compound (4*R*,6a*S*,9*S*,11b*S*)-Methyl 7-((diethylamino)methyl)-8-hydroxy-4,9,11b-trimethyltetradecahydro-6a,9-methanocyclohepta[a]naphthalene-4-carboxylate (**23a**)

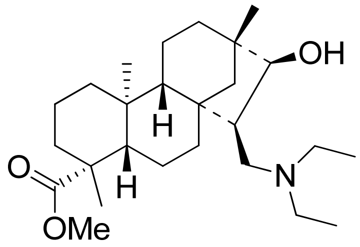

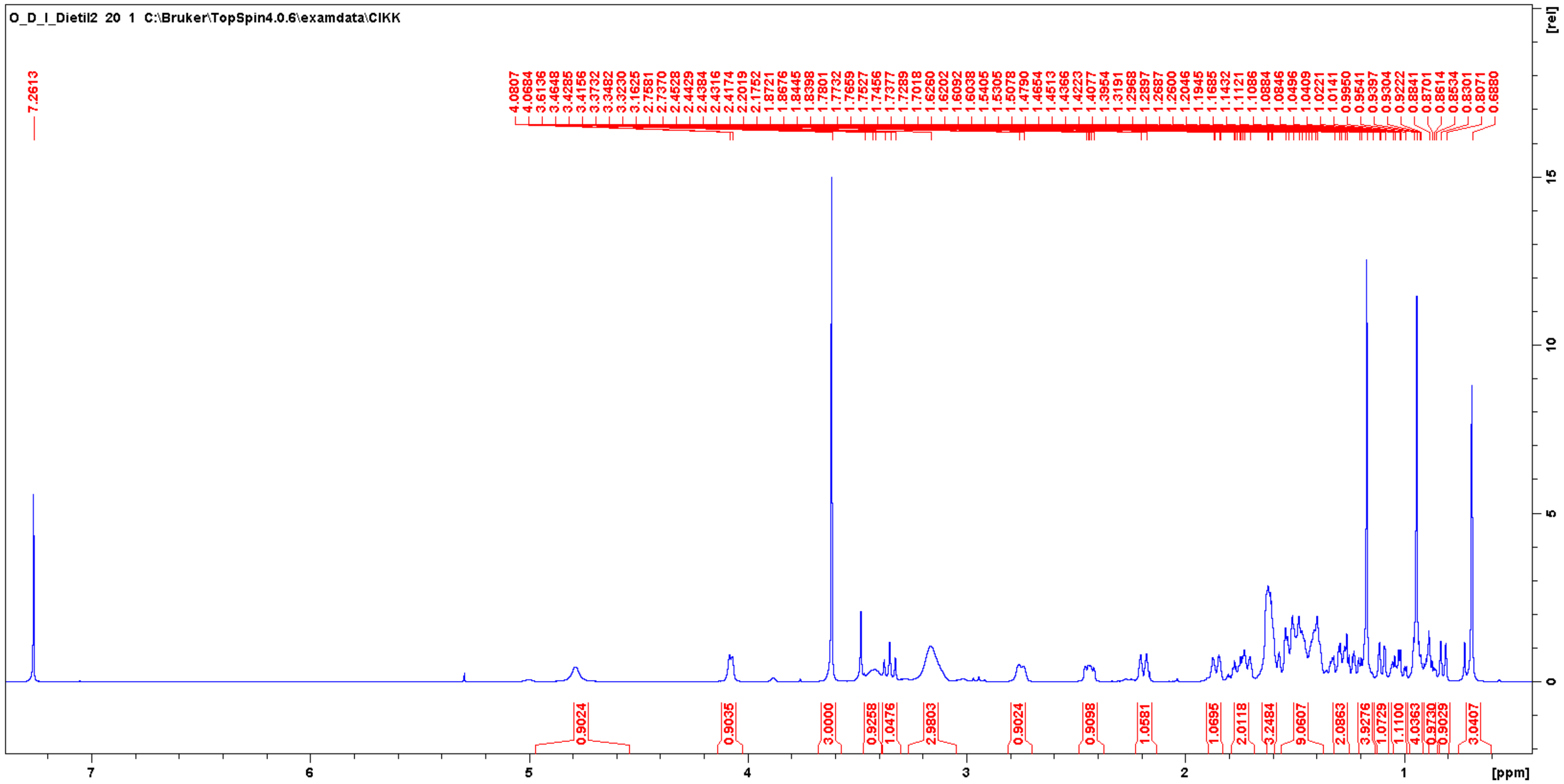

^{13}C-NMR of compound (4*R*,6a*S*,9*S*,11b*S*)-Methyl 7-((diethylamino)methyl)-8-hydroxy-4,9,11b-trimethyltetradecahydro-6a,9-methanocyclohepta[a]naphthalene-4-carboxylate (**23a**)

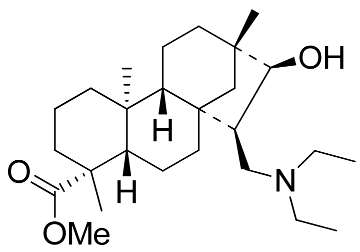

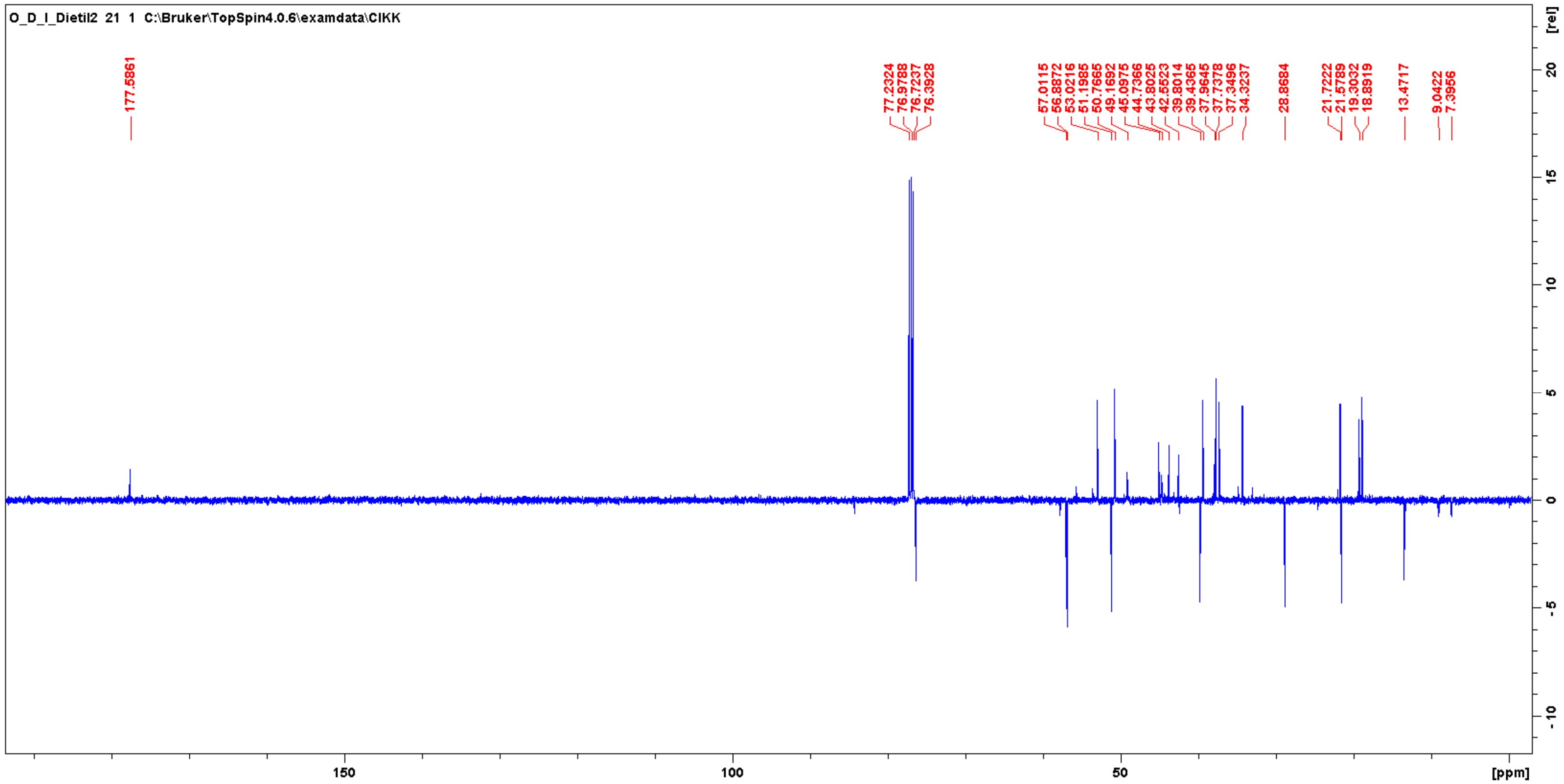

COSY of compound (4*R*,6a*S*,9*S*,11b*S*)-Methyl 7-((diethylamino)methyl)-8-hydroxy-4,9,11b-trimethyltetradecahydro-6a,9-methanocyclohepta[a]naphthalene-4-carboxylate (**23a**)

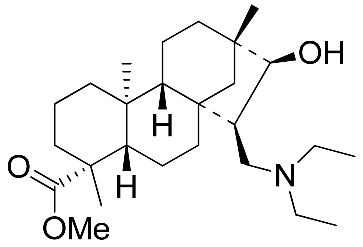

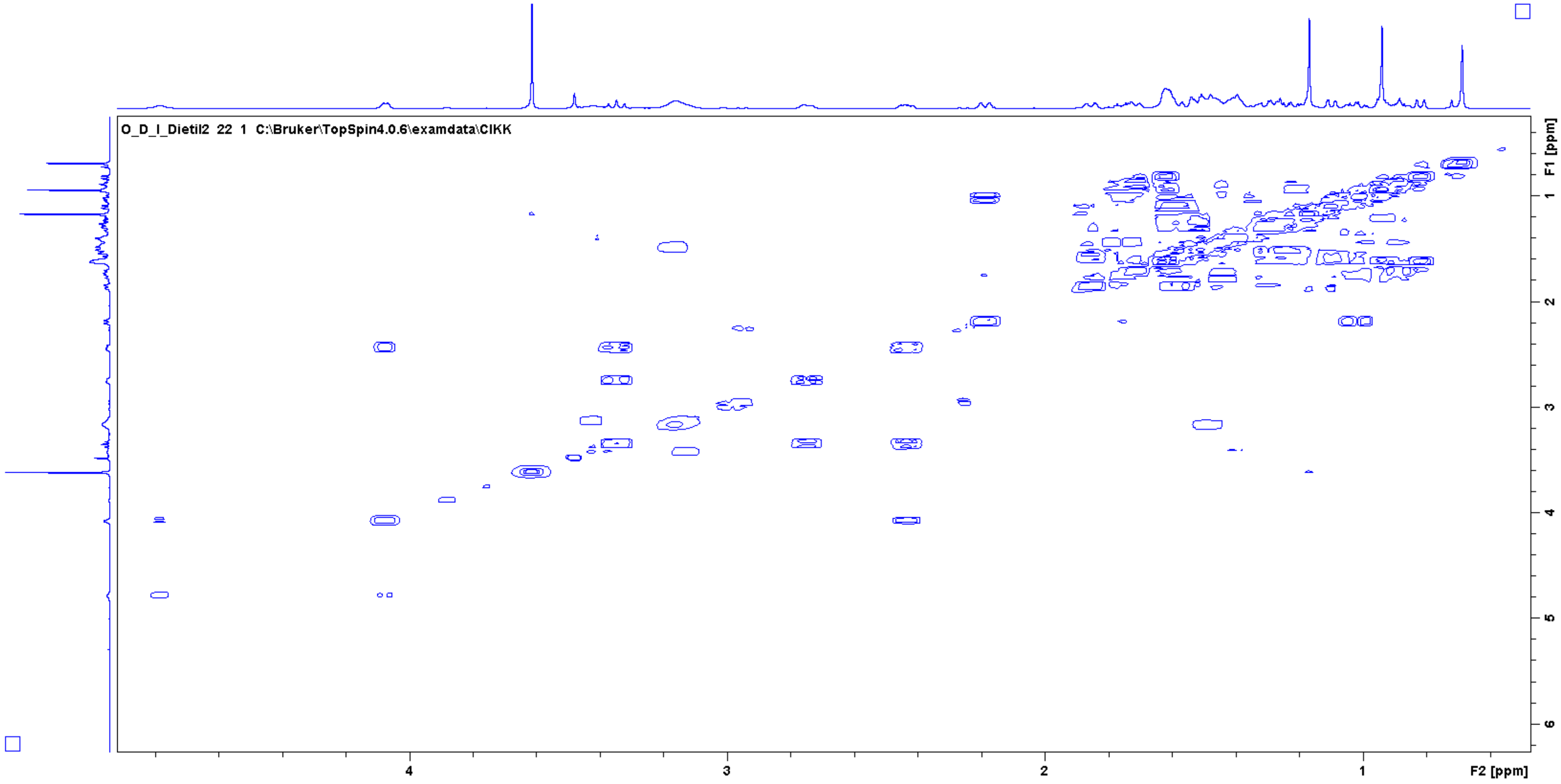

HSQC of compound (4*R*,6a*S*,9*S*,11b*S*)-Methyl 7-((diethylamino)methyl)-8-hydroxy-4,9,11b-trimethyltetradecahydro-6a,9-methanocyclohepta[a]naphthalene-4-carboxylate (**23a**)

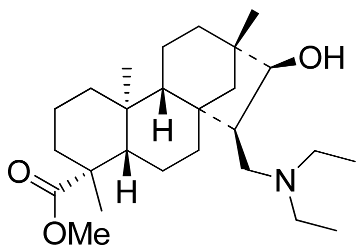

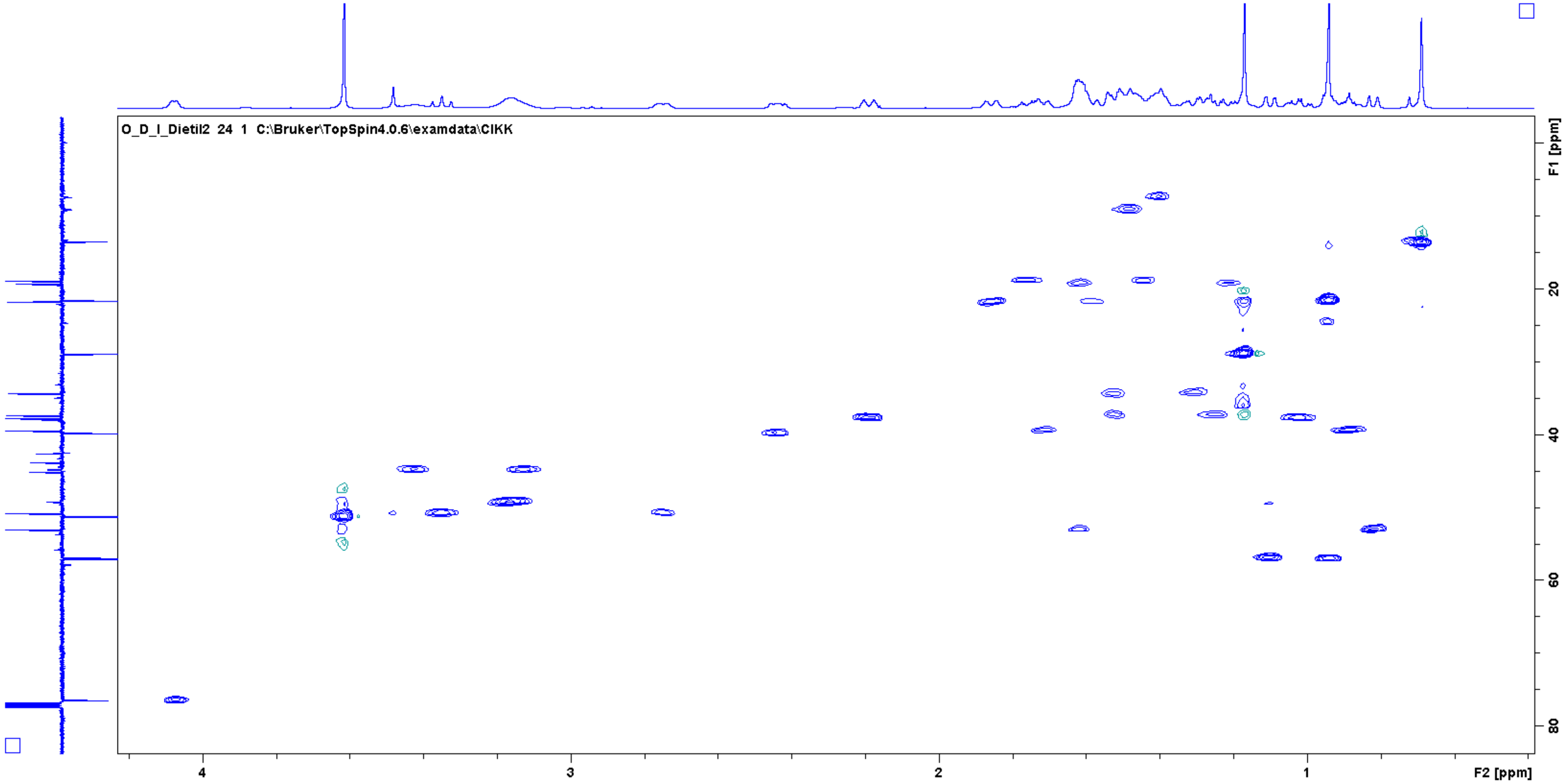

HMBC of compound (4*R*,6a*S*,9*S*,11b*S*)-Methyl 7-((diethylamino)methyl)-8-hydroxy-4,9,11b-trimethyltetradecahydro-6a,9-methanocyclohepta[a]naphthalene-4-carboxylate (**23a**)

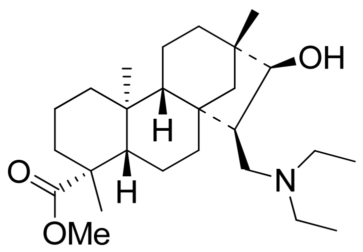

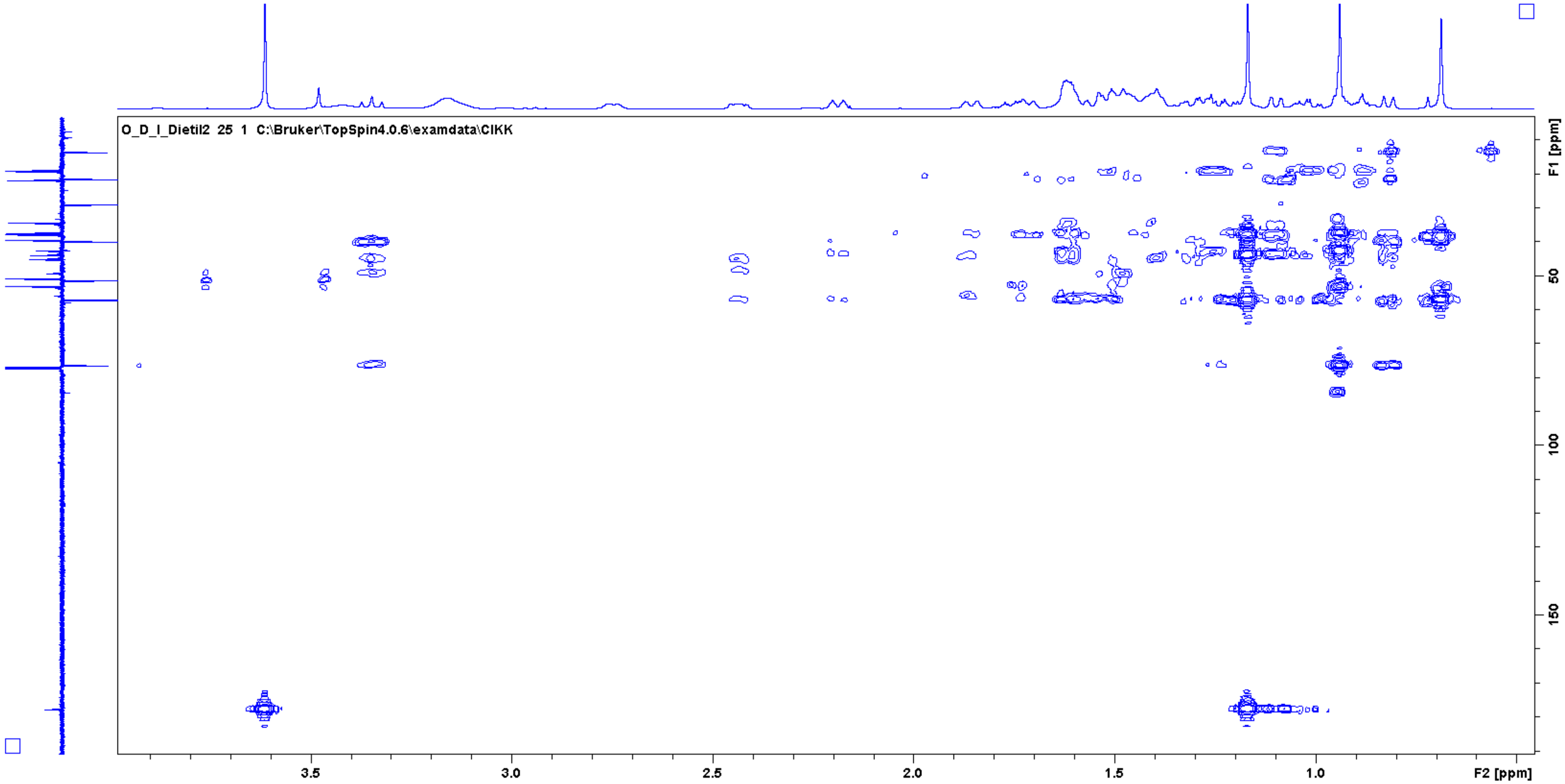

^{1}H-NMR of compound (4*R*,6a*S*,8*R*,9*S*,11b*S*)-Methyl 7-((diethylamino)methyl)-8-hydroxy-4,9,11b-trimethyltetradecahydro-6a,9-methanocyclohepta[a]naphthalene-4-carboxylate (**23b**)

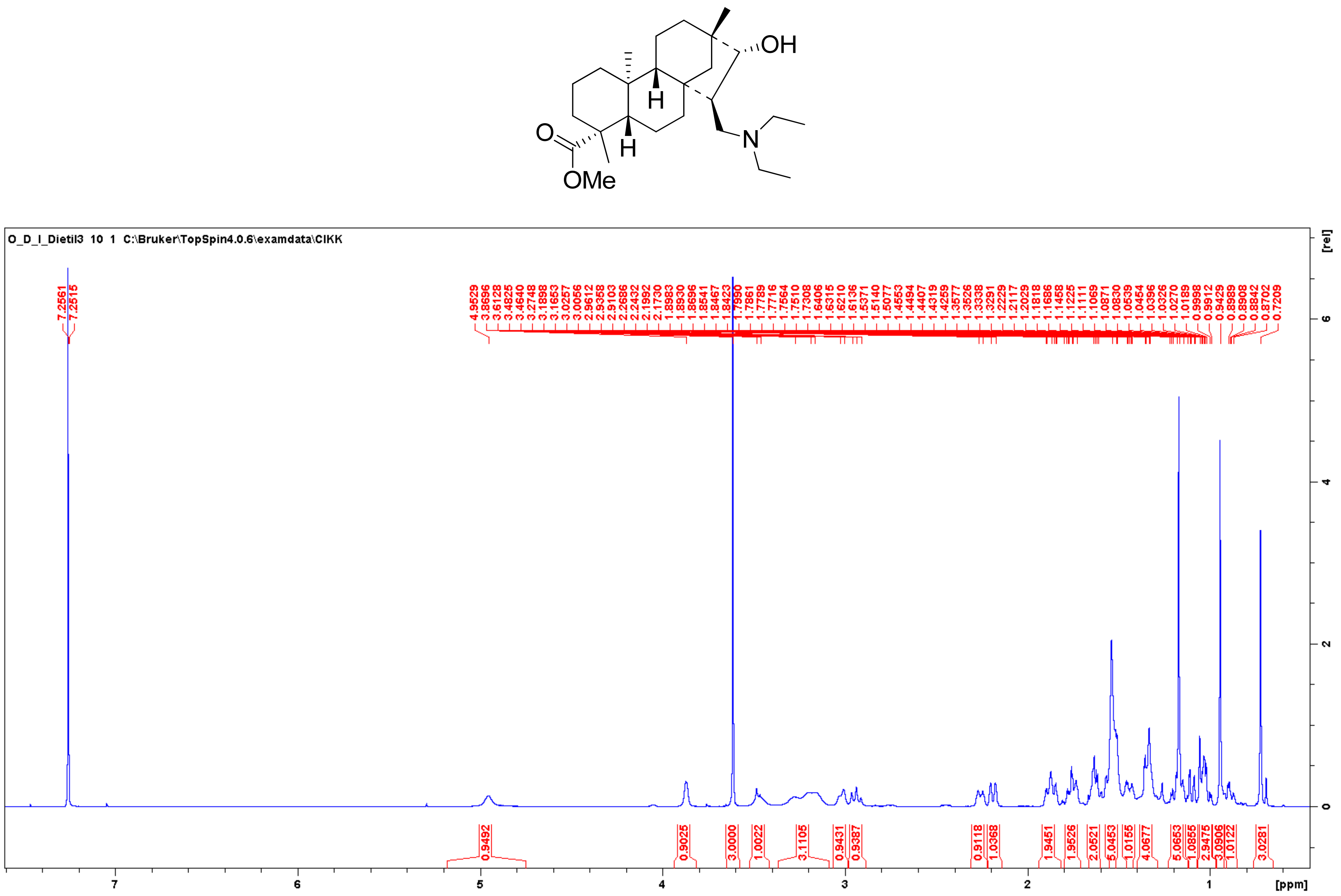

^{13}C-NMR of compound (4*R*,6a*S*,8*R*,9*S*,11b*S*)-Methyl 7-((diethylamino)methyl)-8-hydroxy-4,9,11b-trimethyltetradecahydro-6a,9-methanocyclohepta[a]naphthalene-4-carboxylate (**23b**)

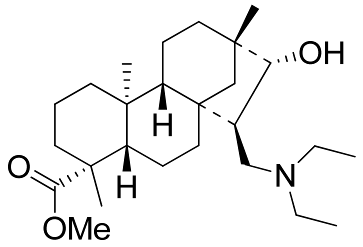

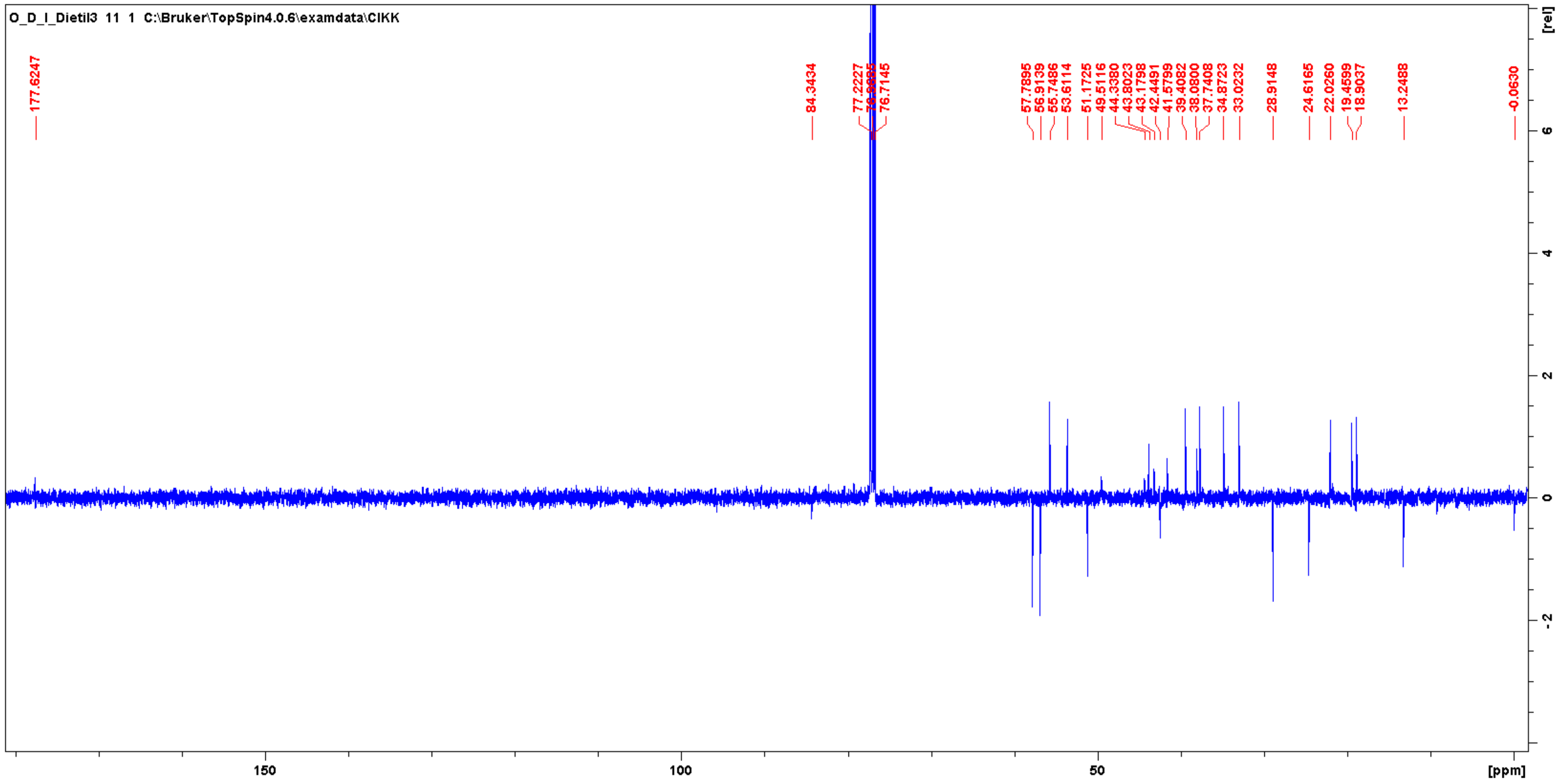

COSY of compound (4*R*,6a*S*,8*R*,9*S*,11b*S*)-Methyl 7-((diethylamino)methyl)-8-hydroxy-4,9,11b-trimethyltetradecahydro-6a,9-methanocyclohepta[a]naphthalene-4-carboxylate (**23b**)

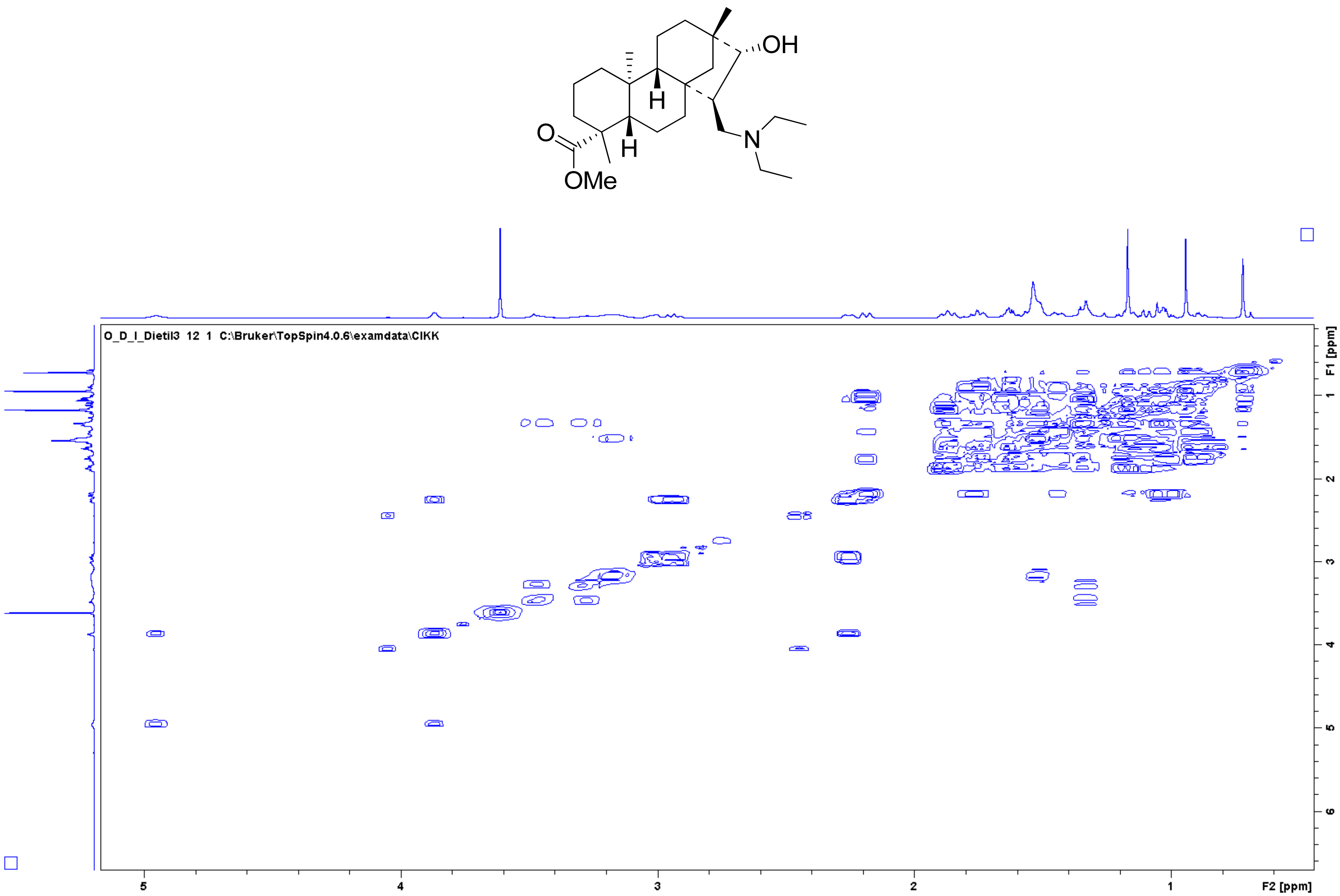

HSQC of compound (4*R*,6a*S*,8*R*,9*S*,11b*S*)-Methyl 7-((diethylamino)methyl)-8-hydroxy-4,9,11b-trimethyltetradecahydro-6a,9-methanocyclohepta[a]naphthalene-4-carboxylate (**23b**)

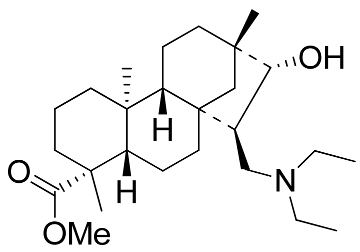

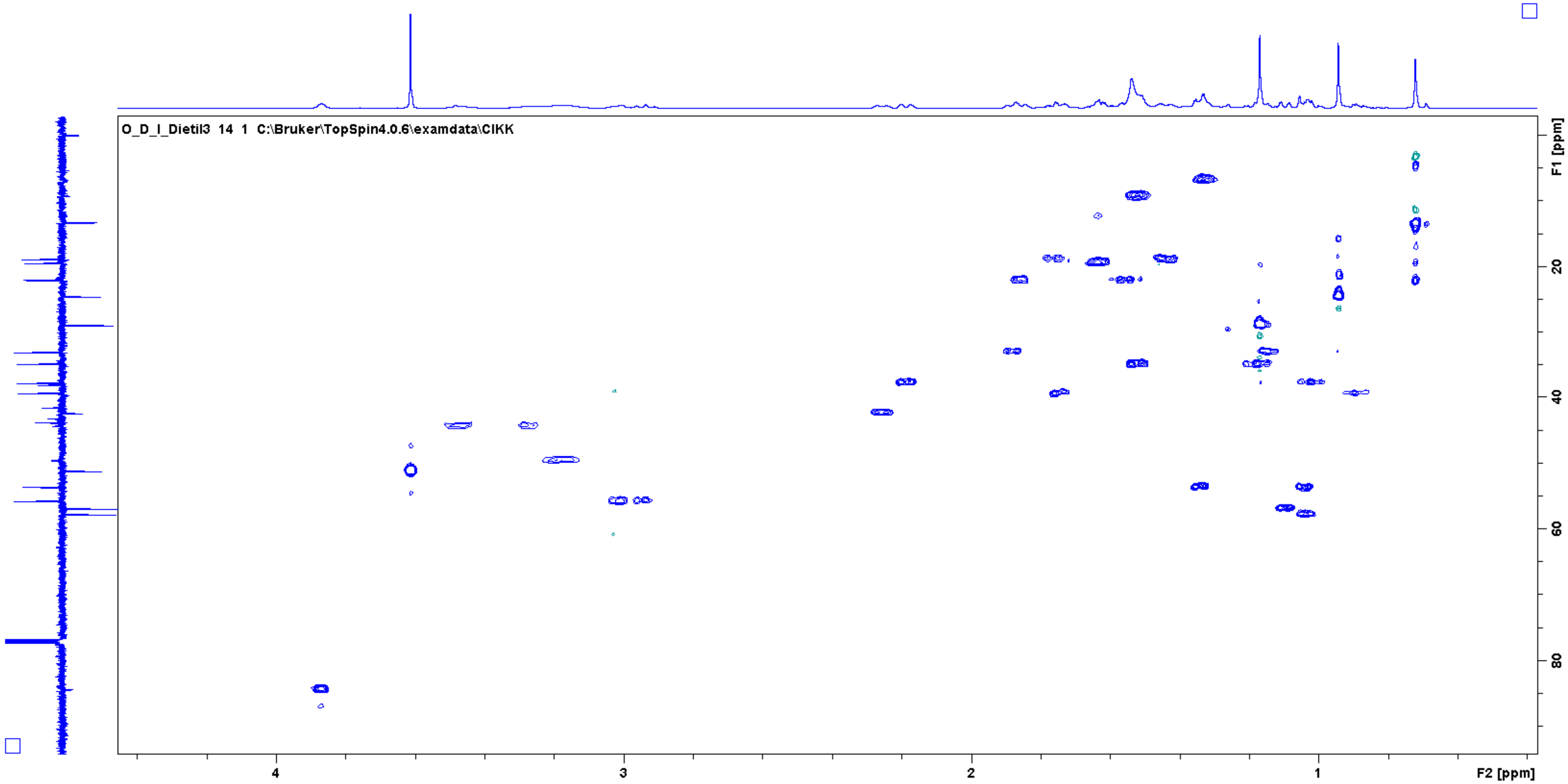

HMBC of compound (4*R*,6a*S*,8*R*,9*S*,11b*S*)-Methyl 7-((diethylamino)methyl)-8-hydroxy-4,9,11b-trimethyltetradecahydro-6a,9-methanocyclohepta[a]naphthalene-4-carboxylate (**23b**)

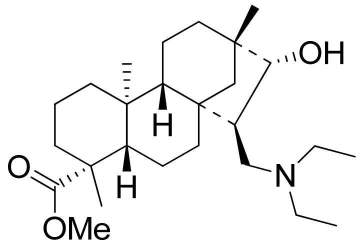

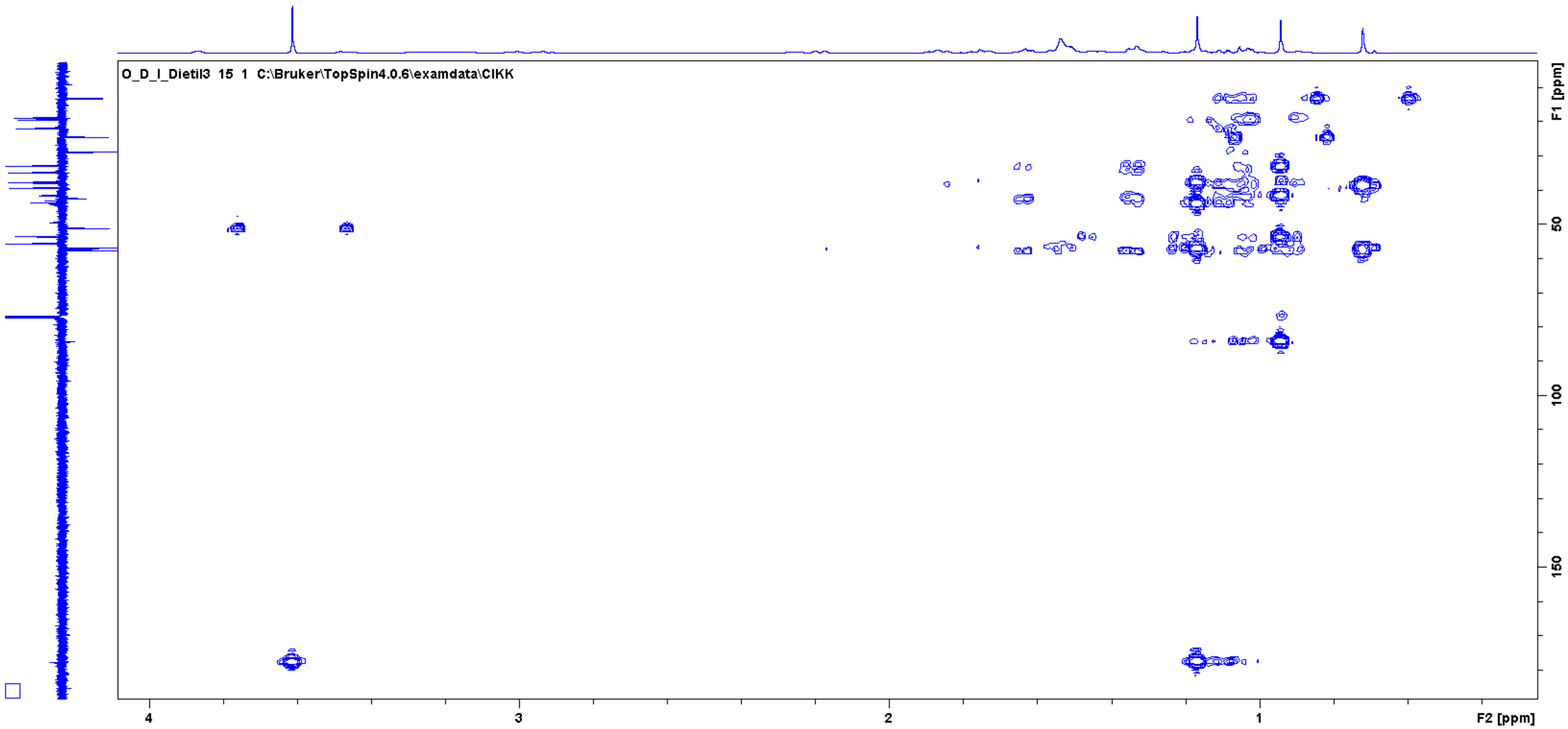

^{1}H-NMR of compound (4*R*,6a*S*,9*S*,11b*S*)-Methyl 8-hydroxy-4,9,11b-trimethyl-7-((methylamino)methyl)tetradecahydro-6a,9-methanocyclohepta[a]naphthalene-4-carboxylate (**24**)

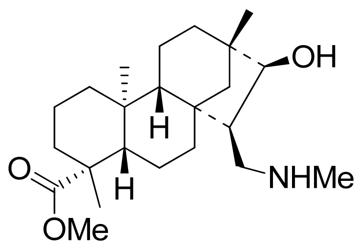

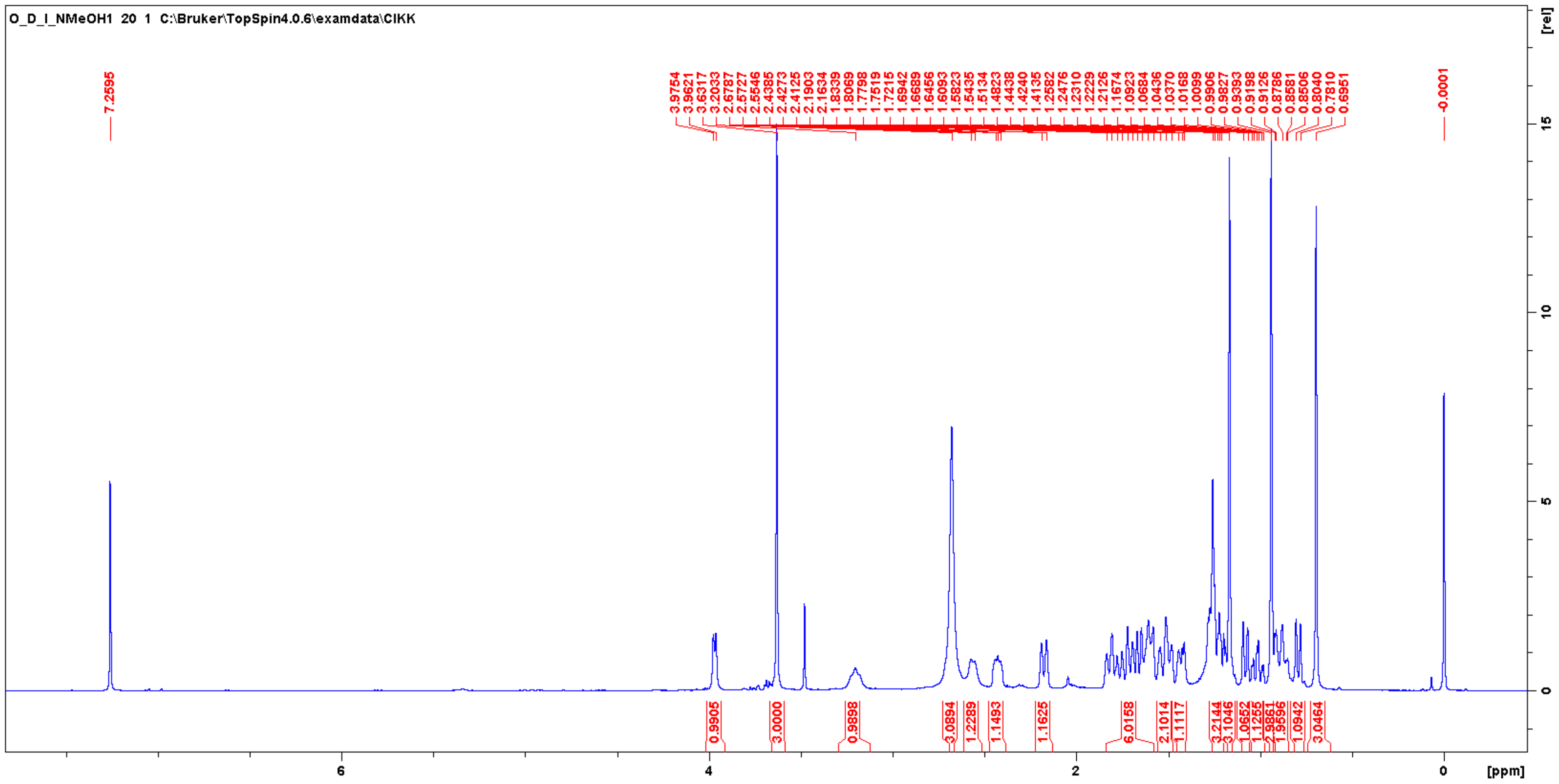

^{13}C-NMR of compound (4*R*,6a*S*,9*S*,11b*S*)-Methyl 8-hydroxy-4,9,11b-trimethyl-7-((methylamino)methyl)tetradecahydro-6a,9-methanocyclohepta[a]naphthalene-4-carboxylate (**24**)

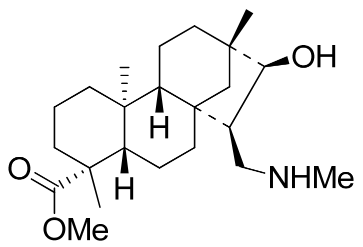

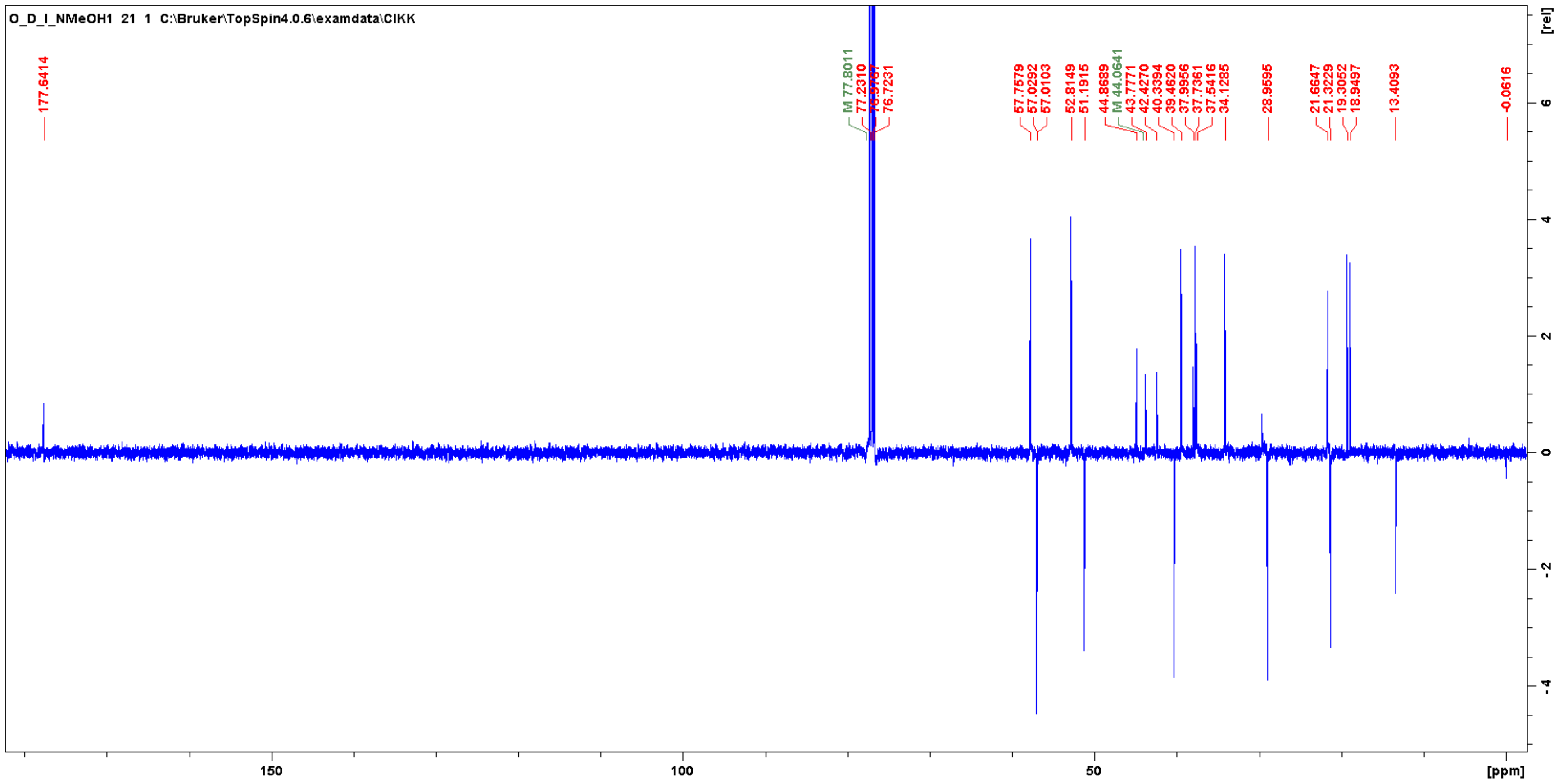

COSY of compound (4*R*,6a*S*,9*S*,11b*S*)-Methyl 8-hydroxy-4,9,11b-trimethyl-7-((methylamino)methyl)tetradecahydro-6a,9-methanocyclohepta[a]naphthalene-4-carboxylate (**24**)

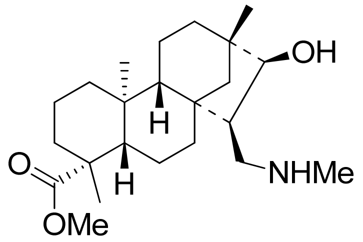

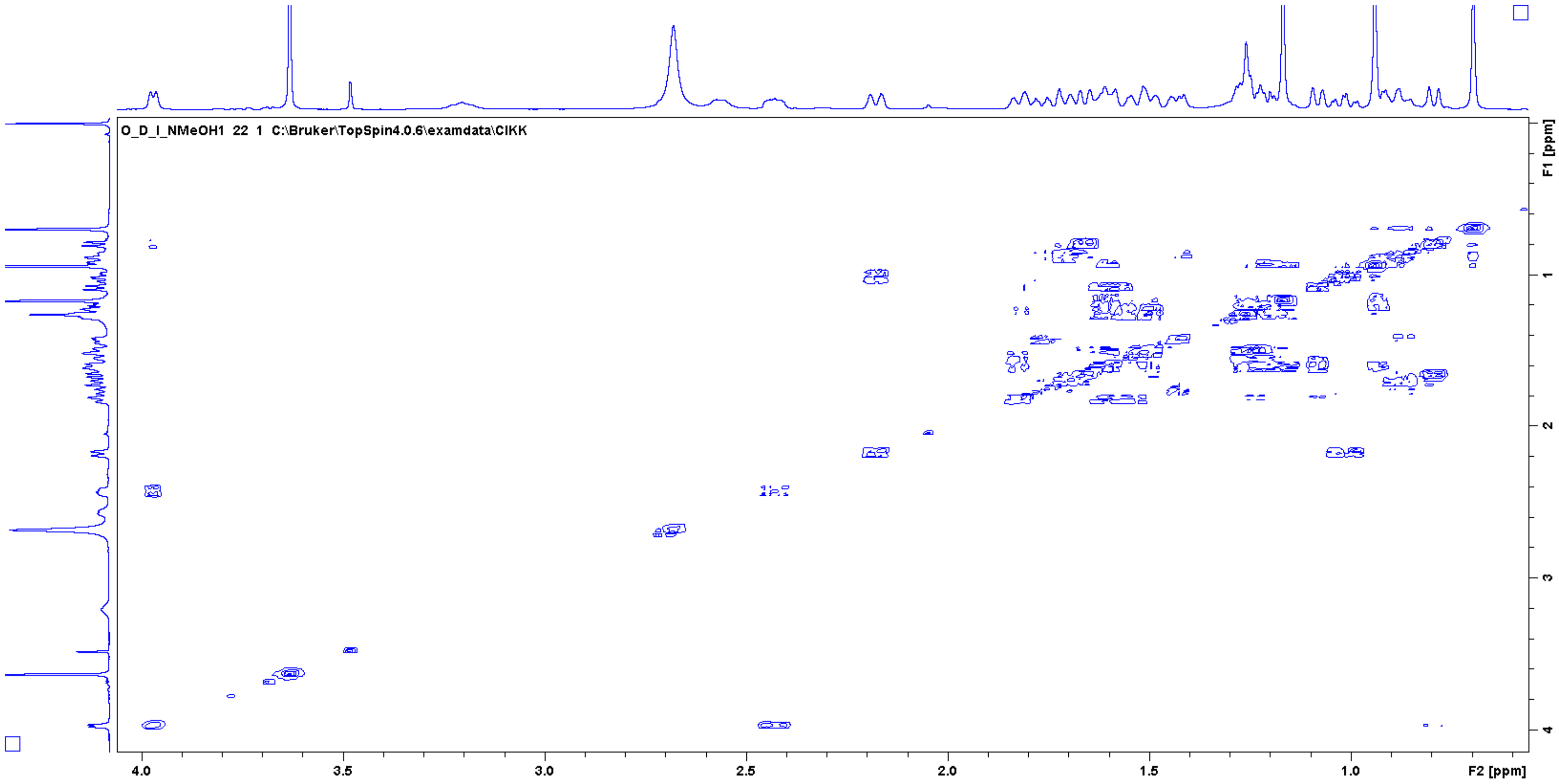

NOESY of compound (4*R*,6a*S*,9*S*,11b*S*)-Methyl 8-hydroxy-4,9,11b-trimethyl-7-((methylamino)methyl)tetradecahydro-6a,9-methanocyclohepta[a]naphthalene-4-carboxylate (**24**)

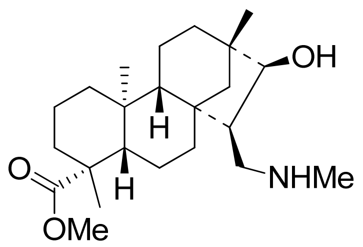

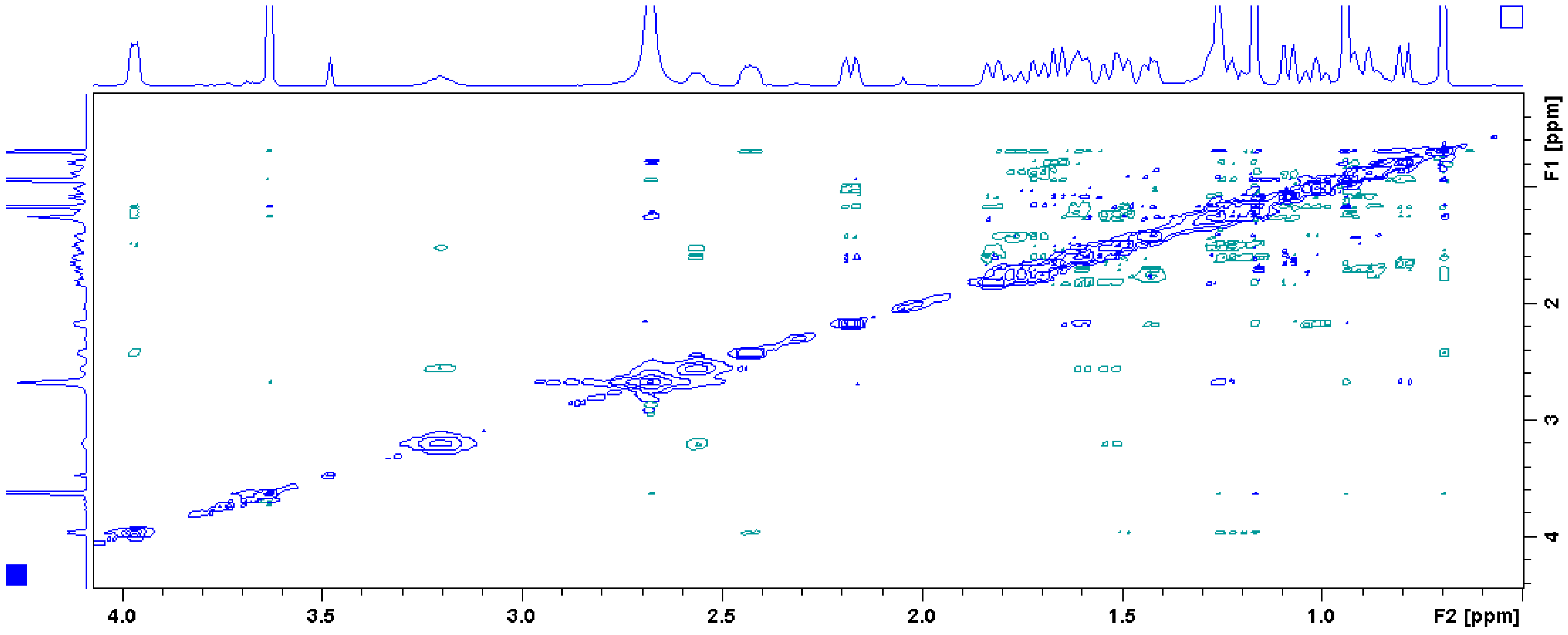

HSQC of compound (4*R*,6a*S*,9*S*,11b*S*)-Methyl 8-hydroxy-4,9,11b-trimethyl-7-((methylamino)methyl)tetradecahydro-6a,9-methanocyclohepta[a]naphthalene-4-carboxylate (**24**)

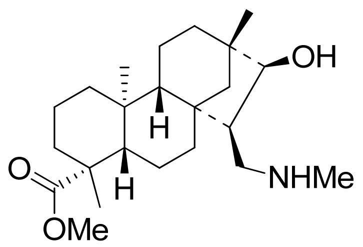

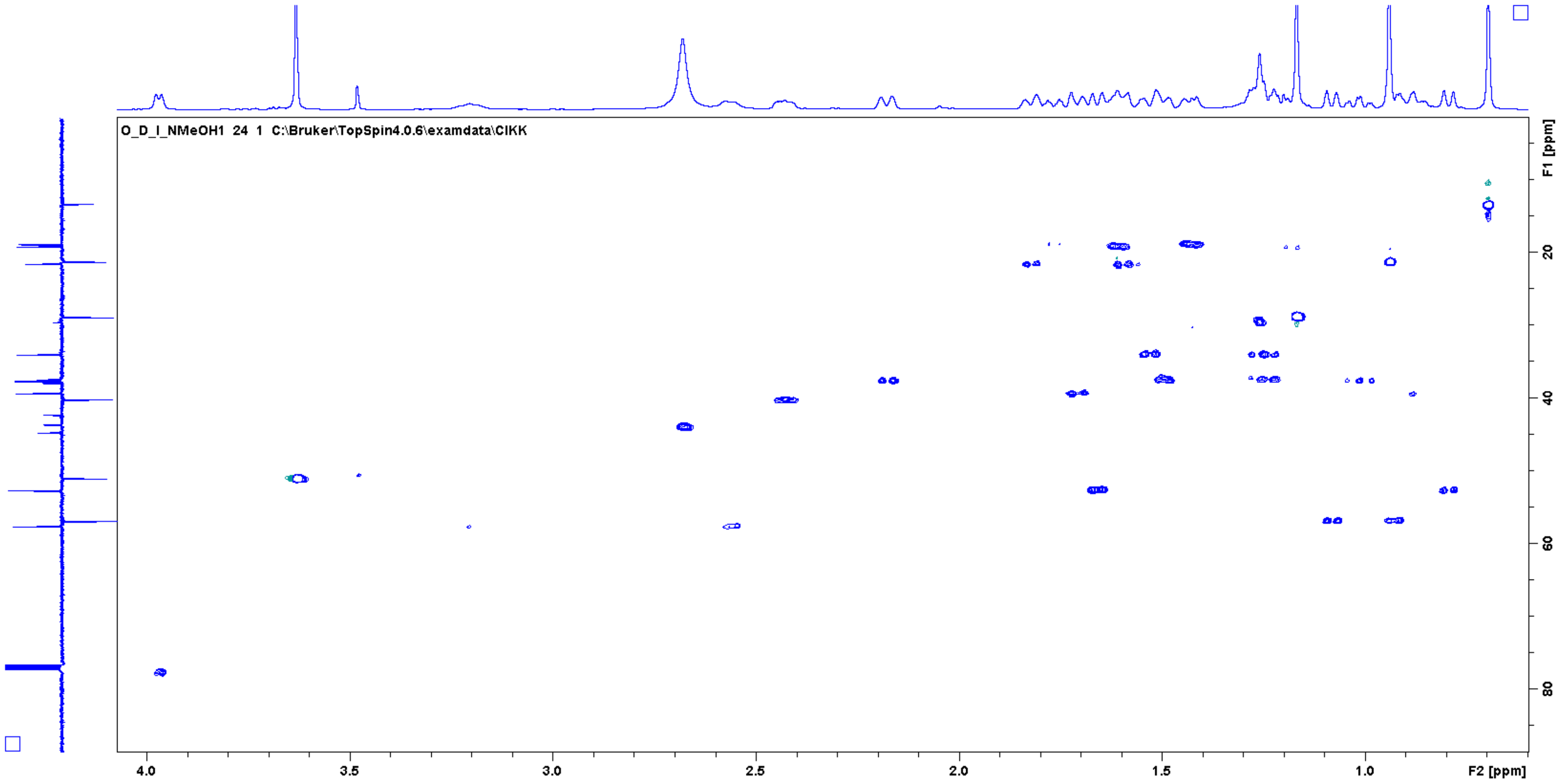

HMBC of compound (4*R*,6a*S*,9*S*,11b*S*)-Methyl 8-hydroxy-4,9,11b-trimethyl-7-((methylamino)methyl)tetradecahydro-6a,9-methanocyclohepta[a]naphthalene-4-carboxylate (**24**)

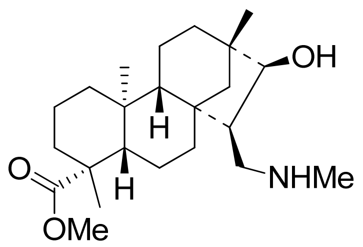

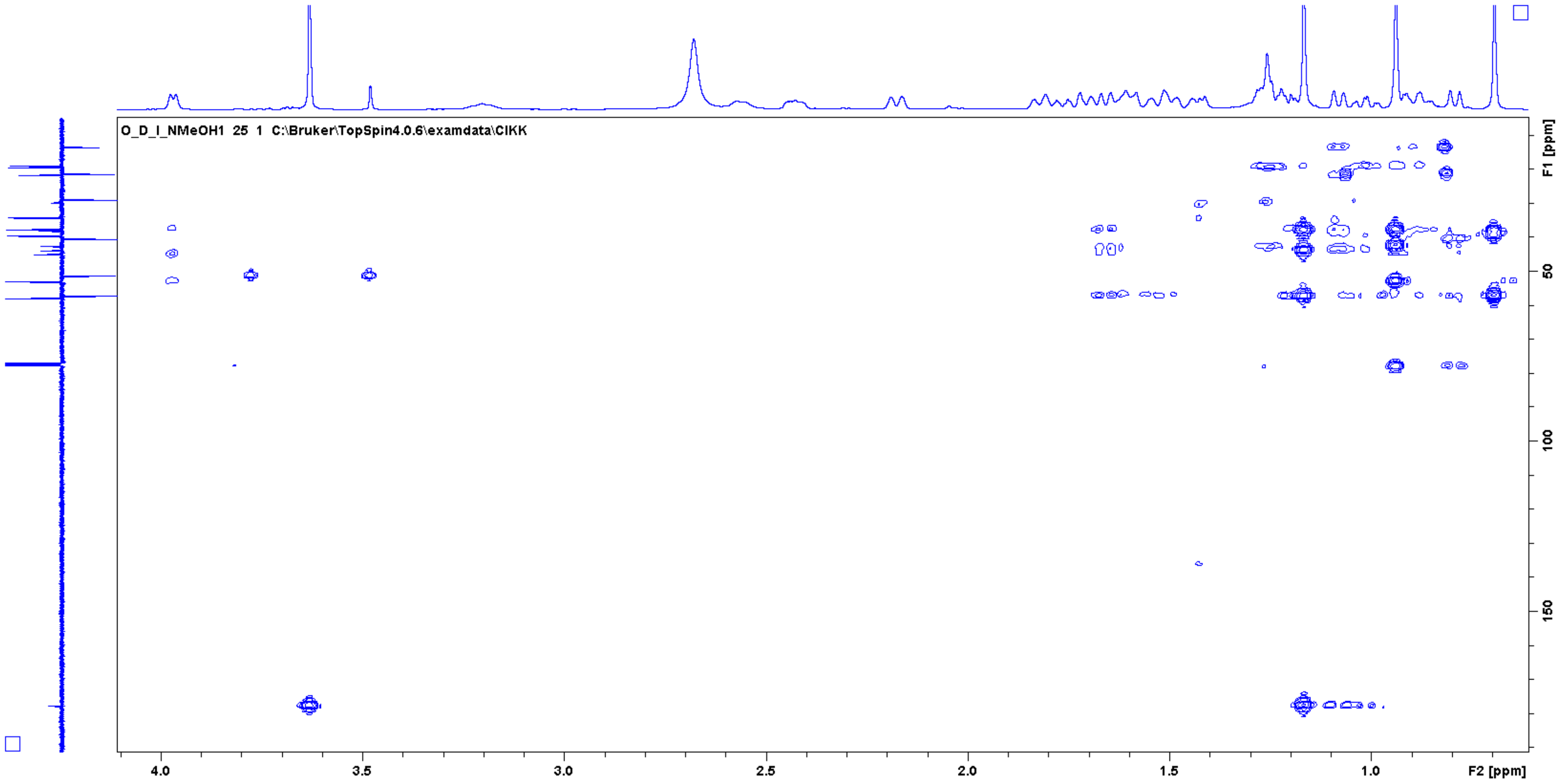

^{1}H-NMR of compound (4*R*,6a*S*,8*R*,9*S*,11b*S*)-Methyl 8-hydroxy-4,9,11b-trimethyl-7-((3-phenylthioureido)methyl)tetradecahydro-6a,9-methanocyclohepta[a]naphthalene-4-carboxylate (**25**)

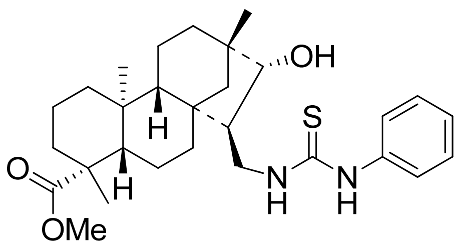

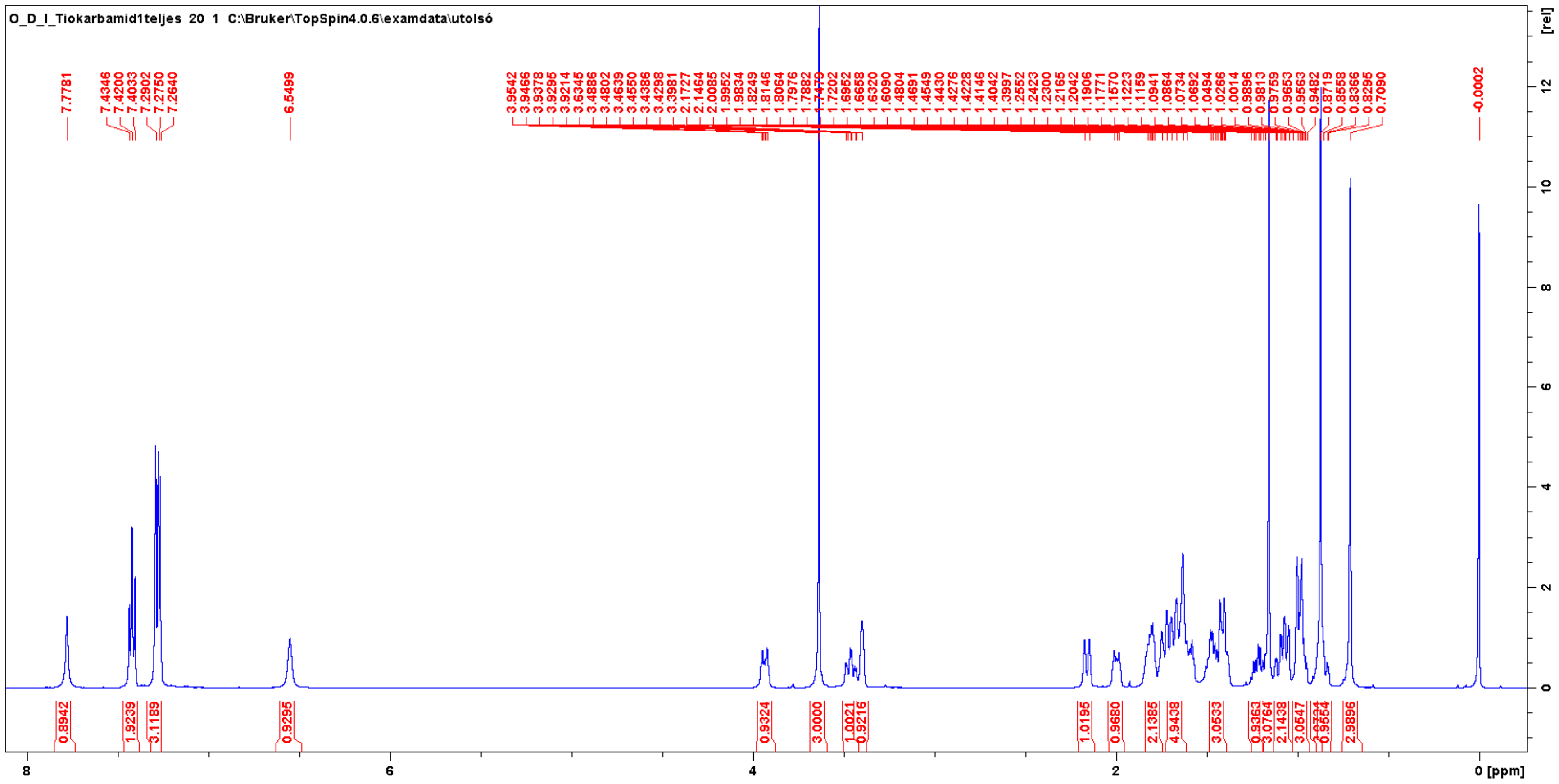

^{13}C-NMR of compound (4*R*,6a*S*,8*R*,9*S*,11b*S*)-Methyl 8-hydroxy-4,9,11b-trimethyl-7-((3-phenylthioureido)methyl)tetradecahydro-6a,9-methanocyclohepta[a]naphthalene-4-carboxylate (**25**)

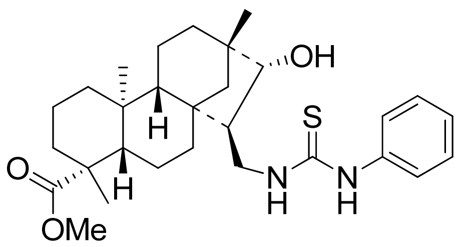

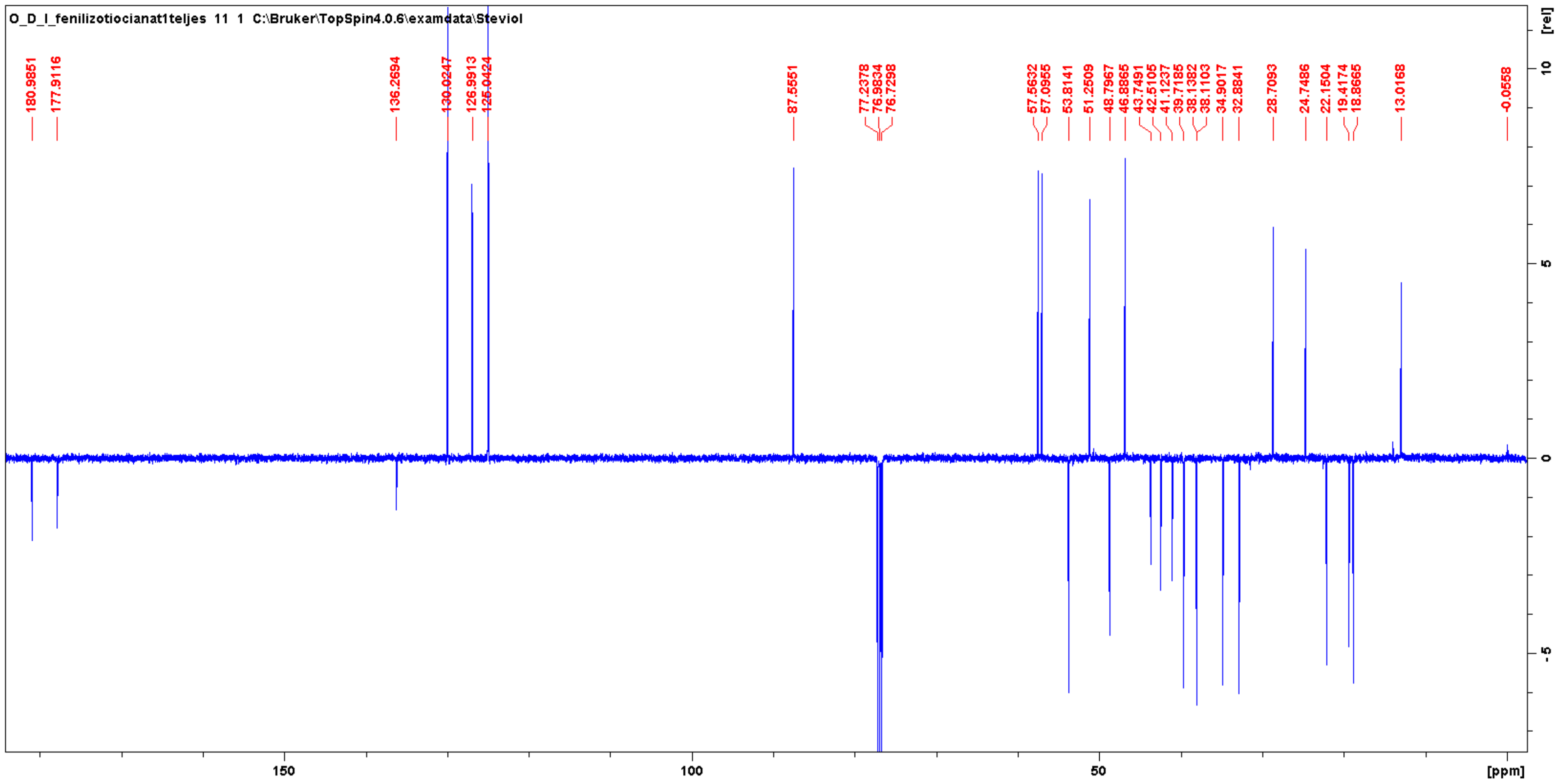

COSY of compound (4*R*,6a*S*,8*R*,9*S*,11b*S*)-Methyl 8-hydroxy-4,9,11b-trimethyl-7-((3-phenylthioureido)methyl)tetradecahydro-6a,9-methanocyclohepta[a]naphthalene-4-carboxylate (**25**)

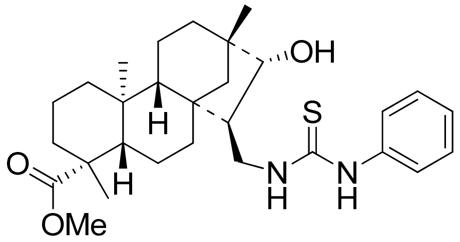

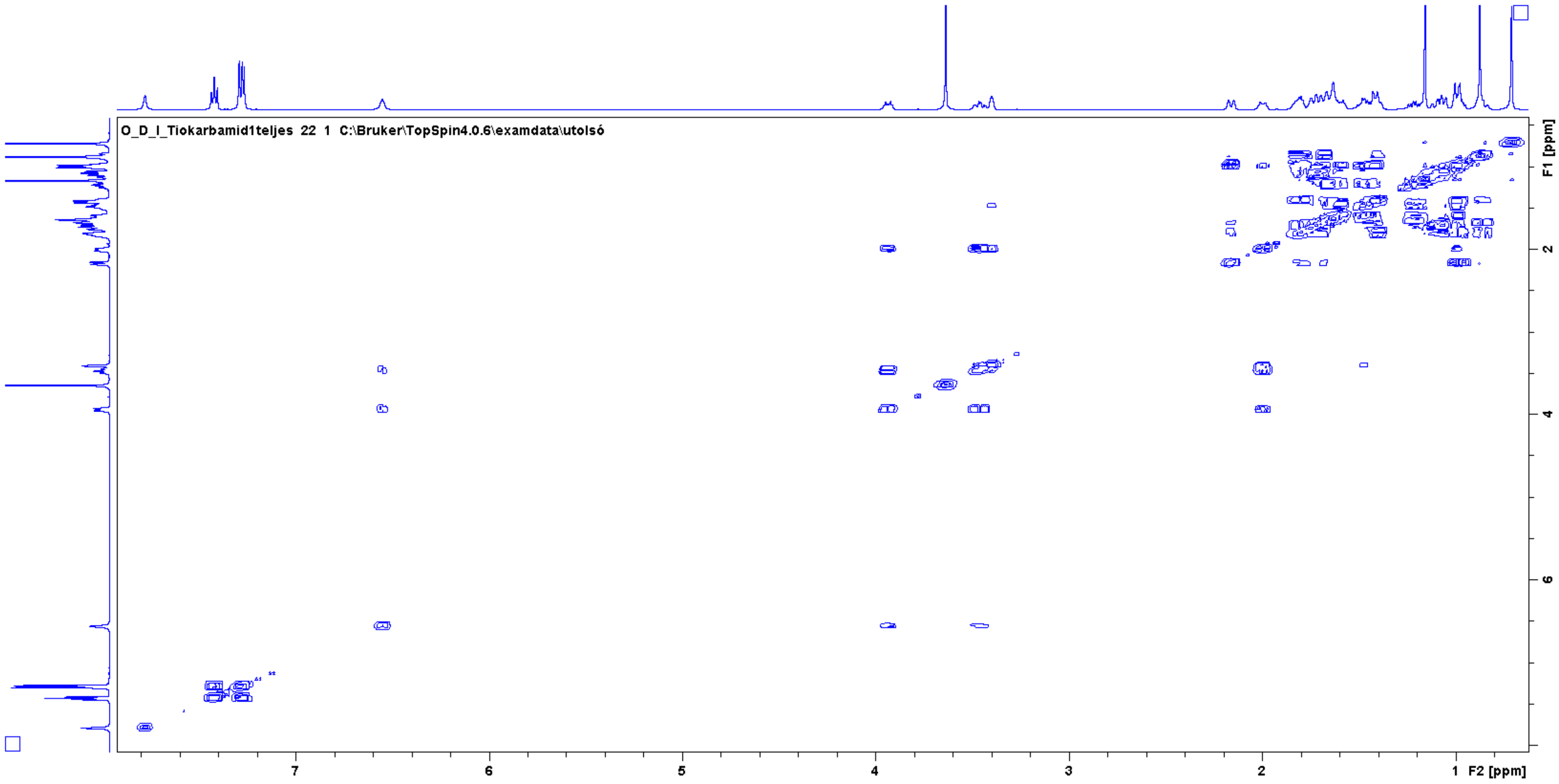

HSQC of compound (4*R*,6a*S*,8*R*,9*S*,11b*S*)-Methyl 8-hydroxy-4,9,11b-trimethyl-7-((3-phenylthioureido)methyl)tetradecahydro-6a,9-methanocyclohepta[a]naphthalene-4-carboxylate (**25**)

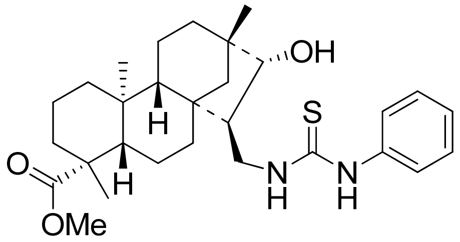

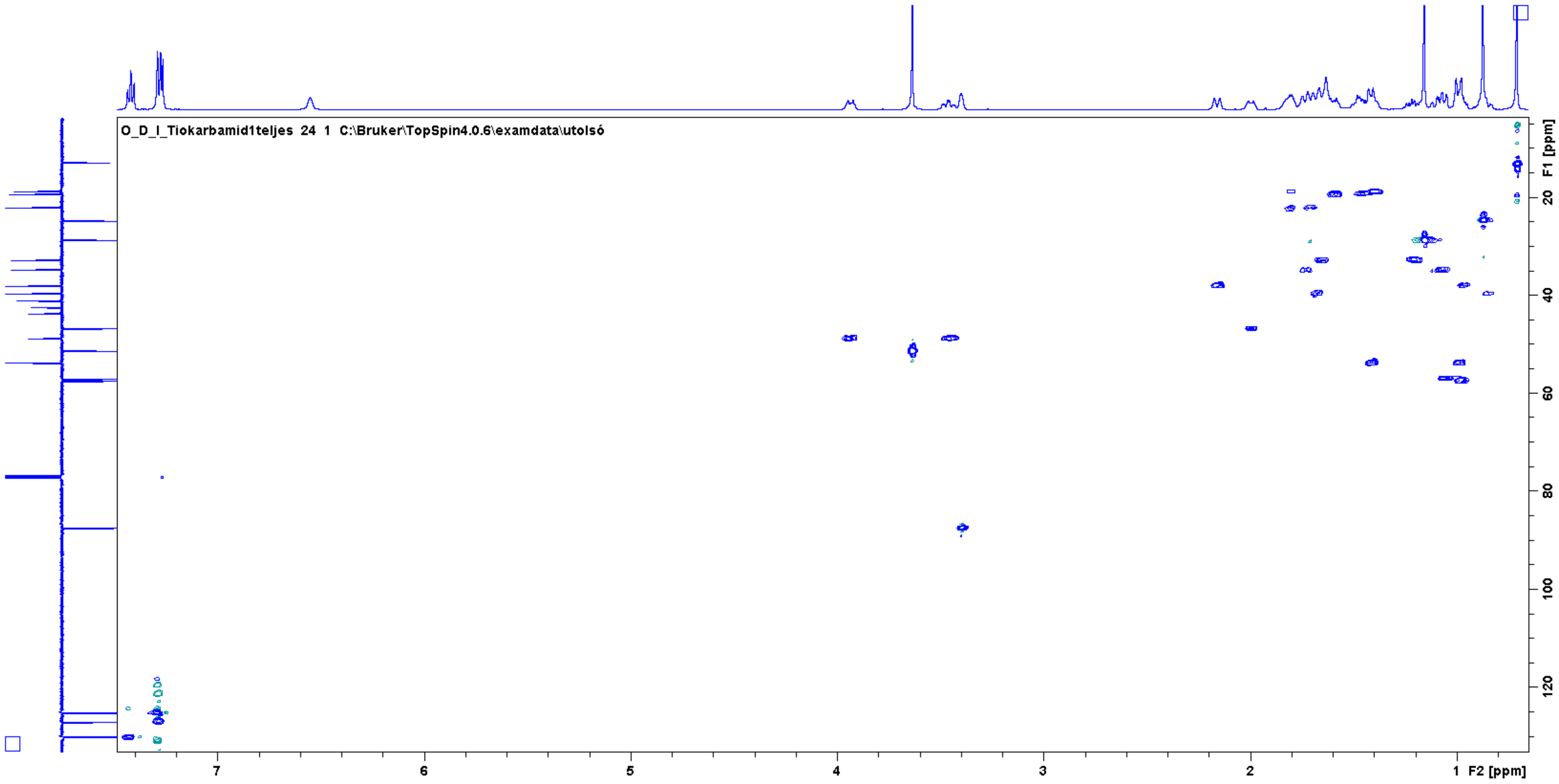

HMBC of compound (4*R*,6a*S*,8*R*,9*S*,11b*S*)-Methyl 8-hydroxy-4,9,11b-trimethyl-7-((3-phenylthioureido)methyl)tetradecahydro-6a,9-methanocyclohepta[a]naphthalene-4-carboxylate (**25**)

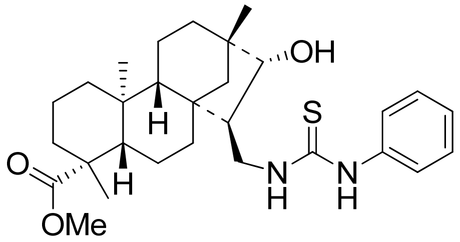

OH
S
O
H
H
N
H
N
H
OMe

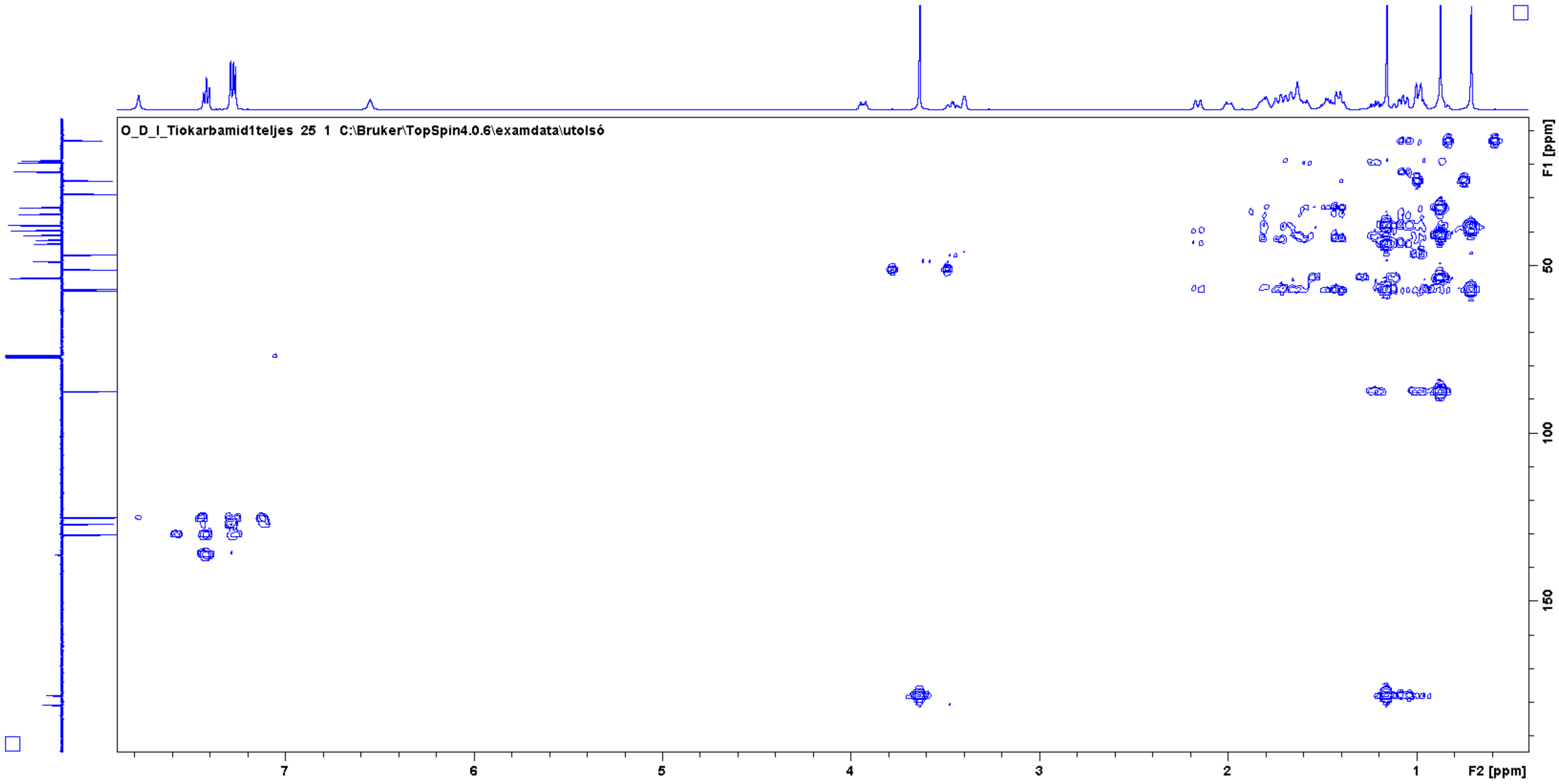

O_D_I_Tiokarbamid1teljes 25 1 C:\Bruker\TopSpin4.0.6\examdata\utolsó
F1 [ppm]
50
100
150
7
6
5
4
3
2
1
F2 [ppm]

^{1}H-NMR of compound (4*R*,6a*S*,8*R*,9*S*,11b*S*)-Methyl 8-hydroxy-4,9,11b-trimethyl-7-((((methylthio)(phenylimino)methyl)amino)methyl)tetradecahydro-6a,9-methanocyclohepta[a]naphthalene-4-carboxylate (**26**)

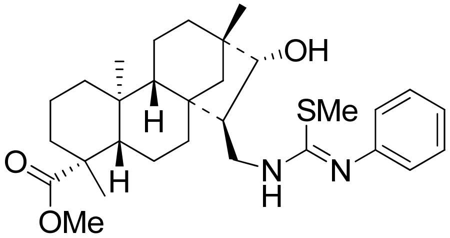

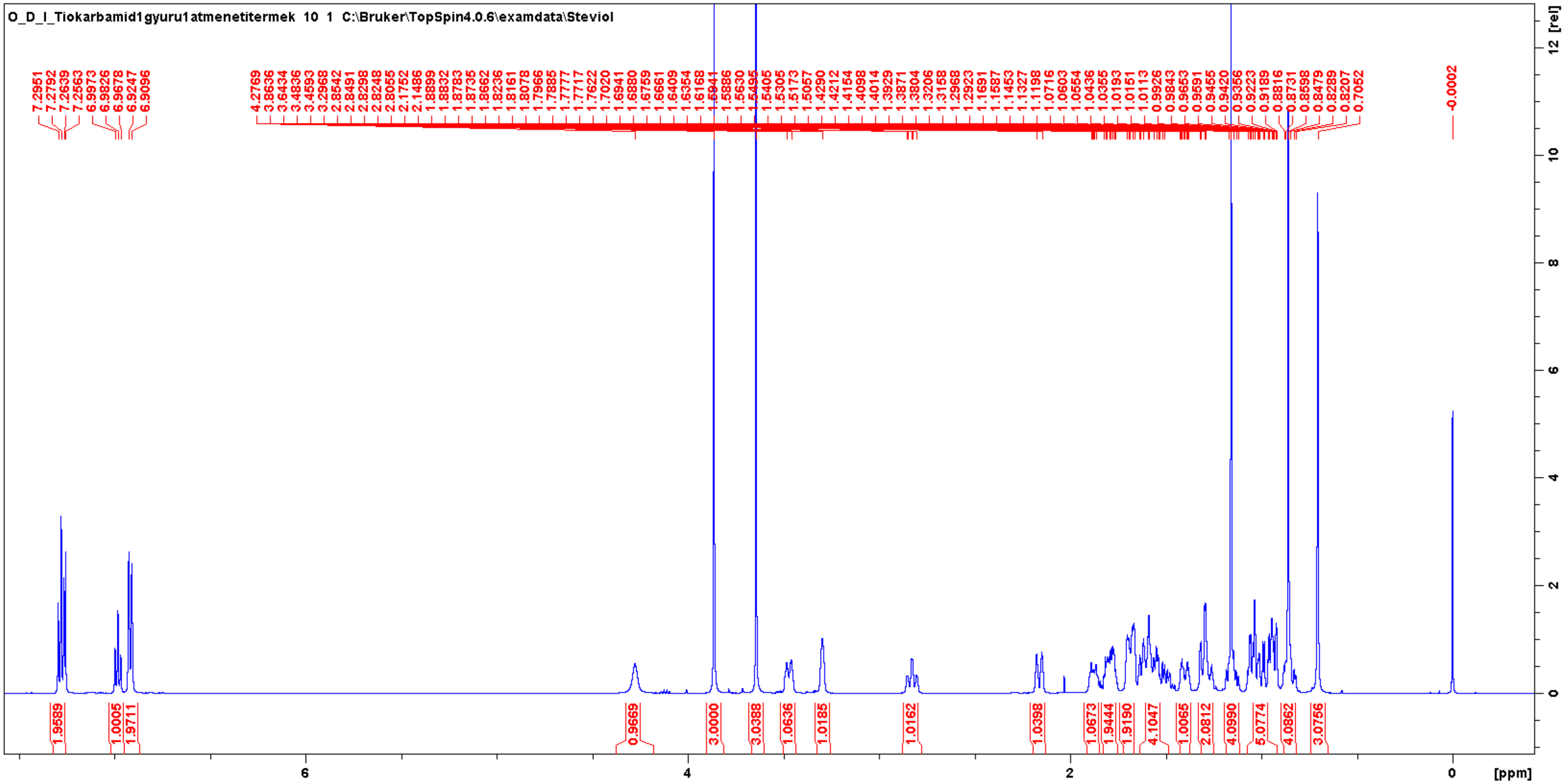

^{13}C-NMR of compound (4*R*,6a*S*,8*R*,9*S*,11b*S*)-Methyl 8-hydroxy-4,9,11b-trimethyl-7-((((methylthio)(phenylimino)methyl)amino)methyl)tetradecahydro-6a,9-methanocyclohepta[a]naphthalene-4-carboxylate (**26**)

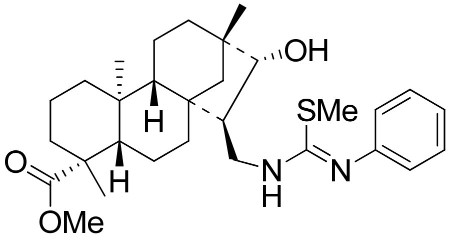

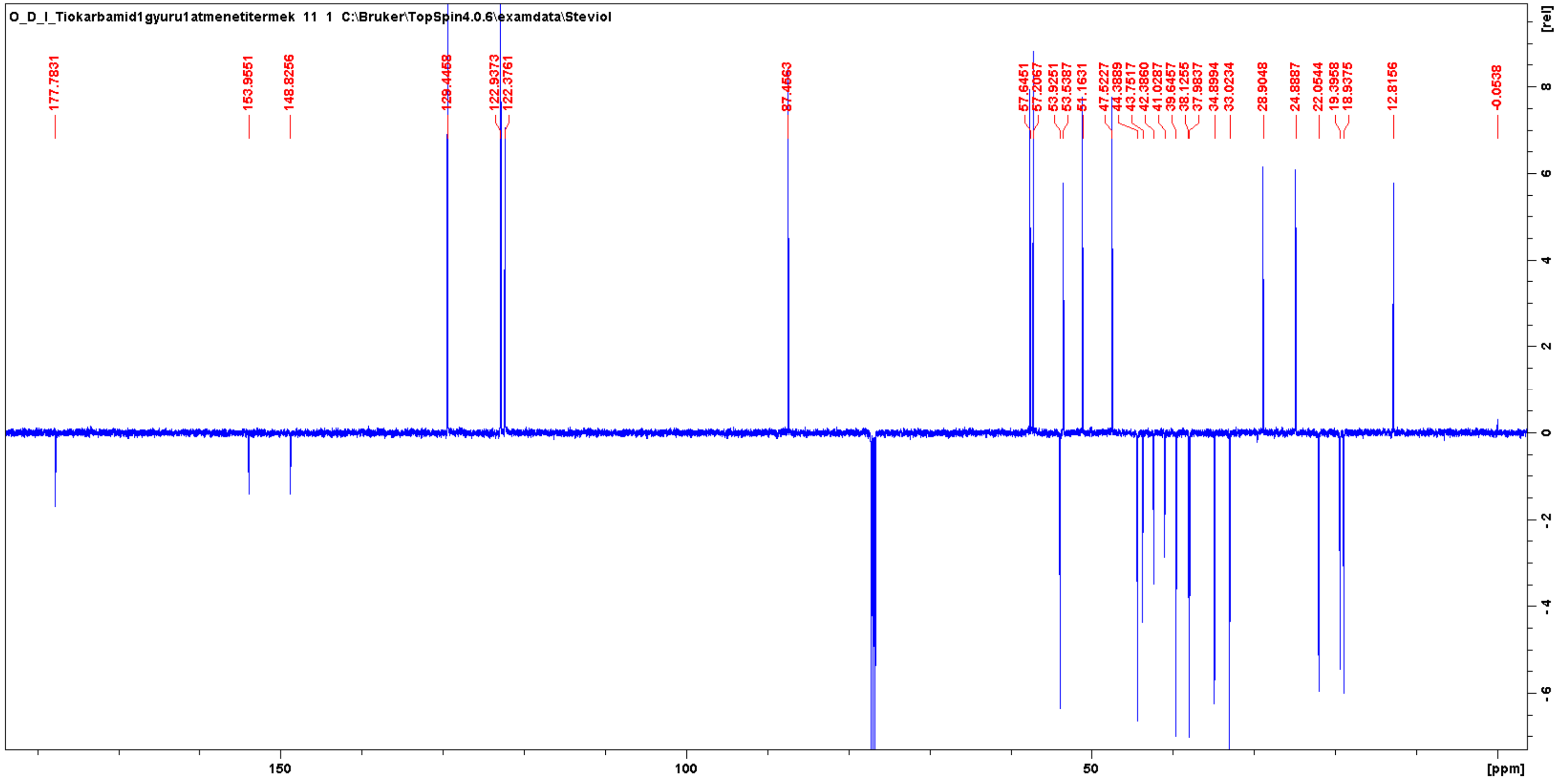

COSY of compound (4*R*,6a*S*,8*R*,9*S*,11b*S*)-Methyl 8-hydroxy-4,9,11b-trimethyl-7-((((methylthio)(phenylimino)methyl)amino)methyl)tetradecahydro-6a,9-methanocyclohepta[a]naphthalene-4-carboxylate (**26**)

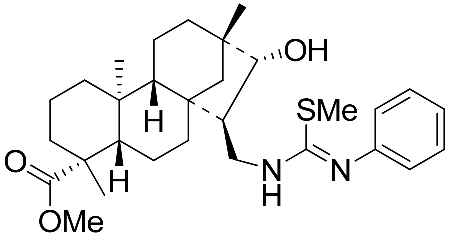

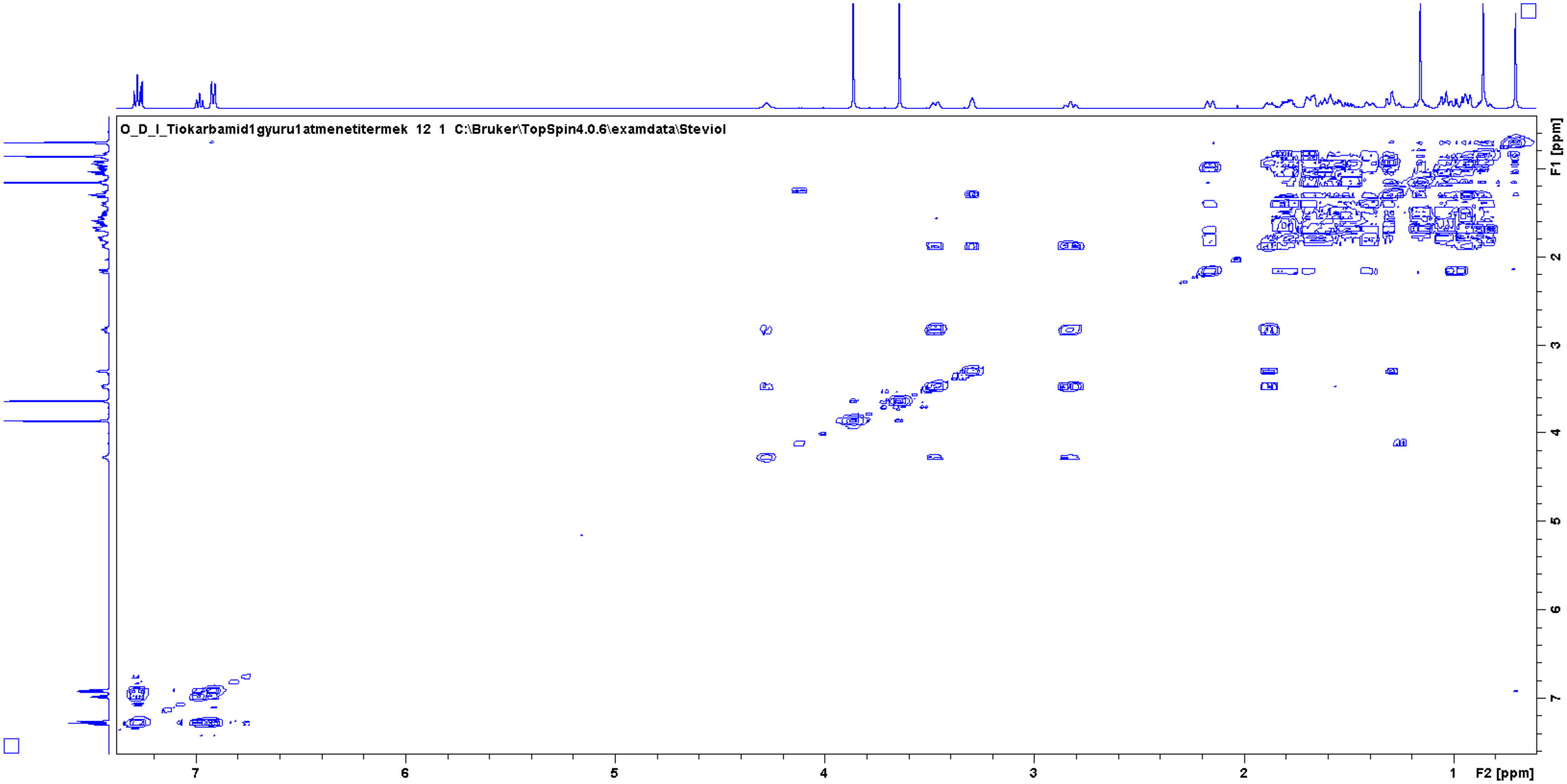

NOESY of compound (4*R*,6a*S*,8*R*,9*S*,11b*S*)-Methyl 8-hydroxy-4,9,11b-trimethyl-7-((((methylthio)(phenylimino)methyl)amino)methyl)tetradecahydro-6a,9-methanocyclohepta[a]naphthalene-4-carboxylate (**26**)

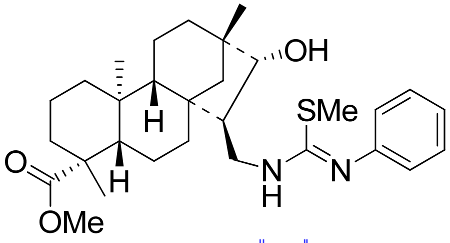

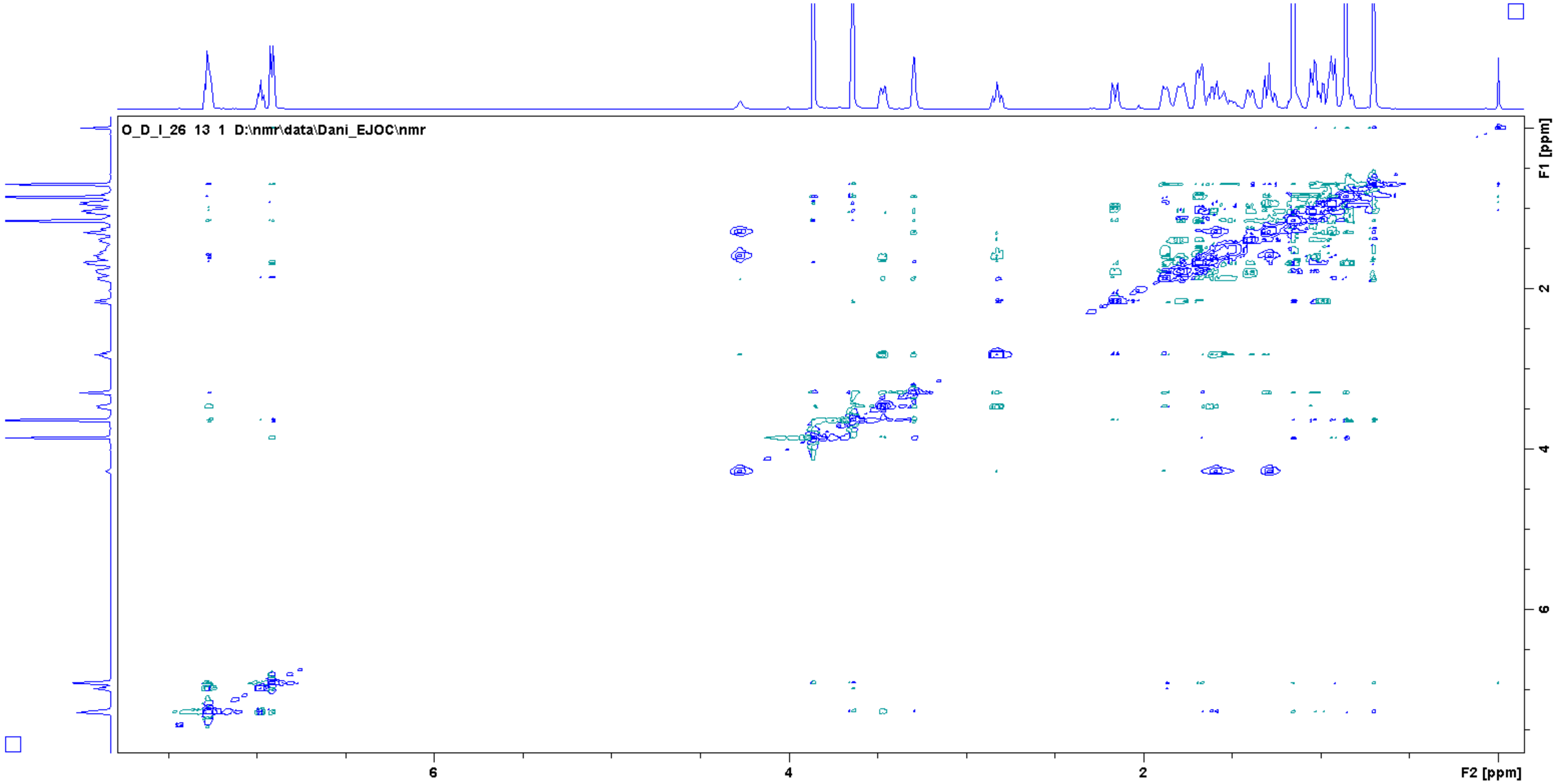

HSQC of compound (4*R*,6a*S*,8*R*,9*S*,11b*S*)-Methyl 8-hydroxy-4,9,11b-trimethyl-7-((((methylthio)(phenylimino)methyl)amino)methyl)tetradecahydro-6a,9-methanocyclohepta[a]naphthalene-4-carboxylate (**26**)

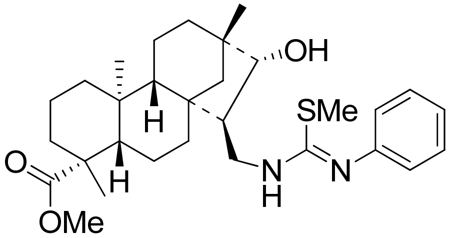

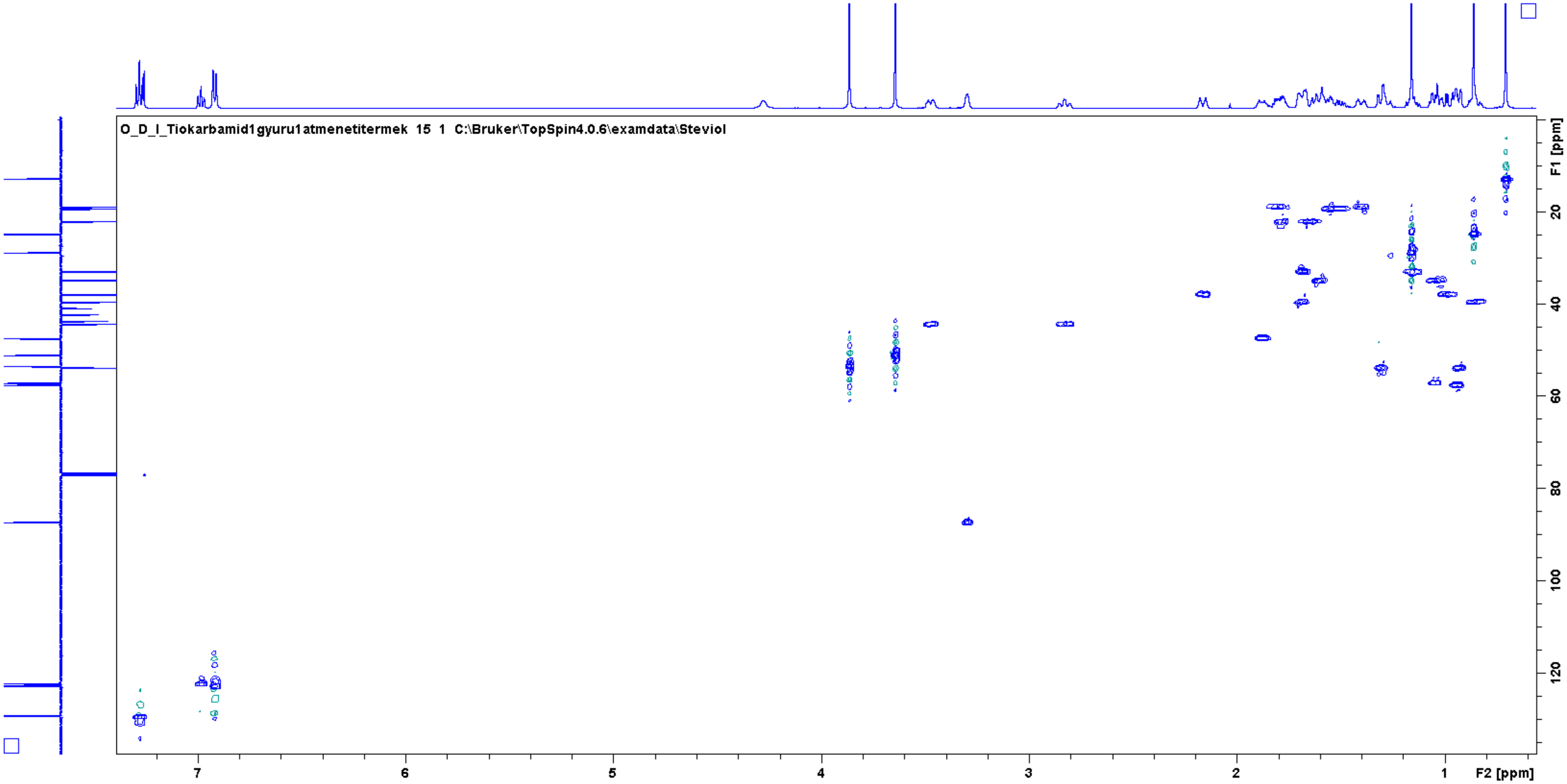

HMBC of compound (4*R*,6a*S*,8*R*,9*S*,11b*S*)-Methyl 8-hydroxy-4,9,11b-trimethyl-7-((((methylthio)(phenylimino)methyl)amino)methyl)tetradecahydro-6a,9-methanocyclohepta[a]naphthalene-4-carboxylate (**26**)

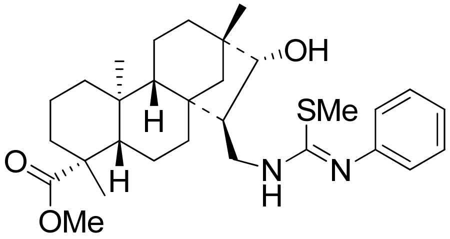

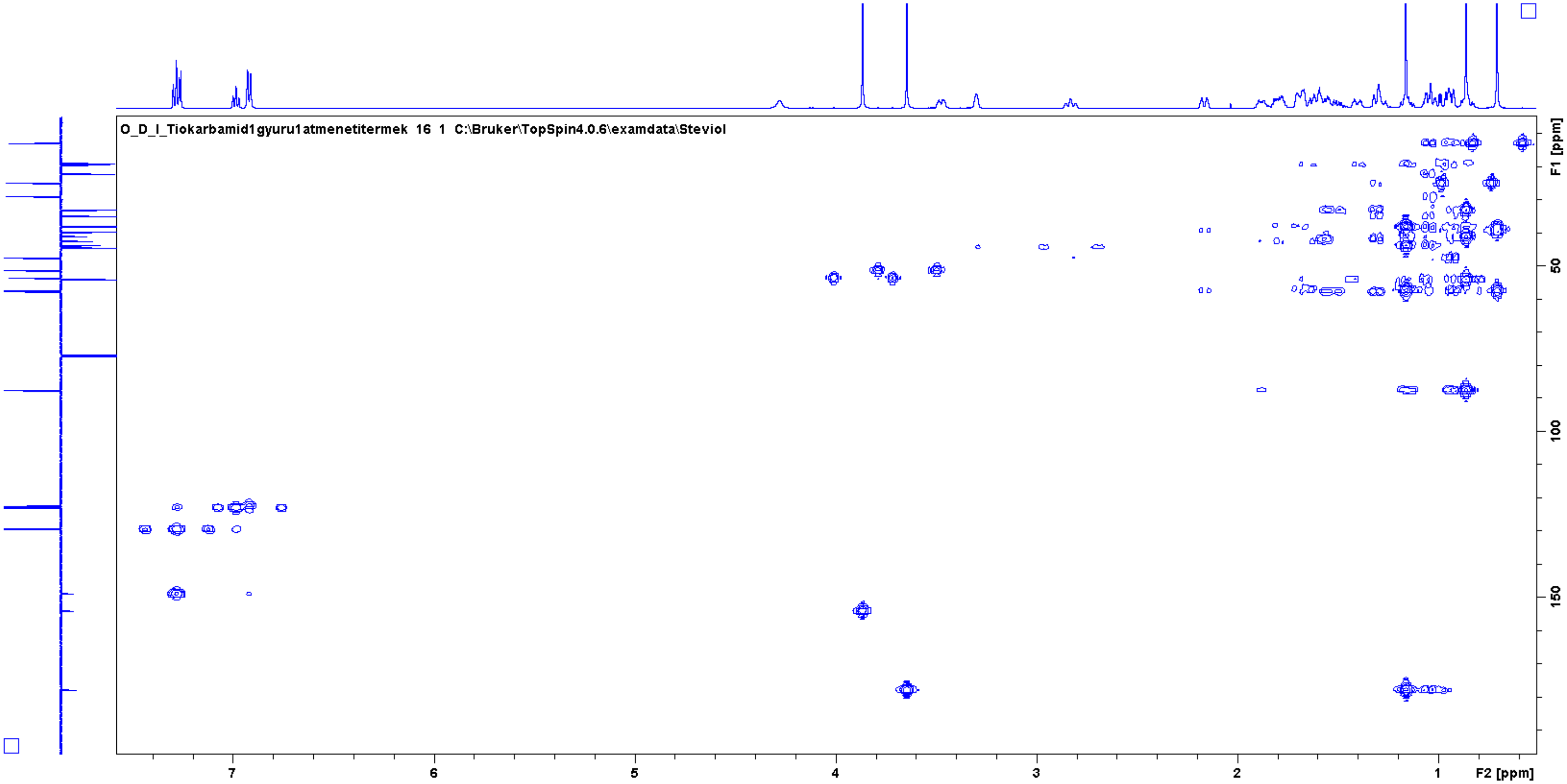

^{1}H-NMR of compound (4*R*,6a*S*,8*R*,9*S*,11b*S*)-Methyl 8-hydroxy-4,9,11b-trimethyl-7-((((ethylthio)(phenylimino)methyl)amino)methyl)tetradecahydro-6a,9-methanocyclohepta[a]naphthalene-4-carboxylate (**28**)

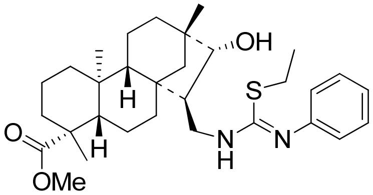

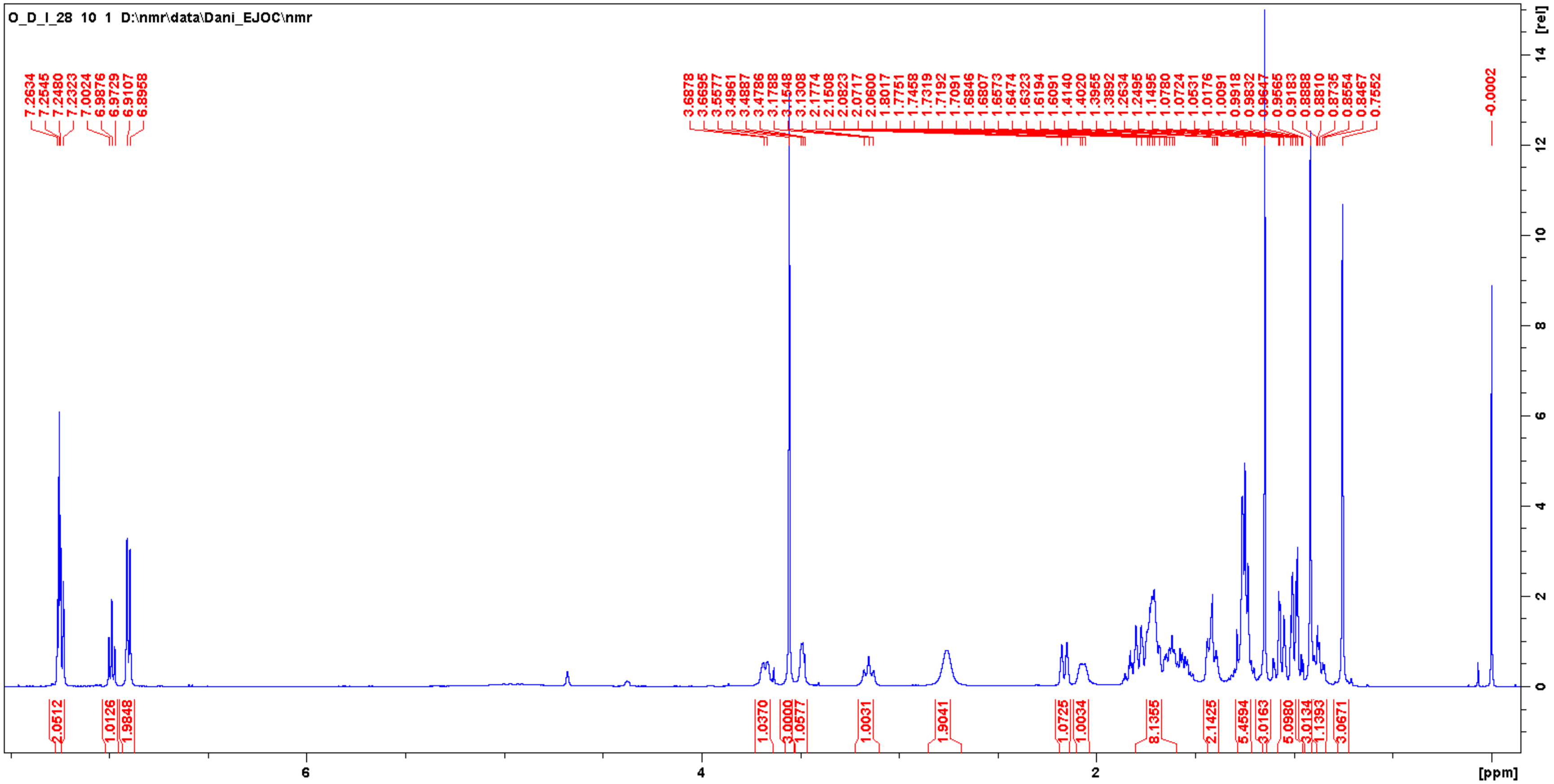

^{13}C-NMR of compound (4*R*,6a*S*,8*R*,9*S*,11b*S*)-Methyl 8-hydroxy-4,9,11b-trimethyl-7-((((ethylthio)(phenylimino)methyl)amino)methyl)tetradecahydro-6a,9-methanocyclohepta[a]naphthalene-4-carboxylate (**28**)

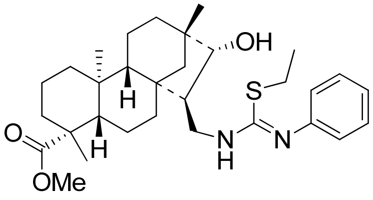

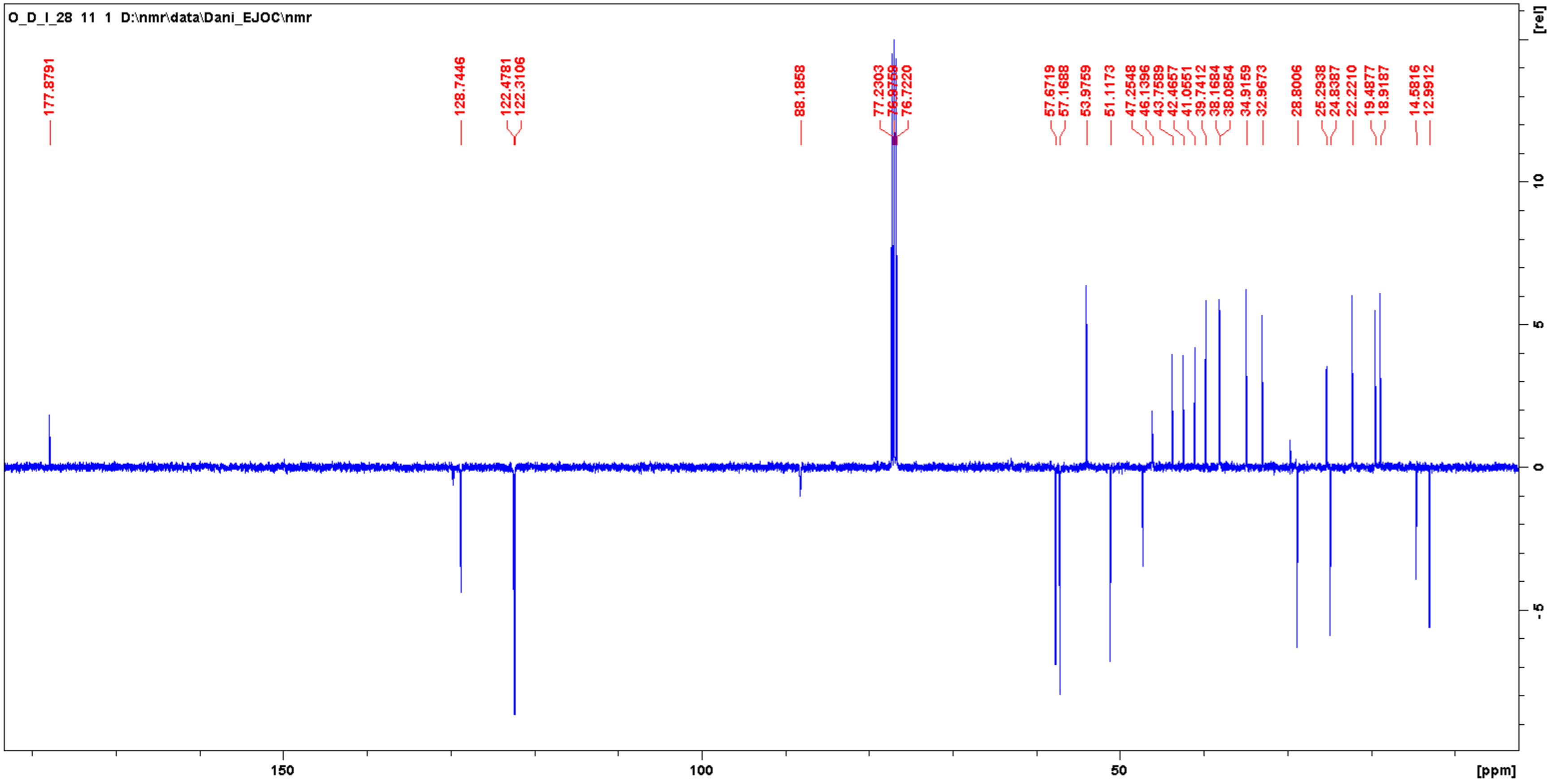

COSY of compound (4*R*,6a*S*,8*R*,9*S*,11b*S*)-Methyl 8-hydroxy-4,9,11b-trimethyl-7-((((ethylthio)(phenylimino)methyl)amino)methyl)tetradecahydro-6a,9-methanocyclohepta[a]naphthalene-4-carboxylate (**28**)

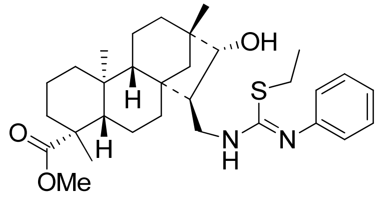

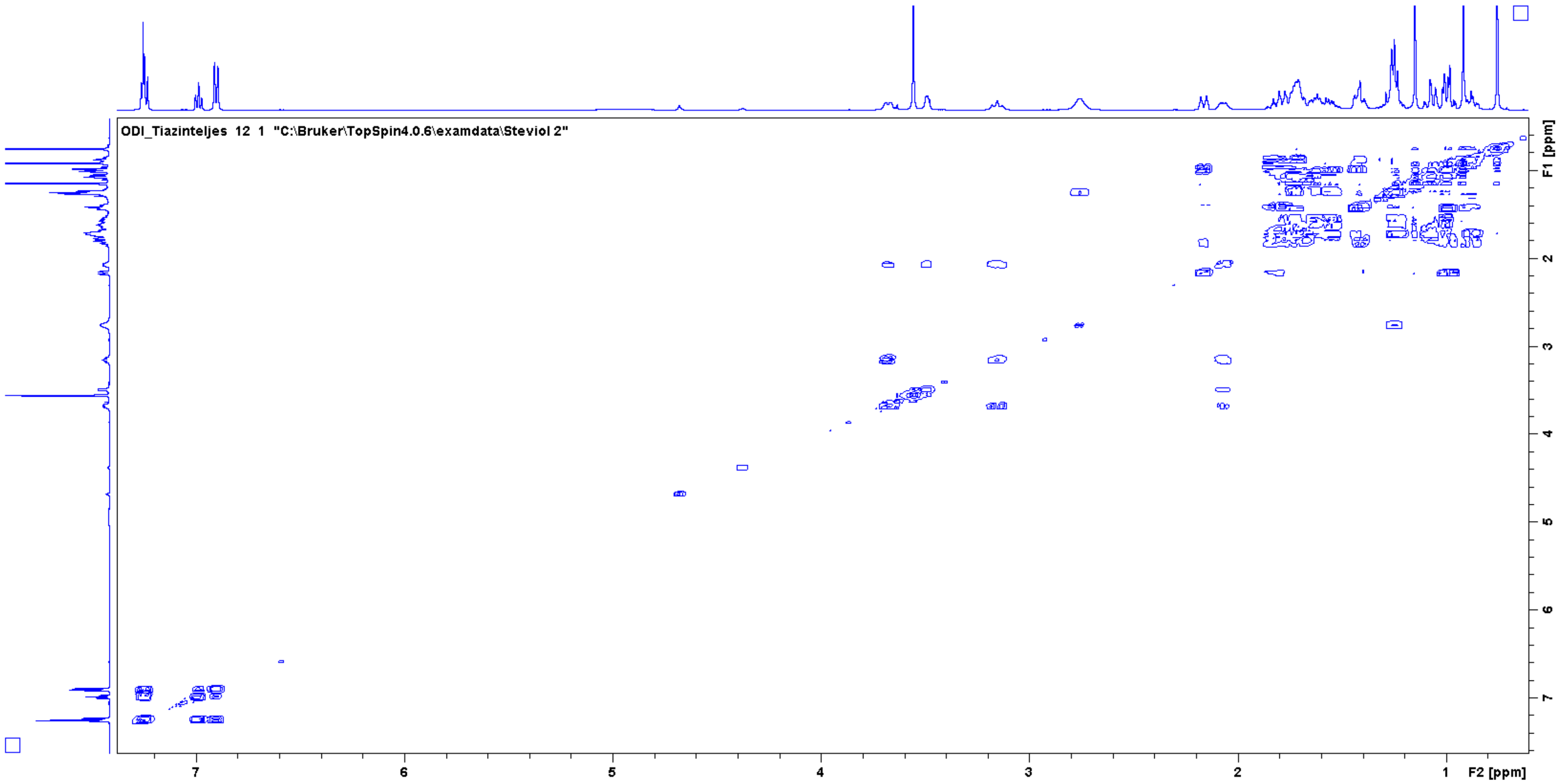

NOESY of compound (4*R*,6a*S*,8*R*,9*S*,11b*S*)-Methyl 8-hydroxy-4,9,11b-trimethyl-7-((((ethylthio)(phenylimino)methyl)amino)methyl)tetradecahydro-6a,9-methanocyclohepta[a]naphthalene-4-carboxylate (**28**)

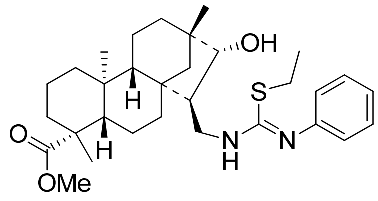

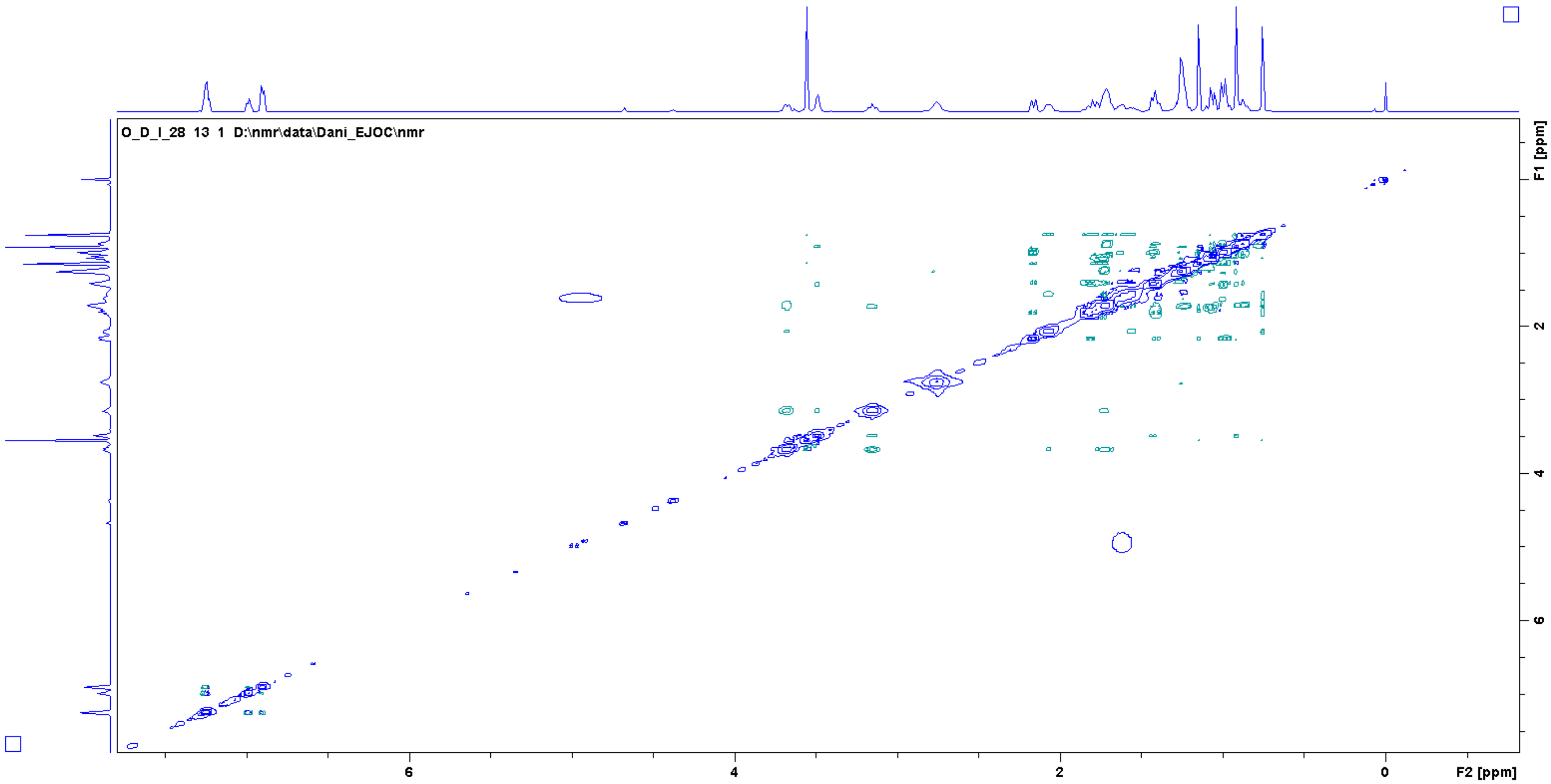

HSQC of compound (4*R*,6a*S*,8*R*,9*S*,11b*S*)-Methyl 8-hydroxy-4,9,11b-trimethyl-7-((((ethylthio)(phenylimino)methyl)amino)methyl)tetradecahydro-6a,9-methanocyclohepta[a]naphthalene-4-carboxylate (**28**)

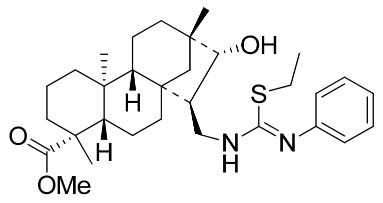

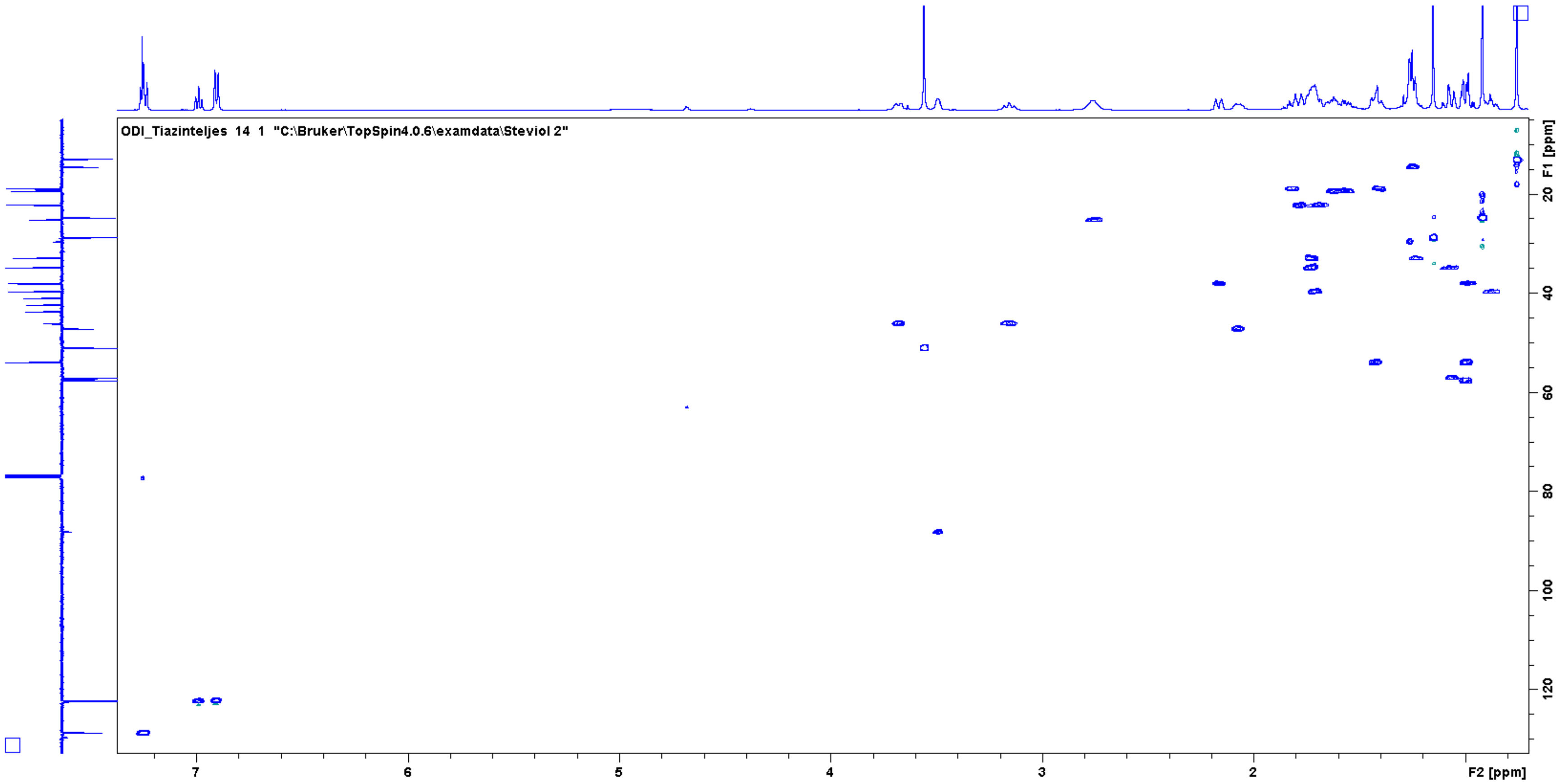

HMBC of compound (4*R*,6a*S*,8*R*,9*S*,11b*S*)-Methyl 8-hydroxy-4,9,11b-trimethyl-7-((((ethylthio)(phenylimino)methyl)amino)methyl)tetradecahydro-6a,9-methanocyclohepta[a]naphthalene-4-carboxylate (**28**)

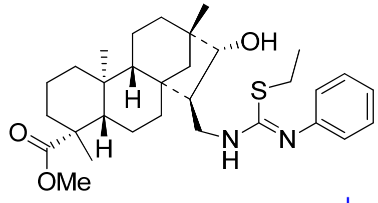

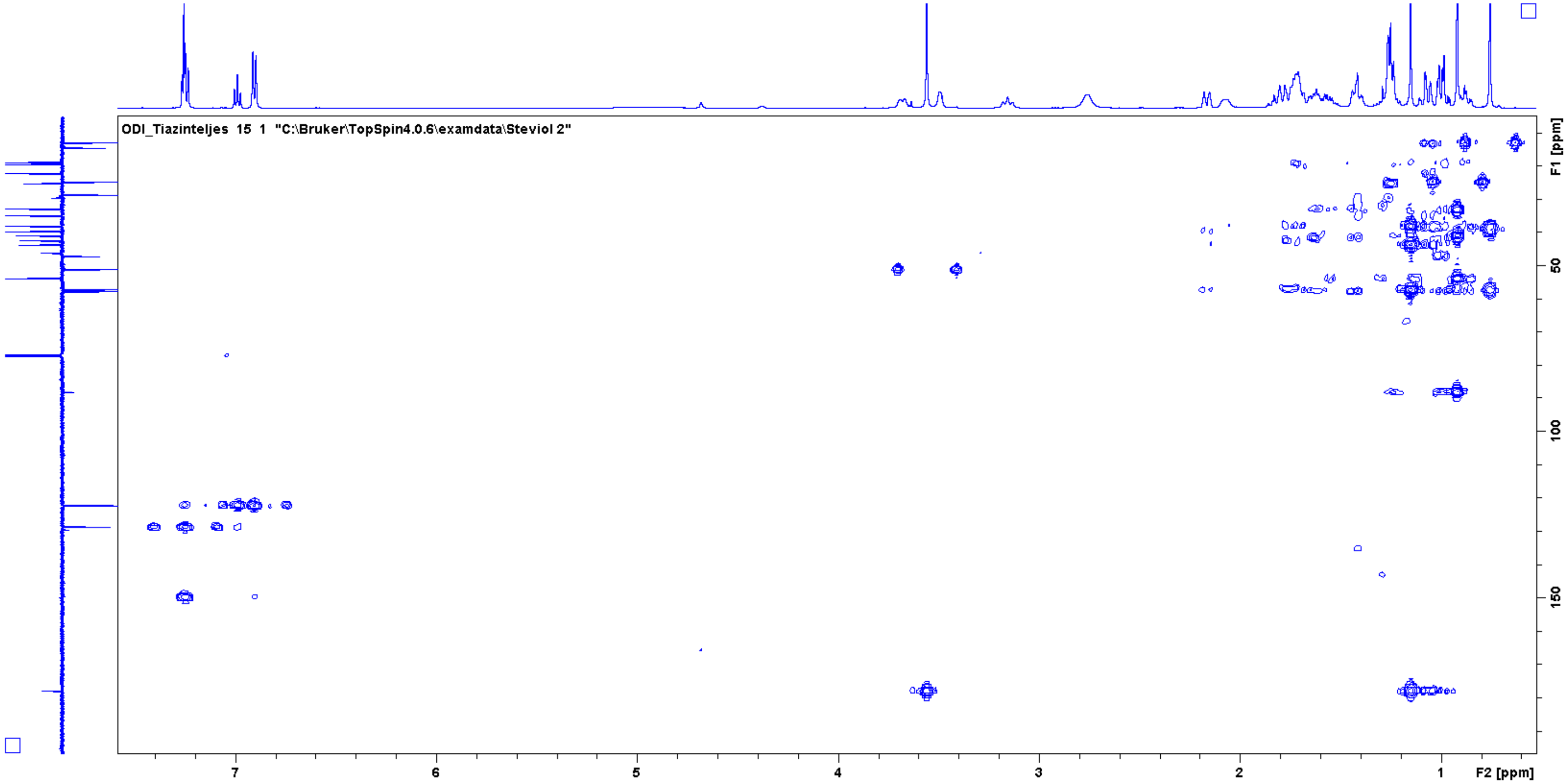

Supplement: Supplementary file 1 [file ijms-22-11232-s001.zip › ijms-1405355-supplementary.pdf]
